# Synthesis and kinetic resolution of substituted tetrahydroquinolines by lithiation then electrophilic quench†
†Electronic supplementary information (ESI) available: Experimental details, spectroscopic data, ReactIR, X-ray and DFT data, and NMR spectra. For products **3a**, **3g**, **10a**, and **11**. CCDC 1578266–1578269. For ESI and crystallographic data in CIF or other electronic format see DOI: 10.1039/c7sc04435f


**DOI:** 10.1039/c7sc04435f

**Published:** 2017-12-14

**Authors:** Nicholas Carter, Xiabing Li, Lewis Reavey, Anthony J. H. M. Meijer, Iain Coldham

**Affiliations:** a Department of Chemistry , University of Sheffield , Brook Hill , Sheffield , S3 7HF , UK . Email: i.coldham@sheffield.ac.uk

## Abstract

Treatment of *N*-Boc-2-aryl-1,2,3,4-tetrahydroquinolines with *n*-butyllithium in THF at –78 °C resulted in efficient lithiation at the 2-position and the organolithiums were trapped with a variety of electrophiles to give substituted products. Variable temperature NMR spectroscopy gave kinetic data that showed that the rate of *tert*-butoxycarbonyl (Boc) rotation was fast (Δ*G*^‡^ ≈ 45 kJ mol^–1^ at –78 °C) and *in situ* ReactIR spectroscopy showed fast lithiation at –78 °C. By carrying out the lithiation in the presence of the chiral ligand sparteine, kinetic resolutions with very high levels of enantioselectivity were achieved. The resulting enantioenriched *N*-Boc-2-aryltetrahydroquinolines were converted to 2,2-disubstituted products without significant loss in enantiopurity. Most electrophiles add at the 2-position and the chemistry provides a way to access tetrahydroquinolines that are fully substituted alpha to the nitrogen atom. Notably, either enantiomer of the 2,2-disubstituted tetrahydroquinolines can be obtained with high selectivity from the same enantiomer of the chiral ligand. Unusually, when methyl cyanoformate was used as the electrophile, substitution occurred in the ortho position of the aryl ring attached at C-2. This change in regioselectivity on changing the electrophile was probed by deuterium isotope studies and by DFT calculations which suggested that the binding of the cyanoformate altered the structure of the intermediate organolithium. Secondary amine products can be prepared by removing the Boc group with acid or by inducing the Boc group to rearrange to the 2-position in the presence of triethylborane and this carbonyl N-to-C rearrangement occurs with retention of configuration from the intermediate enantiomerically enriched organolithium species.

## Abstract 2

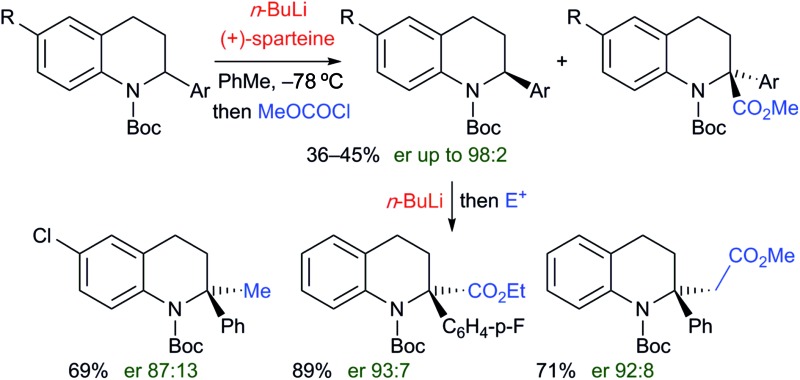
Treatment of *N*-Boc-2-aryl-1,2,3,4-tetrahydroquinolines with *n*-butyllithium in THF at –78 °C resulted in efficient lithiation at the 2-position and the organolithiums were trapped with a variety of electrophiles to give substituted products.

## Introduction

**Scheme 1:**
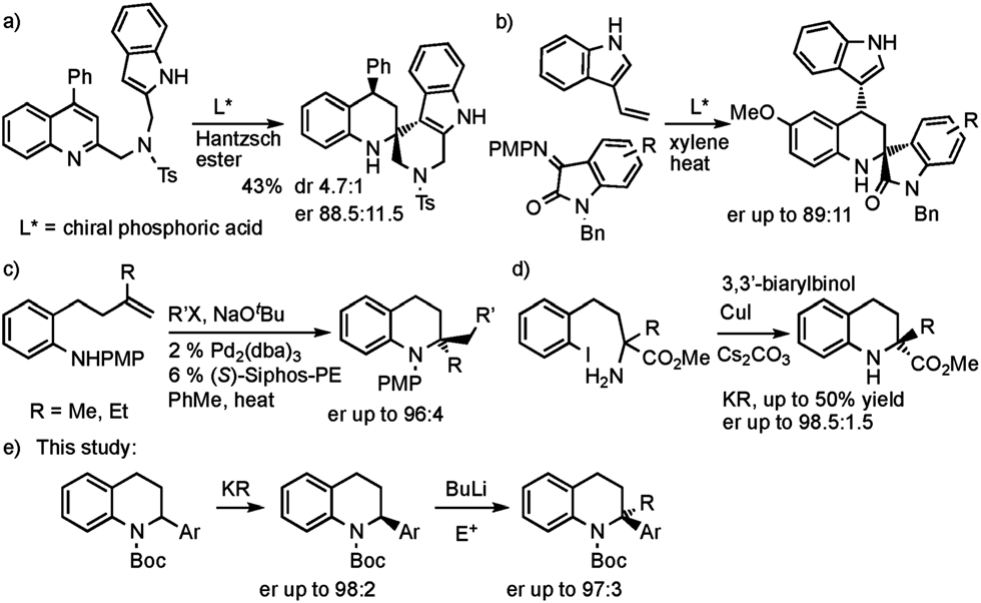
Asymmetric methods to 2,2-disubstituted tetrahydroquinolines.

The tetrahydroquinoline ring system is of great importance in natural products and medicinal compounds.^1^ Substituted 1,2,3,4-tetrahydroquinolines are present in alkaloids such as angustureine, cuspareine, galipinine, martinellic acid, virantmycin, many with (for example) antiviral, antibacterial, antifungal, antimalarial, or antitumour activities.^1^ The majority of syntheses of tetrahydroquinolines involve the reduction of quinolines or dihydroquinolines,^2^ a Povarov type reaction,^3^ or a cyclization process to make one of the bonds in the partially saturated ring.^4^ This often leads to tetrahydroquinolines that are monosubstituted at positions in the partially saturated ring, for example at C-2, rather than 2,2-disubstituted products. The ability to prepare 2,2-disubstituted tetrahydroquinolines would be attractive, opening up a greater diversity of products and exploring more chemical space. However, there are few examples of their enantioselective preparation from achiral compounds. You and co-workers reported an isolated example of an asymmetric reduction of a substituted quinoline and intramolecular trapping with an indole (Scheme 1a).^5^ Zhao, Shi and co-workers developed an asymmetric Povarov reaction (Scheme 1b).^6^ Hopkins and Wolfe reported a palladium catalyzed carboamination to give 2,2-dialkyl tetrahydroquinolines (Scheme 1c).4*d* Enantioselective intramolecular *N*-arylation by Cai and co-workers was found with copper catalysis (Scheme 1d).^7^ Here we report the use of deprotonation followed by electrophile trapping as a convenient approach to 2,2-disubstituted tetrahydroquinolines (Scheme 1e). This strategy has found only limited use in a racemic sense with 2-cyanotetrahydroquinolines.^8^ Our research group has been studying the lithiation then electrophilic quench of *N*-Boc heterocycles, with a particular recent focus on piperidines,^9^ and tetrahydroisoquinolines.^10^,^11^ We show in this study that we can extend our lithiation chemistry with *N*-Boc-2-aryl-heterocycles to tetrahydroquinolines. These substrates have been found to undergo highly enantioselective kinetic resolution.^12^,^13^ The organolithium is configurationally stable at –78 °C and can be trapped to give 2,2-disubstituted tetrahydroquinolines with excellent enantioselectivities.

## Results and discussion

**Scheme 2:**
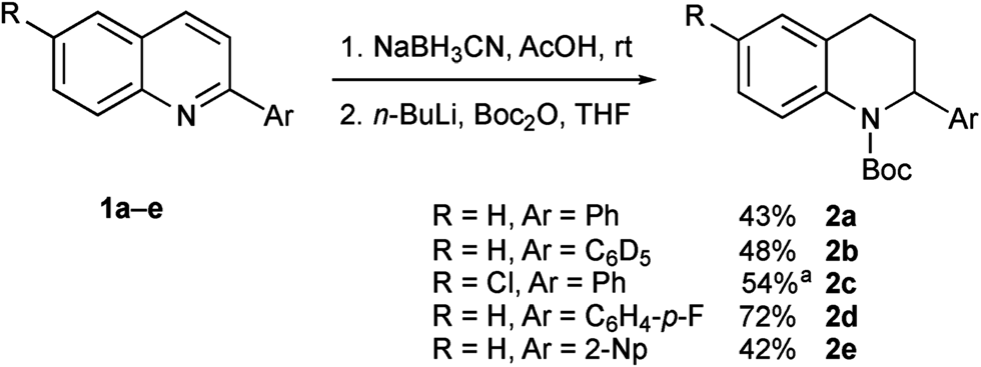
Preparation of tetrahydroquinolines **2a–e**. ^a^Boc-ON rather than Boc_2_O was used.

We needed access to *N*-Boc-2-aryltetrahydroquinolines to test our lithiation chemistry. These were prepared from the quinolines **1a–e** by reduction with sodium cyanoborohydride in acetic acid using a known method,^14^ followed by Boc protection of the amine to give the novel products **2a–e** (Scheme 2).

**Fig. 1:**
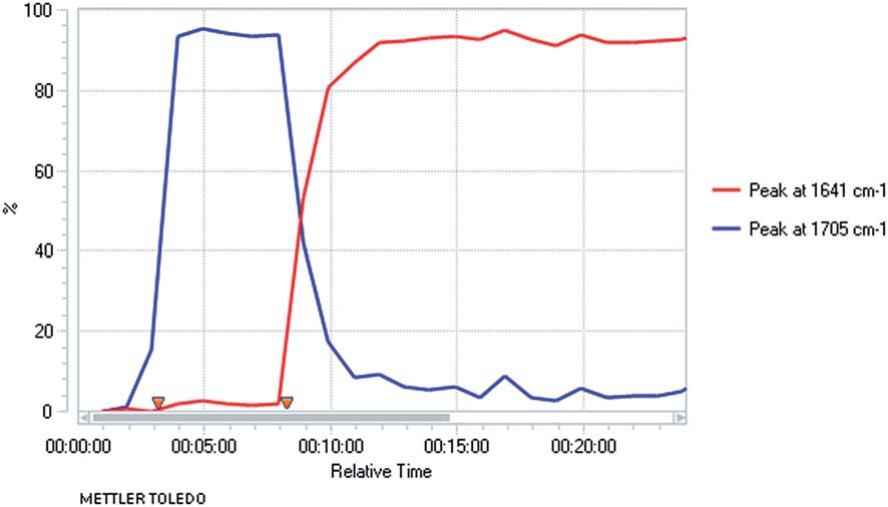
*In situ* IR spectroscopy of the deprotonation of **2a** with *n*-BuLi, THF at –78 °C with time in h:min:s with *n*-BuLi added at time 8 min (*ν*_C=O_**2a** 1705 cm^–1^, *ν*_C=O_ lithiated **2a** 1641 cm^–1^).

We were aware from earlier studies that the rate of lithiation is dependent on the orientation of the Boc group.9*d* Therefore we probed the rate of rotation of the Boc group in compound **2a** by using VT-NMR spectroscopy (see ESI†) and found activation parameters for Boc rotation, giving Δ*G*^‡^ ≈ 47 kJ mol^–1^ at –78 °C for each rotamer. This suggests that the Boc group rotates quickly (*t*_1/2_ ≈ 1 s) even at –78 °C. This was confirmed by ReactIR spectroscopy, which showed rapid lithiation (within a few minutes) at this temperature (Fig. 1 and ESI†).

**Scheme 3:**
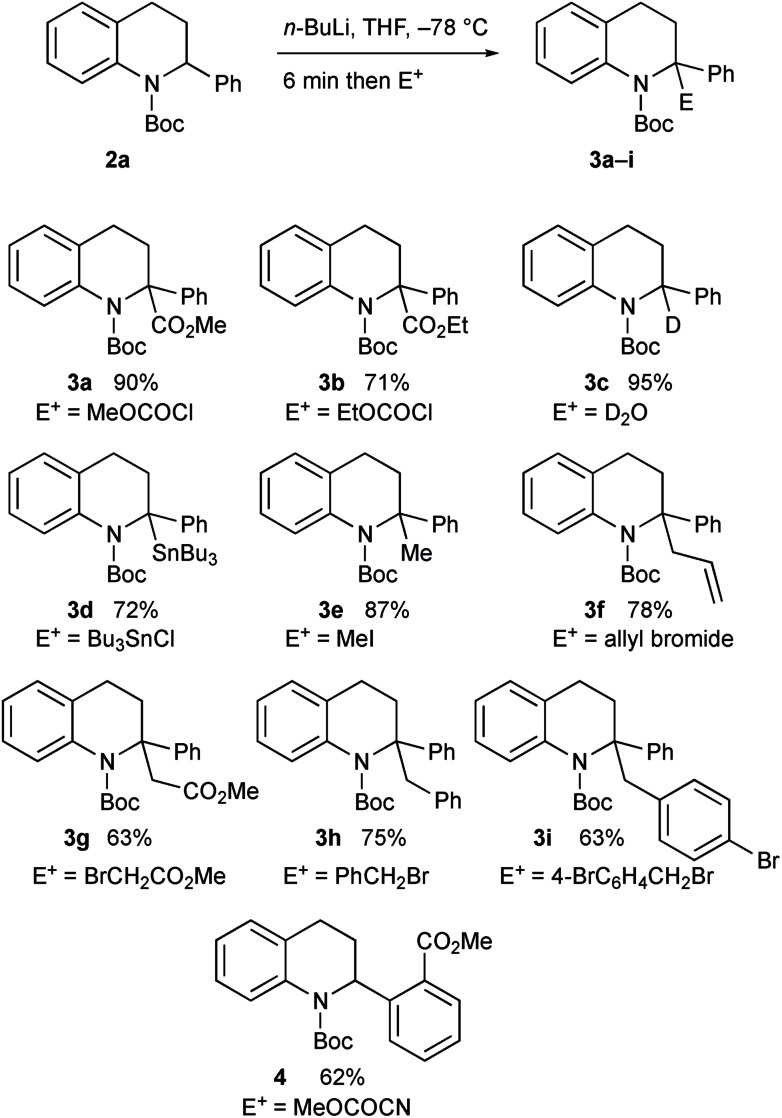
Lithiation–trapping of tetrahydroquinoline **2a**.

The intermediate organolithium could be quenched with a selection of electrophiles to give the 2,2-disubstituted tetrahydroquinoline products **3a–i** (Scheme 3). Generally good yields of the products were obtained. The only exception to this was the use of the electrophile methyl cyanoformate, which gave the product **4** rather than the expected product **3a**. This is discussed further below.

**Scheme 4:**
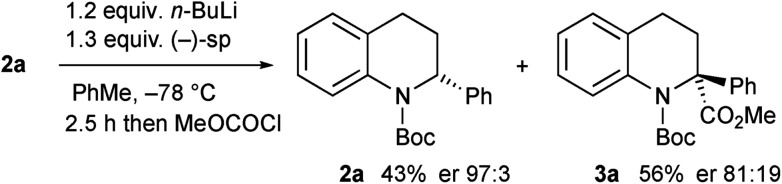
Kinetic resolution of tetrahydroquinoline **2a** with (–)-sparteine.

Kinetic resolution of the tetrahydroquinoline **2a** was studied using (–)-sparteine as the chiral ligand in PhMe and adding *n*-BuLi to this mixture.^15^ Moderately good enantiomer ratios of the recovered tetrahydroquinoline **2a** and the product **3a** were obtained by this method. However improved results were found by pre-mixing the *n*-BuLi and (–)-sparteine before adding the tetrahydroquinoline **2a** (Scheme 4). The deprotonation was relatively slow under these conditions and despite being a kinetic resolution, it was best to use 1.2 equiv. *n*-BuLi to achieve a suitable rate of reaction. The recovered tetrahydroquinoline **2a** was isolated with high enantiomer ratio (er 97 : 3) and this equates to a selectivity factor (*k*_rel_) = 20.

**Scheme 5:**
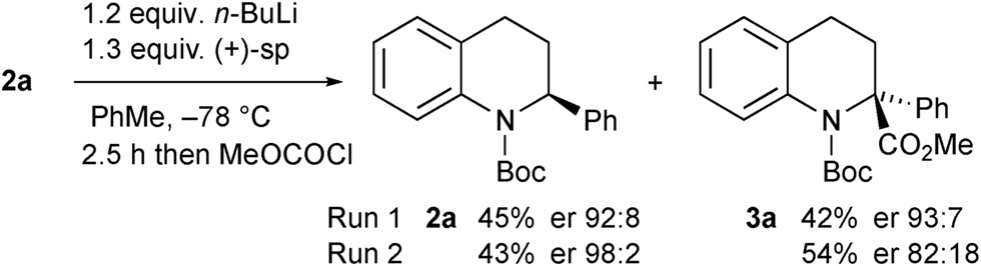
Kinetic resolution of tetrahydroquinoline **2a** with (+)-sparteine.

**Scheme 6:**
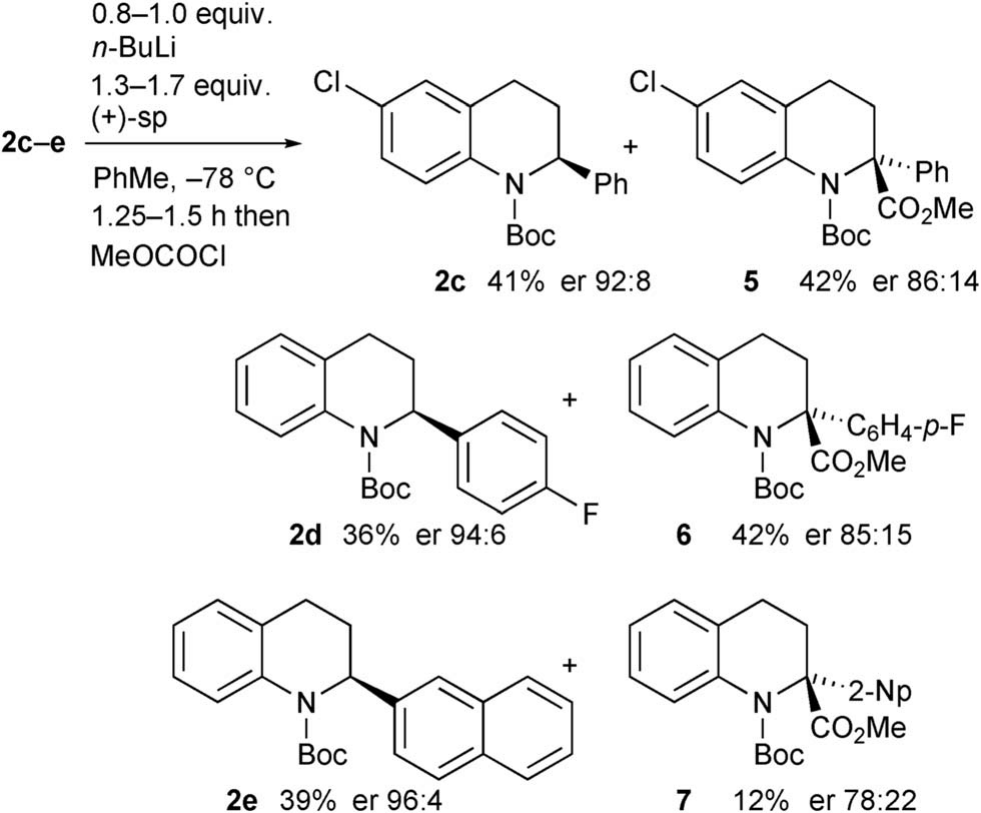
Kinetic resolution of tetrahydroquinolines **2c–e** with (+)-sparteine.

The opposite enantiomer of the recovered starting material **2a** could be obtained by using (+)-sparteine in the kinetic resolution (Scheme 5). Several runs were conducted, all with very good enantioselectivities. Recrystallisation of the product **3a** from run 1 gave material that was suitable for single crystal X-ray analysis. This confirmed the absolute configuration to be (*R*)-**3a** as indicated (see ESI†), and as expected based on previous findings of the stereoselectivity preference for BuLi·sparteine in the deprotonation of *N*-Boc-piperidines.^15^,^16^ The kinetic resolution was extended to the tetrahydroquinolines **2c–e** (Scheme 6). High enantiomer ratios of the recovered tetrahydroquinolines were obtained in each case, particularly if more than just a slight excess of sparteine was added to the reaction mixture. The quenched products could be separated from the recovered starting material to give the desired tetrahydroquinolines (*S*)-**2c–e**.

**Scheme 7:**
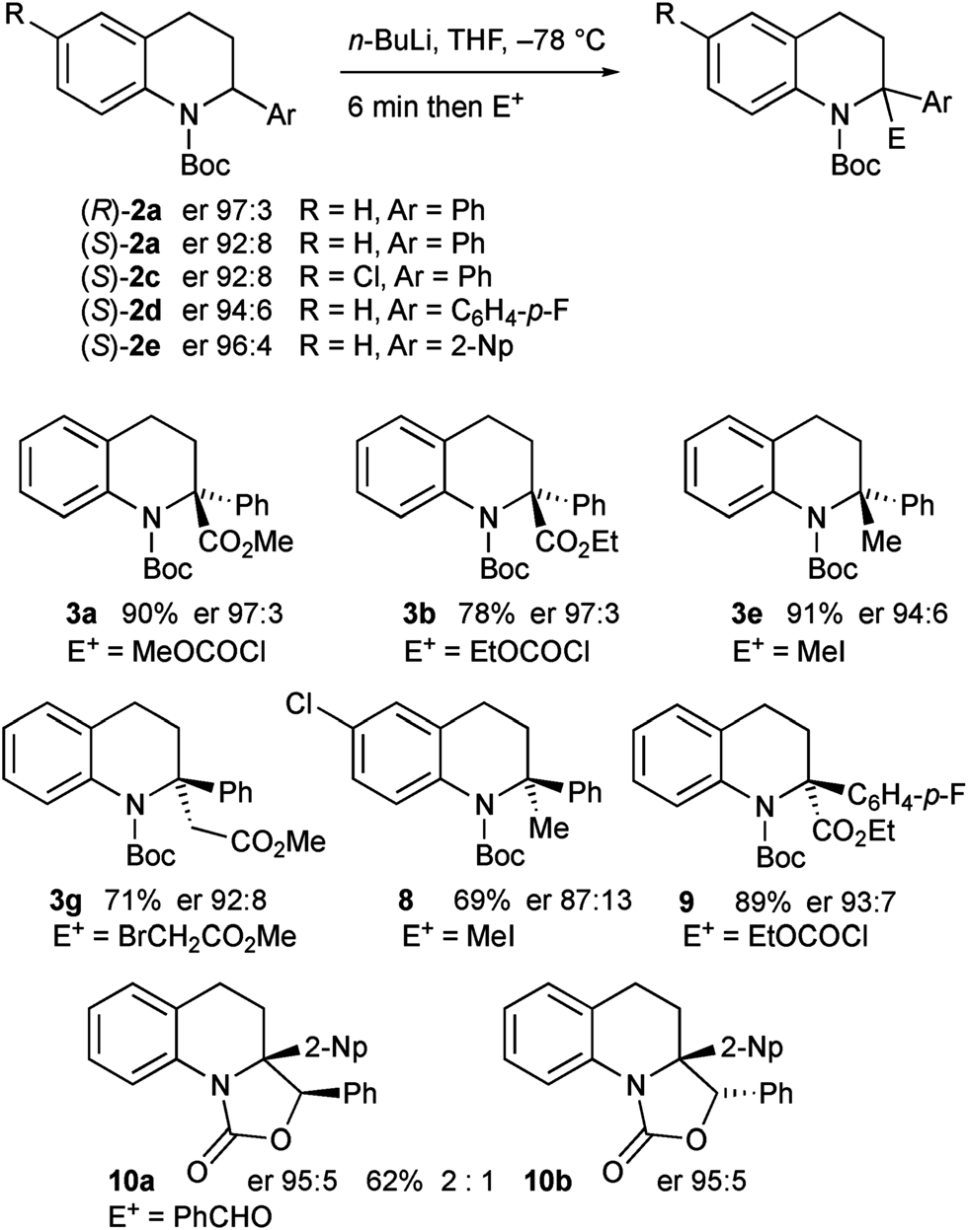
Lithiation–quench of enantioenriched tetrahydroquinolines **2a**, **2c–e**.

The enantioenriched 2-aryltetrahydroquinolines **2a** and **2c–e** were treated with *n*-BuLi in THF at –78 °C and the resulting organolithiums were found to be configurationally stable. After electrophilic quench, the products **3a–b**, **3e**, **3g**, and **8–10** were obtained with high enantiomer ratios (Scheme 7). A slight loss of enantioselectivity was noticeable on using iodomethane as the electrophile, possibly as this reacts more slowly allowing some racemization on warming prior to quench.16*b* The tetrahydroquinoline **3g** was recrystallised and its absolute configuration was confirmed by single crystal X-ray analysis. The major diastereomer of the oxazolidinones **10** was confirmed by X-ray analysis. For X-ray data, see ESI.†

**Scheme 8:**
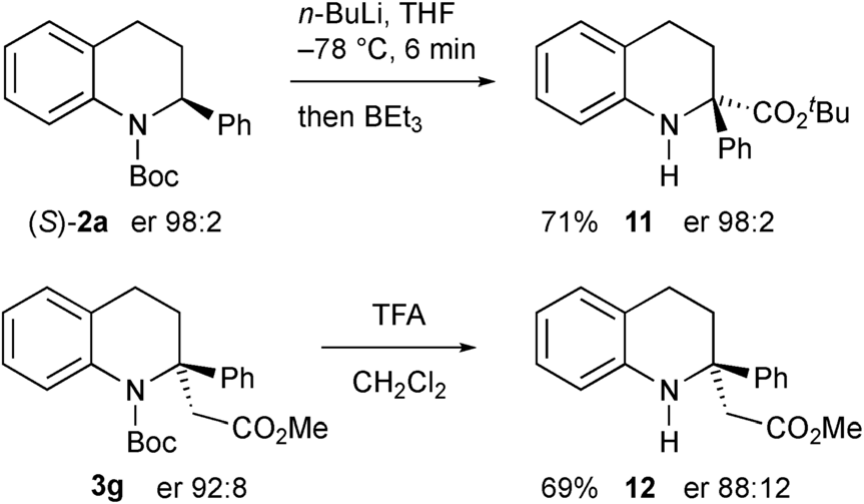
Formation of secondary amine products.

Another electrophile that we were interested in testing was a trialkylborane, as this could result in a borate intermediate that should be prone to rearrange.^17^ However we found that, instead of quenching the organolithium, addition of triethylborane promoted Boc group migration to give the tetrahydroquinoline **11** (Scheme 8).^18^ The absolute configuration of the product **11** was confirmed by single crystal X-ray analysis showing that the rearrangement occurred with retention of configuration starting with the highly enantioenriched tetrahydroquinoline **2a**. We speculate that the borane coordinates to the carbonyl oxygen atom to effect the migration and this must be preferable to direct reaction of the organolithium on the boron atom. The same reaction occurred with BEt_3_ as a catalyst (0.2 equiv. gave product **11**, 59% yield), or even in the absence of any catalyst (42% yield of **11** on using BuLi then warming without any BEt_3_). We were able to remove the Boc group from the nitrogen atom in the products by using trifluoroacetic acid (TFA), for example from compound **3g** to give the secondary amine **12** with only minimal loss of enantiopurity.

**Scheme 9:**
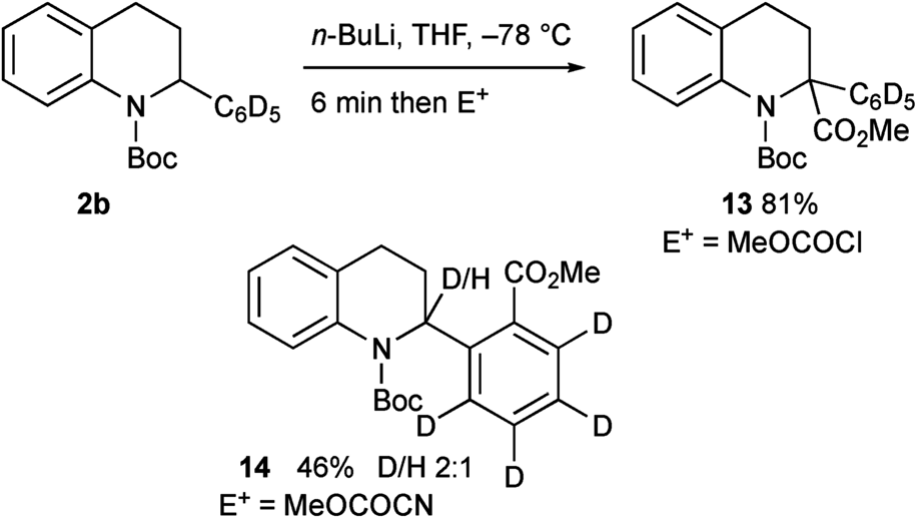
Lithiation–trapping of tetrahydroquinoline **2b**.

We were surprised to find that the electrophile methyl cyanoformate gave the product **4** rather than the expected product **3a**. We were concerned that there may have been competitive *ortho* lithiation,^19^ although this would be contrary to the formation of the products **3a–i**. We recently uncovered an example of such an unusual change in regioselectivity with a benzylic organolithium on changing the electrophile,11*c* and wanted to probe this reactivity further. Treatment of the deuterated tetrahydroquinoline **3c** under the same conditions (1.2 equiv. *n*-BuLi in THF) followed by addition of MeOCOCN returned recovered starting material **3c**, indicating a large kinetic isotope effect.^20^ This suggests that the benzylic proton in **2a** is indeed removed and not the *ortho* proton. Forcing the deprotonation with 3 equiv. *n*-BuLi for 1 h before addition of MeOCOCN still gave predominantly recovered **3c** but did give a small amount (8% yield) of the product **4** (no deuterium present). Treatment of the tetrahydroquinoline **2b** with *n*-BuLi then MeOCOCl gave the expected 2,2-disubstituted product **13** (Scheme 9). However, with MeOCOCN as the electrophile, the *ortho* substituted product **14** was obtained in moderate yield as a mixture in which the major product had deuterium at the 2-position. Hence the reaction must proceed by initial deprotonation alpha to the nitrogen atom. Most electrophiles react at C-2 to give the products **3a–i**. With methyl cyanoformate, substitution occurs at the ortho position and then rearomatisation takes place (see ESI†). The transfer of the proton (or deuterium) is likely to occur non-selectively and indeed on using enantioenriched tetrahydroquinoline **2a** (er 92 : 8, prepared as described in Scheme 5), the product **4** was formed with low selectivity (er 61 : 39).

**Fig. 2:**
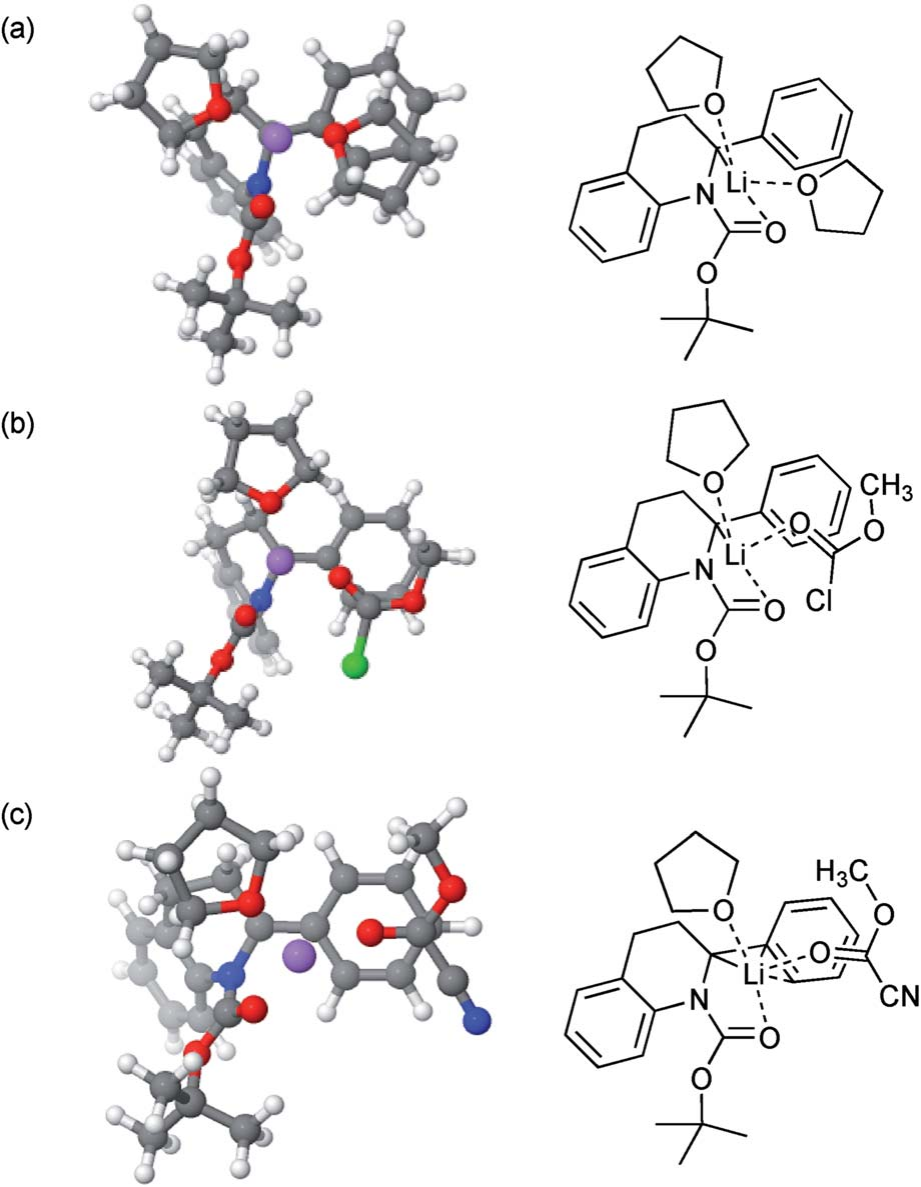
Lithiated intermediates by DFT [6-311G(d,p) basis set with B3LYP functional] and their ChemDraw representations. (a) In THF; (b) with MeOCOCl; (c) with MeOCOCN.

Calculations (using DFT/B3LYP-GD3BJ/6-311G**; see Computational methods below) initially focused on Boc rotation of tetrahydroquinoline **2a**, for which we found an activation Gibbs energy of 48.7 kJ mol^–1^ in fair agreement with the experimental value of about 44 kJ mol^–1^ at 298 K. Subsequent calculations focused on the structures of the intermediate organolithium. In particular, we studied the complexation of the intermediate lithiated species with MeOCOCl and MeOCOCN. This gave insight into the potential reason for the change in regioselectivity. The minimised structures had the lithium atom coordinated to the carbonyl oxygen atom and close to C-2 when coordinated to THF or MeOCOCl (Fig. 2a and b). On the other hand, there was clearly an η^3^ co-ordination of the lithium atom when MeOCOCN was bound (Fig. 2c). An alternative explanation could be that released cyanide could affect the regiochemistry, however an experiment in which MeOCOCl was added to the organolithium after addition of one equivalent of NaCN returned only the alpha-substituted product **3a**. Thus, we surmise that a change in structure of the organolithium on complexing the different electrophiles (MeOCOCl or MeOCOCN) must be influencing the regiochemistry on reaction with the electrophile, although the precise way that this happens will be subject to further study.^21^

## Experimental

A representative method for the kinetic resolution of the tetrahydroquinoline **2a** is given below. For further details and all data, see ESI.†

*n*-BuLi (0.6 mL, 0.39 mmol, 2.5 M in hexane) was added to freshly distilled (+)-sparteine (106 mg, 0.45 mmol) in PhMe (4 mL) at –78 °C. After 30 min, tetrahydroquinoline **2a** (101 mg, 0.33 mmol, 0.3 M solution in toluene) was added. After 2.5 h, methyl chloroformate (0.09 mL, 1.15 mmol) was added. The mixture was allowed to warm to room temperature over 16 h and then MeOH (1 mL) was added. Purification by column chromatography on silica gel, eluting with petrol–EtOAc (95 : 5), gave the recovered tetrahydroquinoline (*S*)-**2a** (44 mg, 43%) as an amorphous solid; m.p. 60–61 °C; er 98 : 2, determined by CSP HPLC with a Cellulose-2 column; [*α*]23D –103.9 (0.6, CHCl_3_); and in addition the tetrahydroquinoline (*R*)-**3a** (65 mg, 54%) as an amorphous solid; m.p. 71–74 °C; er 82 : 18, determined by CSP HPLC.

## Computational methods

All calculations were performed using density functional theory, employing the B3LYP^22^ functional as implemented in the D.01 version of Gaussian 09.^23^ Calculations included dispersion corrections using the GD3-BJ^24^ method. All calculations used the 6-311G(d,p)^25^ basis set. Solvent was included *via* the PCM method^26^ as implemented in Gaussian with the default parameters for THF.

The starting positions of coordinated solvent and electrophile molecules were varied to obtain the lowest energy structures. Frequency calculations were performed on all optimized structures to confirm that these were true minima. One transition state calculation was performed, for which a single imaginary frequency was found, as expected. For the calculations on **2a** no imaginary frequencies were found. The two complexes presented in Fig. 2a and b also showed no imaginary frequencies. The complex presented in Fig. 2c showed a single imaginary frequency of –16.5 cm^–1^. Inspection shows this mode is largely a torsional mode of the ligands around the lithium–ligand bond. Re-running the calculation with a finer integration grid and tighter optimization convergence led to a structure without imaginary frequencies. This latter structure is reported in the ESI.† All Gibbs energies reported were evaluated at 298.15 K and standard pressure. For the precise keywords used in each of the calculations see the ESI.†

## Conclusions

In conclusion, we have developed a rapid access to 2,2-disubstituted tetrahydroquinolines from 2-aryltetrahydroquinolines by deprotonation with *n*-butyllithium followed by electrophilic quench. The reaction proceeds with retention of configuration on using enantiomerically enriched starting materials. Surprisingly, methyl cyanoformate reacted at the ortho position of the 2-aryl substituent and this change in regioselectivity on change in the electrophile is proposed, on the basis of DFT studies, to result from a small change in the structure of the organolithium intermediate. Excellent enantioselectivities in the kinetic resolution were obtained in the presence of the chiral ligand sparteine. This chemistry therefore provides a new method to prepare highly enantiomerically enriched 2-aryltetrahydroquinolines and tetrahydroquinolines that are fully substituted at the 2 position.

## Conflicts of interest

There are no conflicts of interest to declare.

## Supplementary Material

Supplementary information

Crystal structure data
