# Supplementary material for: Synthesis and kinetic resolution of substituted tetrahydroquinolines by lithiation then electrophilic quench
Source: Chem Sci. 2017 Dec 14;9(5):1352–7. doi: 10.1039/c7sc04435f (PMC5887238; doi:10.1039/c7sc04435f)
Supplement: Supplementary file 1 [file SC-009-C7SC04435F-s001.pdf]

Electronic Supplementary Material (ESI) for Chemical Science.
This journal is © The Royal Society of Chemistry 2018

Electronic Supplementary Information for:

Synthesis and Kinetic Resolution of Substituted Tetrahydroquinolines by Lithiation then Electrophilic Quench

Nicholas Carter, Xiabing Li, Lewis Reavey, Anthony J. H. M. Meijer and Iain Coldham*

Department of Chemistry, University of Sheffield, Brook Hill, Sheffield, S3 7HF, UK

Contents

1.	Experimental Procedures and Characterisation Data	S-2
	1.1 Racemic lithiation–trapping	S-2
	1.2 Kinetic resolutions	S-28
	1.3 Lithiation-trapping of enantioenriched tetrahydroquinolines	S-37
	1.4 Removal of the Boc group	S-44
2.	ReactIR traces for lithiation of tetrahydroquinoline **2a**	S-47
3.	X-ray data for compound (*R*)-**3a**	S-50
4.	X-ray data for compound (*R*)-**3g**	S-60
5.	X-ray data for compound (±)-**10a**	S-71
6.	X-ray data for compound (*S*)-**11**	S-82
7.	Variable temperature NMR spectra for tetrahydroquinoline **2a**	S-92
8.	CSP-HPLC traces	S-95
9.	^{1}H and ^{13}C NMR spectra	S-110
10.	References	S-136
11.	DFT data	S-137

1. Experimental Procedures and Characterisation Data

1.1 Racemic lithiation–trapping

(RS)-tert-Butyl 2-Phenyl-1,2,3,4-tetrahydroquinoline-1-carboxylate 2a

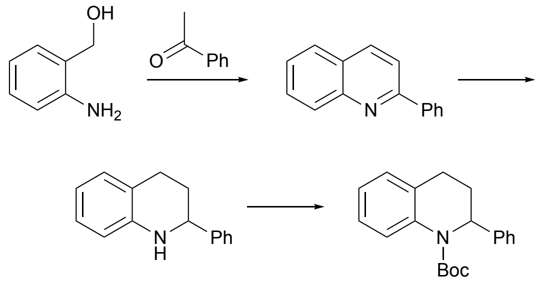

2a

Potassium *tert*-butoxide (4.56 g, 40.6 mmol) was added to 2-aminobenzyl alcohol (5.00 g, 40.6 mmol), benzophenone (7.40 g, 40.6 mmol), and acetophenone (4.74 mL, 40.6 mmol) in THF (20 mL) and the resulting mixture was stirred at 90 °C for 16 h. The mixture was allowed to cool to room temperature and was filtered through Celite. The resulting solution was added to a saturated solution of NH_4Cl (100 mL) and the aqueous layer was extracted with EtOAc (3 × 100 mL). The combined organic layers were dried ($MgSO_4$), filtered, and evaporated. The residue was purified by column chromatography on silica gel, eluting with petrol–EtOAc (95:5) and then EtOAc (10 mL) and aqueous HCl (50 mL, 2 M) were added. The organic layer was extracted with aqueous HCl (2 × 50 mL) and the combined aqueous layers were basified with aqueous NaOH (2 M) to pH 13 to obtain a suspension that was extracted with EtOAc (3 × 100 mL). The combined organic layers were dried ($MgSO_4$), filtered and evaporated to give 2-phenylquinoline (5.31 g, 64%) as an amorphous brown solid; m.p. 81–82 °C (lit.[1] 80–82 °C); ^{1}H NMR (400 MHz, $CDCl_3$) δ = 8.26 (1H, d, *J* = 8.5 Hz, CH), 8.22–8.17

(3H, m, 3 × CH), 7.91 (1H, d, J = 8.5 Hz, CH), 7.87–7.85 (1H, m, CH), 7.78–7.74 (1H, m, CH), 7.58–7.54 (3H, m, 3 × CH), 7.52–7.47 (1H, m, CH). Data consistent with the literature.[1,2]

Sodium cyanoborohydride (3.25 g, 51.7 mmol) was added to this quinoline (5.30 g, 25.8 mmol) and acetic acid (30 mL) at room temperature. After 16 h, the mixture was poured into saturated aqueous sodium carbonate and after allowing to stir for 15 min was extracted with CH_2Cl_2 (3 × 50 mL). The combined organic layers were dried ($MgSO_4$), filtered, and evaporated. The residue was purified by column chromatography on silica gel, eluting with petrol–EtOAc (97:3), to give 2-phenyl-1,2,3,4-tetrahydroquinoline (2.80 g, 52%) as an amorphous white solid; m.p. 37–38 °C (lit.[3] oil); ^{1}H NMR (400 MHz, $CDCl_3$) δ = 7.44–7.35 (4H, m, 4 × CH), 7.34–7.29 (1H, m, CH), 7.06 (2H, t, J = 7.0 Hz , 2 × CH), 6.68 (1H, t, J = 7.0 Hz, CH), 6.58 (1H, d, J = 8.0 Hz, CH), 4.47 (1H, dd, J = 9.0, 3.0 Hz, CH), 4.10 (1H, s, NH), 2.99–2.89 (1H, m, CH), 2.77 (1H, dt, J = 16.0, 5.0 Hz, CH), 2.19–2.12 (1H, m, CH), 2.07–1.98 (1H, m, CH). Data consistent with the literature.[3,4]

n-BuLi (4.80 mL, 11.5 mmol, 2.4 M in hexanes) was added to the 2-phenyl-1,2,3,4-tetrahydroquinoline (1.51 g, 7.2 mmol) in THF (30 mL) at –78 °C. After 1 h, Boc_2O (4.72 g, 21.6 mmol) in THF (30 mL) was added. The mixture was allowed to warm to room temperature over 16 h, was diluted with 10% sodium hydrogencarbonate solution and was extracted with Et_2O (2 × 50 mL). The combined organic layers were dried ($MgSO_4$), filtered, and evaporated. The residue was purified by column chromatography on silica gel, eluting with petrol–Et_2O (98:2), to give the carbamate **2a** (1.85 g, 83%) as an amorphous solid; m.p. 55–57 °C; R_f 0.27 [petrol–EtOAc (95:5)]; FT-IR ν_{max}(film)/cm^{-1} 2970, 1705, 1490; ^{1}H NMR (400 MHz, $CDCl_3$) δ = 7.83 (1H, d, J = 8.0 Hz, CH), 7.32–7.24 (3H, m, 3 × CH), 7.24–7.18 (3H, m, 3 × CH), 7.10 (1H, d,

J = 6.5 Hz, CH), 7.06–7.00 (1H, m, CH), 5.37 (1H, t, J = 8.0 Hz, CH), 2.74–2.59 (2H, m, 2 × CH), 2.50–2.41 (1H, m, CH), 1.91–1.80 (1H, m, CH), 1.37 (9H, s, *t*-Bu); ^{13}C NMR (100 MHz, $CDCl_3$) δ = 154.0 (C=O), 144.5 (C), 138.2 (C), 132.5 (C), 128.3 (CH), 127.5 (CH), 126.5 (CH), 126.3 (CH), 125.8 (CH), 124.7 (CH), 123.4 (CH), 80.9 (C), 58.7 (CH), 33.5 (CH_2), 28.2 (CH_3), 26.3 (CH_2); HRMS (ES) Found: MH^+, 310.1815. $C_{20}H_{24}NO_2$ requires MH^+, 310.1807; LRMS *m*/*z* (ES) 310 (20%, MH^+), 295 (100), 254 (90).

Resolution between the enantiomers of the carbamate **2a** was achieved using a Beckman system fitted with a Lux × 3u Cellulose–1 column (250 mm × 4.60 mm i.d.) as the stationary phase with a mixture of *n*-hexane–isopropanol (99:1 v/v) as the mobile phase at a flow rate of 1.0 $mL·min^{-1}$; ambient temperature, detection by UV absorbance at 254 nm. Injection volume 20 μL of the sample prepared in a 2 $g·L^{-1}$ solution of the eluent. Under these conditions, components were eluted at 6.8 min and 12.8 min. Alternatively, resolution between the enantiomers of the carbamate **2a** was achieved using a Beckman system fitted with a Lux Cellulose–2 column (250 mm × 4.60 mm i.d.) as the stationary phase with a mixture of *n*-hexane–isopropanol (99:1 v/v) as the mobile phase at a flow rate of 1.0 $mL·min^{-1}$; ambient temperature, detection by UV absorbance at 254 nm. Injection volume 20 μL of the sample prepared in a 2 $g·L^{-1}$ solution of the eluent. Under these conditions, the components were eluted at 5.3 min and 6.2 min.

(RS)-tert-Butyl 2-Phenyl-1,2,3,4-tetrahydroquinoline-1-carboxylate (phenyl-d5) 2b

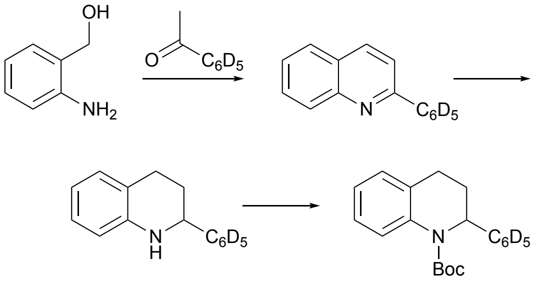

2b

Acetyl chloride (6.02 mL, 84.7 mmol) in anhydrous CS_2 (5 mL) was added dropwise to d_6-benzene (6.0 mL, 67.7 mmol) and $AlCl_3$ (11.3 g, 84.7 mmol) in anhydrous CS_2 (15 mL) at 0 °C. The mixture was allowed to warm to room temperature and was stirred for 5 h. The mixture was heated to 50 °C for 16 h. After cooling to room temperature, the mixture was poured into ice water (100 mL) and was extracted with CH_2Cl_2 (2 × 50 mL). The combined organic layers were washed with saturated Na_2CO_3 (20 mL) and brine (20 mL), dried (Na_2SO_4), filtered and the solvent was removed under reduced pressure. The mixture was purified using column chromatography on silica gel, eluting with petrol–EtOAc (95:5), to give acetophenone (phenyl-d_5) (7.91g, 93%) as an oil; HRMS (GC/MS) Found: M^+, 125.0883. $C_8H_3D_5O$ requires M^+, 125.0884; Data consistent with the literature.[5]

Potassium *tert*-butoxide (40.9 mL, 40.6 mmol, 1.0 M in THF) was added to 2-aminobenzyl alcohol (5.04 g, 40.9 mmol), benzophenone (7.46 g, 40.9 mmol), and acetophenone (phenyl-d_5) (4.77 mL, 40.9 mmol) in THF (20 mL) and the resulting mixture was stirred at 90 °C for 16 h. The mixture was allowed to cool to room temperature and was filtered through Celite. The resulting solution was added to a saturated solution of NH_4Cl (100 mL) and the aqueous layer was extracted with EtOAc (3 × 100 mL). The combined organic layers were dried ($MgSO_4$), filtered, and evaporated. The residue was purified by column chromatography on silica gel, eluting

with petrol–EtOAc (95:5) and then EtOAc (10 mL) and aqueous HCl (50 mL, 2 M) were added. The organic layer was extracted with aqueous HCl (2 × 50 mL) and the combined aqueous layers were basified with aqueous NaOH (2 M) to pH 13 to obtain a suspension that was extracted with EtOAc (3 × 100 mL). The combined organic layers were dried ($MgSO_4$), filtered and evaporated to give 2-phenylquinoline (phenyl-d_5) (7.4 g, 86%) as an amorphous yellow solid; m.p. 69–71 °C; R_f 0.58 [petrol–EtOAc (9:1)]; ^{1}H NMR (400 MHz, $CDCl_3$) δ = 8.25 (1H, d, *J* = 8.5 Hz, CH), 8.21 (1H, d, *J* = 8.5 Hz, CH), 7.88 (1H, d, *J* = 8.5 Hz, CH), 7.84 (1H, d, *J* = 8.5 Hz, CH), 7.81–7.73 (1H, m, CH), 7.59–7.52 (1H, m, CH); ^{13}C NMR (100 MHz, $CDCl_3$, deuterated carbons could not be observed) δ = 157.4 (C), 148.3 (C), 139.5 (C), 136.8 (CH), 129.8 (CH), 129.7 (CH), 127.5 (CH), 127.2 (C), 126.3 (CH), 119.1 (CH); HRMS (ES) Found MH^+, 211.1278. $C_{15}H_7D_5N$ requires MH^+, 211.1278; LRMS *m/z* (ES) 211 (100%, MH^+). No data reported.[6]

Sodium cyanoborohydride (4.33 g, 68.9 mmol) was added to this quinoline (7.23 g, 34.4 mmol) and acetic acid (100 mL) at room temperature. After 16 h, the mixture was poured into saturated aqueous sodium carbonate and after allowing to stir for 15 min was extracted with CH_2Cl_2 (3 × 70 mL). The combined organic layers were dried ($MgSO_4$), filtered, and evaporated. The residue was purified by column chromatography on silica gel, eluting with petrol–EtOAc (9:1), to give 2-phenyl-1,2,3,4-tetrahydroquinoline (phenyl-d_5) (4.67 g, 63%) as an oil; R_f 0.80 [petrol–EtOAc (9:1)]; FT-IR ν_{max} (ATR)/cm^{-1} 3390, 3050, 3015, 2920, 2840, 1605, 1585; ^{1}H NMR (400 MHz, $CDCl_3$) δ = 7.13–7.05 (2H, m, 2 × CH), 6.74 (1H, td, *J* = 7.5, 1.0 Hz, CH), 6.64–6.58 (1H, m, CH), 4.51 (1H, dd, *J* = 9.5, 3.0 Hz, CH), 4.10 (1H, s, NH), 3.06–2.94 (1H, m, CH), 2.81 (1H, dt, *J* = 16.5, 5.0 Hz, CH), 2.25–2.15 (1H, m, CH), 2.14–2.00 (1H, m, CH); ^{13}C NMR (100 MHz, $CDCl_3$, deuterated carbons could not all be

observed) δ = 144.8 (C), 144.7 (C), 129.4 (CH), 128.1 (t, *J* = 24.0 Hz, CD), 127.0 (CH), 126.2 (t, *J* = 24.0 Hz, CD), 120.9 (C), 117.2 (CH), 114.0 (CH), 56.2 (CH), 31.1 (CH_2), 26.4 (CH_2); HRMS (ES) Found MH^+, 215.1595. $C_{15}H_{11}D_5N$ requires MH^+, 215.1591; LRMS *m/z* (ES) 215 (100%, MH^+).

n-BuLi (1.57 mL, 3.77 mmol, 2.4 M in hexanes) was added to the 2-phenyl-1,2,3,4-tetrahydroquinoline (phenyl-d_5) (0.5 g, 2.35 mmol) in THF (10 mL) at –78 °C. After 1 h, Boc_2O (0.61 g, 2.82 mmol) in THF (10 mL) was added. The mixture was allowed to warm to room temperature over 16 h, was diluted with 10% sodium hydrogencarbonate solution and was extracted with Et_2O (2 × 30 mL). The combined organic layers were dried ($MgSO_4$), filtered, and evaporated. The residue was purified by column chromatography on silica gel, eluting with petrol–Et_2O (97:3), to give the carbamate **2b** (0.56 g, 76%) as an amorphous solid; m.p. 57–58 °C; R_f 0.59 [petrol–EtOAc (9:1)]; FT-IR ν_{max}(film)/cm^{-1} 3025, 3000, 2970, 2845, 1700, 1490; ^1H NMR (400 MHz, $CDCl_3$) δ = 7.86 (1H, d, *J* = 8.0 Hz, CH), 7.32–7.21 (1H, m, CH), 7.15–7.09 (1H, m, CH), 7.05 (1H, td, *J* = 7.5, 1.0 Hz, CH), 5.44–5.37 (1H, m, CH), 2.76–2.60 (2H, m, 2 × CH), 2.52–2.42 (1H, m, CH), 1.93–1.82 (1H, m, CH), 1.40 (9H, s, *t*-Bu); ^{13}C NMR (100 MHz, $CDCl_3$, deuterated carbons could not all be observed) δ = 154.1 (C=O), 144.4 (C), 138.2 (C), 132.5 (C), 127.8 (t, *J* = 24.0 Hz, CD), 127.5 (CH), 126.3 (CH), 125.4 (t, *J* = 24.0 Hz, CD), 124.7 (CH), 123.4 (CH), 80.9 (C), 58.6 (CH), 33.5 (CH_2), 28.2 (CH_3), 26.3 (CH_2); HRMS (ES) Found: MNa^+, 337.1935. $C_{20}H_{18}D_5NO_2Na$ requires MNa^+, 337.1935; LRMS *m/z* (ES) 337 (5%), 259 (100).

(RS)-tert-Butyl 6-Chloro-2-phenyl-1,2,3,4-tetrahydroquinoline-1-carboxylate 2c

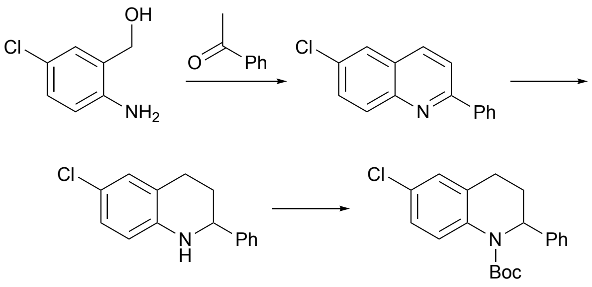

2c

Potassium *tert*-butoxide (32.4 mL, 32.4 mmol, 1.0 M in THF) was added to 2-amino-5-chlorobenzyl alcohol (5.12 g, 32.4 mmol), benzophenone (5.90 g, 32.4 mmol), and acetophenone (3.79 mL, 32.4 mmol) in THF (20 mL) and the resulting mixture was stirred at 90 °C for 16 h. The mixture was allowed to cool to room temperature and was filtered through Celite. The resulting solution was added to a saturated solution of NH_4Cl (100 mL) and the aqueous layer was extracted with EtOAc (3 × 100 mL). The combined organic layers were dried ($MgSO_4$), filtered, and evaporated. The residue was purified by column chromatography on silica gel, eluting with petrol–EtOAc (9:1) and then EtOAc (10 mL) and aqueous HCl (50 mL, 2 M) were added. The organic layer was extracted with aqueous HCl (2 × 50 mL) and the combined aqueous layers were basified with aqueous NaOH (2 M) to pH 13 to obtain a suspension that was extracted with EtOAc (3 × 100 mL). The combined organic layers were dried ($MgSO_4$), filtered and evaporated to give 6-chloro-2-phenylquinoline (6.20 g, 65%) as a solid; m.p. 102–104 °C (lit.[7] 110–111 °C); ^{1}H NMR (400 MHz, $CDCl_3$) δ = 8.18–8.12 (4H, m, 4 × CH), 7.91 (1H, d, *J* 8.5 Hz, CH), 7.82 (1H, d, *J* 2.5 Hz, CH), 7.68 (1H, dd, *J* 9.0, 2.5 Hz, CH), 7.58–7.48 (3H, m, 3 × CH). Data consistent with the literature.[7]

Sodium cyanoborohydride (3.16 g, 50.4 mmol) was added to this quinoline (6.02 g, 25.2 mmol) and acetic acid (150 mL) at room temperature. After 16 h, the mixture was

poured into saturated aqueous sodium carbonate and after allowing to stir for 15 min was extracted with CH_2Cl_2 (3 × 50 mL). The combined organic layers were dried ($MgSO_4$), filtered, and evaporated to give 6-chloro-2-phenyl-1,2,3,4-tetrahydroquinoline (4.90 g, 80%) as an oil; ^{1}H NMR (400 MHz, $CDCl_3$) δ = 7.40–7.30 (5H, m, 5 × CH), 7.00–6.97 (2H, m, 2 × CH), 6.49 (1H, d, *J* 8.0 Hz, CH), 4.45 (1H, dd, *J* 9.5, 3.5 Hz, CH), 4.09 (1H, s, NH), 2.95–2.86 (1H, m, CH), 2.73 (1H, dt, *J* 16.5, 5.0 Hz, CH), 2.17–2.10 (1H, m, CH), 2.04–1.94 (1H, m, CH). Data consistent with the literature.[8]

n-BuLi (1.37 mL, 3.3 mmol, 2.4 M in hexanes) was added to the 6-chloro-2-phenyl-1,2,3,4-tetrahydroquinoline (0.5 g, 2.05 mmol) in THF (15 mL) at –78 °C. After 20 min, 2-(Boc-oxyimino)-2-phenylacetonitrile (0.92 g, 3.74 mmol) was added. The mixture was allowed to warm to room temperature over 16 h, was diluted with 20% sodium hydrogencarbonate solution (10 mL) and was extracted with Et_2O (2 × 100 mL). The combined organic layers were dried ($MgSO_4$), filtered, and evaporated. The residue was purified by column chromatography on silica gel, eluting with petrol–Et_2O (97:3), to give the carbamate **2c** (0.44 g, 68%) as an amorphous solid; m.p. 77–80 °C; R_f 0.55 [petrol–EtOAc (9:1)]; FT-IR ν_{max}(film)/cm^{-1} 3025, 2955, 1705, 1490; ^{1}H NMR (400 MHz, $CDCl_3$) δ = 7.80 (1H, d, *J* = 9.0 Hz, CH), 7.33–7.26 (2H, m, 2 × CH), 7.25–7.15 (4H, m, 4 × CH), 7.09 (1H, d, *J* = 2.5 Hz, CH), 5.38 (1H, t, *J* = 7.0 Hz, CH), 2.70–2.57 (2H, m, 2 × CH), 2.46–2.38 (1H, m, CH), 1.92–1.83 (1H, m, CH), 1.36 (9H, s, *t*-Bu); ^{13}C NMR (100 MHz, $CDCl_3$) δ = 154.0 (C=O), 143.9 (C), 136.8 (C), 133.8 (C), 128.4 (CH), 128.3 (C), 127.4 (CH), 126.7 (CH), 126.3 (CH), 125.8 (CH), 125.7 (CH), 81.3 (C), 58.4 (CH), 32.8 (CH_2), 28.1 (CH_3), 26.0 (CH_2); HRMS (ES) Found MNa^+, 366.1233. $C_{20}H_{22}{}^{35}ClNaNO_2$ requires MNa^+, 366.1231; Found MNa^+,

368.1207. $C_{20}H_{22}{}^{37}ClNaNO_2$ requires MNa^+, 368.1209; LRMS *m/z* (ES) 368 (<5%), 366 (5) 290 (30), 288 (100).

Resolution between the enantiomers of the carbamate **2c** was achieved using a Beckman system fitted with a Lux Cellulose–2 column (250 mm × 4.60 mm i.d.) as the stationary phase with a mixture of *n*-hexane–isopropanol (99:1 v/v) as the mobile phase at a flow rate of 1.0 mL·min^{-1}; ambient temperature, detection by UV absorbance at 254 nm. Injection volume 20 µL of the sample prepared in a 2 g·L^{-1} solution of the eluent. Under these conditions, the components were eluted at 4.6 min and 5.6 min.

(RS)-tert-Butyl 2-(4-Fluorophenyl)-1,2,3,4-tetrahydroquinoline-1-carboxylate 2d

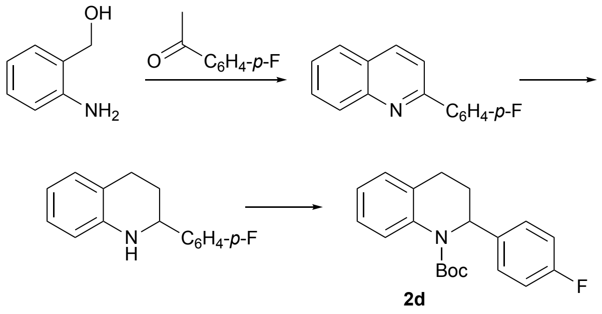

Potassium *tert*-butoxide (9.11 g, 81.2 mmol) was added to 2-aminobenzyl alcohol (5.00 g, 40.6 mmol), benzophenone (7.40 g, 40.6 mmol), and *p*-fluoro-acetophenone (4.93 mL, 40.6 mmol) in THF (20 mL) and the resulting mixture was stirred at 90 °C for 16 h. The mixture was allowed to cool to room temperature and was filtered through Celite. The resulting solution was added to a saturated solution of NH_4Cl (100 mL) and the aqueous layer was extracted with EtOAc (3 × 100 mL). The combined organic layers were dried ($MgSO_4$), filtered, and evaporated. The residue was purified by column chromatography on silica gel, eluting with petrol–EtOAc (95:5) and then EtOAc (10 mL) and aqueous HCl (50 mL, 2 M) were added. The organic layer was

extracted with aqueous HCl (2 × 50 mL) and the combined aqueous layers were basified with aqueous NaOH (2 M) to pH 13 to obtain a suspension that was extracted with EtOAc (3 × 100 mL). The combined organic layers were dried ($MgSO_4$), filtered and evaporated to give 2-(4-fluorophenyl)quinoline (3.64 g, 40%) as a solid; m.p. 84–85 °C (lit.[9] 92–93 °C); ^{1}H NMR (400 MHz, $CDCl_3$) δ = 8.31–8.16 (4H, m, 4 × CH), 7.87 (2H, d, *J* = 8.5 Hz, 2 × CH), 7.80–7.75 (1H, m, CH), 7.60–7.55 (1H, m, CH), 7.27–7.21 (2H, m, 2 × CH). Data consistent with the literature.[9]

Sodium cyanoborohydride (0.60 g, 9.6 mmol) was added to this quinoline (1.00 g, 4.48 mmol) and acetic acid (15 mL) at room temperature. After 16 h, the mixture was poured into saturated aqueous sodium carbonate and after allowing to stir for 15 min was extracted with CH_2Cl_2 (3 × 30 mL). The combined organic layers were dried ($MgSO_4$), filtered, and evaporated to give 2-(4-fluorophenyl)-1,2,3,4-tetrahydroquinoline (0.89 g, 88%) as an oil; ^{1}H NMR (400 MHz, $CDCl_3$) δ = 7.43–7.36 (2H, m, 2 × CH), 7.11–7.02 (4H, m, 4 × CH), 6.71 (1H, td, *J* = 7.5, 1.0 Hz, CH), 6.58 (1H, d, *J* = 7.5 Hz, CH), 4.46 (1H, dd, *J* = 9.5, 3.0 Hz, CH), 4.05 (1H, s, NH), 3.02–2.91 (1H, m, CH), 2.77 (1H, dt, *J* = 16.5, 4.5 Hz, CH), 2.17–2.09 (1H, m, CH), 2.05–1.94 (1H, m, CH). Data consistent with the literature.[3]

n-BuLi (2.64 mL, 6.34 mmol, 2.4 M in hexanes) was added to 2-(4-fluorophenyl)-1,2,3,4-tetrahydroquinoline (0.90 g, 3.96 mmol) in THF (15 mL) at –78 °C. After 1 h, Boc_2O (2.59 g, 11.9 mmol) in THF (45 mL) was added. The mixture was allowed to warm to room temperature over 16 h, was diluted with 10% sodium hydrogencarbonate solution and was extracted with Et_2O (3 × 50 mL). The combined organic layers were dried ($MgSO_4$), filtered, and evaporated. The residue was purified by column chromatography on silica gel, eluting with petrol–Et_2O (95:5), to give the carbamate **2d** (1.06 g, 82%) as an amorphous solid; m.p. 79–80 °C; R_f 0.36 [petrol–EtOAc

(95:5)]; FT-IR v_{max}(film)/cm^{-1} 3075, 3040, 3005, 2975, 2930, 1700, 1510, 1485; ^{1}H NMR (400 MHz, $CDCl_3$) δ = 7.89 (1H, d, *J* = 7.5 Hz, CH), 7.30 (1H, t, *J* = 7.5 Hz, CH), 7.27–7.19 (2H, m, 2 × CH), 7.17–7.11 (1H, m, CH), 7.10–7.04 (1H, m, CH), 7.04–6.93 (2H, m, 2 × CH), 5.42 (1H, t, *J* = 7.5 Hz, CH), 2.77–2.58 (2H, m, 2 × CH), 2.52–2.40 (1H, m, CH), 1.91–1.77 (1H, m, CH), 1.45 (9H, s, *t*-Bu); ^{13}C NMR (100 MHz, $CDCl_3$) δ = 161.6 (d, *J* = 244.0 Hz, C), 154.0 (C=O), 140.4 (d, *J* = 2.5 Hz, C), 138.0 (C), 132.4 (C), 127.6 (CH), 127.4 (d, *J* = 8.0 Hz, CH), 126.4 (CH), 124.8 (CH), 123.6 (CH), 115.2 (d, *J* = 21.5 Hz, CH), 81.0 (C), 58.1 (CH), 33.6 (CH_2), 28.2 (CH_3), 26.2 (CH_2); ^{19}F NMR (377 MHz, $CDCl_3$) δ = 116.6; HRMS (ES) Found: MH^+, 328.1713. $C_{20}H_{23}NO_2F$ requires MH^+, 328.1713; LRMS *m/z* (ES) 328 (5%), 313 (90), 272 (100); Found: C, 73.62; H, 6.99; N, 4.11. $C_{20}H_{22}FNO_2$ requires C, 73.37; H, 6.77; N, 4.28.

Resolution between the enantiomers of the carbamate **2d** was achieved using a Beckman system fitted with a Lux Cellulose–2 column (250 mm × 4.60 mm i.d.) as the stationary phase with a mixture of *n*-hexane–isopropanol (99:1 v/v) as the mobile phase at a flow rate of 1.0 mL·min^{-1}; ambient temperature, detection by UV absorbance at 254 nm. Injection volume 20 μL of the sample prepared in a 2 g·L^{-1} solution of the eluent. Under these conditions, the components were eluted at 4.9 min and 5.9 min.

***(RS)-tert*-Butyl 2-(Naphthalen-2-yl)-1,2,3,4-tetrahydroquinoline-1-carboxylate 2e**

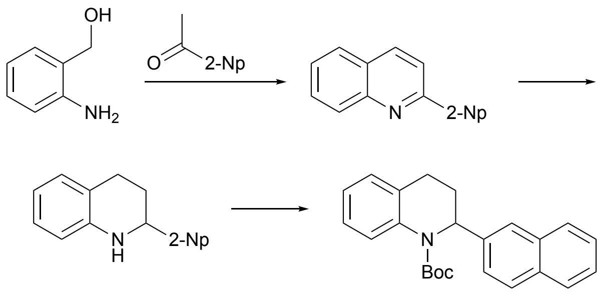

2e

Potassium *tert*-butoxide (41.0 mL, 41.0 mmol, 1.0 M in THF) was added to 2-aminobenzyl alcohol (5.05 g, 41.0 mmol), benzophenone (7.48 g, 41.0 mmol), and 2-acetylnaphthalene (6.98 g, 41.0 mmol) in THF (20 mL) and the resulting mixture was stirred at 90 °C for 16 h. The mixture was allowed to cool to room temperature and was filtered through Celite. The resulting solution was added to a saturated solution of NH_4Cl (100 mL) and the aqueous layer was extracted with EtOAc (3 × 100 mL). The combined organic layers were dried ($MgSO_4$), filtered, and evaporated. The residue was purified by column chromatography on silica gel, eluting with petrol–EtOAc (9:1) and then EtOAc (10 mL) and aqueous HCl (50 mL, 2 M) were added. The organic layer was extracted with aqueous HCl (2 × 50 mL) and the combined aqueous layers were basified with aqueous NaOH (2 M) to pH 13 to obtain a suspension that was extracted with EtOAc (3 × 100 mL). The combined organic layers were dried ($MgSO_4$), filtered and evaporated to give 2-(naphthalen-2-yl)quinoline (8.43 g, 80%) as a solid; m.p. 143–145 °C (lit.[10] 150 °C); ^{1}H NMR (400 MHz, $CDCl_3$) δ = 8.65 (1H, s, CH), 8.45–8.38 (1H, m, CH), 8.29 (1H, d, *J* = 8.5 Hz, CH), 8.25 (1H, d, *J* = 8.5 Hz, CH), 8.07–7.99 (3H, m, 3 × CH), 7.97–7.90 (1H, m, CH), 7.86 (1H, d, *J* = 8.0 Hz, CH), 7.79 (1H, t, *J* = 8.0 Hz, CH), 7.61–7.53 (3H, m, 3 × CH). Data consistent with the literature.[10]

Sodium cyanoborohydride (4.07 g, 64.8 mmol) was added to this quinoline (8.26 g, 32.4 mmol) and acetic acid (300 mL) at room temperature. After 16 h, the mixture was poured into saturated aqueous sodium carbonate and after allowing to stir for 15 min was extracted with CH_2Cl_2 (3 × 100 mL). The combined organic layers were dried ($MgSO_4$), filtered, and evaporated. The residue was purified by column chromatography on silica gel, eluting with petrol–EtOAc (9:1), to give 2-naphthyl-1,2,3,4-tetrahydroquinoline (5.48 g, 65%) as an amorphous solid; m.p. 79–81 °C (no lit. melting point reported); ^{1}H NMR (400 MHz, $CDCl_3$) δ = 7.93–7.85 (4H, m, 4 × CH), 7.60–7.50 (3H, m, 3 × CH), 7.14–7.06 (2H, m, 2 × CH), 6.75 (1H, td, *J* = 7.5, 1.0 Hz, CH), 6.66–6.62 (1H, m, CH), 4.65 (1H, dd, *J* = 9.5, 3.0 Hz, CH), 4.18 (1H, s, NH), 3.07–2.96 (1H, m, CH), 2.82 (1H, dt, *J* = 16.5, 5.0 Hz, CH), 2.29–2.20 (1H, m, CH), 2.20–2.08 (1H, m, CH). Data consistent with the literature.[4]

n-BuLi (2.73 mL, 6.27 mmol, 2.3 M in hexanes) was added to 2-naphthyl-1,2,3,4-tetrahydroquinoline (1.02 g, 3.92 mmol) in THF (20 mL) at –78 °C. After 20 min, Boc_2O (1.45 g, 6.66 mmol) in THF (10 mL) was added. The mixture was allowed to warm to room temperature over 16 h, was diluted with 10% sodium hydrogencarbonate solution and was extracted with Et_2O (3 × 50 mL). The combined organic layers were dried ($MgSO_4$), filtered, and evaporated. The residue was purified by column chromatography on silica gel, eluting with petrol–Et_2O (97:3), to give the carbamate **2e** (0.92 g, 65%) as an amorphous solid; m.p. 93–94 °C; R_f 0.49 [petrol–EtOAc (9:1)]; FT-IR ν_{max}(film)/cm^{-1} 3055, 3005, 2975, 2930, 2870, 2845, 1695, 1490; ^{1}H NMR (400 MHz, $CDCl_3$) δ = 7.90 (1H, d, *J* = 8.0 Hz, CH), 7.85–7.74 (3H, m, 3 × CH), 7.68 (1H, s, CH), 7.51–7.42 (2H, m, 2 × CH), 7.35 (1H, dd, *J* = 8.5, 2.0 Hz, CH), 7.33–7.28 (1H, m, CH), 7.13 (1H, m, CH), 7.13 (1H, dd, *J* = 7.5, 1.0 Hz, CH), 7.07 (1H, td, *J* = 7.5, 1.0 Hz, CH), 5.59–5.52 (1H, m, CH), 2.80–2.62 (2H, m, 2 × CH), 2.58–2.46 (1H, m,

CH), 2.03–1.90 (1H, m, CH), 1.36 (9H, s, *t*-Bu); ^{13}C NMR (100 MHz, $CDCl_3$) δ = 154.1 (C=O), 141.9 (C), 138.2 (C), 133.3 (C), 132.5 (C), 132.4 (C), 128.2 (CH), 127.8 (CH), 127.6 (CH), 127.5 (CH), 126.4 (CH), 126.0 (CH), 125.5 (CH), 124.8 (CH), 124.4 (CH), 124.3 (CH), 123.5 (CH), 81.0 (C), 58.8 (CH), 33.5 (CH_2), 28.2 (CH_3), 26.4 (CH_2); HRMS (ES) Found: MNa^+, 382.1785. $C_{24}H_{25}NO_2Na$ requires MNa^+, 382.1778; LRMS *m/z* (ES) 382 (25%, MNa^+), 304 (90, MH^+-*t*-Bu+H), 176 (100).

Resolution between the enantiomers of the carbamate **2e** was achieved using a Beckman system fitted with a Lux Cellulose–2 column (250 mm × 4.60 mm i.d.) as the stationary phase with a mixture of *n*-hexane–isopropanol (99:1 v/v) as the mobile phase at a flow rate of 1.0 mL·min^{-1}; ambient temperature, detection by UV absorbance at 254 nm. Injection volume 20 μL of the sample prepared in a 2 g·L^{-1} solution of the eluent. Under these conditions, the components were eluted at 5.8 min and 8.8 min.

(RS)-tert-Butyl 2-Methyl 2-Phenyl-1,2,3,4-tetrahydroquinoline-1,2-dicarboxylate 3a

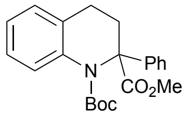

3a

n-BuLi (0.15 mL, 0.38 mmol, 2.5 M in hexanes) was added dropwise to the carbamate **2a** (100 mg, 0.32 mmol) in THF (1.3 mL) at –78 °C. After 6 min, methyl chloroformate (0.09 mL, 1.13 mmol) was added. The mixture was allowed to warm to room temperature over 16 h then MeOH (1 mL) was added. The solvent was evaporated and the residue was purified by column chromatography on silica gel, eluting with petrol–EtOAc (95:5), to give the carbamate **3a** (108 mg, 90%) as an amorphous solid; m.p. 104–106 °C; R_f 0.12 [petrol–EtOAc (95:5)]; FT-IR ν_{max}

(film)/cm^{-1} 3030, 2975, 2945, 2930, 1750, 1700, 1490; ^{1}H NMR (400 MHz, $CDCl_3$) δ = 7.90 (1H, d, *J* = 8.0 Hz, CH), 7.65–7.59 (2H, m, 2 × CH), 7.36–7.30 (2H, m, 2 × CH), 7.28–7.21 (2H, m, 2 × CH), 7.09–7.04 (1H, m, CH), 7.01 (1H, td, *J* = 7.5, 1.0 Hz, CH), 3.80 (3H, s, CH_3), 2.88–2.79 (1H, m, CH), 2.60–2.53 (1H, m, CH), 2.52–2.44 (1H, m, CH), 2.21–2.12 (1H, m, CH), 1.31 (9H, s, *t*-Bu); ^{13}C NMR (100 MHz, $CDCl_3$) δ = 173.5 (C=O), 153.8 (C=O), 142.3 (C), 137.7 (C), 131.7 (C), 127.7 (CH), 127.6 (CH), 126.8 (CH), 126.7 (CH), 126.5 (CH), 124.4 (CH), 123.4 (CH), 82.1 (C), 68.7 (C), 52.4 (CH_3), 39.4 (CH_2), 27.9 (CH_3), 25.2 (CH_2); HRMS (ES) Found: MH^+, 368.1865. $C_{22}H_{26}NO_4$ requires MH^+, 368.1862; LRMS *m*/*z* (ES) 368 (20%), 312 (20), 268 (100); Found: C, 72.02; H, 6.74; N, 3.66. $C_{22}H_{25}NO_4$ requires C, 71.91; H, 6.86; N, 3.81.

Resolution between the enantiomers of the carbamate **3a** was achieved using a Beckman system fitted with a Chiralpak IA column (250 mm × 4.60 mm i.d.) as the stationary phase with a mixture of *n*-hexane–isopropanol (99:1 v/v) as the mobile phase at a flow rate of 1.0 mL·min^{-1}; ambient temperature, detection by UV absorbance at 254 nm. Injection volume 20 μL of the sample prepared in a 2 g·L^{-1} solution of the eluent. Under these conditions, the components were eluted at 13.5 min and 21 min.

Alternatively, to test for the effect of cyanide on the regiochemistry: *n*-BuLi (0.16 mL, 0.38 mmol, 2.4 M in hexanes) was added to the carbamate **2a** (100 mg, 0.32 mmol) and NaCN (20 mg, 0.32 mmol) in THF (4 mL) at –78 °C. After 6 min, MeOCOCN (0.08 mL, 1 mmol) was added. The mixture was allowed to warm to room temperature over 16 h then MeOH (1 mL) was added. The solvent was evaporated and the residue was purified by column chromatography on silica gel, eluting with petrol–EtOAc (96:4), to give the carbamate **3a** (98 mg, 83%); data as above.

(RS)-tert-Butyl 2-Ethyl 2-Phenyl-1,2,3,4-tetrahydroquinoline-1,2-dicarboxylate 3b

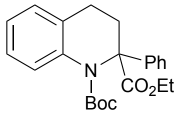

3b

n-BuLi (0.15 mL, 0.38 mmol, 2.5 M in hexanes) was added dropwise to the carbamate **2a** (100 mg, 0.32 mmol) in THF (1.3 mL) at –78 °C. After 6 min, ethyl chloroformate (0.09 mL, 1.13 mmol) was added. The mixture was allowed to warm to room temperature over 16 h then MeOH (1 mL) was added. The solvent was evaporated and the residue was purified by column chromatography on silica gel, eluting with petrol–EtOAc (95:5), to give the carbamate **3b** (87 mg, 71%) as an oil; R_f 0.3 [petrol–EtOAc (95:5)]; FI-IR ν_{max} (film)/cm^{-1} 2975, 2915, 2850, 1740, 1710; ^{1}H NMR (400 MHz, $CDCl_3$) δ = 7.88 (1H, d, *J* 8.0 Hz, CH), 7.63 (2H, d, *J* 7.5 Hz, 2 × CH), 7.35–7.00 (6H, m, 6 × CH), 4.34–4.20 (2H, m, CH_2), 2.87 (1H, ddd, *J* 14.5, 10.0, 3.0 Hz, CH), 2.61–2.46 (1H, m, CH), 2.53–2.46 (1H, m, CH), 2.20–2.14 (1H, m, CH), 1.33 (9H, s, *t*-Bu), 1.33 (3H, t, *J* 7.0 Hz, CH_3); ^{13}C NMR (100 MHz, $CDCl_3$) δ = 172.8 (C=O), 153.8 (C=O), 142.4 (C), 137.8 (C), 131.9 (C), 127.7 (2 × CH), 127.5 (CH), 126.8 (CH), 126.4 (CH), 124.4 (CH), 123.4 (CH), 82.0 (C), 68.6 (C), 61.5 (CH_2), 39.6 (CH_2), 27.9 (CH_3), 25.2 (CH_2), 14.2 (CH_3); HRMS (ES) Found: MH^+, 382.2001. $C_{23}H_{28}NO_4$ requires MH^+, 382.2018. LRMS *m*/*z* (ES) 382 (40%), 282 (100).

Resolution between the enantiomers of the carbamate **3b** was achieved using a Beckman system fitted with a Lux × 3u Cellulose–1 column (250 mm × 4.60 mm i.d.) as the stationary phase with a mixture of *n*-hexane–isopropanol (99.5:0.5 v/v) as the mobile phase at a flow rate of 1.0 mL·min^{-1}; ambient temperature, detection by UV absorbance at 254 nm. Injection volume 20 μL of the sample prepared in a 2 g·L^{-1}

solution of the eluent. Under these conditions, the components were eluted at 11.5 min and 12.5 min.

(*RS*)-*tert*-Butyl 2-Phenyl-1,2,3,4-tetrahydro(2-^{2}H)quinoline-1-carboxylate 3c

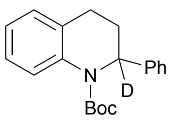

3c

n-BuLi (0.09 mL, 0.21 mmol, 2.4 M in hexanes) was added dropwise to the carbamate **2a** (53 mg, 0.17 mmol) in THF (1 mL) at –78 °C. After 6 min, deuterium oxide (0.3 mL) was added and the mixture was allowed to warm to room temperature over 16 h. The solvent was evaporated and the residue was purified by column chromatography on silica gel, eluting with petrol–EtOAc (95:5), to give the carbamate **3c** (41 mg, 76%, ~95%D) as an amorphous solid; m.p. 56–58 °C; R_f 0.33 [petrol–EtOAc (95:5)]; FT-IR ν_{max} (film)/cm^{-1} 3060, 3025, 2975, 2930, 1695, 1605; ^{1}H NMR (400 MHz, $CDCl_3$) δ = 7.85 (1H, d, *J* = 8.0 Hz, CH), 7.33–7.19 (6H, m, 6 × CH), 7.11 (1H, d, *J* = 6.5 Hz, CH), 7.05 (1H, td, *J* = 7.5, 1.0 Hz, CH), 5.38 (0.05H for compound **2a**, t, *J* = 8.0 Hz, CH), 2.75–2.59 (2H, m, 2 × CH), 2.50–2.41 (1H, m, CH), 1.91–1.81 (1H, m, CH), 1.38 (9H, s, *t*-Bu); ^{13}C NMR (100 MHz, $CDCl_3$) δ = 154.0 (C=O), 144.5 (C), 138.2 (C), 132.5 (C), 128.3 (CH), 127.5 (CH), 126.5 (CH), 126.3 (CH), 125.8 (CH), 124.7 (CH), 123.4 (CH), 80.9 (C), 58.3 (t, *J* = 23.5 Hz, CD), 33.4 (CH_2), 28.2 (CH_3), 26.3 (CH_2); HRMS (ES) Found: MNa^+, 333.1699. $C_{20}H_{22}DNO_2Na$ requires MNa^+, 333.1689; LRMS *m*/*z* (ES) 333 (100%), 277 (40), 233 (30).

***(RS)-tert*-Butyl 2-Phenyl-2-(tributylstannyl)-1,2,3,4-tetrahydroquinoline-1-carboxylate 3d**

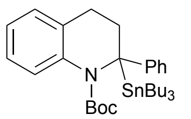

3d

n-BuLi (0.16 mL, 0.39 mmol, 2.4 M in hexanes) was added dropwise to the carbamate **2a** (101 mg, 0.33 mmol) in THF (1.3 mL) at –78 °C. After 6 min, tributyltin chloride (0.31 mL, 1.15 mmol) was added and the mixture was allowed to warm to room temperature over 16 h then MeOH (1 mL) was added. The solvent was evaporated and the residue was purified by column chromatography on silica gel, eluting with petrol–EtOAc (97:3), to give the carbamate **3d** (141 mg, 72%) as an amorphous solid; m.p. 52–54 °C; R_f 0.75 [petrol–EtOAc (95:5)]; FT-IR ν_{max} (film)/cm^{-1} 2955, 2920, 2870, 1670, 1490; ^{1}H NMR (400 MHz, $CDCl_3$) δ = 7.40–7.35 (1H, m, CH), 7.20–7.14 (2H, m, 2 × CH), 7.09–7.01 (3H, m, 3 × CH), 6.99–6.92 (2H, m, 2 × CH), 6.92–6.86 (1H, m, CH), 2.85–2.74 (1H, m, CH), 2.70–2.51 (3H, m, 3 × CH), 1.54 (9H, s, *t*-Bu), 1.47–1.33 (6H, m, 3 × CH_2), 1.33–1.20 (6H, m, 3 × CH_2), 0.87 (9H, t, J = 7.0 Hz, 3 × CH_3), 0.82–0.69 (6H, m, 3 × CH_2); ^{13}C NMR (100 MHz, $CDCl_3$) δ = 156.1 (C=O), 146.3 (C), 139.0 (C), 131.7 (C), 128.1 (CH), 127.9 (CH), 126.5 (CH), 124.9 (CH), 124.6 (CH), 123.8 (CH), 123.4 (CH), 81.2 (C), 60.5 (C), 33.4 (CH_2), 29.1 (CH_2), 28.4 (CH_3), 27.7 (CH_2), 25.2 (CH_2), 13.7 (CH_3), 13.4 (CH_2); HRMS (ES) Found: MNa^+, 622.2706. $C_{32}H_{49}NO_2Na^{120}Sn$ requires MNa^+, 622.2683; LRMS *m/z* (ES) 622 (100%), 486 (25), 412 (90).

***(RS)-tert*-Butyl 2-Methyl 2-Phenyl-1,2,3,4-tetrahydroquinoline-1-carboxylate 3e**

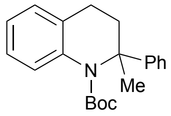

3e

n-BuLi (0.15 mL, 0.38 mmol, 2.5 M in hexanes) was added dropwise to the carbamate **2a** (100 mg, 0.32 mmol) in THF (1.3 mL) at –78 °C. After 6 min, iodomethane (0.1 mL, 1.13 mmol) was added and the mixture was allowed to warm to room temperature over 16 h then MeOH (1 mL) was added. The solvent was evaporated and the residue was purified by column chromatography on silica gel, eluting with petrol–EtOAc (95:5), to give the carbamate **3e** (91 mg, 87%) as an oil; R_f 0.5 [petrol–EtOAc (95:5)]; FT-IR ν_{max} (film)/cm^{-1} 3025, 2930, 2850, 2825, 1730; ^{1}H NMR (400 MHz, $CDCl_3$) δ = 7.73–6.98 (9H, m, 9 × CH), 2.79– 2.72 (1H, m, CH), 2.54– 2.48 (1H, m, CH), 2.05 (3H, s, CH_3), 1.99–1.93 (2H, m, 2 × CH), 1.21 (9H, s *t*-Bu); ^{13}C NMR (100 MHz, $CDCl_3$) δ = 154.5 (C=O), 149.3 (C), 139.1 (C), 132.9 (C), 128.1 (CH), 127.4 (CH), 126.0 (CH), 125.9 (CH), 124.8 (CH), 124.7 (CH), 122.7 (CH), 80.8 (C), 62.7 (C), 44.4 (CH_2), 27.9 (CH_3), 27.5 (CH_3), 24.9 (CH_2); HRMS (ES) Found: MH^+, 324.1949. $C_{21}H_{26}NO_2$ requires MH^+, 324.1964; LRMS *m/z* (ES) 324 (50%), 268 (100).

Resolution between the enantiomers of the carbamate **3e** was achieved using a Beckman system fitted with a Lux × 3u Cellulose–1 column (250 mm × 4.60 mm i.d.) as the stationary phase with a mixture of *n*-hexane–isopropanol (99:1 v/v) as the mobile phase at a flow rate of 1.0 mL·min^{-1}; ambient temperature, detection by UV absorbance at 254 nm. Injection volume 20 μL of the sample prepared in a 2 g·L^{-1} solution of the eluent. Under these conditions, the components were eluted at 5.6 min and 6.5 min.

(RS)-tert-Butyl 2-Allyl 2-Phenyl-1,2,3,4-tetrahydroquinoline-1-carboxylate 3f

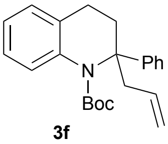

3f

n-BuLi (0.15 mL, 0.38 mmol, 2.5 M in hexanes) was added dropwise to the carbamate **2a** (100 mg, 0.32 mmol) in THF (1.3 mL) at –78 °C. After 6 min, allyl bromide (0.1 mL, 1.13 mmol) was added and the mixture was allowed to warm to room temperature over 16 h then MeOH (1 mL) was added. The solvent was evaporated and the residue was purified by column chromatography on silica gel, eluting with petrol−EtOAc (95:5), to give the carbamate **3f** (88 mg, 78%) as an amorphous solid; m.p. 58–60 °C; R_f 0.5 [petrol–EtOAc (95:5)]; FT-IR ν_{max} (film)/cm^{-1} 3055, 3030, 2980, 2920, 1690, 1640; ^{1}H NMR (400 MHz, $CDCl_3$) δ = 7.63−6.96 (9H, m, 9 × CH), 5.92−5.82 (1H, m, CH), 5.20 (1H, dd, *J* 17.0, 2.0 Hz, CH), 5.11 (1H, dd, *J* 10.0, 2.0 Hz, CH), 3.59 (1H, dd, *J* 13.0, 7.0 Hz, CH), 2.97 (1H, dd, *J* 13.0, 7.0 Hz, CH), 2.79 (1H, dt, *J* 15.0, 3.0 Hz, CH), 2.31 (1H, dt, *J* 15.0, 3.0 Hz, CH), 2.07 (1H, td, *J* 13.0, 3.0 Hz, CH), 1.90 (1H, dt, *J* 13.0, 3.0 Hz, CH), 1.19 (9H, s, *t*-Bu); ^{13}C NMR (100 MHz, $CDCl_3$) δ = 154.4 (C=O), 149.1 (C), 139.8 (C), 133.6 (CH), 132.7 (C), 128.1 (2 × CH), 127.3 (CH), 126.0 (CH), 124.9 (CH), 122.7 (CH), 118.6 (CH_2), 116.1 (CH), 80.9 (C), 64.7 (C), 44.2 (CH_2), 40.7 (CH_2), 27.9 (CH_3), 24.1 (CH_2); HRMS (ES) Found: MH^+, 350.2133. $C_{23}H_{28}NO_2$ requires MH^+, 350.2120; LRMS *m*/*z* (ES) 350 (100%), 335 (80).

(RS)-tert-Butyl 2-(2-Methoxy-2-oxoethyl)-2-phenyl-1,2,3,4-tetrahydroquinoline-1-carboxylate 3g

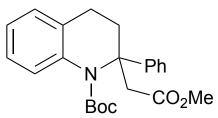

3g

n-BuLi (0.16 mL, 0.38 mmol, 2.4 M in hexanes) was added dropwise to the carbamate **2a** (97 mg, 0.31 mmol) in THF (1.3 mL) at –78 °C. After 6 min, methyl bromoacetate (0.1 mL, 1.1 mmol) was added and the mixture was allowed to warm to room temperature over 16 h then MeOH (1 mL) was added. The solvent was evaporated and the residue was purified by column chromatography on silica gel, eluting with petrol–EtOAc (97:3), to give the carbamate **3g** (76 mg, 63%) as an amorphous solid; m.p. 93–95 °C; R_f 0.37 [petrol–EtOAc (9:1)]; FT-IR ν_{max} (film)/cm^{-1} 3060, 3030, 3000, 2950, 2920, 1735, 1700, 1490; ^{1}H NMR (400 MHz, $CDCl_3$) δ = 7.61–7.56 (1H, m, CH), 7.40–7.25 (5H, m, 5 × CH), 7.23–7.16 (1H, m, CH) , 7.09–7.03 (1H, m, CH), 6.98 (1H, td, *J* = 7.5, 1.0 Hz, CH), 3.83 (1H, d, *J* = 12.5 Hz, CH), 3.56 (3H, s, OCH_3), 3.23 (1H, d, *J* = 12.5 Hz, CH), 2.83–2.72 (1H, m, CH), 2.62 (1H, td, *J* = 13.0, 3.0 Hz, CH), 2.31 (1H, dt, *J* = 15.0, 3.0 Hz, CH), 2.00 (1H, dt, *J* = 13.0, 3.0 Hz, CH), 1.21 (9H, s, *t*-Bu); ^{13}C NMR (100 MHz, $CDCl_3$) δ = 170.9 (C=O), 154.1 (C=O), 148.2 (C), 138.9 (C), 132.5 (C), 128.2 (CH), 127.3 (CH), 126.3 (CH), 125.9 (CH), 124.8 (CH), 124.5 (CH), 123.0 (CH), 81.2 (C), 63.7 (C), 51.6 (CH_3), 44.1 (CH_2), 40.7 (CH_2), 27.8 (CH_3), 24.0 (CH_2); HRMS (ES) Found: MH^+, 382.2008. $C_{23}H_{28}NO_4$ requires MH^+, 382.2013; LRMS *m/z* (ES) 382 (5%), 282 (100).

Resolution between the enantiomers of the tetrahydroquinoline **3g** was achieved using a Beckman system fitted with a Lux Cellulose–2 column (250 mm × 460 mm i.d.) as the stationary phase with a mixture of *n*-hexane–isopropanol (99.2:0.8 v/v) as the

mobile phase at a flow rate of 1 mL·min^{-1}; ambient temperature, detection by UV absorbance at 254 nm. Injection volume 20 μL of the sample prepared in a 2 g·L^{-1} solution of the eluent. Under these conditions, the components were eluted at 18 min and 20 min.

(*RS*)-*tert*-Butyl 2-Benzyl-2-phenyl-1,2,3,4-tetrahydroquinoline-1-carboxylate 3h

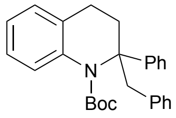

3h

n-BuLi (0.15 mL, 0.38 mmol, 2.5 M in hexanes) was added dropwise to the carbamate **2a** (100 mg, 0.32 mmol) in THF (1.3 mL) at –78 °C. After 6 min, benzyl bromide (0.1 mL, 1.13 mmol) was added and the mixture was allowed to warm to room temperature over 16 h then MeOH (1 mL) was added. The solvent was evaporated and the residue was purified by column chromatography on silica gel, eluting with petrol–EtOAc (95:5), to give the carbamate **3h** (98 mg, 76%) as plates; m.p. 138–140 °C; R_f 0.4 [petrol–EtOAc (95:5)]; FT-IR ν_{max} (film)/cm^{-1} 3059, 3030, 2930, 2960, 1690; ^{1}H NMR (400 MHz, $CDCl_3$) δ = 7.48 (2H, d, *J* 7.0 Hz, CH), 7.41 (2H, t, *J* 8.0 Hz, 2 × CH), 7.33–7.28 (3H, m, 3 × CH), 7.24–6.78 (7H, m, 7 × CH), 4.19 (1H, d, *J* 13.0 Hz, CH), 3.45 (1H, d, *J* 13.0 Hz, CH), 2.78 (1H, dt, *J* 13.0, 3.0 Hz, CH), 2.25 (1H, dt, *J* 13.0, 3.0 Hz, CH), 2.19 (1H, dt, *J* 13.0, 3.0 Hz, CH), 1.96 (1H, dt, *J* 13.0, 3.0 Hz, CH), 1.29 (9H, s, *t*-Bu); ^{13}C NMR (100 MHz, $CDCl_3$) δ = 154.6 (C=O), 149.3 (C), 138.9 (C), 137.0 (C), 132.7 (C), 131.2 (CH), 128.2 (CH), 127.8 (CH), 126.7 (CH), 126.5 (CH), 126.1 (CH), 125.4 (CH), 125.3 (CH), 125.1 (CH), 122.5 (CH), 81.1 (C), 65.7 (C), 43.6 (CH_2), 39.9 (CH_2), 28.0 (CH_3), 23.9 (CH_2); HRMS (ES) Found: MH^+, 400.2262. $C_{27}H_{30}NO_2$ requires MH^+, 400.2277; LRMS *m*/*z* (ES) 400 (100%).

***(RS)-tert*-Butyl 2-(4-Bromobenzyl)-2-phenyl-1,2,3,4-tetrahydroquinoline-1-carboxylate 3i**

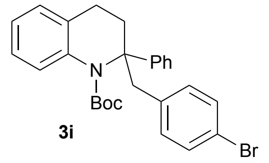

n-BuLi (0.15 mL, 0.37 mmol, 2.5 M in hexanes) was added dropwise to the carbamate **2a** (100 mg, 0.32 mmol) in THF (1.3 mL) at –78 °C. After 6 min, 4-bromobenzyl bromide (282 mg, 1.13 mmol) was added and the mixture was allowed to warm to room temperature over 16 h then MeOH (1 mL) was added. The solvent was evaporated and the residue was purified by column chromatography on silica gel, eluting with petrol–EtOAc (95:5), to give the carbamate **3i** (97 mg, 63%) as cubes; m.p. 139–141 °C; R_f 0.33 [petrol–EtOAc (95:5)]; FT-IR ν_{max} (film)/cm^{-1} 3035, 3010, 2980, 2930, 1700; ^{1}H NMR (400 MHz, $CDCl_3$) δ = 7.46–6.82 (13H, m, 13 × CH), 4.15 (1H, d, *J* 13.0 Hz, CH), 3.40 (1H, d, *J* 13.0 Hz, CH), 2.78 (1H, t, *J* 13.0 Hz, CH), 2.24–2.12 (2H, m, 2 × CH), 1.96 (1H, dt, *J* 13.0, 3.0 Hz, CH), 1.29 (9H, s, *t*-Bu); ^{13}C NMR (100 MHz, $CDCl_3$) δ = 154.1 (C=O), 148.9 (C), 138.8 (C), 136.1 (C), 132.8 (CH), 132.6 (C), 130.9 (CH), 128.3 (CH), 126.8 (CH), 126.2 (CH), 125.7 (CH), 125.1 (CH), 125.0 (CH), 122.7 (CH), 120.6 (C), 81.3 (C), 65.5 (C), 43.2 (CH_2), 39.9 (CH_2), 28.0 (CH_3), 23.9 (CH_2); HRMS (ES) Found: MH^+, 478.1364. $C_{27}H_{29}NO_2{}^{79}Br$ requires MH^+, 478.1382; LRMS *m*/*z* (ES) 480 (30%, MH^+ for ^{81}Br), 478 (30, MH^+ for ^{79}Br), 424 (100), 422 (100).

Resolution between the enantiomers of the carbamate **3i** was achieved using a Beckman system fitted with a Lux × 3u Cellulose–1 column (250 mm × 4.60 mm i.d.) as the stationary phase with a mixture of *n*-hexane–isopropanol (99:1 v/v) as the

mobile phase at a flow rate of 1.0 mL·min^{-1}; ambient temperature, detection by UV absorbance at 254 nm. Injection volume 20 μL of the sample prepared in a 2 g·L^{-1} solution of the eluent. Under these conditions, the components were eluted at 7.2 min and 7.9 min.

***(RS)-tert*-Butyl 2-[2-(Methoxycarbonyl)phenyl]-1,2,3,4-tetrahydroquinoline-1-carboxylate 4**

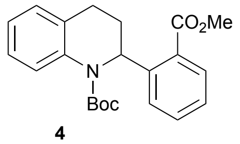

4

n-BuLi (0.16 mL, 0.39 mmol, 2.4 M in hexanes) was added dropwise to the carbamate **2a** (101 mg, 0.33 mmol) in THF (1.3 mL) at –78 °C. After 6 min, methyl cyanoformate (0.09 mL, 1.14 mmol) was added. After 1 h, MeOH (1 mL) was added and the mixture was allowed to warm to room temperature. The solvent was evaporated and the residue was purified by column chromatography on silica gel, eluting with petrol–EtOAc (95:5), to give the carbamate **4** (74 mg, 62%) as an amorphous solid; m.p. 73–75 °C; R$_f$ 0.46 [petrol–EtOAc (9:1)]; FT-IR ν_{max} (film)/cm^{-1} 3070, 3030, 3005, 2975, 2950, 2930, 1715, 1700, 1600, 1490; ^{1}H NMR (400 MHz, $CDCl_3$) δ = 7.95–7.88 (2H, m, 2 × CH), 7.47–7.41 (1H, m, CH), 7.37–7.24 (3H, m, 3 × CH), 7.17–7.12 (1H, m, CH), 7.06 (1H, td, *J* = 7.5, 1.0 Hz, CH), 6.09 (1H, dd, *J* = 10.5, 7.0 Hz, CH), 3.95 (3H, s, OCH_3), 2.83–2.60 (3H, m, 3 × CH), 1.63–1.51 (1H, m, CH), 1.25 (9H, s, *t*-Bu); ^{13}C NMR (100 MHz, $CDCl_3$) δ = 167.5 (C=O), 153.8 (C=O), 148.8 (C), 138.7 (C), 133.9 (C), 132.7 (CH), 130.2 (CH), 127.7 (C), 127.2 (CH), 126.5 (CH), 126.2 (CH), 125.9 (CH), 124.4 (CH), 123.4 (CH), 80.7 (C), 56.7 (CH), 52.0 (CH_3), 33.8 (CH_2), 27.9 (CH_3), 27.3

(CH_2); HRMS (ES) Found: MNa^+, 390.1681. $C_{22}H_{25}NO_4Na$ requires MNa^+, 390.1676; LRMS *m/z* (ES) 390 (15%), 268 (100).

From deuterium isotope studies (see manuscript), compound **4** is suggested to arise from lithiation of **2a** at C-2, followed by coordination of MeOCOCN to the organolithium to give an intermediate (see Fig 2c in the manuscript) that reacts at the ortho position. This would lead initially to a dearomatised intermediate but the C–H bond alpha to the ester is likely to break easily and rearomatisation would rapidly give product **4** according to the following scheme:

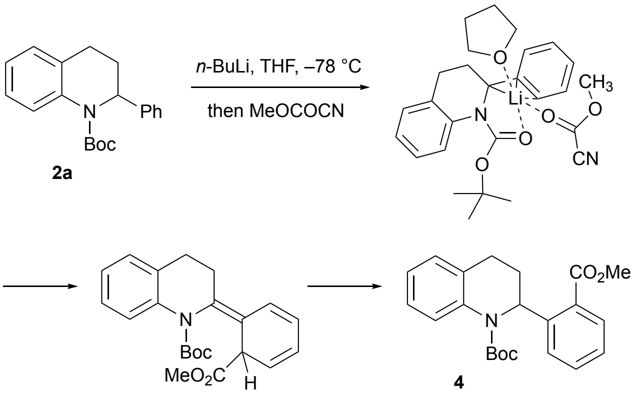

(RS)-tert-Butyl 2-Methyl 2-Phenyl-1,2,3,4-tetrahydroquinoline-1,2-dicarboxylate (phenyl-d5) 13

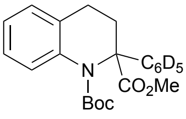

13

n-BuLi (0.17 mL, 0.39 mmol, 2.3 M in hexanes) was added dropwise to the carbamate **2b** (102 mg, 0.33 mmol) in THF (1.3 mL) at –78 °C. After 6 min, methyl chloroformate (0.09 mL, 1.13 mmol) was added. The mixture was allowed to warm to room temperature over 16 h then MeOH (1 mL) was added. The solvent was evaporated and the residue was purified by column chromatography on silica gel, eluting with petrol–EtOAc (95:5), to give the carbamate **13** (98 mg, 81%) as an

amorphous solid; m.p. 107–108 °C; R_f 0.38 [petrol–EtOAc (9:1)]; FT-IR ν_{max} (film)/cm^{-1} 3020, 3005, 2975, 2955, 2890, 2835, 1735, 1690, 1430; ^{1}H NMR (400 MHz, $CDCl_3$) δ = 7.93 (1H, d, *J* = 8.0 Hz, CH), 7.33–7.24 (1H, m, CH), 7.13–6.99 (2H, m, 2 × CH), 3.82 (3H, s, OCH_3), 2.93–2.77 (1H, m, CH), 2.66–2.44 (2H, m, 2 × CH), 2.26–2.12 (1H, m, CH), 1.33 (9H, s, *t*-Bu); ^{13}C NMR (100 MHz, $CDCl_3$, deuterated carbons could not be observed) δ = 173.5 (C=O), 153.8 (C=O), 142.2 (C), 137.8 (C), 131.6 (C), 127.6 (CH), 126.5 (CH), 124.3 (CH), 123.4 (CH), 82.1 (C), 68.7 (C), 52.4 (CH_3), 39.5 (CH_2), 27.9 (CH_3), 25.2 (CH_2); HRMS (ES) Found: MNa^+, 395.1993. $C_{22}H_{20}D_5NO_4Na$ requires MNa^+, 395.1990; LRMS *m/z* (ES) 395 (10%), 273 (100).

(*RS*)-*tert*-Butyl 2-[2-(Methoxycarbonyl)phenyl]-1,2,3,4-tetrahydroquinoline-1-carboxylate (phenyl-d4) and (*RS*)-*tert*-Butyl 2-[2-(Methoxycarbonyl)phenyl]-1,2,3,4-tetrahydro(2-^{2}H) quinoline-1-carboxylate (phenyl-d4) 14

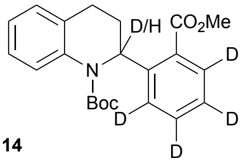

14

n-BuLi (0.17 mL, 0.39 mmol, 2.3 M in hexanes) was added dropwise to the carbamate **2b** (102 mg, 0.33 mmol) in THF (1.3 mL) at –78 °C. After 6 min, methyl cyanoformate (0.09 mL, 1.14 mmol) was added. The mixture was allowed to warm to room temperature over 16 h then MeOH (1 mL) was added. The solvent was evaporated and the residue was purified by column chromatography on silica gel, eluting with petrol–EtOAc (95:5), to give the carbamates **14** (58 mg, 46%) as an amorphous solid, ratio H:D 1:2; m.p. 69–71 °C; R_f 0.19 [petrol–EtOAc (95:5)]; FT-IR ν_{max} (film)/cm^{-1} 3025, 3005, 2970, 2950, 2845, 1710, 1695, 1490; ^{1}H NMR (400 MHz,

$CDCl_3$) δ = 7.92 (1H, d, *J* = 8.5 Hz, CH), 7.34–7.25 (1H, m, CH), 7.15 (1H, d, *J* = 7.5 Hz, CH), 7.10–7.04 (1H, m, CH), 6.10 (0.33H, dd, *J* = 10.0, 7.0 Hz, CH), 3.95 (3H, s, OCH_3), 2.84–2.59 (3H, m, 3 × CH), 1.64–1.51 (1H, m, CH), 1.25 (9H, s, *t*-Bu); ^{13}C NMR (100 MHz, $CDCl_3$, deuterated carbons could not be observed) δ = 167.5 (C=O), 153.9 (C=O), 148.8 and 148.7 (C), 138.7 (C), 133.9 (C), 127.6 (C), 127.2 (CH), 126.5 (CH), 124.4 (CH), 123.4 (CH), 80.7 (C), 56.6 (CH), 52.0 (CH_3), 33.7 and 33.6 (CH_2), 27.9 (CH_3), 27.2 (CH_2); HRMS (ES) Found: MNa^+, 394.1931. $C_{22}H_{21}D_4NO_4Na$ requires MNa^+, 394.1927 for *α*-H; Found: MNa^+, 395.1985. $C_{22}H_{20}D_5NO_4Na$ requires MNa^+, 395.1990 for *α*-D; LRMS *m*/*z* (ES) 395 (15%), 394 (20), 273 (80), 272 (100).

1.2 Kinetic resolutions

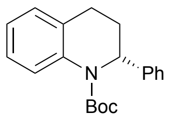

2a (er 97:3)

n-BuLi (1.6 mL, 3.9 mmol, 2.5 M in hexane) was added to freshly distilled (–)-sparteine (984 mg, 1.0 mmol) in PhMe (20 mL) at –78 °C. After 30 min, the tetrahydroquinoline **2a** (1.0 g, 3.2 mmol, 0.3 M solution in toluene) was added. After 2.5 h, methyl chloroformate (0.9 mL, 11 mmol) was added dropwise. The mixture was allowed to warm to room temperature over 16 h and then MeOH (5 mL) was added. Purification by flash column chromatography on silica gel, eluting with petrol−EtOAc (95:5), gave the recovered tetrahydroquinoline (*R*)-**2a** (427 mg, 43%) as plates; m.p. 60–61°C; other data as above; er 97:3, determined by CSP HPLC with the Lux × 3u Cellulose–1 column (major component eluted at 12.8 min); $[\alpha]_D^{23}$ +113 (0.7, $CHCl_3$).

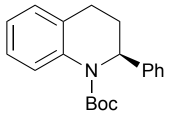

2a (er 92:8)

In the same way as above, *n*-BuLi (0.16 mL, 0.39 mmol, 2.45 M in hexanes), (+)-sparteine (100 mg, 0.43 mmol), PhMe (4 mL), tetrahydroquinoline **2a** (101 mg, 0.33 mmol) and methyl chloroformate (0.09 mL, 1.15 mmol) gave, after purification by column chromatography on silica gel, eluting with petrol–EtOAc (97:3), recovered tetrahydroquinoline (*S*)-**2a** (46 mg, 45%) as an amorphous solid; m.p. 51–54 °C; other data as above; er 92:8, determined by CSP-HPLC with the Cellulose-2 column (major component eluted at 6.6 min); $[\alpha]_D^{25}$ –85.5 (0.8, $CHCl_3$); in addition, the carbamate (*R*)-**3a** (51 mg, 42%) was isolated; m.p. 71–74 °C; other data as above; er 93:7, determined by CSP-HPLC (major component eluted at 22.5 min); $[\alpha]_D^{25}$ +76.6 (0.9, $CHCl_3$).

Alternatively, the *n*-BuLi can be added last: (+)-sparteine (106 mg, 0.45 mmol) and the tetrahydroquinoline **2a** (108 mg, 0.35 mmol) in toluene (8 mL) were cooled to –78 °C then *n*-BuLi (0.17 mL, 0.42 mmol, 2.5 M in hexanes) was added. After 2.5 h, ethyl chloroformate (0.12 mL, 1.22 mmol) was added to give, after purification by column chromatography on silica gel, eluting with petrol–EtOAc (97:3), the recovered tetrahydroquinoline (*S*)-**2a** (49 mg, 45%); data as above; er 90:10 determined by CSP-HPLC (major component eluted at 6.0 min); together with the carbamate (*R*)-**3b** (53 mg, 40%); data as above; er 90:10 by CSP-HPLC (major component eluted at 9.4 min).

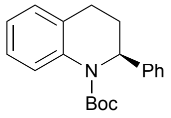

2a (er 98:2)

In the same way as above, *n*-BuLi (0.16 mL, 0.39 mmol, 2.5 M in hexanes), (+)-sparteine (106 mg, 0.45 mmol), PhMe (4 mL), tetrahydroquinoline **2a** (101 mg, 0.33

mmol) and methyl chloroformate (0.09 mL, 1.15 mmol) gave, after purification by column chromatography on silica gel, eluting with petrol–EtOAc (97:3), recovered tetrahydroquinoline (*S*)-**2a** (44 mg, 43%) as an amorphous solid; data as above; er 98:2, determined by CSP-HPLC with the Cellulose-2 column (major component eluted at 6.1 min); $[\alpha]_D^{23}$ –103.9 (0.6, $CHCl_3$); in addition, the carbamate (*R*)-**3a** (65 mg, 54%) was isolated; data as above; er 82:18 by CSP-HPLC (major component eluted at 23 min); $[\alpha]_D^{24}$ +67.5 (1.0, $CHCl_3$).

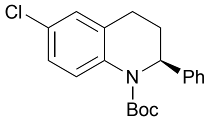

2c (er 92:8)

In the same way as above, *n*-BuLi (0.12 mL, 0.29 mmol, 2.4 M in hexanes), (+)-sparteine (119 mg, 0.51 mmol), PhMe (4 mL), tetrahydroquinoline **2c** (101 mg, 0.29 mmol) and methyl chloroformate (0.08 mL, 1.03 mmol) with a reaction time of 75 min gave, after purification by column chromatography on silica gel, eluting with petrol–EtOAc (97:3), recovered tetrahydroquinoline (*S*)-**2c** (40 mg, 41%) as a gum; data as above; er 92:8, determined by CSP-HPLC (major component eluted at 5.4 min); $[\alpha]_D^{23}$ –98.9 (0.1, $CHCl_3$); in addition, the carbamate **5** (54 mg, 42%) was isolated as an amorphous solid:

(*R*)-1-*tert*-Butyl 2-Methyl 6-Chloro-2-phenyl-1,2,3,4-tetrahydroquinoline-1,2-dicarboxylate 5

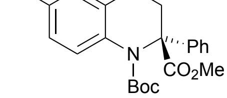

5

m.p. 41–43 °C; R_f 0.17 [petrol–EtOAc (95:5)]; FT-IR ν_{max} (film)/cm^{-1} 3065, 3025, 2975, 2970, 1745, 1705, 1485; ^{1}H NMR (400 MHz, $CDCl_3$) δ = 7.86 (1H, d, *J* = 9.0

Hz, CH), 7.62–7.56 (2H, m, 2 × CH), 7.37–7.30 (2H, m, 2 × CH), 7.28–7.19 (2H, m, 2 × CH), 7.06 (1H, d, J = 2.5 Hz, CH), 3.80 (3H, s, OCH_3), 2.78 (1H, ddd, J = 15.5, 8.5, 2.5 Hz, CH), 2.58–2.41 (2H, m, 2 × CH), 2.23–2.14 (1H, m, CH), 1.30 (9H, m, *t*-Bu); ^{13}C NMR (100 MHz, $CDCl_3$) δ = 173.1 (C=O), 153.6 (C=O), 141.9 (C), 136.4 (C), 133.2 (C), 128.4 (C), 127.8 (CH), 127.3 (CH), 127.0 (CH), 126.6 CH), 126.5 (CH), 125.6 (CH), 82.5 (C), 68.7 (C), 52.5 (OCH_3), 38.8 (CH_2), 27.8 (CH_3), 25.0 (CH_2); HRMS (ES) Found MH^+, 424.1305. $C_{24}H_{23}{}^{35}ClNO_4$ requires MH^+, 424.1310; Found MH^+, 426.1284. $C_{24}H_{23}{}^{37}ClNO_4$ requires MH^+, 426.1291; LRMS *m/z* (ES) 426 (5%), 424 (10), 304 (30), 302 (100); er 86:14, determined by CSP-HPLC (Lux Cellulose-2 column, major component eluted at 7.3 min), $[\alpha]_D^{25}$ +51.2 (1.2, $CHCl_3$).

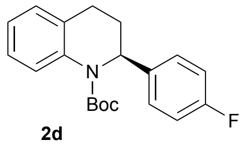

2d (er 94:6)

In the same way as above, *n*-BuLi (0.13 mL, 0.32 mmol, 2.5 M in hexanes), (+)-sparteine (133 mg, 0.57 mmol), PhMe (4 mL), tetrahydroquinoline **2d** (106 mg, 0.32 mmol) and methyl chloroformate (0.09 mL, 1.13 mmol) with a reaction time of 90 min gave, after purification by column chromatography on silica gel, eluting with petrol–EtOAc (97:3), recovered tetrahydroquinoline (*S*)-**2d** (39 mg, 36%) as an amorphous solid; data as above; er 94:6, determined by CSP-HPLC (major component eluted at 6.2 min); $[\alpha]_D^{24}$ –77.7 (0.1, $CHCl_3$); in addition, the carbamate **6** (53 mg, 42%) was isolated as an amorphous solid:

(*R*)-1-*tert*-Butyl 2-Methyl-2-(4-fluorophenyl)-1,2,3,4-tetrahydroquinoline-1,2-dicarboxylate 6

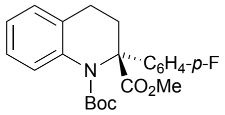

6

m.p. 42–44 °C; R$_f$ 0.25 [petrol–EtOAc (95:5)]; FT-IR ν_{max} (film)/cm^{-1} 3080, 3070, 3030, 3005, 2980, 1745, 1710; ^{1}H NMR (400 MHz, $CDCl_3$) δ = 7.88 (1H, d, *J* = 8.0 Hz, CH), 7.65–7.58 (2H, m, 2 × CH), 7.30–7.24 (1H, m, CH), 7.10–7.06 (1H, m, CH), 7.06–6.98 (3H, m, 3 × CH), 3.81 (3H, s, OCH_3), 2.89–2.80 (1H, m, CH), 2.60–2.43 (2H, m, 2 × CH), 2.17–2.08 (1H, m, CH), 1.34 (9H, s, *t*-Bu); ^{13}C NMR (100 MHz, $CDCl_3$) δ = 173.4 (C=O), 161.7 (d, *J* = 246.0 Hz, C), 153.7 (C=O), 138.1 (d, *J* = 3.0 Hz, C), 137.6 (C), 131.5 (C), 128.5 (d, *J* = 8.5 Hz, CH), 127.6 (CH), 126.5 (CH), 124.3 (CH), 123.5 (CH), 114.5 (d, *J* = 21.5 Hz, CH), 82.2 (C), 68.3 (C), 52.5 (CH_3), 39.5 (CH_2), 27.9 (CH_3), 25.1 (CH_2); ^{19}F NMR (377 MHz, $CDCl_3$) δ = 116.5; HRMS (ES) Found: MH$^+$, 386.1754. $C_{22}H_{25}NO_4F$ requires MH$^+$, 386.1768; LRMS *m/z* (ES) 386 (85%), 371 (20), 330 (100), 286 (75); er 85:15, determined by CSP-HPLC (ChiralPak IA, major component eluted at 18.0 min), $[\alpha]_D^{23}$ +59.8 (2.1, $CHCl_3$).

Alternatively, the enantioenriched tetrahydroquinoline **2d** was formed by kinetic resolution with trapping the organolithium by MeOCOCN:

n-BuLi (0.24 mL, 0.58 mmol, 2.4 M in hexane) was added to freshly distilled (+)-sparteine (230 mg, 0.98 mmol) in PhMe (5 mL) at –78 °C. After 30 min, the tetrahydroquinoline **2d** (189 mg, 0.58 mmol, 0.3 M solution in toluene) was added. After 2.5 h, methyl cyanoformate (0.16 mL, 2.0 mmol) was added. The mixture was allowed to warm to room temperature over 16 h and then MeOH (1 mL) was added. Purification by flash column chromatography on silica gel, eluting with petrol–EtOAc (97:3), gave the recovered tetrahydroquinoline (*S*)-**2d** (55 mg, 29%) as an amorphous

solid; data as above; er 98:2, determined by CSP-HPLC (major component eluted at 6.2 min); in addition, the carbamate **6** (51 mg, 23%) was isolated as an amorphous solid; data as above; er 81:19, determined by CSP-HPLC; in addition, the ortho substituted product *tert*-Butyl 2-[4-Fluoro-2-(methoxycarbonyl)phenyl]-1,2,3,4-tetrahydroquinoline-1-carboxylate shown below was isolated (63 mg, 28%) as an amorphous solid:

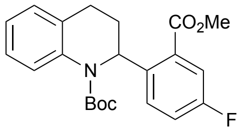

m.p. 64–66 °C; R_f 0.63 [petrol–EtOAc (9:1)]; FT-IR ν_{max} (ATR)/cm^{-1} 3020, 3005, 2980, 2955, 2930, 1725, 1700, 1585, 1490; ^{1}H NMR (400 MHz, $CDCl_3$) δ = 7.86 (1H, d, *J* = 8.0 Hz, CH), 7.62 (1H, dd, *J* = 9.5, 3.0 Hz, CH), 7.36–7.25 (2H, m, 2 × CH), 7.18–7.11 (2H, m, 2 × CH), 7.07 (1H, td, *J* = 7.5, 1.0 Hz, CH), 6.07 (1H, dd, *J* = 10.5, 7.0 Hz, CH), 3.95 (3H, s, OCH_3), 2.81–2.59 (3H, m, 3 × CH), 1.59–1.47 (1H, m, CH), 1.27 (9H, s, *t*-Bu); ^{13}C NMR (100 MHz, $CDCl_3$) δ = 166.3 (d, *J* = 2.5 Hz, C=O), 160.5 (d, *J* = 245.5 Hz, C), 153.8 (C=O), 144.8 (d, *J* = 4.0 Hz, C), 138.5 (C), 134.0 (C), 129.2 (d, *J* = 7.0 Hz, C), 127.9 (d, *J* = 7.5 Hz, CH), 127.2 (CH), 126.5 (CH), 124.5 (CH), 123.6 (CH), 119.8 (d, *J* = 21.5 Hz, CH), 116.7 (d, *J* = 24.0 Hz, CH), 80.8 (C), 56.2 (CH), 52.3 (CH_3), 33.9 (CH_2), 28.0 (CH_3), 27.3 (CH_2); ^{19}F NMR (377 MHz, $CDCl_3$) δ = 115.9; HRMS (ES) Found: MNa^+, 408.1586. $C_{22}H_{24}FNO_4Na$ requires MNa^+, 408.1582; er 56:44, determined by CSP-HPLC. Resolution between the enantiomers was achieved using a Beckman system fitted with a Daicel ChiralPak IA column (250 mm × 460 mm i.d.) as the stationary phase with a mixture of *n*-hexane:isopropanol (99:1 v/v) as the mobile phase at a flow rate of 1 mL·min^{-1}; ambient temperature, detection by UV absorbance at 254 nm. Injection volume was 20

μL of the sample prepared in a 2 g·L^{-1} solution of the eluent. Under these conditions, the components were eluted at 9.2 min and 9.8 min.

These results suggest that coordination of sparteine to the organolithium affects the regioselectivity in the electrophilic quench, since some of the product **6** was formed together with the ortho-substituted product on using methyl cyanoformate.

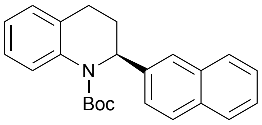

2e (er 96:4)

In the same way as above, *n*-BuLi (0.09 mL, 0.23 mmol, 2.5 M in hexanes), (+)-sparteine (90 mg, 0.37 mmol), PhMe (4 mL), tetrahydroquinoline **2e** (100 mg, 0.29 mmol) and methyl chloroformate (0.08 mL, 1.0 mmol) with a reaction time of 90 min gave, after purification by column chromatography on silica gel, eluting with petrol–EtOAc (97:3), recovered tetrahydroquinoline (*S*)-**2e** (40 mg, 39%) as a gum; data as above; er 96:4, determined by CSP-HPLC (major component eluted at 9.1 min); $[\alpha]_D^{20}$ –61.8 (0.1, $CHCl_3$); in addition, the carbamate **7** (20 mg, ~12%) was isolated as an amorphous solid:

1-*tert*-Butyl 2-Methyl 2-(Naphthalen-2-yl)-1,2,3,4-tetrahydroquinoline-1,2-dicarboxylate 7

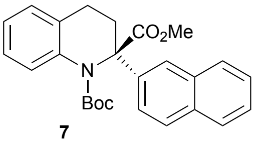

7

m.p. 47–49 °C; R_f 0.40 [petrol–EtOAc (9:1)]; FT-IR ν_{max} (film)/cm^{-1} 3065, 2975, 2925, 2850, 1745, 1710; ^{1}H NMR (400 MHz, $CDCl_3$) δ = 8.08 (1H, d, J = 1.5 Hz, CH),

7.99 (1H, d, J = 8.0 Hz, CH), 7.86–7.76 (4H, m, 4 × CH), 7.51–7.45 (2H, m, 2 × CH), 7.33–7.27 (1H, m, CH), 7.08 (1H, dd, J = 7.5, 1.5 Hz, CH), 7.03 (1H, td, J = 7.5, 1.5 Hz, CH), 3.84 (3H, s, OCH_3), 2.84 (1H, ddd, J = 14.5, 9.0, 3.0 Hz, CH), 2.63 (1H, ddd, J = 13.5, 8.0, 3.0 Hz, CH), 2.51 (1H, ddd, J = 14.5, 8.0, 3.0 Hz, CH), 2.36–2.26 (1H, m, CH), 1.29 (9H, s, *t*-Bu); ^{13}C NMR (100 MHz, $CDCl_3$) δ = 173.5 (C=O), 153.9 (C=O), 139.6 (C), 137.8 (C), 132.8 (C), 132.2 (C), 131.5 (C), 128.4 (CH), 127.6 (CH), 127.3 (CH), 127.3 (CH), 126.5 (CH), 125.9 (CH), 125.9 (CH), 125.4 (CH), 125.3 (CH), 124.4 (CH), 123.5 (CH), 82.2 (C), 68.9 (C), 52.5 (CH_3), 39.0 (CH_2), 27.9 (CH_3), 25.2 (CH_2); HRMS (ES) Found: MNa^+, 440.1835. $C_{26}H_{27}NO_4Na$ requires MNa^+, 440.1832; LRMS *m/z* (ES) 440 (15%), 318 (100); er 78:22, determined by CSP-HPLC (Lux Cellulose-2 column, major component eluted at 13.1 min); $[\alpha]_D^{24}$ +30.5 (0.6, $CHCl_3$).

In addition, a mixture (ratio 2.5:1 or 1:2.5) of the tetrahydroquinolines shown below were isolated (59 mg, 49%):

***tert*-Butyl 2-[1-(Methoxycarbonyl)naphthalen-2-yl]-1,2,3,4-tetrahydroquinoline-1-carboxylate and *tert*-Butyl 2-[3-(Methoxycarbonyl)naphthalen-2-yl]-1,2,3,4-tetrahydroquinoline-1-carboxylate**

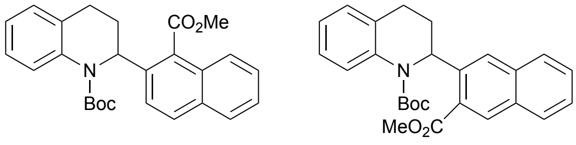

R_f 0.26 [petrol–EtOAc (9:1)]; FT-IR ν_{max} (ATR)/cm^{-1} 3025, 2970, 2920, 2845, 1700, 1490; ^{1}H NMR (400 MHz, $CDCl_3$) δ = 7.48–7.36 (1H, m, CH), 7.31–7.05 (7H, m, 7 × CH), 6.64–6.42 (2H, m, 2 × CH), 5.21 (0.3H, br s, CH), 4.83 (0.7H, s, CH), 3.60 (2.1H, s, CH_3), 3.29 (0.9H, s, CH_3), 3.12–2.70 (4H, m, 2 × CH_2), 1.52–1.36 (9H, m, *t*-Bu); ^{13}C NMR (100 MHz, $CDCl_3$, some carbons could not be observed) δ = 171.5

(C=O), 152.7 (C=O), 139.7 (C), 135.1 (C), 133.0 (C), 131.3 (C), 128.2 (CH), 128.1 (CH), 127.9 (CH), 127.7 (CH), 127.6 (CH), 127.3 (CH), 127.2 (CH), 126.2 (CH), 125.9 (CH), 125.1 (CH), 124.9 (CH), 81.6 (C), 81.2 (C), 52.6 (CH_3), 52.0 (CH_3), 47.5 (CH), 29.7 (2 × CH_2), 28.2 (CH_3), 28.1 (CH_3), 27.5 (CH_2), 27.3 (CH_2); HRMS (ES) Found: MNa^+, 440.1840 and 440.1846. $C_{26}H_{27}NO_4Na$ requires MNa^+, 440.1832.

This result indicates that, even with methyl chloroformate then the electrophile can sometimes prefer to react on the aromatic ring rather than at C-2 of the tetrahydroquinoline.

We have observed similar results with MeOCOCl with a lithiated azepane ring.[11] We believe that the results fit best with lithiation at C-2 but with reaction either at C-2 or at the aromatic ring (η^3 allyl/benzyl type reactivity). To probe this further, other experiments were conducted, whereby the organolithium generated from the naphthyl compound **2e** was trapped with methyl chloroformate or with iodomethane. This gave only the alpha-substituted product **7** (47% yield) or the alpha-methylated product shown below (73% yield), respectively.

These results support the formation of the 2-lithiated intermediate and that reaction occurs at C-2 with most electrophiles. However, with some electrophiles (particularly cyanoformates – see formation of **4**) or under some conditions (such as in the presence of sparteine – see above) then electrophilic quench can occur on the aromatic ring.

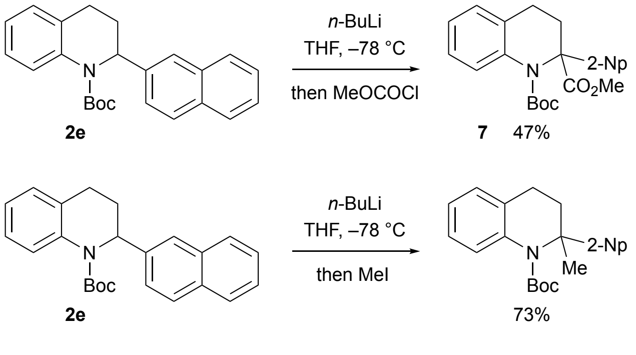

Data for compound **7** are identical to that described above. Data for the methylated compound are given below:

***tert*-Butyl 2-Methyl-2-(naphthalen-2-yl)-1,2,3,4-tetrahydroquinoline-1-carboxylate**

n-BuLi (0.13 mL, 0.31 mmol, 2.4 M in hexanes) was added to the carbamate **2e** (91 mg, 0.25 mmol) in THF (2 mL) at –78 °C. After 6 min, iodomethane (0.06 mL, 0.89 mmol) was added and the mixture was allowed to warm to room temperature over 16 h then MeOH (1 mL) was added. Purification by column chromatography on silica gel, eluting with petrol–EtOAc (95:5), gave the carbamate (70 mg, 73%) as an oil; R_f 0.61 [petrol–EtOAc (9:1)]; FT-IR ν_{max} (ATR)/cm^{-1} 3065, 3055, 2970, 2965, 1695, 1490; ^{1}H NMR (400 MHz, $CDCl_3$) δ = 7.90–7.80 (4H, m, 4 × CH), 7.77 (1H, d, *J* = 8.0 Hz, CH), 7.60–7.53 (1H, m, CH), 7.53–7.43 (2H, m, 2 × CH), 7.32–7.24 (1H, m, CH), 7.14 (1H, d, *J* = 7.5 Hz, CH), 7.08–7.00 (1H, m, CH), 2.86–2.72 (1H, m, CH), 2.54 (1H, dt, *J* = 15.0, 4.5 Hz, CH), 2.17 (3H, s, CH_3), 2.11–2.00 (2H, m, CH_2), 1.15 (9H, s, *t*-Bu); ^{13}C NMR (100 MHz, $CDCl_3$) δ = 154.5 (C=O), 146.8 (C), 139.1 (C), 133.3 (C), 133.0 (C), 131.9 (C), 128.0 (CH), 127.8 (CH), 127.4 (2 × CH), 126.1 (CH), 126.0 (CH), 125.4 (CH), 124.8 (CH), 123.7 (CH), 123.0 (CH), 122.8 (CH), 80.9 (C), 62.9 (C), 44.4 (CH_2), 27.9 (CH_3), 27.6 (CH_3), 25.1 (CH_2); HRMS (ES) Found: MNa^+, 396.1938. $C_{25}H_{27}NO_2Na$ requires MNa^+, 396.1934; LRMS *m/z* (ES) 396 (10%, MNa^+), 318 (100).

1.3 Lithiation-trapping of enantioenriched tetrahydroquinolines

General procedure:

n-BuLi (1.2 equiv., 2.5 M solution in hexanes) was added dropwise to a solution of enantioenriched **2a**, **2c** or **2d** (0.16 mmol) in THF (0.65 mL, 0.25 M) at –78 °C. After 6 min, the electrophile (0.56 mmol, 3.5 equiv.) was added dropwise. The mixture was allowed to warm to room temperature over 16 h then MeOH (1 mL) was added. The solvent was evaporated and the residue was purified by column chromatography on silica gel.

(*R*)-1-*tert*-Butyl 2-Methyl 2-Phenyl-3,4-dihydroquinoline-1,2(2*H*)-dicarboxylate 3a

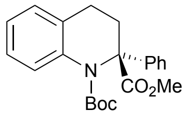

3a

Using the general procedure, tetrahydroquinoline (*R*)-**2a** (50 mg, 0.16 mmol, er 97:3), *n*-BuLi (76 µL, 0.19 mmol, 2.5 M in hexanes) and methyl chloroformate (45 µL, 0.6 mmol) gave, after purification by column chromatography on silica gel, eluting with petrol–EtOAc (95:5), the carbamate (*R*)-**3a** (53 mg, 90%) as an amorphous solid; m.p. 74–76 °C; er 97:3, determined by CSP HPLC (major component eluted at 17.9 min); $[\alpha]_D^{24}$ +94.1 (2.2, $CHCl_3$).

(*R*)-1-*tert*-Butyl 2-Ethyl 2-Phenyl-3,4-dihydroquinoline-1,2(2*H*)-dicarboxylate 3b

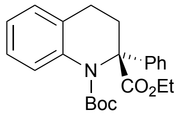

3b

Using the general procedure, tetrahydroquinoline (*R*)-**2a** (50 mg, 0.16 mmol, er 97:3), *n*-BuLi (76 μL, 0.19 mmol, 2.5 M in hexanes) and ethyl chloroformate (54 μL, 0.6 mmol) gave, after purification by column chromatography on silica gel, eluting with petrol−EtOAc (95:5), the carbamate (*R*)-**3b** (48 mg, 78%) as an oil; er 97:3, determined by CSP HPLC (major component eluted at 11.8 min); $[\alpha]_D^{23}$ +93.9 (0.83, $CHCl_3$).

(*R*)-*tert*-Butyl 2-Methyl-2-phenyl-3,4-dihydroquinoline-1(2*H*)-carboxylate 3e

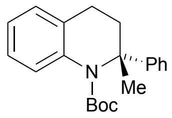

3e

Using the general procedure, tetrahydroquinoline (*R*)-**2a** (50 mg, 0.16 mmol, er 97:3), *n*-BuLi (76 μL, 0.19 mmol, 2.5 M in hexanes) and iodomethane (35 μL, 0.6 mmol) gave, after purification by column chromatography on silica gel, eluting with petrol−EtOAc (95:5), the carbamate **3e** (48 mg, 91%) as an oil; er 94:6, determined by CSP HPLC (major component eluted at 6.5 min); $[\alpha]_D^{24}$ +20.9 (2.2, $CHCl_3$).

(*R*)-*tert*-Butyl 2-(2-Methoxy-2-oxoethyl)-2-phenyl-1,2,3,4-tetrahydroquinoline-1-carboxylate 3g

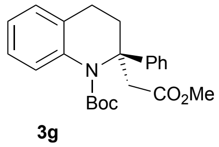

3g

Using the general procedure, tetrahydroquinoline (*S*)-**2a** (30 mg, 0.1 mmol, er 92:8), *n*-BuLi (48 μL, 0.11 mmol, 2.4 M in hexanes) and methyl bromoacetate (32 μL, 0.33 mmol) gave, after purification by column chromatography on silica gel, eluting with petrol–EtOAc (97:3), the carbamate **3g** (26 mg, 71%) as an amorphous solid; m.p. 96–98 °C; er 92:8, determined by CSP HPLC (major component eluted at 18.1 min); $[\alpha]_D^{25}$ +50.6 (1.7, $CHCl_3$).

(*S*)-*tert*-Butyl 6-Chloro-2-methyl-2-phenyl-1,2,3,4-tetrahydroquinoline-1-carboxylate 8

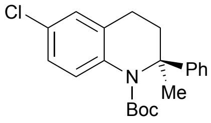

8

Using the general procedure, tetrahydroquinoline (*S*)-**2c** (34 mg, 0.10 mmol, er 92:8), *n*-BuLi (50 μL, 0.12 mmol, 2.4 M in hexanes) and iodomethane (21 μL, 0.34 mmol) gave, after purification by column chromatography on silica gel, eluting with petrol–EtOAc (97:3), the carbamate (*S*)-**8** (24 mg, 69%) as an oil; er 87:13, determined by CSP HPLC with a Daicel Chiralpak IA column using *n*-hexane–isopropanol (99:1 v/v) (major component eluted at 9.1 min); $[\alpha]_D^{25}$ –18.9 (1.1, $CHCl_3$); R_f 0.63 [petrol–EtOAc (94:6)]; FT-IR ν_{max} (film)/cm^{-1} 3065, 2975, 1700, 1600, 1480; ^1H NMR (400 MHz, $CDCl_3$) δ = 7.66 (1H, d, *J* = 9.0 Hz, CH), 7.38–7.31 (4H, m, 4 × CH), 7.27–7.21 (1H, m, CH), 7.18 (1H, dd, *J* = 9.0, 2.5 Hz, CH), 7.09 (1H, d, *J* = 2.5 Hz, CH), 2.78–2.64 (1H, m, CH), 2.53–2.41 (1H, m, CH), 2.02 (3H, s, CH_3), 1.99–1.92 (2H, m, 2 × CH), 1.18 (9H, s, *t*-Bu); ^{13}C NMR (100 MHz, $CDCl_3$) δ = 154.3 (C=O), 148.9 (C), 137.7

(C), 134.5 (C), 128.2 (CH), 127.6 (C), 127.1 (CH), 126.0 (2 × CH), 124.6 (2 × CH), 81.2 (C), 62.8 (C), 44.0 (CH_2), 27.9 (CH_3), 27.5 (CH_3), 24.8 (CH_2); HRMS (ES) Found MH^+, 380.1394. $C_{21}H_{24}{}^{35}ClNaNO_2$ requires MH^+, 380.1388; Found MH^+, 382.1369. $C_{21}H_{24}{}^{37}ClNaNO_2$ requires MH^+, 382.1366; LRMS *m/z* (ES) 382 (<5%), 380 (5), 304 (30), 302 (100).

(*S*)-*tert*-Butyl 2-Ethyl-2-(4-fluorophenyl)-1,2,3,4-tetrahydroquinoline-1,2-dicarboxylate 9

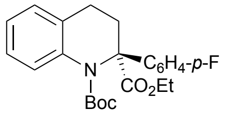

9

Using the general procedure, tetrahydroquinoline (*S*)-**2d** (25 mg, 0.08 mmol, er 94:6), *n*-BuLi (36 μL, 0.09 mmol, 2.5 M in hexanes) and ethyl chloroformate (25 μL, 0.26 mmol) gave, after purification by column chromatography on silica gel, eluting with petrol–EtOAc (97:3), the carbamate (*S*)-**9** (26 mg, 89%) as an amorphous solid; m.p. 34–36 °C; er 93:7, determined by CSP HPLC with a Lux Cellulose-1 column using *n*-hexane–isopropanol (99.5:0.5 v/v) (major component eluted at 11.0 min); $[\alpha]_D^{23}$ –75.3 (1.0, $CHCl_3$).

***cis*-3a-(Naphthalen-2-yl)-3-phenyl-3H,4H,5H-[1,3]oxazolo[3,4-a]quinolin-1-one 10a and *trans*-3a-(Naphthalen-2-yl)-3-phenyl-3H,4H,5H-[1,3]oxazolo[3,4-a]quinolin-1-one 10b**

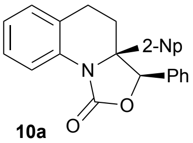

10a

and

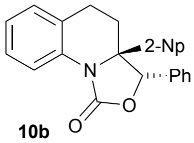

10b

Using the general procedure, tetrahydroquinoline (*S*)-**2e** (35 mg, 0.10 mmol, er 96:4), *n*-BuLi (50 µL, 0.12 mmol, 2.4 M in hexanes) and benzaldehyde (20 µL, 0.2 mmol), gave, after purification by column chromatography on silica gel, eluting with petrol–EtOAc (95:5), the carbamate (3*R*,3a*S*)-**10a** (16 mg, 42%) as an amorphous solid and the carbamate (3*S*,3a*S*)-**10b** (8 mg, 20%) as a gum:

Data for **10a**:

m.p. 172–174 °C; er 95.5:4.5, determined by CSP HPLC (major component eluted at 35 min); $[\alpha]_D^{24}$ = –228.9 (0.6, $CHCl_3$); R_f 0.15 [petrol–EtOAc (9:1)]; FT-IR ν_{max} (film)/cm^{-1} 3060, 2970, 2885, 2880, 1745, 1575, 1490, 1455; ^1H NMR (400 MHz, $CDCl_3$) δ = 8.80 (1H, d, *J* = 8.5 Hz, CH), 7.73–7.60 (2H, m, 2 × CH), 7.48–7.31 (5H, m, 5 × CH), 7.23–7.14 (1H, m, CH), 7.14–7.05 (2H, m, 2 × CH), 7.05–6.94 (4H, m, 4 × CH), 6.62 (1H, d, *J* = 7.0 Hz, CH), 5.61 (1H, s, CH), 3.06–2.93 (1H, m, CH), 2.91–2.74 (1H, m, CH), 2.52–2.34 (2H, m, 2 × CH); ^{13}C NMR (100 MHz, $CDCl_3$) δ = 153.4 (C=O), 134.9 (C), 133.4 (C), 133.4 (C), 132.7 (C), 132.3 (C), 129.2 (CH), 128.8 (CH), 128.3 (CH), 128.1 (2 × CH), 127.8 (CH), 127.5 (CH), 127.3 (CH), 126.7 (3 × CH), 126.4 (CH), 126.2 (CH), 124.5 (C), 123.6 (CH), 123.5 (CH), 118.0 (CH), 86.9 (CH), 68.8 (C), 30.8 (CH_2), 24.5 (CH_2); HRMS (ES) Found: MH^+, 392.1649. $C_{27}H_{22}NO_2$ requires MH^+, 392.1645; LRMS *m*/*z* (ES) 414 (10%), 392 (100).

Resolution between the enantiomers of the carbamate *cis*-**10a** was achieved using a Beckman system fitted with a Daicel ChiralPak IA column (250 mm × 460 mm i.d.) as the stationary phase with a mixture of *n*-hexane:isopropanol (95:5 v/v) as the mobile phase at a flow rate of 0.7 mL·min^{-1}; ambient temperature, detection by UV

absorbance at 254 nm. Injection volume was 20 μL of the sample prepared in a 2 g·L^{-1} solution of the eluent. Under these conditions, the components were eluted at 33 min and 50 min.

The stereochemistry of the major isomer was determined on the basis of an X-ray crystal structure of the racemic product, formed by using *n*-BuLi in THF followed by the addition of PhCHO. This gave the same two products, 64% yield, ratio 2:1 and the major isomer had m.p. 160–162 °C. The X-ray data for this compound have been submitted to CCDC 1578268.

Data for **10b**:

er 95.5:4.5, determined by CSP HPLC (major component eluted at 38.7 min); $[\alpha]_D^{24}$ = –17.2 (0.2, $CHCl_3$); R_f 0.14 [petrol–EtOAc (95:5)]; FT-IR ν_{max} (film)/cm^{-1} 3060, 3035, 2965, 2955, 2930, 2845, 1745, 1600, 1580, 1490; ^{1}H NMR (400 MHz, $CDCl_3$) 8.43 (1H, dd, *J* = 8.5, 1.0 Hz, CH), 7.93 (1H, d, *J* = 8.5 Hz, CH), 7.89–7.83 (1H, m, CH), 7.82–7.76 (1H, m, CH), 7.73 (1H, d, *J* = 1.5 Hz, CH), 7.56–7.44 (6H, m, 6 × CH), 7.42–7.30 (3H, m, 3 × CH), 7.02 (1H, td, *J* = 7.5, 1.0 Hz CH), 6.93 (1H, d, *J* = 7.5 Hz, CH), 5.59 (1H, s, CH), 2.72–2.62 (1H, m, CH), 2.47–2.34 (1H, m, CH), 2.12 (1H, ddd, *J* = 13.5, 5.5, 1.5 Hz, CH), 1.73 (1H, td, *J* = 13.5, 5.5 Hz, CH); ^{13}C NMR (100 MHz, $CDCl_3$) δ = 155.2 (C=O), 138.8 (C), 135.1 (C), 134.8 (C), 133.2 (C), 132.8 (C), 129.7 (CH), 129.1 (CH), 129.0 (CH), 128.7 (CH), 128.3 (CH), 127.6 (CH), 127.2 (CH), 126.7 (CH), 126.7 (CH), 126.5 (CH), 125.4 (C), 125.0 (CH), 124.1 (CH), 122.5 (CH), 120.4 (CH), 85.3 (CH), 67.6 (C), 27.8 (CH_2), 24.3 (CH_2); HRMS (ES) Found: MH^+, 392.1657. $C_{27}H_{22}NO_2$ requires MH^+, 392.1645; LRMS *m/z* (ES) 414 (5%), 392 (100).

Resolution between the enantiomers of the carbamate *trans*-**10b** was achieved using a Beckman system fitted with a Daicel ChiralPak IA (250 mm × 460 mm i.d.) as the

stationary phase with a mixture of *n*-hexane:isopropanol (95:5 v/v) as the mobile phase at a flow rate of 0.7 mL·min^{-1}; ambient temperature, detection by UV absorbance at 254 nm. Injection volume was 20 μL of the sample prepared in a 2 g·L^{-1} solution of the eluent. Under these conditions, the components were eluted at 38 min and 96 min.

1.4 Removal of the Boc group

(*S*)-*tert*-Butyl 2-Phenyl-1,2,3,4-tetrahydroquinoline-2-carboxylate 11

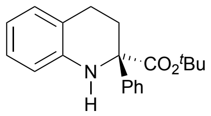

11

n-BuLi (43 μL, 0.10 mmol, 2.4 M in hexanes) was added to the tetrahydroquinoline (*S*)-**2a** (26 mg, 0.09 mmol, er 98:2) in THF (2 mL) at –78 °C. After 6 min, BEt_3 (0.171 mL, 0.171 mmol, 1.0 M in hexanes) was added. The mixture was allowed to warm to room temperature over 16 h and MeOH (1 mL) was added. The solvent was evaporated and the residue was purified by column chromatography on silica gel, eluting with petrol–EtOAc (97:3), to give the amine (*S*)-**11** (19 mg, 71%) as an amorphous white solid; m.p. 78–80 °C; er 98:2, determined by CSP HPLC (major component eluted at 11.8 min); $[\alpha]_D^{24}$ = –94.2 (0.4, $CHCl_3$); R_f 0.71 [petrol–EtOAc (9:1)]; FT-IR ν_{max} (film)/cm^{-1} 3400, 3055, 2980, 2930, 1720, 1480; ^{1}H NMR (400 MHz, $CDCl_3$) δ = 7.60–7.54 (2H, m, 2 × CH), 7.40–7.33 (2H, m, 2 × CH), 7.33–7.27 (1H, m, CH), 7.11–7.05 (1H, m, CH), 6.96 (1H, d, *J* = 7.5 Hz, CH), 6.77–6.72 (1H, m, CH), 6.68 (1H, td,

J = 7.5, 1.0 Hz, CH), 4.95 (1H, s, NH), 2.78–2.69 (1H, m, CH), 2.56–2.43 (2H, m, 2 × CH), 2.41–2.31 (1H, m, CH), 1.46 (9H, s, *t*-Bu); ^{13}C NMR (100 MHz, $CDCl_3$) δ = 172.5 (C=O), 143.0 (C), 142.7 (C), 129.0 (CH), 128.5 (CH), 127.5 (CH), 127.0 (CH), 125.7 (CH), 120.4 (C), 117.4 (CH), 114.3 (CH), 82.2 (C), 63.5 (C), 31.2 (CH_2), 27.9 (CH_3), 24.0 (CH_2); HRMS (ES) Found: MH^+, 310.1800. $C_{20}H_{24}NO_2$ requires MH^+, 310.1802; LRMS *m/z* (ES) 310 (100%), 254 (80); Found: C, 77.57; H, 7.65; N, 4.28. $C_{20}H_{23}NO_2$ requires C, 77.64; H, 7.49; N, 4.53.

Resolution between the enantiomers of the carbamate **11** was achieved using a Beckman system fitted with a Daicel ChiralPak IA column (250 mm × 4.60 mm i.d.) as the stationary phase with a mixture of *n*-hexane:isopropanol (99:1 v/v) as the mobile phase at a flow rate of 1 mL·min^{-1}; ambient temperature, detection by UV absorbance at 254 nm. Injection volume was 20 μL of the sample prepared in a 2 g·L^{-1} solution of the eluent. Under these conditions, the components were eluted at 10 min and 12 min.

(*R*)-Methyl 2-(2-Phenyl-1,2,3,4-tetrahydroquinolin-2-yl)acetate 12

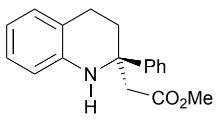

12

Trifluoroacetic acid (0.03 mL, 0.37 mmol) was added to the tetrahydroquinoline **3g** (14 mg, 0.037 mmol, er 92:8) in CH_2Cl_2 (5 mL) at room temperature. After 3 days, the solvent was evaporated and aqueous NaOH (2 mL, 1 M) was added. The mixture was extracted with CH_2Cl_2 (2 × 25 mL). The combined organic layers were dried ($MgSO_4$), filtered, and the solvent was evaporated. The residue was purified by column chromatography on silica gel, eluting with petrol–EtOAc (97:3), to give the amine (*R*)-**12** (7 mg, 69%) as an oil; er 88:12, determined by CSP HPLC (major component

eluted at 24.6 min); $[\alpha]_D^{20}$ = –92.8 (0.3, $CHCl_3$); R_f 0.55 [petrol–EtOAc (9:1)]; FT-IR ν_{max} (film)/cm^{-1} 3405 (N–H), 3050, 3020, 2955, 2910, 2840, 1715, 1605, 1480; ^{1}H NMR (400 MHz, $CDCl_3$) δ = 7.43–7.37 (2H, m, 2 × CH), 7.32 (2H, t, *J* = 7.0 Hz, 2 × CH), 7.27–7.20 (1H, m, CH) , 7.09 (1H, t, *J* = 7.5 Hz, CH), 6.92 (1H, d, *J* = 7.5 Hz, CH), 6.74 (1H, d, *J* = 8.0 Hz, CH), 6.69–6.61 (1H, m, CH), 5.64 (1H, s, NH), 3.53 (3H, s, OCH_3), 3.14 (1H, d, *J* = 15.5 Hz, CH), 2.90 (1H, d, *J* = 15.5 Hz, CH), 2.61 (1H, dt, *J* = 16.5, 4.5 Hz, CH), 2.41–2.29 (1H, m, CH), 2.28–2.17 (1H, m, CH), 2.06 (1H, td, *J* = 12.0, 4.5 Hz, CH); ^{13}C NMR (100 MHz, $CDCl_3$) δ = 171.9 (C=O), 145.0 (C), 143.5 (C), 129.1 (CH), 128.4 (CH), 127.2 (CH), 126.7 (CH), 125.7 (CH), 120.2 (C), 116.9 (CH), 114.0 (CH), 56.8 (C), 51.6 (CH_3), 46.3 (CH_2), 35.2 (CH_2), 23.7 (CH_2); HRMS (ES) Found: MH^+, 282.1489. $C_{18}H_{20}NO_2$ requires MH^+, 282.1489; LRMS *m/z* (ES) 282 (100).

Resolution between the enantiomers of the carbamate **12** was achieved using a Beckman system fitted with a Daicel ChiralCel OJ column (250 mm × 4.60 mm i.d.) as the stationary phase with a mixture of *n*-hexane:isopropanol (99:1 v/v) as the mobile phase at a flow rate of 1 mL·min^{-1}; ambient temperature, detection by UV absorbance at 254 nm. Injection volume was 20 μL of the sample prepared in a 2 g·L^{-1} solution of the eluent. Under these conditions, the components were eluted at 25 min and 36 min.

2. ReactIR traces for lithiation of tetrahydroquinoline **2a**

Racemic lithiation:

THF (12 mL) was added to a flask equipped with a stirrer bar and ReactIR probe at room temperature under Ar. After cooling to –78 °C, a solution of *N*-Boc-2-phenyltetrahydroquinoline **2a** (619 mg, 2.0 mmol) in THF (2 mL) was added dropwise. The solution was stirred for about 8 min (to verify stability of readout on ReactIR). Then, *n*-BuLi (1.0 mL, 2.5 mmol, 2.5 M in hexane) was added dropwise. The solution was stirred for 30 min.

For *N*-Boc-2-phenyltetrahydroquinoline **2a**, a peak at 1705 cm^{-1} was observed which was assigned to $\nu_{C=O}$ (blue line in 2D plots below). After addition of *n*-BuLi, a new peak at 1641 cm^{-1} appeared which was assigned to $\nu_{C=O}$ of lithiated intermediate (red line in 2D plots below). Complete lithiation of *N*-Boc-2-phenyltetrahydroquinoline **2a** to lithiated intermediate was observed within a few min.

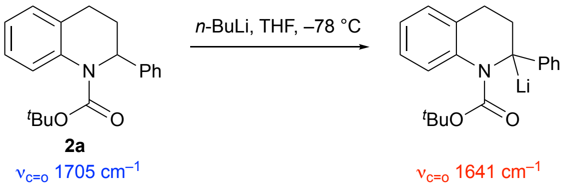

2D plot (time in minutes):

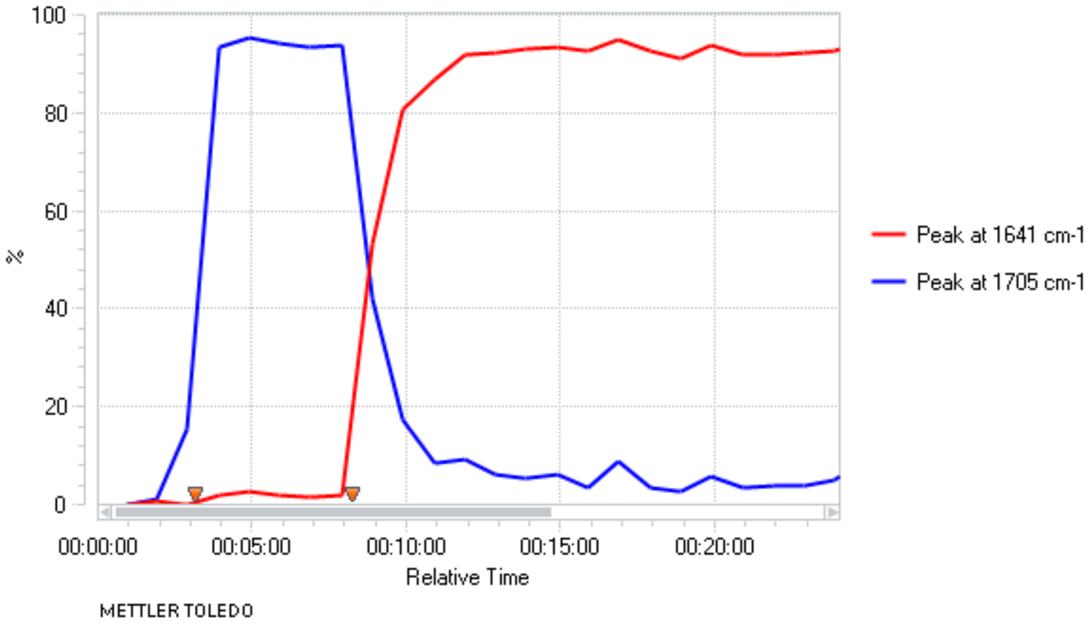

3D plot (time in h:min:sec):

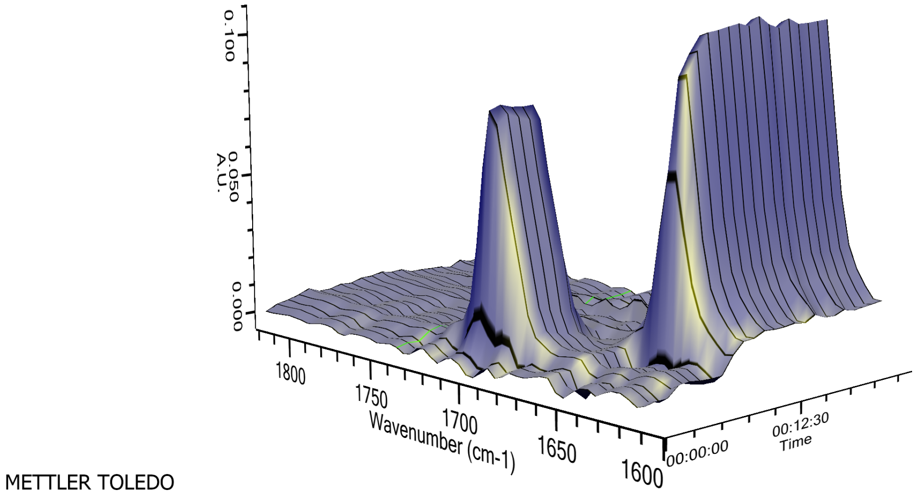

Kinetic resolution:

An experiment was run in which 0.7 equiv. of *n*-BuLi/(–)-sparteine was added to the tetrahydroquinoline **2a** in PhMe at –78 °C and the reaction was monitored:

Toluene (12 mL) was added to a flask equipped with a stirrer bar and ReactIR probe at room temperature under Ar. After cooling to –78 °C, a solution of *N*-Boc-2-phenyltetrahydroquinoline **2a** (619 mg, 2.0 mmol) in THF (2 mL) was added dropwise. The solution was stirred for 10 min (to verify stability of readout on ReactIR). Then, a solution of pre-mixed *n*-BuLi/(–)-sparteine (0.58 mL, 1.4 mmol, 2.5 M in hexane for *n*-BuLi; 0.33 g, 1.4 mmol for chiral ligand) in toluene (2 mL) was added dropwise.

The ReactIR plot is shown below (time in h:min:sec):

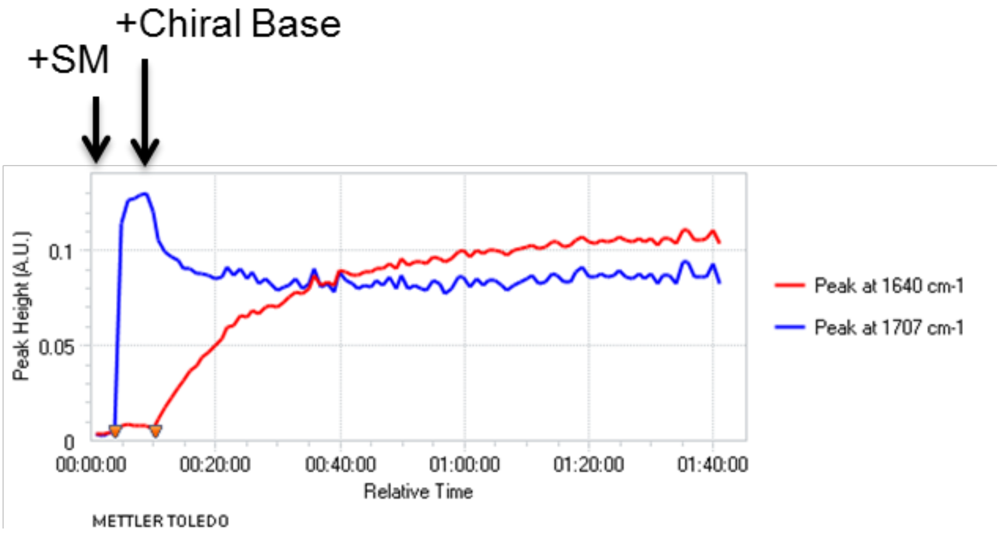

The result shows initial rapid but partial loss of the peak at 1707 cm^{-1} followed by only slight further slow loss over time and this was complemented by the formation of a peak at 1640 cm^{-1} (representing the carbonyl of the organolithium intermediate) that was formed rapidly at first and then much more slowly.

On the basis of these *in situ* ReactIR spectroscopic studies, we conducted the lithiation of *rac*-**2a** by using 0.7 equiv. of *n*-BuLi/(–)-sparteine at –78 °C in toluene for 1 h followed by quenching with MeOCOCl. However, only 26% yield of 2,2-disubstituted product **3a** (er 92:8) was produced, together with recovered starting material **2a** [er 18:82 (*S*:*R*)] in 67% yield. These results indicate that the kinetic resolution is best performed with more than 0.7 equiv. of the chiral base.

3. X-ray data for compound (*R*)-**3a**

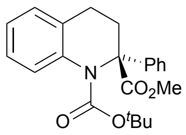

3a

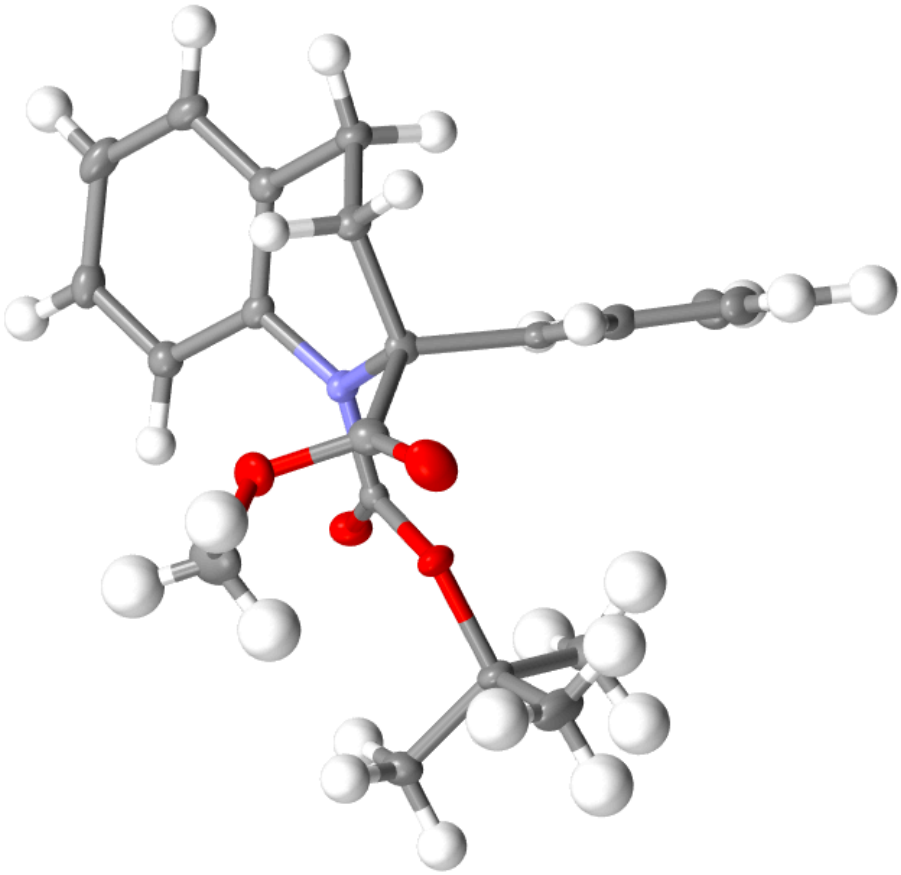

Table S-1. Crystal data and structure refinement for **3a**.

Identification code	carter1_0m	
Empirical formula	C22 H25 N O4	
Formula weight	367.43	
Temperature	100(2) K	
Wavelength	1.54178 ≈	
Crystal system	Orthorhombic	
Space group	$P2_12_12_1$	
Unit cell dimensions	a = 10.3192(3) ≈	α= 90∞.
	b = 11.0867(4) ≈	β= 90∞.
	c = 17.4057(6) ≈	γ = 90∞.
Volume	1991.31(12) $≈^3$	
Z	4	
Density (calculated)	1.226 Mg/m^3	
Absorption coefficient	0.680 mm^{-1}	
F(000)	784	
Crystal size	0.300 x 0.200 x 0.200 mm^3	
Theta range for data collection	4.729 to 66.627∞.	
Index ranges	-12<=h<=12, -13<=k<=13, -20<=l<=20	
Reflections collected	25795	
Independent reflections	3512 [R(int) = 0.0414]	
Completeness to theta = 66.627∞	99.7 %	
Absorption correction	Semi-empirical from equivalents	
Max. and min. transmission	0.89 and 0.79	
Refinement method	Full-matrix least-squares on F^2	
Data / restraints / parameters	3512 / 0 / 248	
Goodness-of-fit on F^2	1.095	
Final R indices [I>2sigma(I)]	R1 = 0.0280, wR2 = 0.0682	
R indices (all data)	R1 = 0.0296, wR2 = 0.0691	
Absolute structure parameter	0.03(6)	
Extinction coefficient	n/a	
Largest diff. peak and hole	0.138 and -0.193 e.$≈^{-3}$	

CCDC 1578266

Table S-2. Atomic coordinates (x 10^4) and equivalent isotropic displacement parameters (≈^{2}x 10^3) for **3a**. U(eq) is defined as one third of the trace of the orthogonalized U^{ij} tensor.

	x	y	z	U(eq)
O(1)	7820(1)	73(1)	8262(1)	16(1)
O(2)	9182(1)	1679(1)	8198(1)	19(1)
O(3)	5014(1)	-639(1)	8774(1)	31(1)
O(4)	5252(1)	1153(1)	8199(1)	24(1)
N(1)	7366(1)	1689(1)	8968(1)	14(1)
C(1)	8795(2)	-247(2)	6995(1)	21(1)
C(2)	8645(2)	-743(2)	7798(1)	19(1)
C(3)	8216(2)	1187(2)	8444(1)	14(1)
C(4)	6424(2)	886(2)	9360(1)	15(1)
C(5)	7126(2)	-79(2)	9841(1)	15(1)
C(6)	6458(2)	-1064(2)	10141(1)	20(1)
C(7)	7082(2)	-1904(2)	10603(1)	26(1)
C(8)	8383(2)	-1769(2)	10784(1)	27(1)
C(9)	7838(2)	-1890(2)	7789(1)	35(1)
C(10)	9927(2)	-945(2)	8202(1)	34(1)
C(11)	5569(2)	1642(2)	9911(1)	18(1)
C(12)	6418(2)	2459(2)	10403(1)	19(1)
C(13)	7059(2)	3349(2)	9870(1)	17(1)
C(14)	7436(2)	2946(2)	9140(1)	15(1)
C(15)	7847(2)	3779(2)	8592(1)	18(1)
C(16)	7977(2)	4986(2)	8785(1)	21(1)
C(17)	7680(2)	5382(2)	9518(1)	24(1)
C(18)	7201(2)	4566(2)	10050(1)	21(1)
C(19)	5504(2)	342(2)	8752(1)	20(1)
C(20)	4412(2)	716(2)	7595(1)	35(1)
C(21)	9046(2)	-776(2)	10509(1)	25(1)
C(22)	8422(2)	63(2)	10044(1)	19(1)

Table S-3. Bond lengths [≈] and angles [∞] for **3a**.

O(1)-C(3)	1.339(2)
O(1)-C(2)	1.482(2)
O(2)-C(3)	1.215(2)
O(3)-C(19)	1.199(2)
O(4)-C(19)	1.343(2)
O(4)-C(20)	1.446(2)
N(1)-C(3)	1.382(2)
N(1)-C(14)	1.428(2)
N(1)-C(4)	1.484(2)
C(1)-C(2)	1.511(2)
C(1)-H(24)	0.9800
C(1)-H(1)	0.9800
C(1)-H(25)	0.9800
C(2)-C(10)	1.514(3)
C(2)-C(9)	1.520(3)
C(4)-C(5)	1.540(2)
C(4)-C(19)	1.546(2)
C(4)-C(11)	1.548(2)
C(5)-C(22)	1.392(3)
C(5)-C(6)	1.393(3)
C(6)-C(7)	1.388(3)
C(6)-H(20)	0.9500
C(7)-C(8)	1.388(3)
C(7)-H(21)	0.9500
C(8)-C(21)	1.381(3)
C(8)-H(2)	0.9500
C(9)-H(3)	0.9800
C(9)-H(5)	0.9800
C(9)-H(4)	0.9800
C(10)-H(8)	0.9800
C(10)-H(6)	0.9800
C(10)-H(7)	0.9800
C(11)-C(12)	1.524(3)
C(11)-H(16)	0.9900
C(11)-H(9)	0.9900
C(12)-C(13)	1.506(2)

C(12)-H(14)	0.9900
C(12)-H(15)	0.9900
C(13)-C(18)	1.393(3)
C(13)-C(14)	1.402(2)
C(14)-C(15)	1.394(2)
C(15)-C(16)	1.387(3)
C(15)-H(13)	0.9500
C(16)-C(17)	1.385(3)
C(16)-H(10)	0.9500
C(17)-C(18)	1.385(3)
C(17)-H(12)	0.9500
C(18)-H(11)	0.9500
C(20)-H(17)	0.9800
C(20)-H(19)	0.9800
C(20)-H(18)	0.9800
C(21)-C(22)	1.391(3)
C(21)-H(22)	0.9500
C(22)-H(23)	0.9500

C(3)-O(1)-C(2)	121.10(13)
C(19)-O(4)-C(20)	114.36(16)
C(3)-N(1)-C(14)	120.00(14)
C(3)-N(1)-C(4)	118.52(14)
C(14)-N(1)-C(4)	121.44(14)
C(2)-C(1)-H(24)	109.5
C(2)-C(1)-H(1)	109.5
H(24)-C(1)-H(1)	109.5
C(2)-C(1)-H(25)	109.5
H(24)-C(1)-H(25)	109.5
H(1)-C(1)-H(25)	109.5
O(1)-C(2)-C(1)	109.96(14)
O(1)-C(2)-C(10)	109.87(14)
C(1)-C(2)-C(10)	113.17(17)
O(1)-C(2)-C(9)	101.61(15)
C(1)-C(2)-C(9)	110.52(16)
C(10)-C(2)-C(9)	111.10(18)
O(2)-C(3)-O(1)	125.56(16)
O(2)-C(3)-N(1)	124.92(16)

O(1)-C(3)-N(1)	109.51(14)
N(1)-C(4)-C(5)	111.07(14)
N(1)-C(4)-C(19)	108.73(14)
C(5)-C(4)-C(19)	112.94(14)
N(1)-C(4)-C(11)	109.51(14)
C(5)-C(4)-C(11)	107.90(13)
C(19)-C(4)-C(11)	106.55(14)
C(22)-C(5)-C(6)	118.02(17)
C(22)-C(5)-C(4)	120.69(16)
C(6)-C(5)-C(4)	121.07(16)
C(7)-C(6)-C(5)	120.87(18)
C(7)-C(6)-H(20)	119.6
C(5)-C(6)-H(20)	119.6
C(8)-C(7)-C(6)	120.54(19)
C(8)-C(7)-H(21)	119.7
C(6)-C(7)-H(21)	119.7
C(21)-C(8)-C(7)	119.07(19)
C(21)-C(8)-H(2)	120.5
C(7)-C(8)-H(2)	120.5
C(2)-C(9)-H(3)	109.5
C(2)-C(9)-H(5)	109.5
H(3)-C(9)-H(5)	109.5
C(2)-C(9)-H(4)	109.5
H(3)-C(9)-H(4)	109.5
H(5)-C(9)-H(4)	109.5
C(2)-C(10)-H(8)	109.5
C(2)-C(10)-H(6)	109.5
H(8)-C(10)-H(6)	109.5
C(2)-C(10)-H(7)	109.5
H(8)-C(10)-H(7)	109.5
H(6)-C(10)-H(7)	109.5
C(12)-C(11)-C(4)	109.97(14)
C(12)-C(11)-H(16)	109.7
C(4)-C(11)-H(16)	109.7
C(12)-C(11)-H(9)	109.7
C(4)-C(11)-H(9)	109.7
H(16)-C(11)-H(9)	108.2
C(13)-C(12)-C(11)	107.22(15)

C(13)-C(12)-H(14)	110.3
C(11)-C(12)-H(14)	110.3
C(13)-C(12)-H(15)	110.3
C(11)-C(12)-H(15)	110.3
H(14)-C(12)-H(15)	108.5
C(18)-C(13)-C(14)	118.86(16)
C(18)-C(13)-C(12)	122.85(16)
C(14)-C(13)-C(12)	118.11(16)
C(15)-C(14)-C(13)	119.64(16)
C(15)-C(14)-N(1)	121.18(15)
C(13)-C(14)-N(1)	119.17(15)
C(16)-C(15)-C(14)	120.18(17)
C(16)-C(15)-H(13)	119.9
C(14)-C(15)-H(13)	119.9
C(15)-C(16)-C(17)	120.53(18)
C(15)-C(16)-H(10)	119.7
C(17)-C(16)-H(10)	119.7
C(18)-C(17)-C(16)	119.19(17)
C(18)-C(17)-H(12)	120.4
C(16)-C(17)-H(12)	120.4
C(17)-C(18)-C(13)	121.37(17)
C(17)-C(18)-H(11)	119.3
C(13)-C(18)-H(11)	119.3
O(3)-C(19)-O(4)	123.27(17)
O(3)-C(19)-C(4)	126.24(17)
O(4)-C(19)-C(4)	110.39(15)
O(4)-C(20)-H(17)	109.5
O(4)-C(20)-H(19)	109.5
H(17)-C(20)-H(19)	109.5
O(4)-C(20)-H(18)	109.5
H(17)-C(20)-H(18)	109.5
H(19)-C(20)-H(18)	109.5
C(8)-C(21)-C(22)	120.41(19)
C(8)-C(21)-H(22)	119.8
C(22)-C(21)-H(22)	119.8
C(5)-C(22)-C(21)	121.04(18)
C(5)-C(22)-H(23)	119.5
C(21)-C(22)-H(23)	119.5

Table S-4. Anisotropic displacement parameters ($\approx^2$x 10^3) for **3a**. The anisotropic displacement factor exponent takes the form: $-2\pi^2[h^2 a^{*2}U^{11} + ... + 2 h k a^* b^* U^{12}]$

	U^{11}	U^{22}	U^{33}	U^{23}	U^{13}	U^{12}
O(1)	19(1)	13(1)	17(1)	-2(1)	4(1)	1(1)
O(2)	17(1)	21(1)	18(1)	-2(1)	4(1)	-3(1)
O(3)	34(1)	30(1)	30(1)	1(1)	-6(1)	-16(1)
O(4)	23(1)	27(1)	24(1)	-1(1)	-8(1)	3(1)
N(1)	15(1)	12(1)	15(1)	1(1)	3(1)	0(1)
C(1)	24(1)	24(1)	17(1)	-3(1)	0(1)	2(1)
C(2)	25(1)	17(1)	17(1)	-3(1)	4(1)	6(1)
C(3)	16(1)	14(1)	12(1)	1(1)	-1(1)	2(1)
C(4)	14(1)	15(1)	17(1)	1(1)	3(1)	-3(1)
C(5)	19(1)	15(1)	12(1)	-2(1)	4(1)	2(1)
C(6)	24(1)	17(1)	20(1)	-2(1)	4(1)	-1(1)
C(7)	39(1)	17(1)	21(1)	2(1)	8(1)	0(1)
C(8)	36(1)	24(1)	21(1)	5(1)	3(1)	13(1)
C(9)	52(1)	17(1)	35(1)	-4(1)	15(1)	-1(1)
C(10)	39(1)	42(1)	20(1)	-4(1)	-3(1)	24(1)
C(11)	16(1)	18(1)	19(1)	1(1)	5(1)	2(1)
C(12)	21(1)	19(1)	17(1)	-1(1)	4(1)	4(1)
C(13)	14(1)	18(1)	18(1)	0(1)	0(1)	2(1)
C(14)	11(1)	14(1)	19(1)	-1(1)	-1(1)	0(1)
C(15)	16(1)	18(1)	19(1)	1(1)	1(1)	1(1)
C(16)	19(1)	16(1)	29(1)	5(1)	1(1)	1(1)
C(17)	20(1)	14(1)	37(1)	-5(1)	-1(1)	1(1)
C(18)	18(1)	20(1)	24(1)	-6(1)	-1(1)	4(1)
C(19)	16(1)	24(1)	20(1)	-1(1)	2(1)	0(1)
C(20)	31(1)	44(1)	29(1)	-5(1)	-14(1)	4(1)
C(21)	22(1)	34(1)	20(1)	2(1)	2(1)	9(1)
C(22)	21(1)	21(1)	16(1)	2(1)	3(1)	0(1)

Table S-5. Hydrogen coordinates (x 10⁴) and isotropic displacement parameters (≈²x 10 ³) for **3a**.

	x	y	z	U(eq)
H(24)	7937	-125	6766	32
H(1)	9289	-820	6681	32
H(25)	9257	524	7015	32
H(20)	5563	-1162	10028	24
H(21)	6614	-2578	10795	31
H(2)	8813	-2350	11094	32
H(3)	7703	-2171	8317	52
H(5)	8292	-2514	7495	52
H(4)	6997	-1724	7550	52
H(8)	10461	-217	8158	50
H(6)	10380	-1625	7962	50
H(7)	9771	-1124	8745	50
H(16)	4955	2137	9609	21
H(9)	5061	1097	10246	21
H(14)	5884	2888	10788	23
H(15)	7081	1977	10677	23
H(13)	8038	3518	8084	21
H(10)	8273	5546	8411	26
H(12)	7802	6204	9656	28
H(11)	6965	4842	10547	25
H(17)	4791	-9	7364	52
H(19)	4315	1340	7201	52
H(18)	3560	520	7811	52
H(22)	9932	-665	10639	30
H(23)	8889	744	9861	23

Table S-6. Hydrogen bonds for **3a** [≈ and ∞].

D-H...A	d(D-H)	d(H...A)	d(D...A)	<(DHA)
C(1)-H(25)...O(2)	0.98	2.42	3.018(2)	118.5
C(10)-H(8)...O(2)	0.98	2.48	3.009(3)	113.4

4. X-ray data for compound (*R*)-**3g**

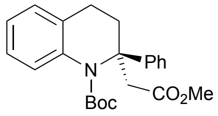

3g

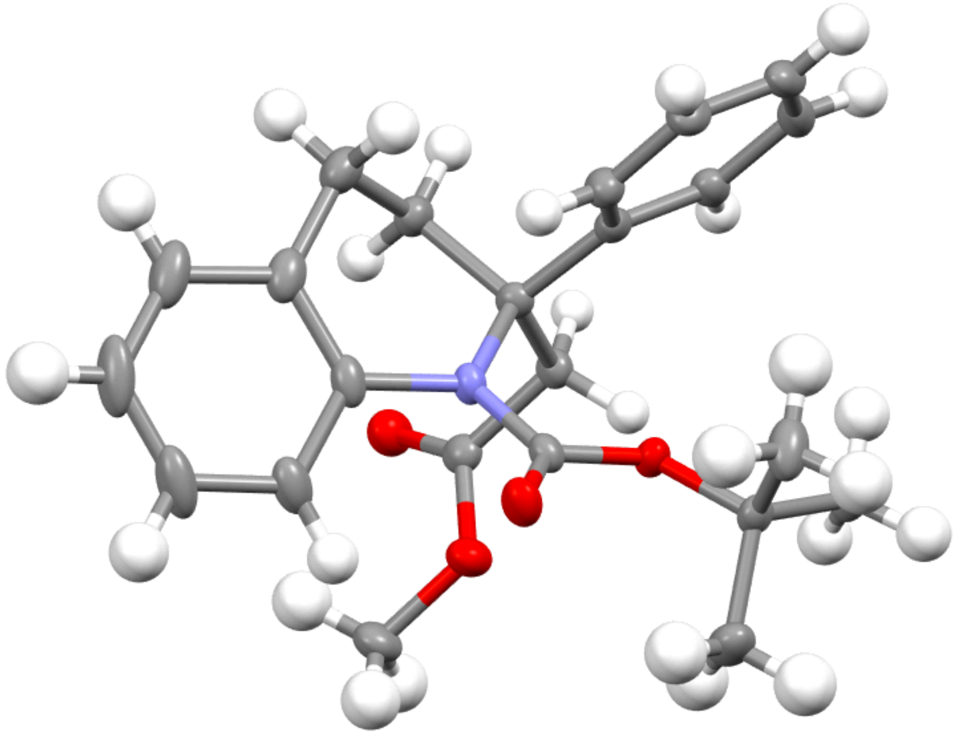

Table S-7. Crystal data and structure refinement for (*R*)-**3g**.

Identification code	CARTERN2_0m	
Empirical formula	C23 H27 N O4	
Formula weight	381.45	
Temperature	97(2) K	
Wavelength	1.54178 ≈	
Crystal system	Monoclinic	
Space group	$P2_1$	
Unit cell dimensions	a = 9.4950(3) ≈	α= 90∞.
	b = 8.8357(3) ≈	β= 90.258(2)∞.
	c = 12.0617(4) ≈	γ = 90∞.
Volume	1011.91(6) $≈^3$	
Z	2	
Density (calculated)	1.252 Mg/m^3	
Absorption coefficient	0.687 mm^{-1}	
F(000)	408	
Crystal size	0.320 x 0.120 x 0.090 mm^3	
Theta range for data collection	3.664 to 66.614∞.	
Index ranges	-11<=h<=11, -10<=k<=9, -14<=l<=14	
Reflections collected	14554	
Independent reflections	3491 [R(int) = 0.0511]	
Completeness to theta = 66.614∞	99.7 %	
Absorption correction	Semi-empirical from equivalents	
Max. and min. transmission	0.91 and 0.87	
Refinement method	Full-matrix least-squares on F^2	
Data / restraints / parameters	3491 / 1 / 258	
Goodness-of-fit on F^2	1.016	
Final R indices [I>2sigma(I)]	R1 = 0.0337, wR2 = 0.0753	
R indices (all data)	R1 = 0.0418, wR2 = 0.0786	
Absolute structure parameter	0.11(12)	
Extinction coefficient	0.0107(11)	
Largest diff. peak and hole	0.161 and -0.141 e.$≈^{-3}$	

CCDC 1578267

Table S-8. Atomic coordinates (x 10^4) and equivalent isotropic displacement parameters (≈^{2}x 10^3)
for (*R*)-**3g**. U(eq) is defined as one third of the trace of the orthogonalized U^{ij} tensor.

	x	y	z	U(eq)
O(1)	4203(2)	4513(2)	7710(1)	21(1)
O(2)	1078(2)	4009(2)	6578(2)	27(1)
O(3)	3201(2)	5368(2)	9292(1)	26(1)
O(4)	423(2)	5412(2)	5114(1)	30(1)
N(1)	2693(2)	6449(2)	7602(2)	20(1)
C(1)	4335(3)	2281(4)	8933(2)	36(1)
C(2)	5165(2)	3421(3)	8261(2)	22(1)
C(3)	3357(2)	5426(3)	8297(2)	19(1)
C(4)	3232(2)	6734(3)	6460(2)	19(1)
C(5)	4808(3)	7055(3)	6515(2)	21(1)
C(6)	5355(3)	7883(3)	7403(2)	25(1)
C(7)	6766(3)	8258(3)	7463(2)	30(1)
C(8)	7678(3)	7794(3)	6643(2)	31(1)
C(9)	-388(3)	3625(3)	6758(2)	33(1)
C(10)	1327(3)	4959(3)	5733(2)	22(1)
C(11)	2860(2)	5392(3)	5678(2)	21(1)
C(12)	6250(3)	4257(4)	8944(3)	37(1)
C(13)	5849(3)	2663(3)	7268(2)	28(1)
C(14)	1612(3)	7385(3)	8058(2)	24(1)
C(15)	1516(3)	8897(3)	7749(2)	28(1)
C(16)	2397(3)	9454(3)	6808(2)	32(1)
C(17)	2502(3)	8164(3)	5982(2)	25(1)
C(18)	494(3)	9802(4)	8252(3)	38(1)
C(19)	-418(3)	9218(4)	9031(2)	45(1)
C(20)	-364(3)	7693(4)	9284(2)	41(1)
C(21)	634(3)	6764(4)	8788(2)	30(1)
C(22)	7164(3)	6955(3)	5757(2)	29(1)
C(23)	5739(3)	6603(3)	5690(2)	24(1)

Table S-9. Bond lengths [≈] and angles [∞] for (*R*)-**3g**.

O(1)-C(3)	1.343(3)
O(1)-C(2)	1.483(3)
O(2)-C(10)	1.342(3)
O(2)-C(9)	1.450(3)
O(3)-C(3)	1.211(3)
O(4)-C(10)	1.204(3)
N(1)-C(3)	1.382(3)
N(1)-C(14)	1.430(3)
N(1)-C(4)	1.493(3)
C(1)-C(2)	1.517(4)
C(1)-H(10)	0.9800
C(1)-H(1)	0.9800
C(1)-H(9)	0.9800
C(2)-C(12)	1.510(4)
C(2)-C(13)	1.520(3)
C(4)-C(5)	1.524(3)
C(4)-C(17)	1.551(3)
C(4)-C(11)	1.555(3)
C(5)-C(23)	1.393(4)
C(5)-C(6)	1.396(4)
C(6)-C(7)	1.382(4)
C(6)-H(25)	0.9500
C(7)-C(8)	1.380(4)
C(7)-H(22)	0.9500
C(8)-C(22)	1.387(4)
C(8)-H(2)	0.9500
C(9)-H(3)	0.9800
C(9)-H(4)	0.9800
C(9)-H(5)	0.9800
C(10)-C(11)	1.507(3)
C(11)-H(27)	0.9900
C(11)-H(26)	0.9900
C(12)-H(6)	0.9800
C(12)-H(8)	0.9800
C(12)-H(7)	0.9800
C(13)-H(12)	0.9800

C(13)-H(11)	0.9800
C(13)-H(13)	0.9800
C(14)-C(15)	1.390(4)
C(14)-C(21)	1.395(4)
C(15)-C(18)	1.398(4)
C(15)-C(16)	1.496(4)
C(16)-C(17)	1.518(4)
C(16)-H(17)	0.9900
C(16)-H(16)	0.9900
C(17)-H(15)	0.9900
C(17)-H(14)	0.9900
C(18)-C(19)	1.380(5)
C(18)-H(18)	0.9500
C(19)-C(20)	1.383(5)
C(19)-H(21)	0.9500
C(20)-C(21)	1.390(4)
C(20)-H(20)	0.9500
C(21)-H(19)	0.9500
C(22)-C(23)	1.390(4)
C(22)-H(24)	0.9500
C(23)-H(23)	0.9500
C(3)-O(1)-C(2)	121.59(18)
C(10)-O(2)-C(9)	115.6(2)
C(3)-N(1)-C(14)	118.09(19)
C(3)-N(1)-C(4)	120.85(18)
C(14)-N(1)-C(4)	120.4(2)
C(2)-C(1)-H(10)	109.5
C(2)-C(1)-H(1)	109.5
H(10)-C(1)-H(1)	109.5
C(2)-C(1)-H(9)	109.5
H(10)-C(1)-H(9)	109.5
H(1)-C(1)-H(9)	109.5
O(1)-C(2)-C(12)	110.1(2)
O(1)-C(2)-C(1)	110.5(2)
C(12)-C(2)-C(1)	112.8(2)
O(1)-C(2)-C(13)	101.47(19)
C(12)-C(2)-C(13)	110.7(2)

C(1)-C(2)-C(13)	110.7(2)
O(3)-C(3)-O(1)	125.0(2)
O(3)-C(3)-N(1)	124.8(2)
O(1)-C(3)-N(1)	110.2(2)
N(1)-C(4)-C(5)	109.38(19)
N(1)-C(4)-C(17)	109.06(19)
C(5)-C(4)-C(17)	107.5(2)
N(1)-C(4)-C(11)	110.68(19)
C(5)-C(4)-C(11)	112.9(2)
C(17)-C(4)-C(11)	107.2(2)
C(23)-C(5)-C(6)	117.6(2)
C(23)-C(5)-C(4)	122.8(2)
C(6)-C(5)-C(4)	119.5(2)
C(7)-C(6)-C(5)	121.5(2)
C(7)-C(6)-H(25)	119.2
C(5)-C(6)-H(25)	119.2
C(8)-C(7)-C(6)	120.2(3)
C(8)-C(7)-H(22)	119.9
C(6)-C(7)-H(22)	119.9
C(7)-C(8)-C(22)	119.4(2)
C(7)-C(8)-H(2)	120.3
C(22)-C(8)-H(2)	120.3
O(2)-C(9)-H(3)	109.5
O(2)-C(9)-H(4)	109.5
H(3)-C(9)-H(4)	109.5
O(2)-C(9)-H(5)	109.5
H(3)-C(9)-H(5)	109.5
H(4)-C(9)-H(5)	109.5
O(4)-C(10)-O(2)	123.5(2)
O(4)-C(10)-C(11)	125.1(2)
O(2)-C(10)-C(11)	111.4(2)
C(10)-C(11)-C(4)	112.54(19)
C(10)-C(11)-H(27)	109.1
C(4)-C(11)-H(27)	109.1
C(10)-C(11)-H(26)	109.1
C(4)-C(11)-H(26)	109.1
H(27)-C(11)-H(26)	107.8
C(2)-C(12)-H(6)	109.5

C(2)-C(12)-H(8)	109.5
H(6)-C(12)-H(8)	109.5
C(2)-C(12)-H(7)	109.5
H(6)-C(12)-H(7)	109.5
H(8)-C(12)-H(7)	109.5
C(2)-C(13)-H(12)	109.5
C(2)-C(13)-H(11)	109.5
H(12)-C(13)-H(11)	109.5
C(2)-C(13)-H(13)	109.5
H(12)-C(13)-H(13)	109.5
H(11)-C(13)-H(13)	109.5
C(15)-C(14)-C(21)	120.3(2)
C(15)-C(14)-N(1)	119.9(2)
C(21)-C(14)-N(1)	119.8(3)
C(14)-C(15)-C(18)	118.6(3)
C(14)-C(15)-C(16)	118.9(2)
C(18)-C(15)-C(16)	122.2(3)
C(15)-C(16)-C(17)	106.8(2)
C(15)-C(16)-H(17)	110.4
C(17)-C(16)-H(17)	110.4
C(15)-C(16)-H(16)	110.4
C(17)-C(16)-H(16)	110.4
H(17)-C(16)-H(16)	108.6
C(16)-C(17)-C(4)	113.4(2)
C(16)-C(17)-H(15)	108.9
C(4)-C(17)-H(15)	108.9
C(16)-C(17)-H(14)	108.9
C(4)-C(17)-H(14)	108.9
H(15)-C(17)-H(14)	107.7
C(19)-C(18)-C(15)	121.3(3)
C(19)-C(18)-H(18)	119.3
C(15)-C(18)-H(18)	119.3
C(18)-C(19)-C(20)	119.5(3)
C(18)-C(19)-H(21)	120.3
C(20)-C(19)-H(21)	120.3
C(19)-C(20)-C(21)	120.3(3)
C(19)-C(20)-H(20)	119.8
C(21)-C(20)-H(20)	119.8

C(20)-C(21)-C(14)	119.7(3)
C(20)-C(21)-H(19)	120.1
C(14)-C(21)-H(19)	120.1
C(8)-C(22)-C(23)	120.3(3)
C(8)-C(22)-H(24)	119.9
C(23)-C(22)-H(24)	119.9
C(22)-C(23)-C(5)	121.0(3)
C(22)-C(23)-H(23)	119.5
C(5)-C(23)-H(23)	119.5

Table S-10. Anisotropic displacement parameters (≈^{2}x 10^3) for (*R*)-**3g**. The anisotropic displacement factor exponent takes the form: $-2\pi^2[h^2 a^{*2}U^{11} + ... + 2 h k a^* b^* U^{12}]$

	U^{11}	U^{22}	U^{33}	U^{23}	U^{13}	U^{12}
O(1)	23(1)	20(1)	19(1)	2(1)	2(1)	5(1)
O(2)	24(1)	27(1)	30(1)	5(1)	-1(1)	-4(1)
O(3)	26(1)	36(1)	17(1)	1(1)	2(1)	5(1)
O(4)	24(1)	34(1)	30(1)	5(1)	-4(1)	0(1)
N(1)	18(1)	23(1)	18(1)	-1(1)	1(1)	4(1)
C(1)	42(2)	29(2)	37(2)	11(1)	10(1)	5(1)
C(2)	22(1)	21(2)	22(1)	5(1)	-1(1)	5(1)
C(3)	16(1)	23(1)	19(1)	-3(1)	1(1)	-1(1)
C(4)	22(1)	20(1)	15(1)	1(1)	2(1)	0(1)
C(5)	21(1)	18(1)	24(1)	4(1)	0(1)	2(1)
C(6)	24(1)	22(2)	29(2)	-1(1)	2(1)	0(1)
C(7)	25(1)	23(2)	40(2)	1(1)	-4(1)	-4(1)
C(8)	19(1)	27(2)	46(2)	12(1)	1(1)	-1(1)
C(9)	25(1)	34(2)	40(2)	6(1)	3(1)	-7(1)
C(10)	25(1)	20(2)	20(1)	-2(1)	2(1)	1(1)
C(11)	22(1)	21(1)	19(1)	-2(1)	2(1)	2(1)
C(12)	30(2)	39(2)	42(2)	-15(1)	-10(1)	10(1)
C(13)	30(2)	25(2)	28(2)	1(1)	3(1)	7(1)
C(14)	18(1)	36(2)	18(1)	-7(1)	-3(1)	5(1)
C(15)	26(1)	30(2)	30(2)	-9(1)	-9(1)	6(1)
C(16)	31(2)	24(2)	40(2)	1(1)	-6(1)	5(1)
C(17)	24(1)	22(2)	27(2)	3(1)	0(1)	3(1)
C(18)	36(2)	40(2)	37(2)	-17(1)	-13(1)	16(1)
C(19)	34(2)	72(3)	29(2)	-24(2)	-7(1)	25(2)
C(20)	26(2)	75(3)	21(2)	-9(2)	0(1)	15(2)
C(21)	21(1)	49(2)	19(1)	0(1)	-2(1)	7(1)
C(22)	23(1)	31(2)	32(2)	11(1)	6(1)	6(1)
C(23)	23(1)	24(2)	24(1)	6(1)	3(1)	2(1)

Table S-11. Hydrogen coordinates (x 10^4) and isotropic displacement parameters (≈^{2}x 10 ^{3}) for (*R*)-**3g**.

	x	y	z	U(eq)
H(10)	3978	2767	9605	54
H(1)	4948	1433	9138	54
H(9)	3542	1904	8489	54
H(25)	4743	8196	7980	30
H(22)	7110	8837	8070	35
H(2)	8649	8046	6684	37
H(3)	-975	4523	6635	49
H(4)	-510	3267	7520	49
H(5)	-671	2826	6239	49
H(27)	3094	5674	4906	25
H(26)	3442	4505	5883	25
H(6)	6737	4995	8476	55
H(8)	6934	3534	9246	55
H(7)	5782	4785	9555	55
H(12)	5121	2183	6810	42
H(11)	6517	1894	7527	42
H(13)	6348	3424	6829	42
H(17)	3346	9745	7077	38
H(16)	1953	10348	6458	38
H(15)	3035	8516	5327	30
H(14)	1542	7889	5729	30
H(18)	425	10839	8053	45
H(21)	-1077	9859	9390	54
H(20)	-1012	7277	9798	49
H(19)	650	5711	8947	36
H(24)	7787	6620	5195	34
H(23)	5396	6045	5072	28

Table S-12. Hydrogen bonds for (*R*)-**3g** [≈ and ∞].

D-H...A	d(D-H)	d(H...A)	d(D...A)	<(DHA)
C(1)-H(10)...O(3)	0.98	2.44	2.965(4)	113.0
C(11)-H(26)...O(1)	0.99	2.32	2.866(3)	114.0
C(12)-H(7)...O(3)	0.98	2.52	3.087(3)	116.5
C(17)-H(14)...O(4)	0.99	2.54	3.300(3)	133.2

5. X-ray data for compound (±)-**10a**

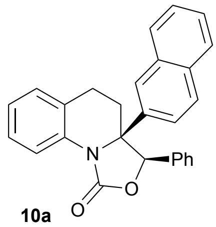

10a

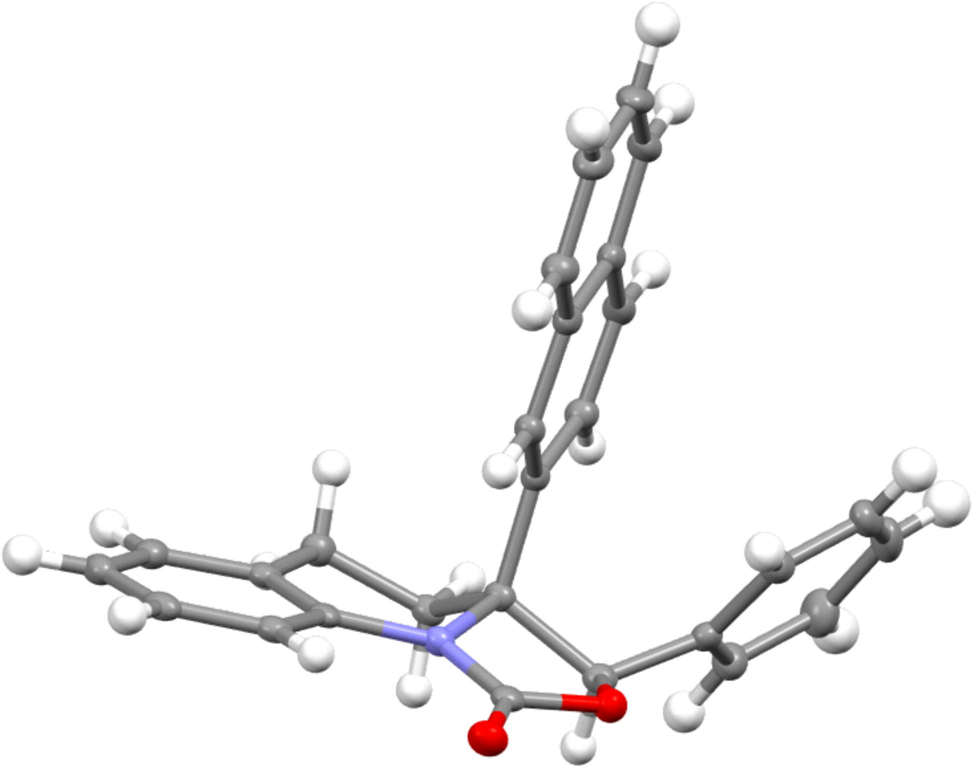

Table S-13. Crystal data and structure refinement for **10a**.

Identification code	OIC276v_0m	
Empirical formula	C27 H21 N O2	
Formula weight	391.45	
Temperature	100(2) K	
Wavelength	1.54178 ≈	
Crystal system	Triclinic	
Space group	P-1	
Unit cell dimensions	a = 7.9717(3) ≈	α= 95.3798(16)∞.
	b = 9.2791(4) ≈	β= 105.3362(17)∞.
	c = 14.2492(6) ≈	γ = 98.2795(16)∞.
Volume	996.24(7) ≈^{3}	
Z	2	
Density (calculated)	1.305 Mg/m^3	
Absorption coefficient	0.647 mm^{-1}	
F(000)	412	
Crystal size	0.180 x 0.080 x 0.050 mm^3	
Theta range for data collection	3.247 to 66.736∞.	
Index ranges	-9<=h<=9, -11<=k<=11, -16<=l<=16	
Reflections collected	29236	
Independent reflections	3496 [R(int) = 0.0356]	
Completeness to theta = 66.736∞	99.1 %	
Absorption correction	Semi-empirical from equivalents	
Max. and min. transmission	0.7528 and 0.6965	
Refinement method	Full-matrix least-squares on F^2	
Data / restraints / parameters	3496 / 0 / 272	
Goodness-of-fit on F^2	1.038	
Final R indices [I>2sigma(I)]	R1 = 0.0321, wR2 = 0.0768	
R indices (all data)	R1 = 0.0387, wR2 = 0.0805	
Extinction coefficient	0.0114(8)	
Largest diff. peak and hole	0.250 and -0.186 e.≈$^{-3}$	

CCDC 1578268

Table S-14. Atomic coordinates (x 10^4) and equivalent isotropic displacement parameters (≈^{2}x 10^3)
for **10a**. U(eq) is defined as one third of the trace of the orthogonalized U^{ij} tensor.

	x	y	z	U(eq)
C(1)	2014(2)	-617(1)	1187(1)	16(1)
C(2)	3547(2)	-786(1)	916(1)	18(1)
C(3)	3937(2)	-2181(1)	747(1)	20(1)
C(4)	2814(2)	-3415(1)	842(1)	21(1)
C(5)	1317(2)	-3235(1)	1128(1)	20(1)
C(6)	896(2)	-1849(1)	1318(1)	16(1)
C(7)	-689(2)	-1706(1)	1691(1)	19(1)
C(8)	-1310(2)	-241(1)	1542(1)	17(1)
C(9)	265(1)	1021(1)	1896(1)	16(1)
C(10)	-199(2)	2493(1)	1542(1)	16(1)
C(11)	2247(2)	2082(1)	1094(1)	16(1)
C(12)	1153(2)	1199(1)	3001(1)	15(1)
C(13)	2953(2)	1413(1)	3384(1)	16(1)
C(14)	3773(2)	1642(1)	4413(1)	18(1)
C(15)	5629(2)	1839(1)	4814(1)	21(1)
C(16)	6367(2)	1997(1)	5809(1)	25(1)
C(17)	5294(2)	1972(1)	6453(1)	26(1)
C(18)	3503(2)	1801(1)	6088(1)	23(1)
C(19)	2693(2)	1637(1)	5062(1)	19(1)
C(20)	838(2)	1454(1)	4651(1)	19(1)
C(21)	90(2)	1230(1)	3660(1)	18(1)
C(22)	-792(2)	3489(1)	2228(1)	18(1)
C(23)	-2601(2)	3402(1)	2088(1)	23(1)
C(24)	-3230(2)	4154(2)	2773(1)	29(1)
C(25)	-2055(2)	4999(1)	3601(1)	29(1)
C(26)	-254(2)	5141(1)	3720(1)	26(1)
C(27)	387(2)	4400(1)	3039(1)	21(1)
N(1)	1539(1)	788(1)	1339(1)	15(1)
O(1)	1423(1)	3171(1)	1361(1)	18(1)
O(2)	3420(1)	2315(1)	706(1)	21(1)

Table S-15. Bond lengths [≈] and angles [∞] for **10a**.

C(1)-C(2)	1.4001(16)
C(1)-C(6)	1.4043(17)
C(1)-N(1)	1.4222(15)
C(2)-C(3)	1.3881(17)
C(2)-H(2)	0.9500
C(3)-C(4)	1.3888(18)
C(3)-H(3)	0.9500
C(4)-C(5)	1.3856(17)
C(4)-H(4)	0.9500
C(5)-C(6)	1.3953(16)
C(5)-H(5)	0.9500
C(6)-C(7)	1.5120(16)
C(7)-C(8)	1.5261(16)
C(7)-H(7A)	0.9900
C(7)-H(7B)	0.9900
C(8)-C(9)	1.5278(16)
C(8)-H(8A)	0.9900
C(8)-H(8B)	0.9900
C(9)-N(1)	1.4717(14)
C(9)-C(12)	1.5290(15)
C(9)-C(10)	1.5589(15)
C(10)-O(1)	1.4519(14)
C(10)-C(22)	1.4941(16)
C(10)-H(10)	1.0000
C(11)-O(2)	1.2090(14)
C(11)-O(1)	1.3598(14)
C(11)-N(1)	1.3678(15)
C(12)-C(13)	1.3711(16)
C(12)-C(21)	1.4223(16)
C(13)-C(14)	1.4202(16)
C(13)-H(13)	0.9500
C(14)-C(15)	1.4156(17)
C(14)-C(19)	1.4204(17)
C(15)-C(16)	1.3678(17)
C(15)-H(15)	0.9500
C(16)-C(17)	1.4096(19)

C(16)-H(16)	0.9500
C(17)-C(18)	1.3641(18)
C(17)-H(17)	0.9500
C(18)-C(19)	1.4174(16)
C(18)-H(18)	0.9500
C(19)-C(20)	1.4175(17)
C(20)-C(21)	1.3620(16)
C(20)-H(20)	0.9500
C(21)-H(21)	0.9500
C(22)-C(23)	1.3920(17)
C(22)-C(27)	1.3951(17)
C(23)-C(24)	1.3863(18)
C(23)-H(23)	0.9500
C(24)-C(25)	1.3835(19)
C(24)-H(24)	0.9500
C(25)-C(26)	1.3855(19)
C(25)-H(25)	0.9500
C(26)-C(27)	1.3837(18)
C(26)-H(26)	0.9500
C(27)-H(27)	0.9500

C(2)-C(1)-C(6)	120.24(11)
C(2)-C(1)-N(1)	121.76(10)
C(6)-C(1)-N(1)	118.00(10)
C(3)-C(2)-C(1)	119.95(11)
C(3)-C(2)-H(2)	120.0
C(1)-C(2)-H(2)	120.0
C(2)-C(3)-C(4)	120.48(11)
C(2)-C(3)-H(3)	119.8
C(4)-C(3)-H(3)	119.8
C(5)-C(4)-C(3)	119.19(11)
C(5)-C(4)-H(4)	120.4
C(3)-C(4)-H(4)	120.4
C(4)-C(5)-C(6)	121.92(11)
C(4)-C(5)-H(5)	119.0
C(6)-C(5)-H(5)	119.0
C(5)-C(6)-C(1)	118.17(10)
C(5)-C(6)-C(7)	119.81(10)

C(1)-C(6)-C(7)	121.99(10)
C(6)-C(7)-C(8)	112.53(9)
C(6)-C(7)-H(7A)	109.1
C(8)-C(7)-H(7A)	109.1
C(6)-C(7)-H(7B)	109.1
C(8)-C(7)-H(7B)	109.1
H(7A)-C(7)-H(7B)	107.8
C(7)-C(8)-C(9)	109.98(9)
C(7)-C(8)-H(8A)	109.7
C(9)-C(8)-H(8A)	109.7
C(7)-C(8)-H(8B)	109.7
C(9)-C(8)-H(8B)	109.7
H(8A)-C(8)-H(8B)	108.2
N(1)-C(9)-C(8)	108.54(9)
N(1)-C(9)-C(12)	110.81(9)
C(8)-C(9)-C(12)	113.42(9)
N(1)-C(9)-C(10)	98.36(8)
C(8)-C(9)-C(10)	112.32(9)
C(12)-C(9)-C(10)	112.34(9)
O(1)-C(10)-C(22)	111.99(9)
O(1)-C(10)-C(9)	102.77(8)
C(22)-C(10)-C(9)	116.48(9)
O(1)-C(10)-H(10)	108.4
C(22)-C(10)-H(10)	108.4
C(9)-C(10)-H(10)	108.4
O(2)-C(11)-O(1)	121.86(10)
O(2)-C(11)-N(1)	128.90(11)
O(1)-C(11)-N(1)	109.24(9)
C(13)-C(12)-C(21)	118.62(10)
C(13)-C(12)-C(9)	122.12(10)
C(21)-C(12)-C(9)	119.16(10)
C(12)-C(13)-C(14)	121.83(10)
C(12)-C(13)-H(13)	119.1
C(14)-C(13)-H(13)	119.1
C(15)-C(14)-C(13)	122.25(11)
C(15)-C(14)-C(19)	118.82(11)
C(13)-C(14)-C(19)	118.91(11)
C(16)-C(15)-C(14)	120.59(12)

C(16)-C(15)-H(15)	119.7
C(14)-C(15)-H(15)	119.7
C(15)-C(16)-C(17)	120.64(12)
C(15)-C(16)-H(16)	119.7
C(17)-C(16)-H(16)	119.7
C(18)-C(17)-C(16)	120.08(11)
C(18)-C(17)-H(17)	120.0
C(16)-C(17)-H(17)	120.0
C(17)-C(18)-C(19)	120.83(12)
C(17)-C(18)-H(18)	119.6
C(19)-C(18)-H(18)	119.6
C(18)-C(19)-C(20)	122.68(11)
C(18)-C(19)-C(14)	119.02(11)
C(20)-C(19)-C(14)	118.29(11)
C(21)-C(20)-C(19)	121.38(11)
C(21)-C(20)-H(20)	119.3
C(19)-C(20)-H(20)	119.3
C(20)-C(21)-C(12)	120.92(11)
C(20)-C(21)-H(21)	119.5
C(12)-C(21)-H(21)	119.5
C(23)-C(22)-C(27)	119.34(11)
C(23)-C(22)-C(10)	117.85(10)
C(27)-C(22)-C(10)	122.59(11)
C(24)-C(23)-C(22)	120.46(12)
C(24)-C(23)-H(23)	119.8
C(22)-C(23)-H(23)	119.8
C(25)-C(24)-C(23)	119.93(12)
C(25)-C(24)-H(24)	120.0
C(23)-C(24)-H(24)	120.0
C(24)-C(25)-C(26)	119.74(12)
C(24)-C(25)-H(25)	120.1
C(26)-C(25)-H(25)	120.1
C(27)-C(26)-C(25)	120.73(12)
C(27)-C(26)-H(26)	119.6
C(25)-C(26)-H(26)	119.6
C(26)-C(27)-C(22)	119.67(12)
C(26)-C(27)-H(27)	120.2
C(22)-C(27)-H(27)	120.2

C(11)-N(1)-C(1)	127.59(9)
C(11)-N(1)-C(9)	111.19(9)
C(1)-N(1)-C(9)	120.98(9)
C(11)-O(1)-C(10)	108.12(9)

Table S-16. Anisotropic displacement parameters (≈^{2}x 10^3) for **10a**. The anisotropic displacement factor exponent takes the form: $-2\pi^2[h^2 a^{*2}U^{11} + ... + 2 h k a^* b^* U^{12}]$

	U11	U22	U33	U23	U13	U12
C(1)	16(1)	19(1)	9(1)	1(1)	0(1)	4(1)
C(2)	17(1)	23(1)	12(1)	3(1)	2(1)	3(1)
C(3)	18(1)	29(1)	13(1)	3(1)	3(1)	8(1)
C(4)	25(1)	22(1)	15(1)	2(1)	3(1)	9(1)
C(5)	22(1)	20(1)	15(1)	3(1)	2(1)	3(1)
C(6)	17(1)	20(1)	10(1)	2(1)	1(1)	3(1)
C(7)	18(1)	18(1)	18(1)	3(1)	5(1)	0(1)
C(8)	15(1)	20(1)	15(1)	1(1)	4(1)	2(1)
C(9)	16(1)	18(1)	14(1)	2(1)	5(1)	3(1)
C(10)	15(1)	19(1)	15(1)	3(1)	3(1)	1(1)
C(11)	17(1)	19(1)	10(1)	1(1)	0(1)	1(1)
C(12)	18(1)	13(1)	14(1)	2(1)	4(1)	2(1)
C(13)	20(1)	15(1)	15(1)	2(1)	6(1)	2(1)
C(14)	22(1)	13(1)	16(1)	2(1)	3(1)	3(1)
C(15)	22(1)	21(1)	19(1)	3(1)	3(1)	2(1)
C(16)	24(1)	23(1)	23(1)	3(1)	-3(1)	1(1)
C(17)	36(1)	22(1)	14(1)	2(1)	-2(1)	5(1)
C(18)	33(1)	20(1)	15(1)	2(1)	6(1)	6(1)
C(19)	26(1)	13(1)	16(1)	2(1)	5(1)	4(1)
C(20)	26(1)	18(1)	17(1)	4(1)	10(1)	5(1)
C(21)	18(1)	18(1)	18(1)	4(1)	5(1)	4(1)
C(22)	22(1)	16(1)	16(1)	5(1)	5(1)	4(1)
C(23)	23(1)	21(1)	23(1)	2(1)	4(1)	5(1)
C(24)	27(1)	27(1)	38(1)	4(1)	14(1)	9(1)
C(25)	42(1)	21(1)	30(1)	2(1)	19(1)	9(1)
C(26)	37(1)	18(1)	20(1)	0(1)	7(1)	1(1)
C(27)	24(1)	18(1)	19(1)	3(1)	4(1)	2(1)
N(1)	16(1)	16(1)	14(1)	2(1)	5(1)	2(1)
O(1)	20(1)	17(1)	18(1)	3(1)	7(1)	2(1)
O(2)	21(1)	24(1)	19(1)	4(1)	9(1)	1(1)

Table S-17. Hydrogen coordinates (x 10^4) and isotropic displacement parameters (≈^{2}x 10 ^{3}) for **10a**.

	x	y	z	U(eq)
H(2)	4320	52	848	21
H(3)	4979	-2293	565	24
H(4)	3069	-4371	713	25
H(5)	554	-4080	1197	23
H(7A)	-381	-1801	2400	22
H(7B)	-1669	-2519	1343	22
H(8A)	-2177	-108	1914	20
H(8B)	-1898	-241	837	20
H(10)	-1145	2257	902	20
H(13)	3672	1409	2948	20
H(15)	6368	1862	4389	26
H(16)	7614	2126	6068	30
H(17)	5820	2074	7142	31
H(18)	2790	1793	6527	27
H(20)	100	1487	5076	23
H(21)	-1159	1093	3406	22
H(23)	-3410	2824	1520	27
H(24)	-4466	4089	2674	35
H(25)	-2482	5481	4086	35
H(26)	549	5753	4276	31
H(27)	1623	4512	3123	25

Table S-18. Hydrogen bonds for **10a** [≈ and ∞].

D-H...A	d(D-H)	d(H...A)	d(D...A)	<(DHA)
C(2)-H(2)...O(2)	0.95	2.32	2.9346(15)	121.5

6. X-ray data for compound (*S*)-**11**

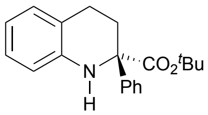

11

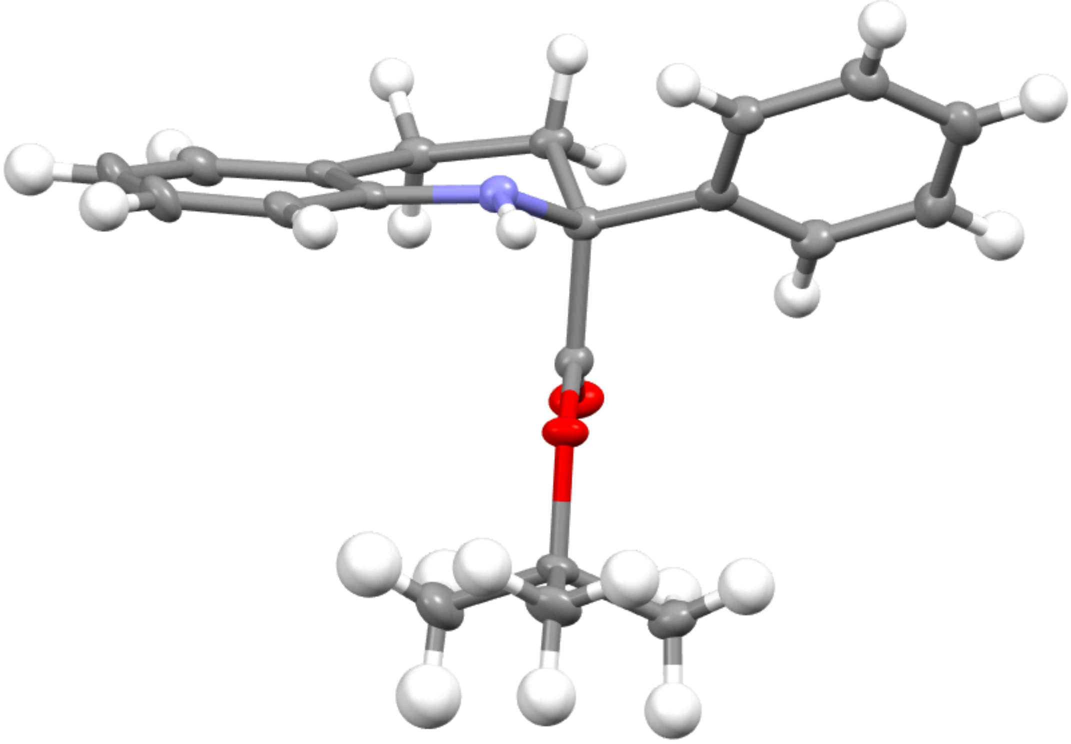

Table S-19. Crystal data and structure refinement for (*S*)-**11**.

Identification code	OIC270A	
Empirical formula	C20 H23 N O2	
Formula weight	309.39	
Temperature	100(2) K	
Wavelength	1.54178 ≈	
Crystal system	Orthorhombic	
Space group	$P2_12_12_1$	
Unit cell dimensions	a = 6.1094(3) ≈	α= 90∞.
	b = 14.2823(6) ≈	β= 90∞.
	c = 19.8409(8) ≈	γ = 90∞.
Volume	1731.24(13) $≈^3$	
Z	4	
Density (calculated)	1.187 Mg/m^3	
Absorption coefficient	0.599 mm^{-1}	
F(000)	664	
Crystal size	0.200 x 0.200 x 0.180 mm^3	
Theta range for data collection	3.813 to 66.624∞.	
Index ranges	-6<=h<=7, -17<=k<=15, -23<=l<=21	
Reflections collected	11998	
Independent reflections	3043 [R(int) = 0.0313]	
Completeness to theta = 66.624∞	99.4 %	
Absorption correction	Semi-empirical from equivalents	
Max. and min. transmission	0.92 and 0.82	
Refinement method	Full-matrix least-squares on F^2	
Data / restraints / parameters	3043 / 0 / 211	
Goodness-of-fit on F^2	1.091	
Final R indices [I>2sigma(I)]	R1 = 0.0285, wR2 = 0.0687	
R indices (all data)	R1 = 0.0303, wR2 = 0.0698	
Absolute structure parameter	0.08(7)	
Extinction coefficient	n/a	
Largest diff. peak and hole	0.152 and -0.169 e.$≈^{-3}$	

CCDC 1578269

Table S-20. Atomic coordinates (x 10^4) and equivalent isotropic displacement parameters (≈^{2}x 10^3) for (*S*)-**11**. U(eq) is defined as one third of the trace of the orthogonalized U^{ij} tensor.

	x	y	z	U(eq)
O(1)	7516(2)	5874(1)	4361(1)	18(1)
O(2)	4309(2)	5207(1)	4663(1)	24(1)
N(1)	9681(2)	4304(1)	4728(1)	16(1)
C(1)	5041(3)	7229(1)	4350(1)	27(1)
C(2)	6521(3)	6613(1)	3932(1)	20(1)
C(3)	6281(3)	5248(1)	4683(1)	17(1)
C(4)	7740(3)	4579(1)	5103(1)	16(1)
C(5)	9418(3)	3718(1)	4167(1)	16(1)
C(6)	11036(3)	3694(1)	3668(1)	21(1)
C(7)	10914(3)	3053(1)	3148(1)	26(1)
C(8)	9149(3)	2440(2)	3102(1)	29(1)
C(9)	5353(4)	6166(2)	3338(1)	35(1)
C(10)	8522(3)	7152(1)	3703(1)	29(1)
C(11)	6450(3)	3687(1)	5280(1)	17(1)
C(12)	5878(3)	3100(1)	4662(1)	20(1)
C(13)	7622(3)	3107(1)	4125(1)	18(1)
C(14)	7523(3)	2484(1)	3586(1)	24(1)
C(15)	8359(3)	5062(1)	5762(1)	17(1)
C(16)	10432(3)	4961(1)	6043(1)	20(1)
C(17)	10877(3)	5319(1)	6680(1)	24(1)
C(18)	9274(3)	5787(1)	7041(1)	24(1)
C(19)	7218(3)	5901(1)	6758(1)	25(1)
C(20)	6765(3)	5543(1)	6127(1)	22(1)

Table S-21. Bond lengths [≈] and angles [∞] for (*S*)-**11**.

O(1)-C(3)	1.334(2)
O(1)-C(2)	1.485(2)
O(2)-C(3)	1.207(2)
N(1)-C(5)	1.401(2)
N(1)-C(4)	1.454(2)
N(1)-H(1N)	0.9023
C(1)-C(2)	1.510(3)
C(1)-H(5)	0.9800
C(1)-H(1)	0.9800
C(1)-H(6)	0.9800
C(2)-C(10)	1.515(3)
C(2)-C(9)	1.518(3)
C(3)-C(4)	1.549(2)
C(4)-C(15)	1.527(2)
C(4)-C(11)	1.538(2)
C(5)-C(6)	1.400(2)
C(5)-C(13)	1.404(2)
C(6)-C(7)	1.380(3)
C(6)-H(14)	0.9500
C(7)-C(8)	1.392(3)
C(7)-H(15)	0.9500
C(8)-C(14)	1.383(3)
C(8)-H(2)	0.9500
C(9)-H(24)	0.9800
C(9)-H(4)	0.9800
C(9)-H(3)	0.9800
C(10)-H(9)	0.9800
C(10)-H(7)	0.9800
C(10)-H(8)	0.9800
C(11)-C(12)	1.525(2)
C(11)-H(17)	0.9900
C(11)-H(18)	0.9900
C(12)-C(13)	1.508(2)
C(12)-H(16)	0.9900
C(12)-H(10)	0.9900
C(13)-C(14)	1.392(3)

C(14)-H(11)	0.9500
C(15)-C(16)	1.391(2)
C(15)-C(20)	1.394(2)
C(16)-C(17)	1.390(3)
C(16)-H(23)	0.9500
C(17)-C(18)	1.385(3)
C(17)-H(22)	0.9500
C(18)-C(19)	1.386(3)
C(18)-H(19)	0.9500
C(19)-C(20)	1.380(3)
C(19)-H(21)	0.9500
C(20)-H(20)	0.9500

C(3)-O(1)-C(2)	121.31(13)
C(5)-N(1)-C(4)	118.28(13)
C(5)-N(1)-H(1N)	112.6
C(4)-N(1)-H(1N)	114.6
C(2)-C(1)-H(5)	109.5
C(2)-C(1)-H(1)	109.5
H(5)-C(1)-H(1)	109.5
C(2)-C(1)-H(6)	109.5
H(5)-C(1)-H(6)	109.5
H(1)-C(1)-H(6)	109.5
O(1)-C(2)-C(1)	110.14(13)
O(1)-C(2)-C(10)	101.72(14)
C(1)-C(2)-C(10)	110.62(15)
O(1)-C(2)-C(9)	109.77(15)
C(1)-C(2)-C(9)	112.95(17)
C(10)-C(2)-C(9)	111.08(16)
O(2)-C(3)-O(1)	125.53(16)
O(2)-C(3)-C(4)	124.21(15)
O(1)-C(3)-C(4)	110.26(13)
N(1)-C(4)-C(15)	111.00(13)
N(1)-C(4)-C(11)	108.16(13)
C(15)-C(4)-C(11)	107.78(13)
N(1)-C(4)-C(3)	111.15(13)
C(15)-C(4)-C(3)	108.87(13)
C(11)-C(4)-C(3)	109.81(13)

C(6)-C(5)-N(1)	119.70(15)
C(6)-C(5)-C(13)	119.58(16)
N(1)-C(5)-C(13)	120.57(15)
C(7)-C(6)-C(5)	120.46(16)
C(7)-C(6)-H(14)	119.8
C(5)-C(6)-H(14)	119.8
C(6)-C(7)-C(8)	120.52(17)
C(6)-C(7)-H(15)	119.7
C(8)-C(7)-H(15)	119.7
C(14)-C(8)-C(7)	118.82(17)
C(14)-C(8)-H(2)	120.6
C(7)-C(8)-H(2)	120.6
C(2)-C(9)-H(24)	109.5
C(2)-C(9)-H(4)	109.5
H(24)-C(9)-H(4)	109.5
C(2)-C(9)-H(3)	109.5
H(24)-C(9)-H(3)	109.5
H(4)-C(9)-H(3)	109.5
C(2)-C(10)-H(9)	109.5
C(2)-C(10)-H(7)	109.5
H(9)-C(10)-H(7)	109.5
C(2)-C(10)-H(8)	109.5
H(9)-C(10)-H(8)	109.5
H(7)-C(10)-H(8)	109.5
C(12)-C(11)-C(4)	112.90(14)
C(12)-C(11)-H(17)	109.0
C(4)-C(11)-H(17)	109.0
C(12)-C(11)-H(18)	109.0
C(4)-C(11)-H(18)	109.0
H(17)-C(11)-H(18)	107.8
C(13)-C(12)-C(11)	113.75(14)
C(13)-C(12)-H(16)	108.8
C(11)-C(12)-H(16)	108.8
C(13)-C(12)-H(10)	108.8
C(11)-C(12)-H(10)	108.8
H(16)-C(12)-H(10)	107.7
C(14)-C(13)-C(5)	118.51(17)
C(14)-C(13)-C(12)	120.54(15)

C(5)-C(13)-C(12)	120.91(15)
C(8)-C(14)-C(13)	122.07(17)
C(8)-C(14)-H(11)	119.0
C(13)-C(14)-H(11)	119.0
C(16)-C(15)-C(20)	118.60(16)
C(16)-C(15)-C(4)	121.48(15)
C(20)-C(15)-C(4)	119.64(15)
C(17)-C(16)-C(15)	120.29(17)
C(17)-C(16)-H(23)	119.9
C(15)-C(16)-H(23)	119.9
C(18)-C(17)-C(16)	120.64(17)
C(18)-C(17)-H(22)	119.7
C(16)-C(17)-H(22)	119.7
C(17)-C(18)-C(19)	119.14(17)
C(17)-C(18)-H(19)	120.4
C(19)-C(18)-H(19)	120.4
C(20)-C(19)-C(18)	120.43(17)
C(20)-C(19)-H(21)	119.8
C(18)-C(19)-H(21)	119.8
C(19)-C(20)-C(15)	120.89(17)
C(19)-C(20)-H(20)	119.6
C(15)-C(20)-H(20)	119.6

Table S-22. Anisotropic displacement parameters (≈^{2}x 10^3) for (*S*)-**11**. The anisotropic displacement factor exponent takes the form: $-2\pi^2$[h^2 $a^{*2}U^{11}$ + ... + 2 h k a* b* U^{12}]

	U^{11}	U^{22}	U^{33}	U^{23}	U^{13}	U^{12}
O(1)	16(1)	17(1)	22(1)	7(1)	0(1)	0(1)
O(2)	13(1)	23(1)	35(1)	7(1)	-2(1)	-1(1)
N(1)	12(1)	16(1)	21(1)	2(1)	2(1)	-2(1)
C(1)	30(1)	21(1)	31(1)	4(1)	3(1)	5(1)
C(2)	23(1)	19(1)	17(1)	6(1)	-2(1)	1(1)
C(3)	16(1)	15(1)	20(1)	1(1)	1(1)	-1(1)
C(4)	12(1)	16(1)	20(1)	2(1)	1(1)	0(1)
C(5)	16(1)	16(1)	17(1)	5(1)	-3(1)	1(1)
C(6)	18(1)	25(1)	18(1)	6(1)	-4(1)	-2(1)
C(7)	29(1)	35(1)	14(1)	2(1)	2(1)	-2(1)
C(8)	39(1)	32(1)	15(1)	-1(1)	-4(1)	-4(1)
C(9)	48(1)	34(1)	24(1)	1(1)	-11(1)	-4(1)
C(10)	30(1)	30(1)	27(1)	13(1)	2(1)	-3(1)
C(11)	13(1)	15(1)	24(1)	5(1)	1(1)	-1(1)
C(12)	16(1)	18(1)	25(1)	4(1)	-2(1)	-3(1)
C(13)	20(1)	17(1)	18(1)	7(1)	-5(1)	0(1)
C(14)	27(1)	25(1)	21(1)	5(1)	-7(1)	-6(1)
C(15)	17(1)	13(1)	22(1)	6(1)	3(1)	-2(1)
C(16)	18(1)	20(1)	23(1)	2(1)	2(1)	1(1)
C(17)	20(1)	28(1)	25(1)	4(1)	-2(1)	-2(1)
C(18)	29(1)	22(1)	20(1)	2(1)	1(1)	-4(1)
C(19)	25(1)	24(1)	26(1)	-2(1)	6(1)	1(1)
C(20)	16(1)	22(1)	26(1)	2(1)	1(1)	2(1)

Table S-23. Hydrogen coordinates (x 10^4) and isotropic displacement parameters (≈^{2}x 10 ^{3}) for (*S*)-**11**.

	x	y	z	U(eq)
H(1N)	10642	4772	4651	20
H(5)	3752	6870	4491	40
H(1)	4576	7769	4082	40
H(6)	5835	7447	4750	40
H(14)	12226	4121	3687	25
H(15)	12043	3031	2820	31
H(2)	9063	1999	2745	34
H(24)	6346	5729	3112	53
H(4)	4899	6654	3021	53
H(3)	4061	5827	3500	53
H(9)	9271	7417	4096	43
H(7)	8071	7660	3400	43
H(8)	9518	6730	3463	43
H(17)	7329	3303	5595	21
H(18)	5082	3867	5514	21
H(16)	5624	2445	4807	24
H(10)	4496	3337	4466	24
H(11)	6300	2077	3550	29
H(23)	11548	4647	5798	24
H(22)	12293	5242	6869	29
H(19)	9581	6026	7478	29
H(21)	6114	6229	6999	30
H(20)	5348	5625	5939	26

Table S-24. Hydrogen bonds for (*S*)-**11** [≈ and ∞].

D-H...A	d(D-H)	d(H...A)	d(D...A)	<(DHA)
C(9)-H(3)...O(2)	0.98	2.48	3.032(2)	115.5
C(1)-H(5)...O(2)	0.98	2.42	2.987(2)	116.1
C(9)-H(3)...O(2)	0.98	2.48	3.032(2)	115.5
C(1)-H(5)...O(2)	0.98	2.42	2.987(2)	116.1
N(1)-H(1N)...O(2)#1	0.90	2.33	3.1104(18)	145.4
N(1)-H(1N)...O(2)#1	0.90	2.33	3.1104(18)	145.4
C(1)-H(5)...O(2)	0.98	2.42	2.987(2)	116.1
C(9)-H(3)...O(2)	0.98	2.48	3.032(2)	115.5

Symmetry transformations used to generate equivalent atoms:

#1 x+1,y,z

7. Variable temperature NMR spectra for tetrahydroquinoline **2a**

Tetrahydroquinoline **2a** (40 mg, 0.13 mmol) in d_8-THF (0.7 mL) was placed in an NMR tube in an NMR spectrometer and was warmed gradually from –78 °C. Warming allowed coalescence of several signals; for example, for the *t*-butyl protons at about 1.4 ppm, which occurred at approximately –38 °C as shown below.

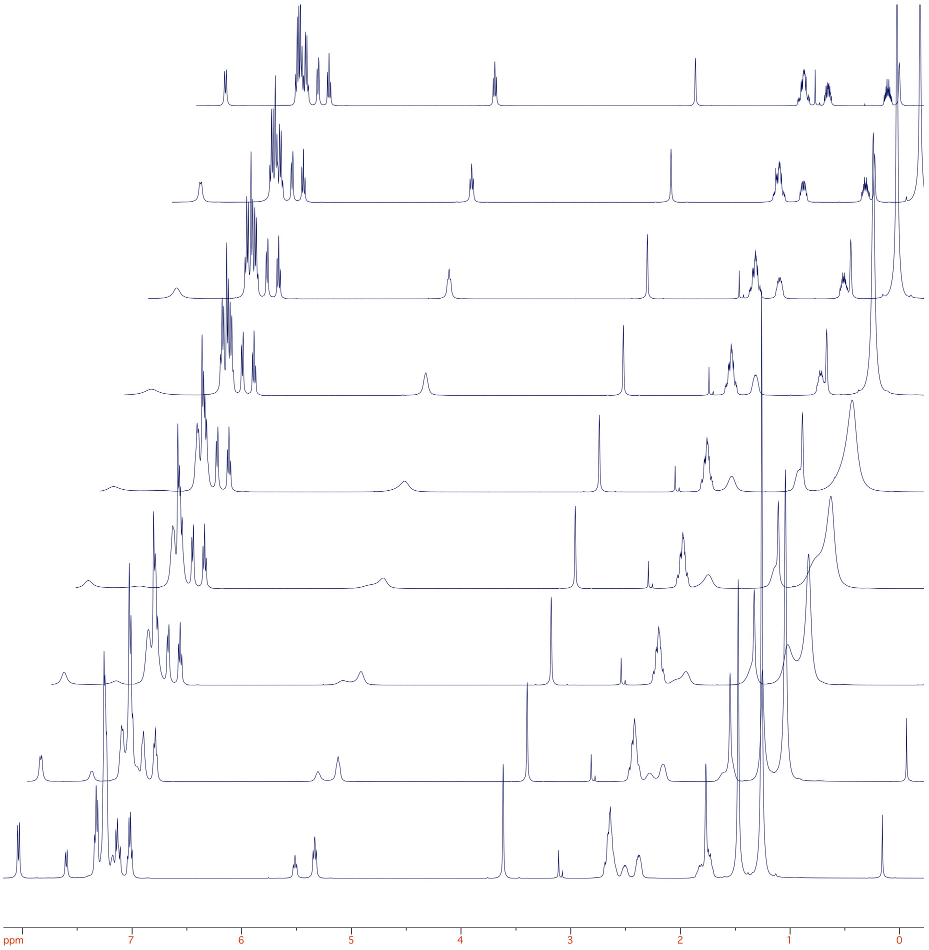

Using line shape analysis to determine $\Delta H^{\ddagger}$ and $\Delta S^{\ddagger}$:

Rate constant at temperatures pre-coalescence: $k = [(\Delta\nu^{o}_{AB})^2 - (\Delta\nu_{AB})^2]^{1/2}\pi/\sqrt{2}$

and at coalescence (~ 231 K): $k = (\Delta\nu^{o}_{AB})\pi/\sqrt{2}$

and post-coalescence: $k = (\Delta\nu^{o}_{AB})^2\pi/2(\Delta\nu_{1/2} - \Delta\nu^{o}_{1/2})$

These equations gave the following data:

Table S-25. Parameters for the line shape analysis.

T/K	1/T	$\Delta\nu^{o}_{AB}$	$\Delta\nu_{AB}$	$\Delta\nu_{1/2}$	k	ln(k/T)
204.5	0.004890	107	107		–	–
213	0.004695	107	105.86		34.605	-1.81731
217.4	0.004600	107	104.29		53.157	-1.40849
223	0.004484	107	98.9		90.720	-0.89939
226.3	0.004419	107	84.22		146.615	-0.43405
231	0.004329	107	50.2		209.911	0.02857
235	0.004255	107			237.694	0.33643
240	0.004167	107		35.45	542.096	0.81480
249	0.004016	107		15.224	1388.836	1.71877
257.9	0.003877	107		8.028	3126.027	2.49495
267	0.003745	107		5.08	6411.420	3.17859
276	0.003623	107		3.687	12736.567	3.83183

$\Delta\nu^{o}_{1/2}$ (width of baseline peak 2.275 Hz)

An Eyring plot of 1/T against ln(k/T) can be drawn for these data from which the enthalpy and entropy can be obtained. However, these values assume an equal proportion (1:1) of each rotamer and therefore the values obtained would only be approximate. The calculated $\Delta G^{\ddagger} \approx 45$ kJ/mol at –78 °C from this line shape analysis approach, which equates to $k = 2.8\ s^{-1}$, $t_{1/2} = 0.25$ s does tally with a fast rate of rotation (and lithiation) at low temperature.

A better approach is to carry out dynamic NMR studies and to determine rate constants from best fit spectra. This was carried out using the software iNMR. The major:minor rotamers from integration are 1.8:1 ratio. The major rotamer was assigned a rate constant k_1 (determined from DNMR) as it converts to the minor rotamer and the reverse rate constant k_{-1} ($1.8 \times k_1$) according to the following equation and data:

$$\text{major rotamer} \underset{k_{-1}}{\overset{k_1}{\rightleftharpoons}} \text{minor rotamer}$$

T/K	1/T	k_1	ln(k_1/T)		k_{-1}	ln(k_{-1}/T)
213	0.004695	10	-3.058707073		18	-2.47092
217.4	0.004600	22	-2.290696521		39.6	-1.70291
223	0.004484	50	-1.495148766		90	-0.90736
226.3	0.004419	83	-1.003020945		149.4	-0.41523
231	0.004329	133	-0.552068582		239.4	0.03572
235	0.004255	223	-0.052413743		401.4	0.53537
240	0.004167	356	0.394291808		640.8	0.98208
249	0.004016	941	1.329490243		1693.8	1.91728
257.9	0.003877	3034	2.465065247		5461.2	3.05285

Eyring plots of 1/T against ln(k/T) for each of the forward and backward processes are shown below (with error bars for ± 4 °C assuming variability of the temperature in the probe):

Forward direction (k_1):

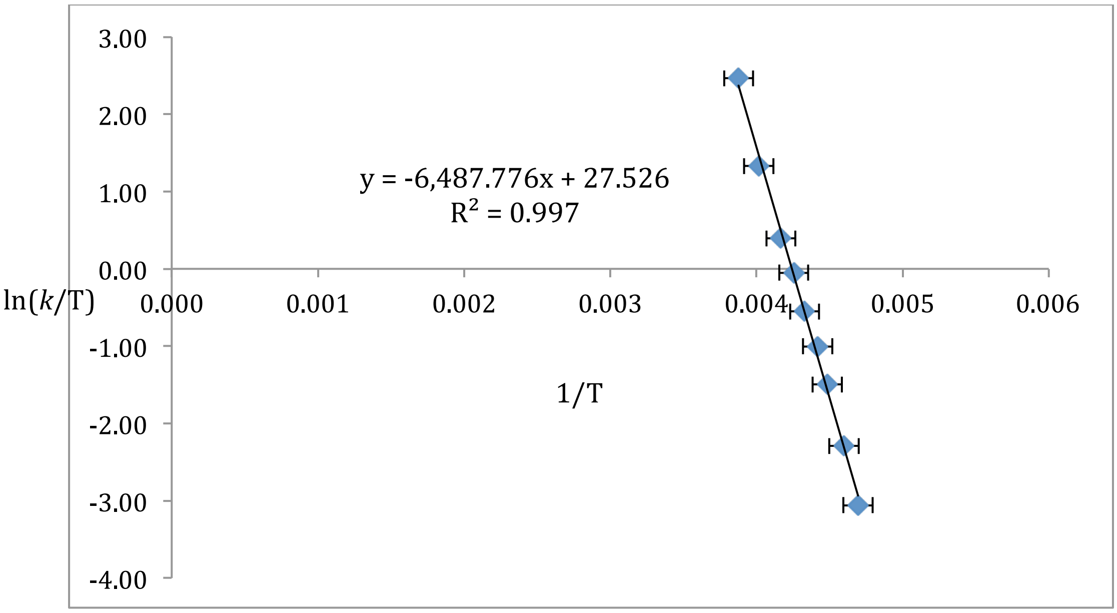

Reverse direction (k_{-1}):

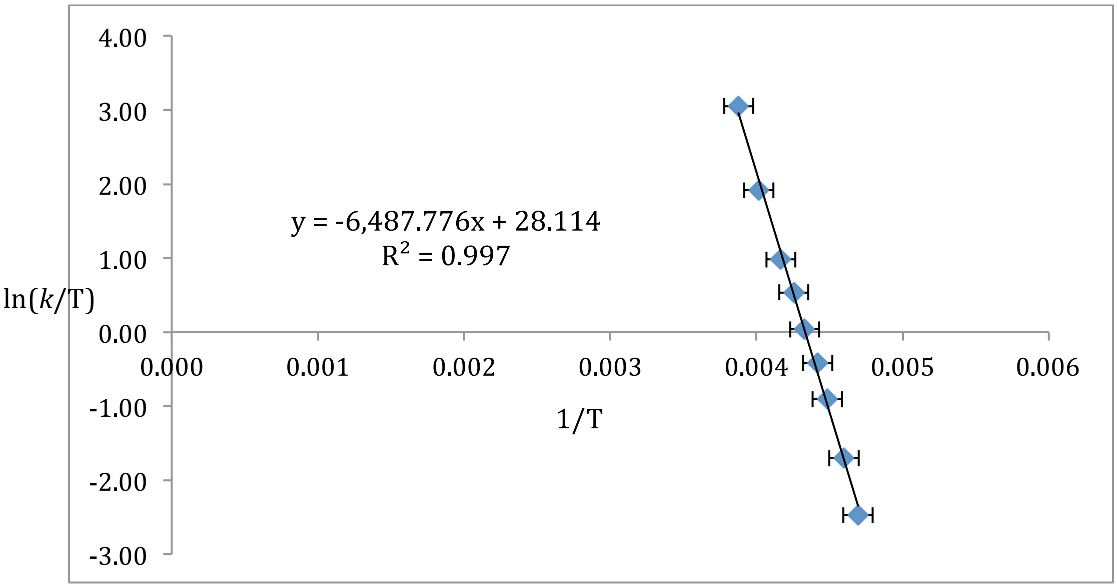

From these Eyring plots:

Forward direction $\Delta H^{\ddagger} \approx 54$ kJ/mol and $\Delta S^{\ddagger} \approx 31$ J/K·mol

$\Delta G^{\ddagger} \approx 47.8$ kJ/mol at –78 °C, that equates to $k = 0.6\ s^{-1}$, $t_{1/2} = 1.1$ s

Reverse direction $\Delta H^{\ddagger} \approx 54$ kJ/mol and $\Delta S^{\ddagger} \approx 36$ J/K·mol

$\Delta G^{\ddagger} \approx 46.9$ kJ/mol at –78 °C, that equates to $k = 1.1\ s^{-1}$, $t_{1/2} = 0.6$ s

8. CSP-HPLC traces

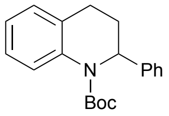

2a

Cellulose–2 column:

	R_t/min	Area/ mV.s	Area/%
1	5.270	6339.820	50.0
2	6.227	6336.386	50.0
	total	12676.206	100.0

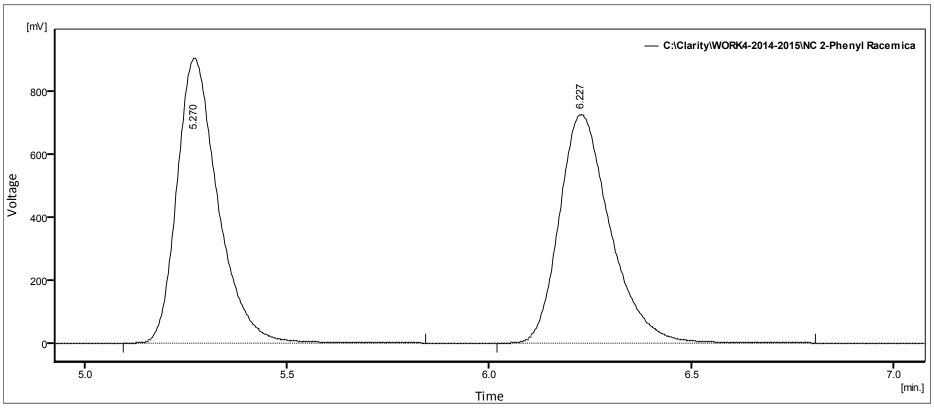

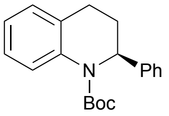

2a (er 98:2)

	R_t/min	Area/ mV.s	Area/%
1	5.220	104.217	1.6
2	6.123	6285.032	98.4
	total	6389.349	100.0

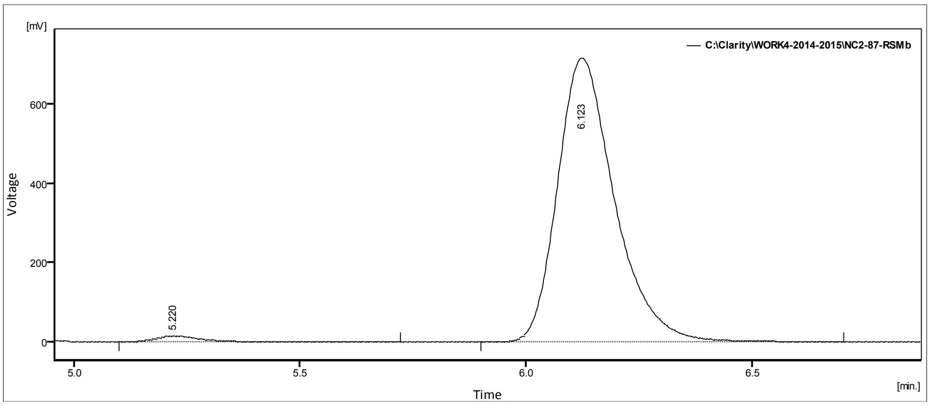

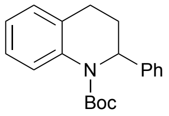

2a

Cellulose–1 column:

	R_t/min	Area/ mV.s	Area/%
1	6.903	5158.494	48.8
2	12.637	5412.822	51.2
	total	11736.328	100.0

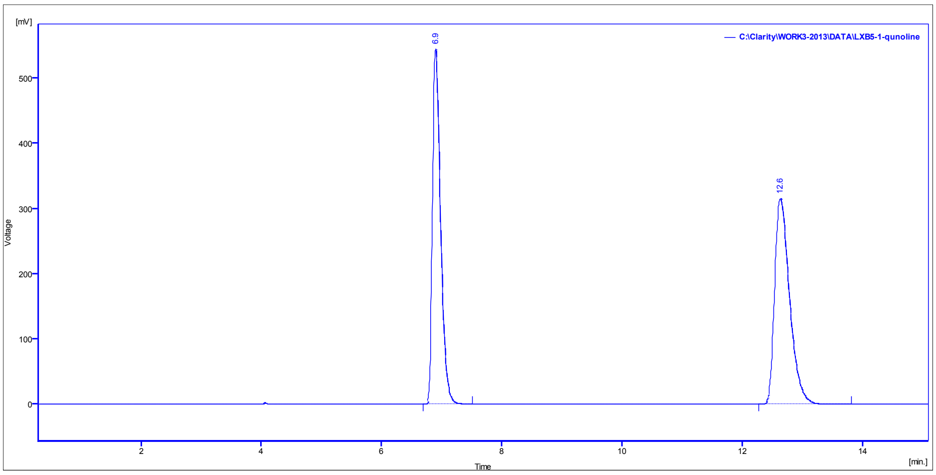

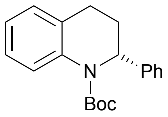

2a (er 97:3)

	R_t/min	Area/ mV.s	Area/%
1	6.890	376.487	3.2
2	12.837	11359.841	96.8
	total	11736.328	100.0

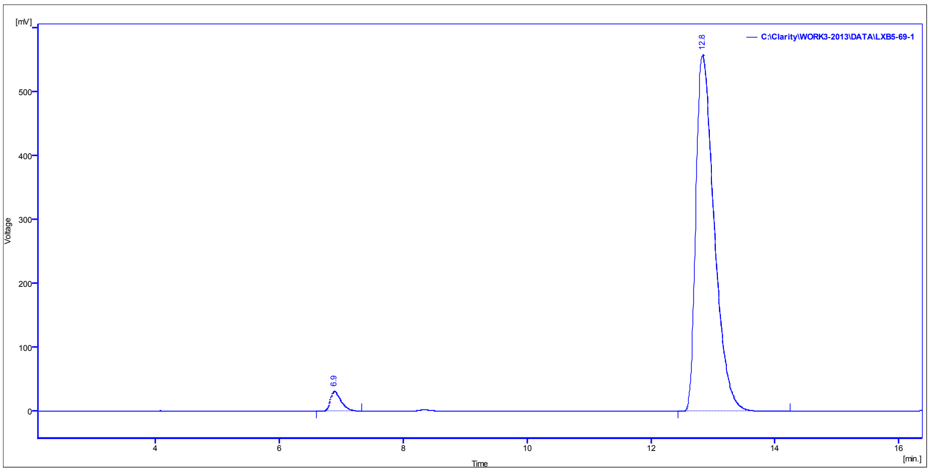

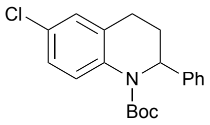

2c

Cellulose–2 column:

	R_t/min	Area/ mV.s	Area/%
1	4.643	7182.967	50.0
2	5.583	7184.082	50.0
	total	14367.049	100.0

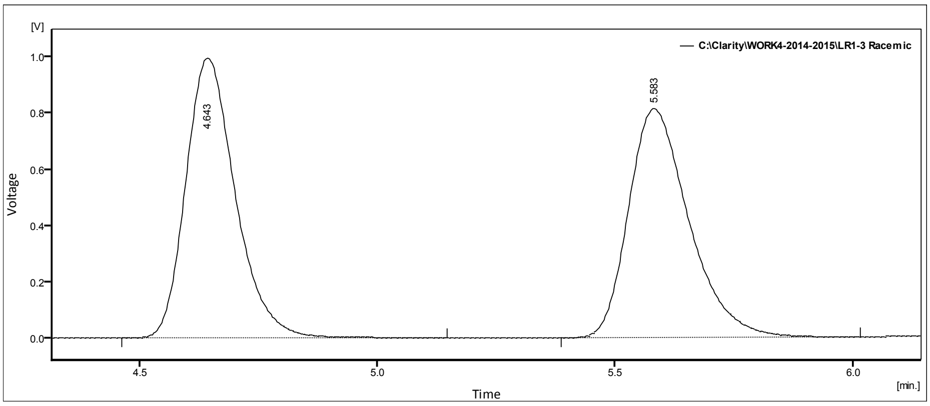

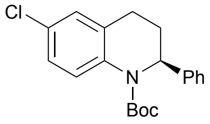

2c (er 92:8)

	R_t/min	Area/ mV.s	Area/%
1	4.557	617.958	8.0
2	5.443	7068.310	92.0
	total	7686.268	100.0

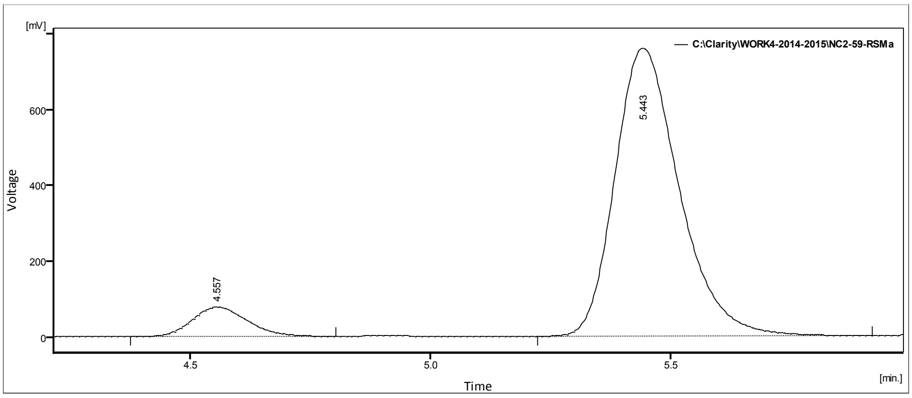

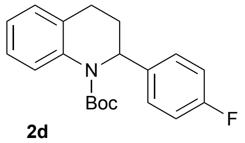

2d

Cellulose–2 column:

	R_t/min	Area/ mV.s	Area/%
1	4.877	1816.571	50.0
2	5.843	1814.579	50.0
	total	3631.150	100.0

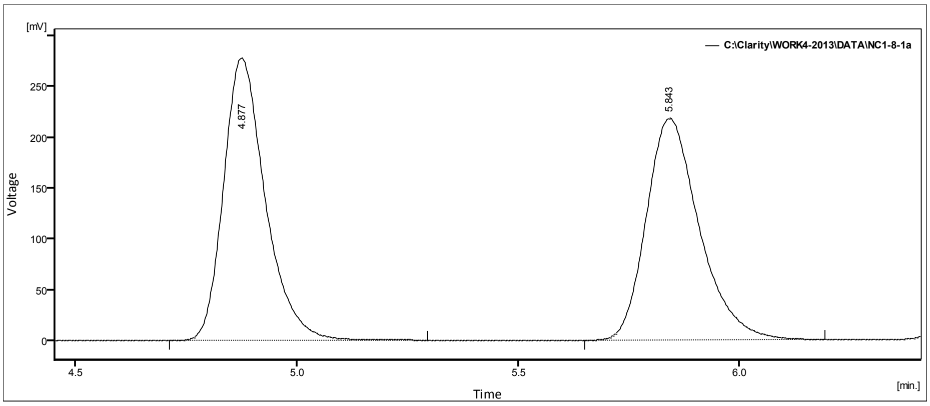

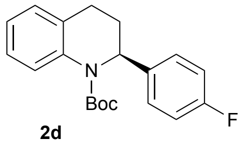

2d (er 94:6)

	R_t/min	Area/ mV.s	Area/%
1	4.987	650.361	5.8
2	6.163	10519.737	94.2
	total	11170.098	100.0

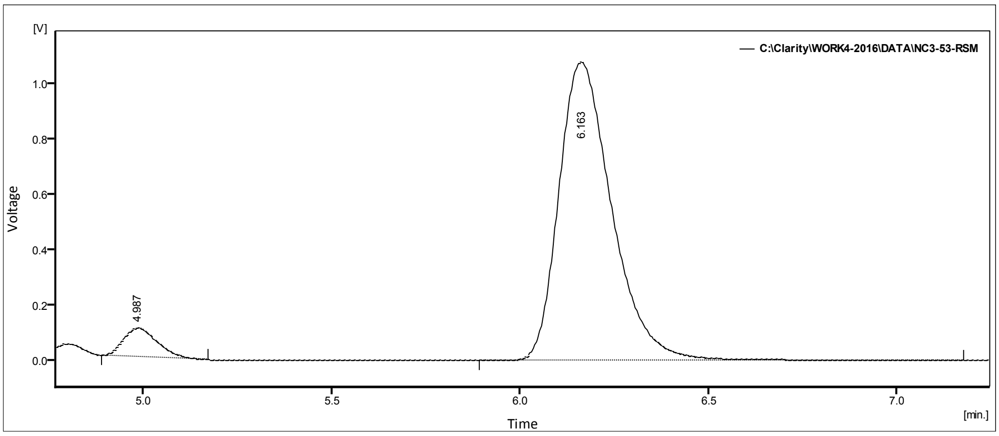

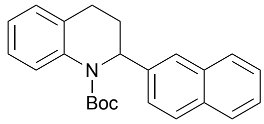

2e

Cellulose–2 column:

	R_t/min	Area/ mV.s	Area/%
1	5.767	3967.360	49.8
2	8.803	3996.123	50.2
	total	7963.483	100.0

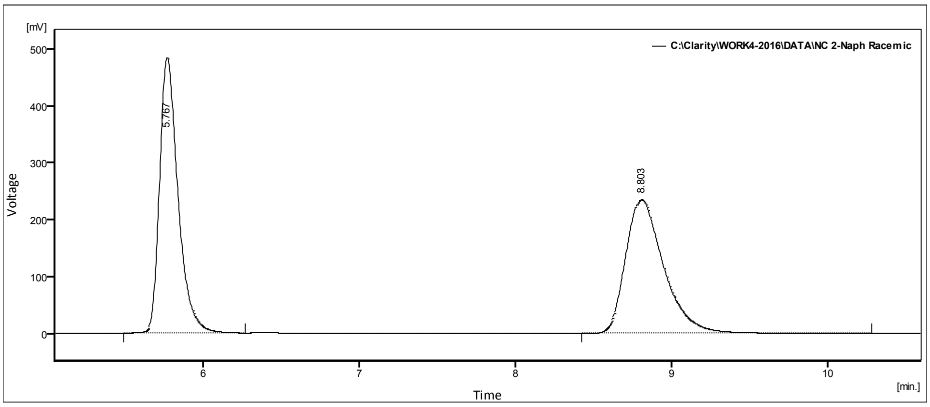

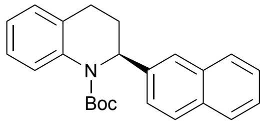

2e (er 96:4)

	R_t/min	Area/ mV.s	Area/%
1	6.083	36.225	4.3
2	9.073	805.644	95.7
	total	841.869	100.0

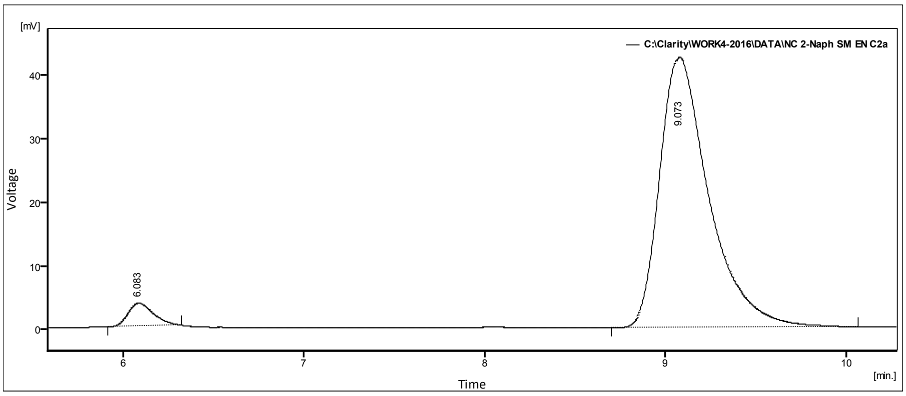

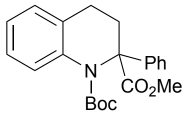

3a

Chiralpak IA column:

	R_t/min	Area/ mV.s	Area/%
1	13.520	1580.772	50.0
2	20.883	1580.230	50.0
	total	3161.002	100.0

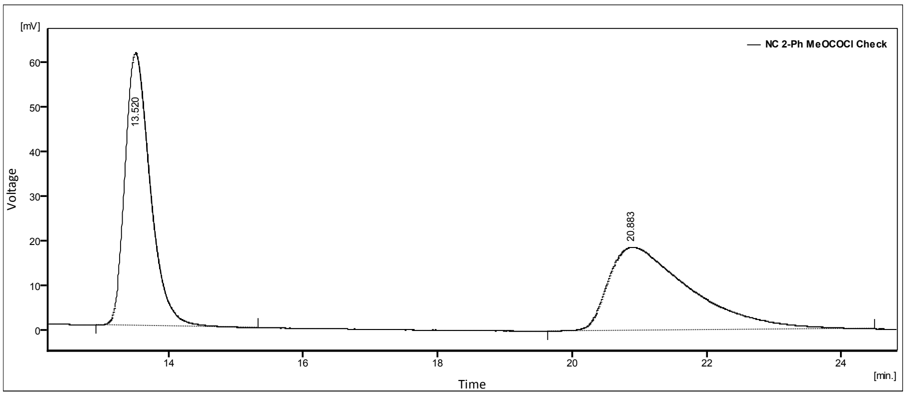

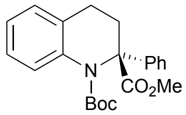

3a (er 93:7)

	R_t/min	Area/ mV.s	Area/%
1	14.400	655.600	7.4
2	22.510	8206.801	92.6
	total	8862.401	100.0

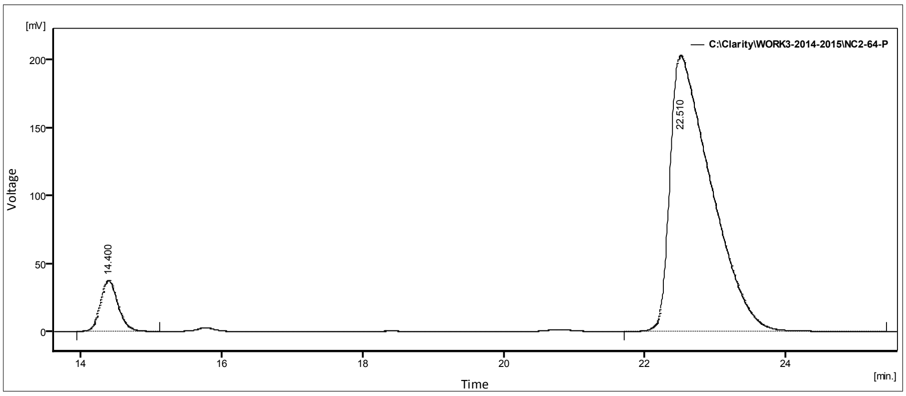

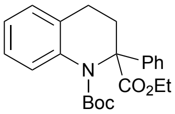

3b

Cellulose–1 column:

	R_t/min	Area/ mV.s	Area/%
1	11.337	4417.325	50.4
2	12.200	4340.606	49.6
	total	8757.931	100.0

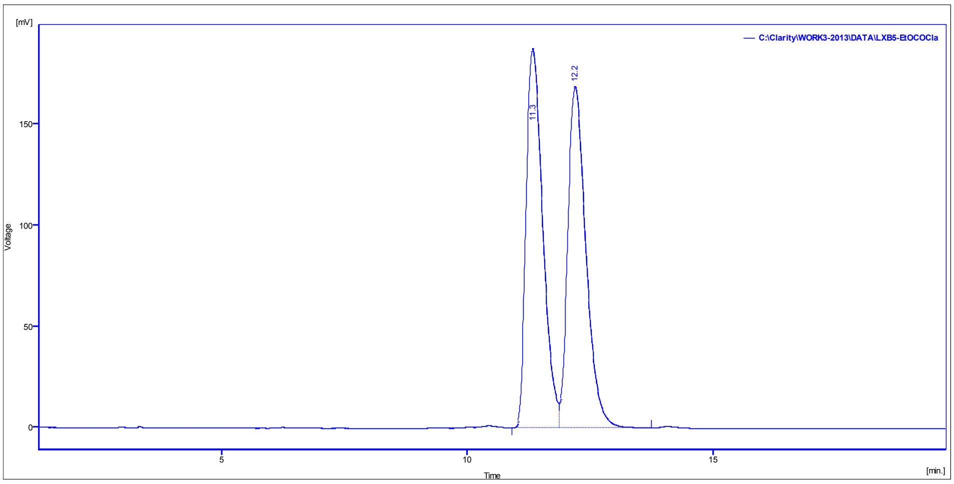

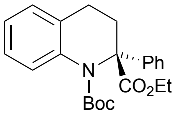

3b (er 97:3)

	R_t/min	Area/ mV.s	Area/%
1	11.773	10375.284	96.6
2	12.850	362.833	3.4
	total	10738.118	100.0

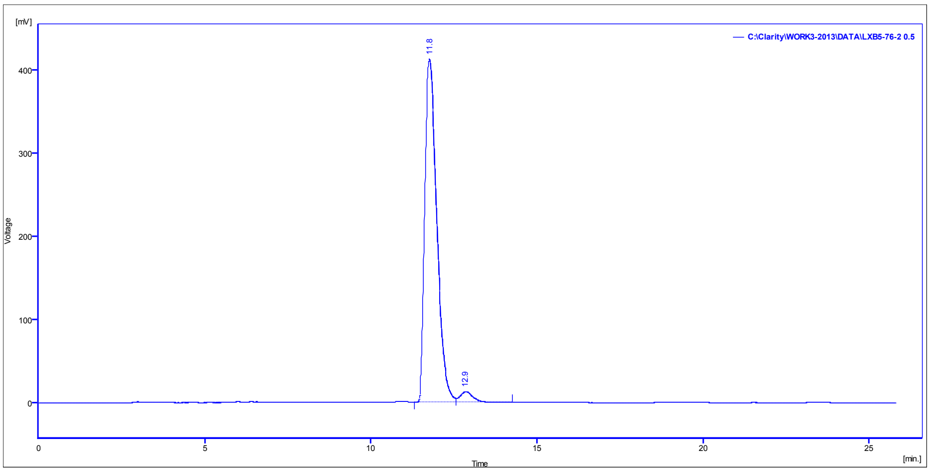

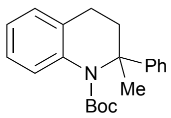

3e

Cellulose–1 column:

	R_t/min	Area/ mV.s	Area/%
1	5.550	6210.511	50.6
2	6.083	6062.368	49.4
	total	12272.879	100.0

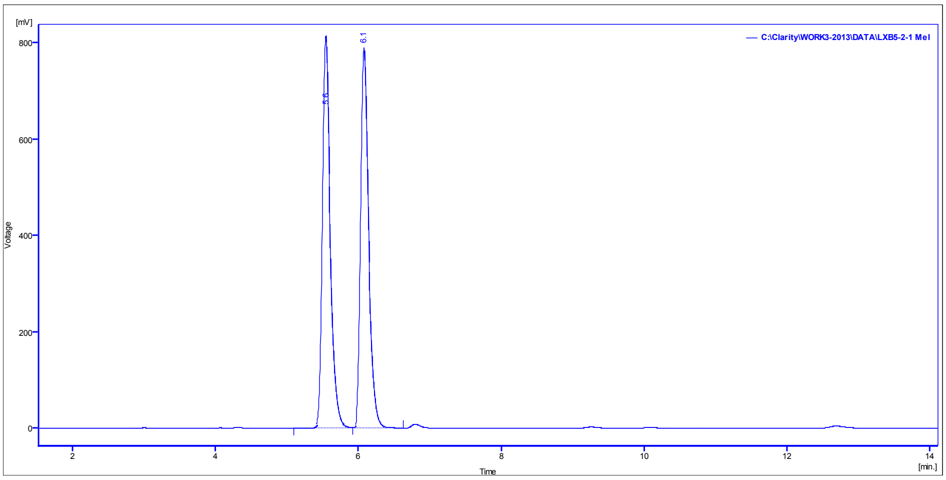

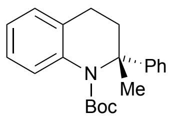

3e (er 94:6)

	R_t/min	Area/ mV.s	Area/%
1	5.600	562.256	6.4
2	6.540	8281.183	93.6
	total	8843.438	100.0

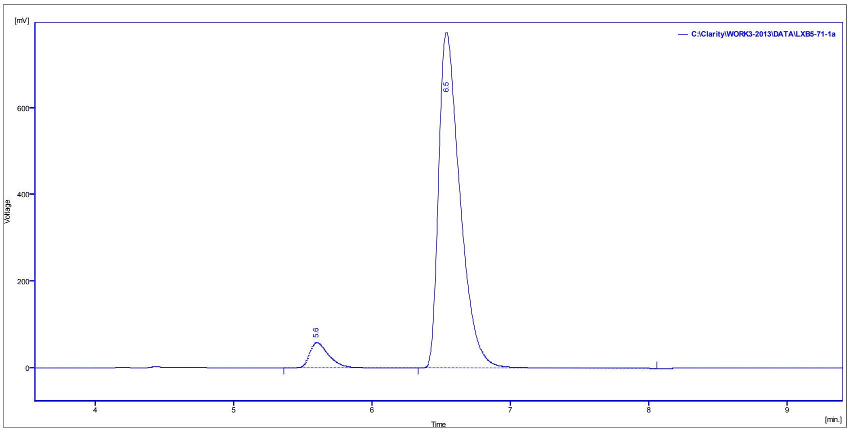

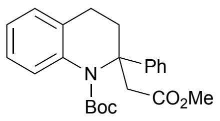

3g

Cellulose–2 column:

	R_t/min	Area/ mV.s	Area/%
1	18.623	2395.425	49.5
2	20.630	2441.602	50.5
	total	4837.026	100.0

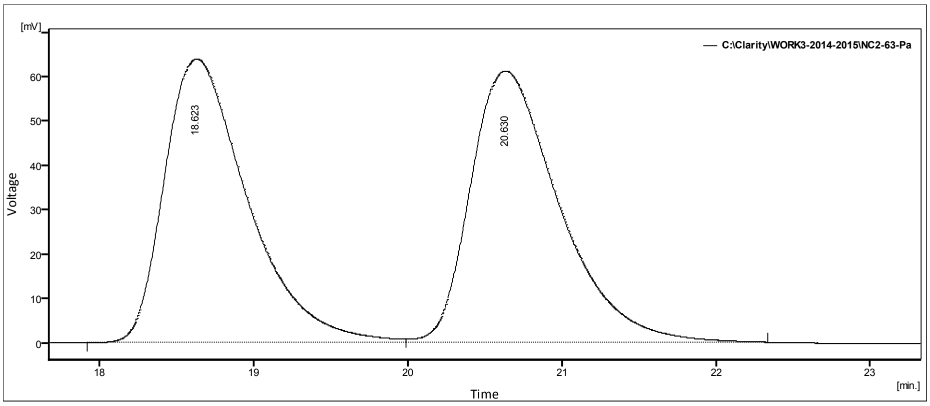

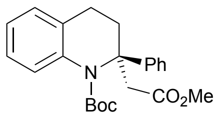

3g (er 92:8)

	R_t/min	Area/ mV.s	Area/%
1	18.053	2011.905	91.7
2	20.107	182.430	8.3
	total	2194.335	100.0

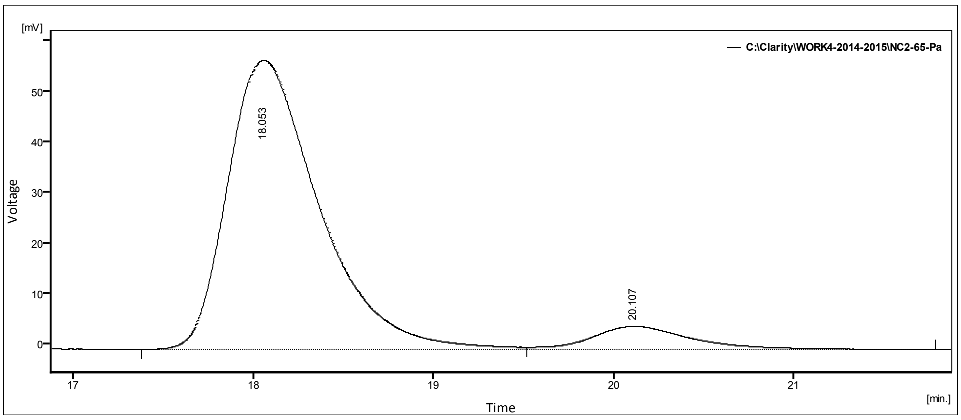

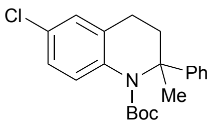

8

Chiralpak IA column:

	R_t/min	Area/ mV.s	Area/%
1	7.220	4517.211	50.0
2	9.070	4517.369	50.0
	total	9034.579	100.0

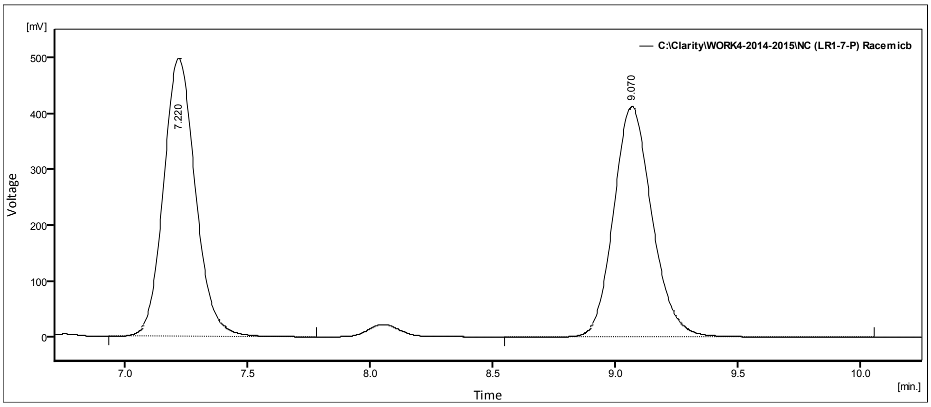

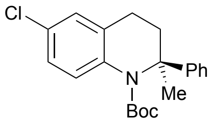

8 (er 87:13)

	R_t/min	Area/ mV.s	Area/%
1	7.220	4517.211	50.0
2	9.070	4517.369	50.0
	total	9034.579	100.0

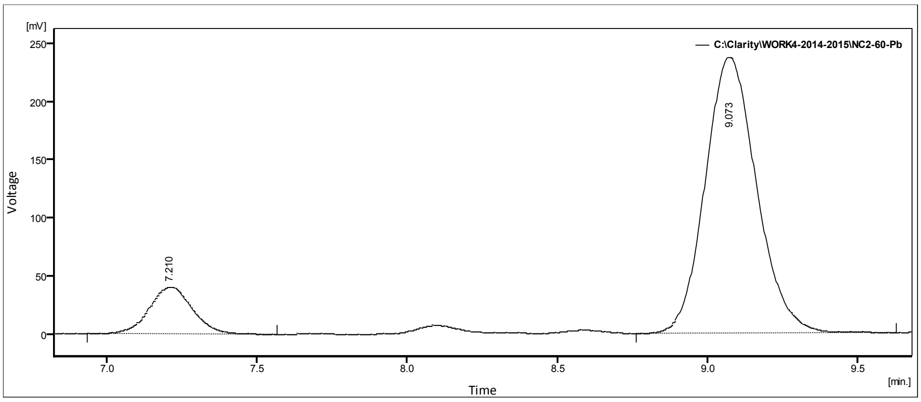

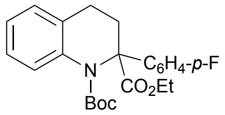

9

Cellulose-1 column:

	R_t/min	Area/ mV.s	Area/%
1	9.460	3773.802	50.0
2	11.073	3778.984	50.0
	total	7552.786	100.0

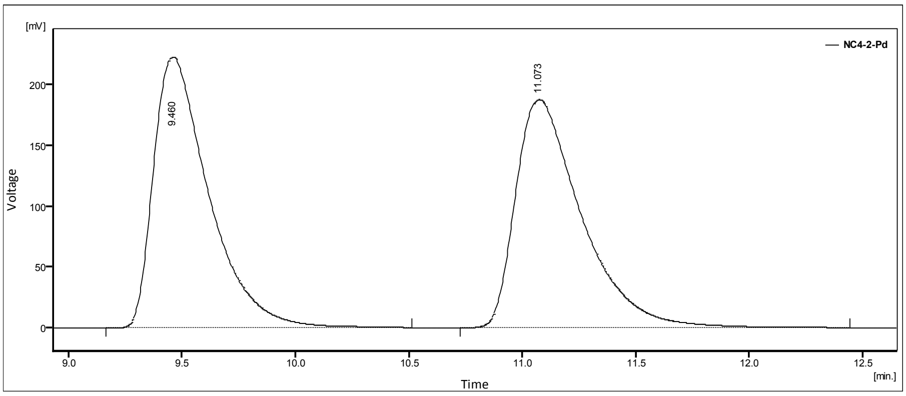

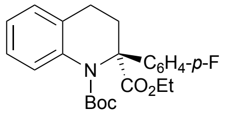

9 (er 93.5:6.5)

	R_t/min	Area/ mV.s	Area/%
1	9.443	164.690	6.6
2	11.017	2330.459	93.4
	total	2495.150	100.0

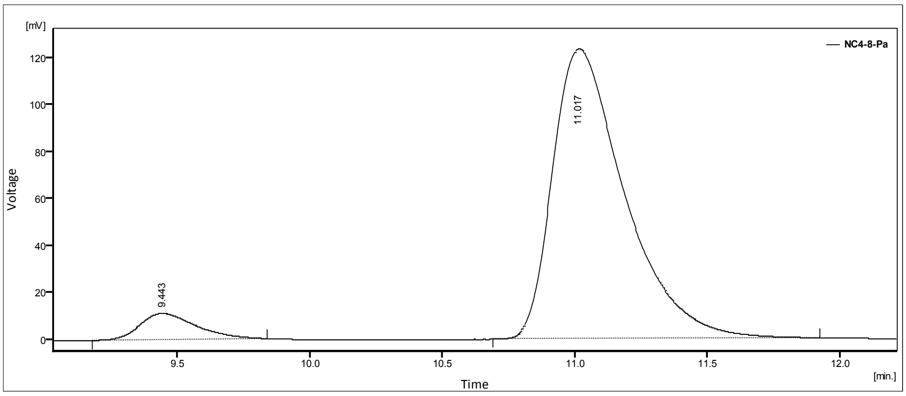

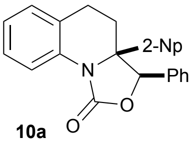

10a (*cis*)

Daicel ChiralPak IA column:

	R_t/min	Area/ mV.s	Area/%
1	32.517	4074.749	50.0
2	49.890	4073.760	50.0
	total	8148.509	100.0

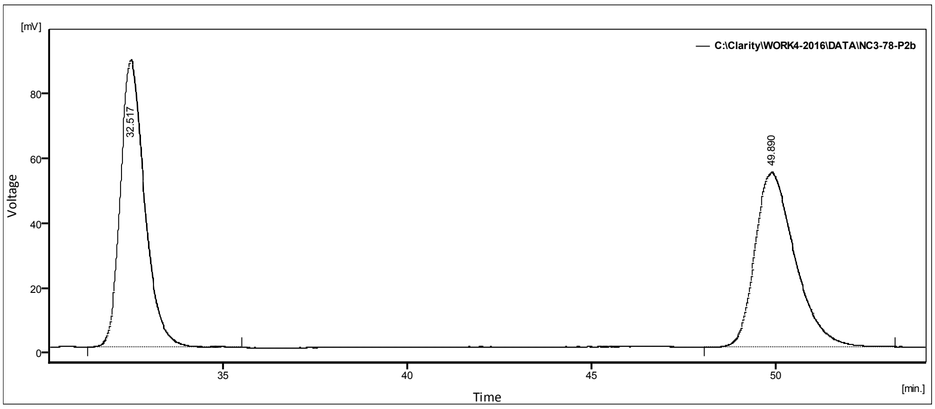

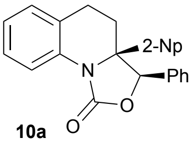

10a (er 95.5:4.5)

	R_t/min	Area/ mV.s	Area/%
1	35.630	10875.569	95.4
2	54.043	529.066	4.6
	total	11404.635	100.0

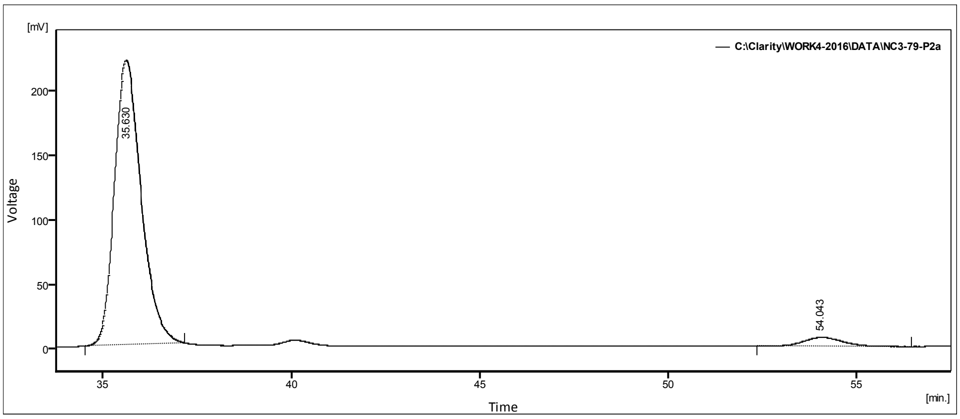

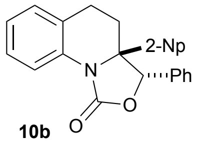

10b (*trans*)

Daicel ChiralPak IA column:

	R_t/min	Area/ mV.s	Area/%
1	37.390	3203.117	50.0
2	95.793	3199.154	50.0
	total	6402.271	100.0

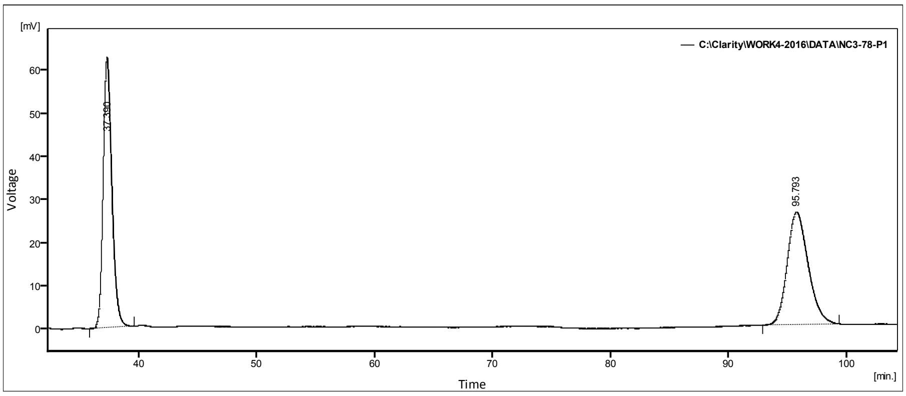

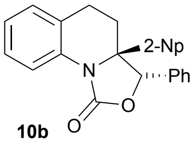

10b (er 95.5:4.5)

	R_t/min	Area/ mV.s	Area/%
1	38.710	4307.014	95.6
2	99.580	196.538	4.4
	total	4503.553	100.0

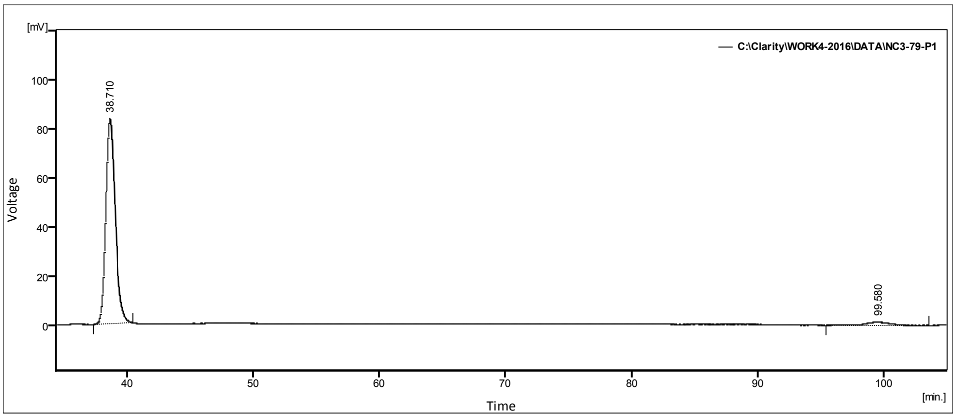

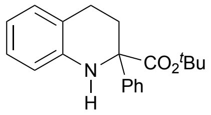

11

Daicel ChiralPak IA column:

	R_t/min	Area/ mV.s	Area/%
1	10.250	3740.048	50.1
2	12.157	3726.581	49.9
	total	7466.628	100.0

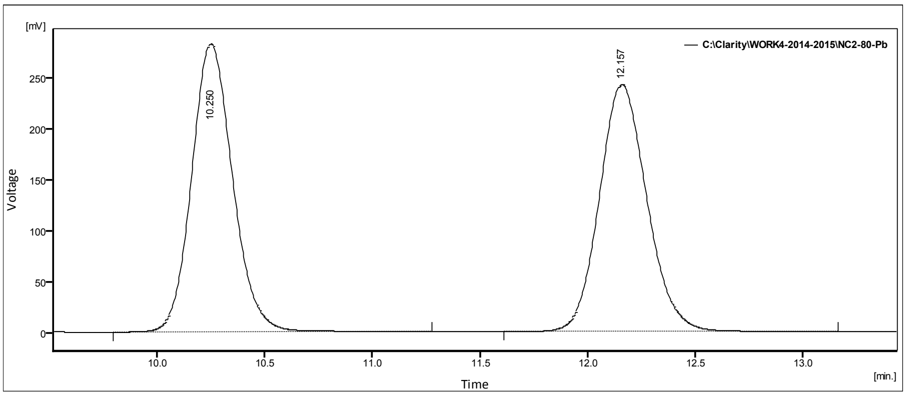

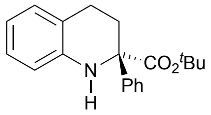

11 (er 98:2)

	R_t/min	Area/ mV.s	Area/%
1	10.237	592.441	1.7
2	11.833	34053.421	98.3
	total	34645.862	100.0

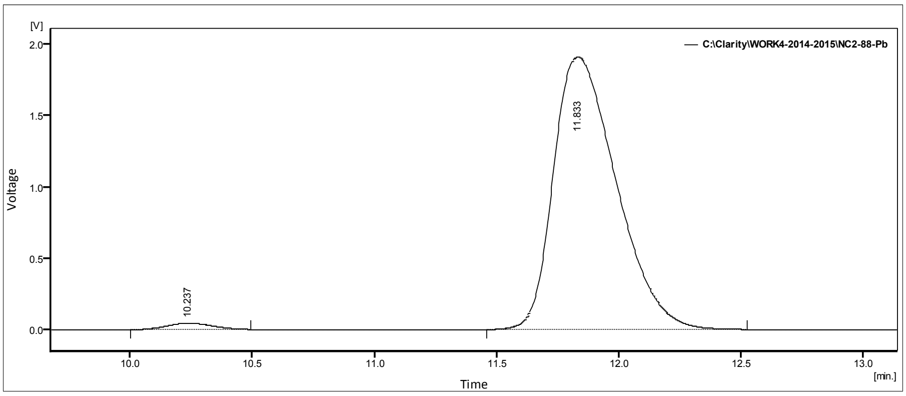

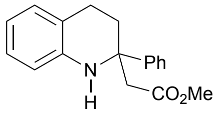

12

Daicel ChiralCel OJ column:

	R_t/min	Area/ mV.s	Area/%
1	24.810	8661.403	50.0
2	36.357	8669.377	50.0
	total	17330.780	100.0

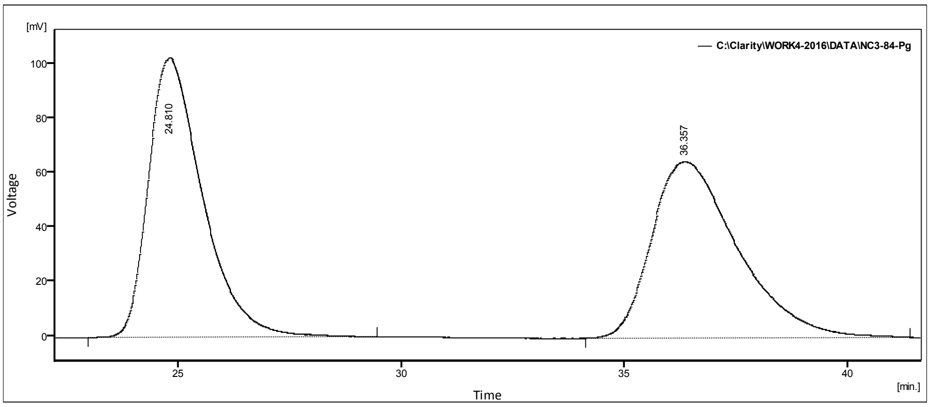

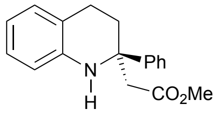

12 (er 88:12)

	R_t/min	Area/ mV.s	Area/%
1	24.587	11195.814	88.3
2	36.810	1486.358	11.7
	total	12682.171	100.0

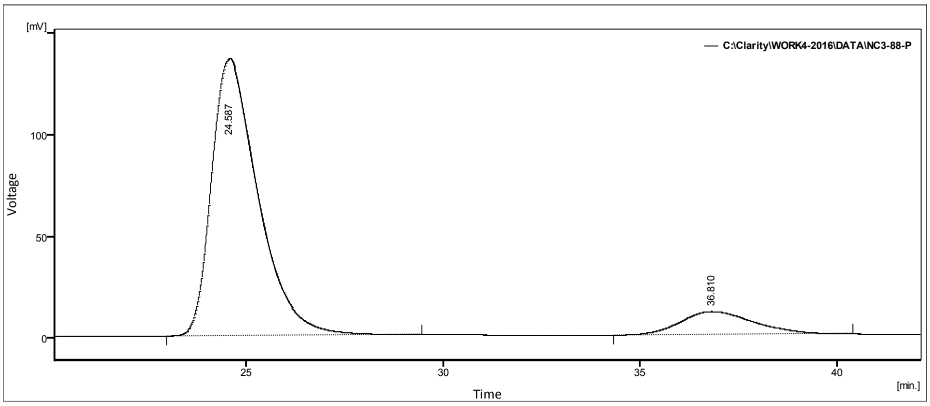

9. ^{1}H and ^{13}C NMR spectra

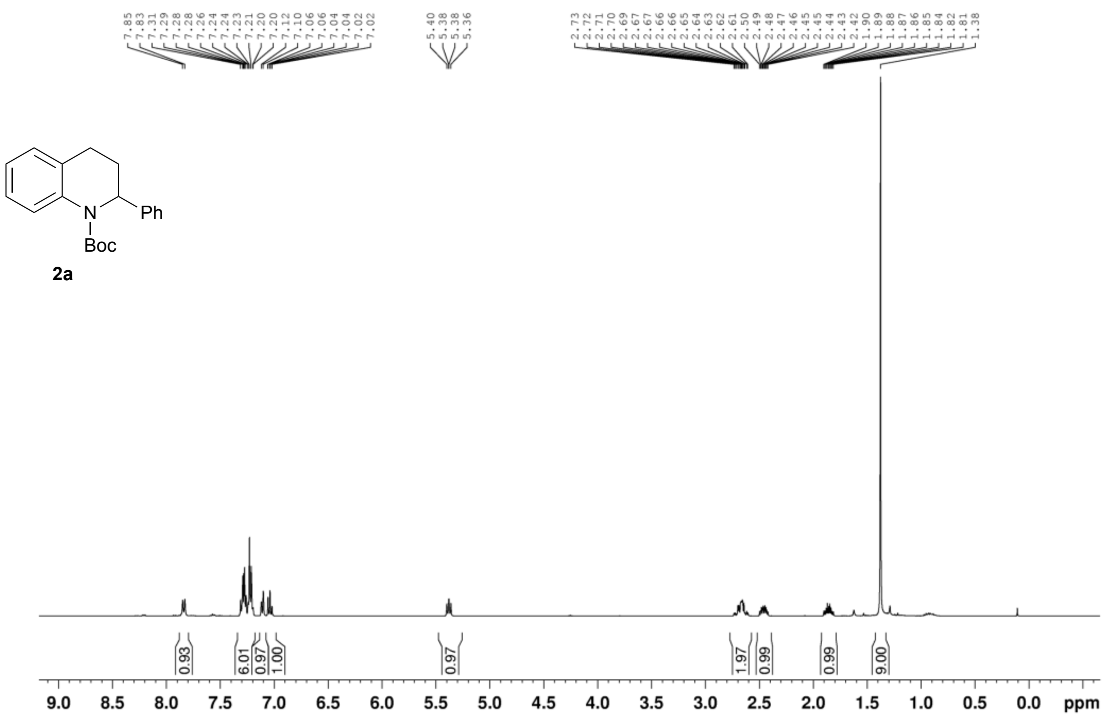

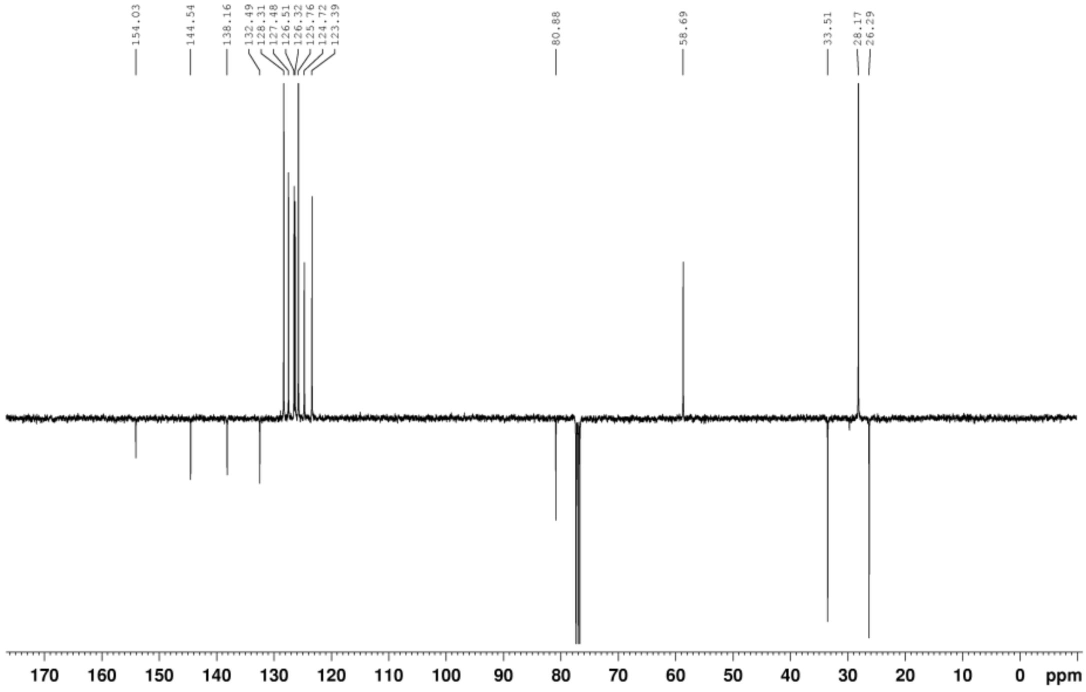

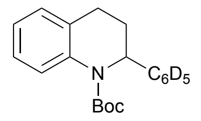
N
C6D5
Boc

2b

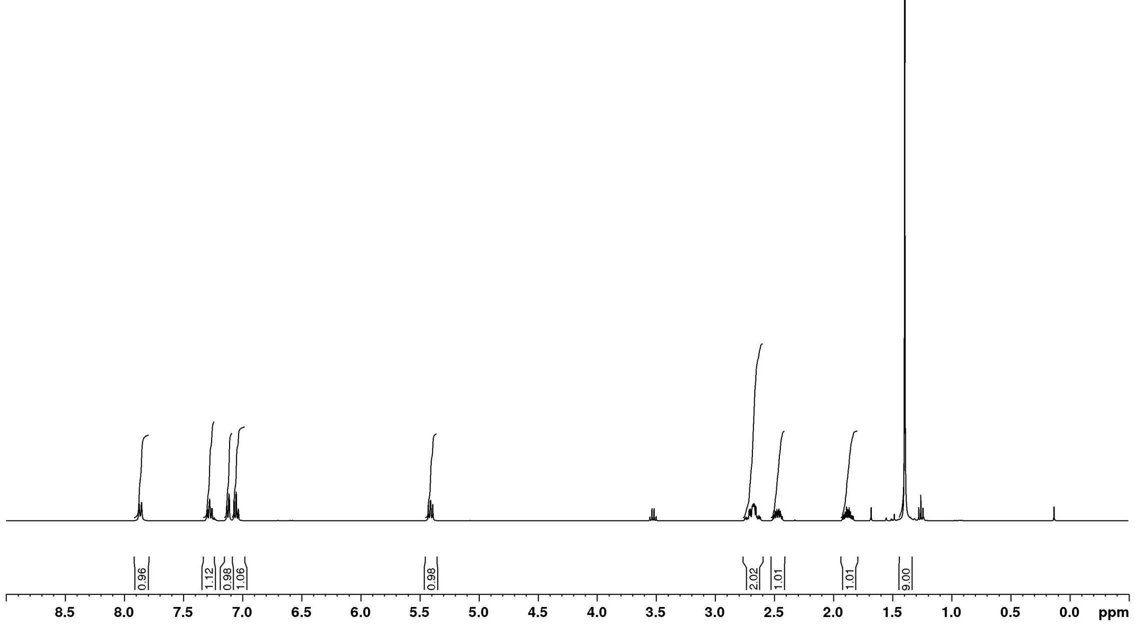
0.96
1.12
0.98
1.06
0.98
2.02
1.01
1.01
9.00
8.5 8.0 7.5 7.0 6.5 6.0 5.5 5.0 4.5 4.0 3.5 3.0 2.5 2.0 1.5 1.0 0.5 0.0 ppm

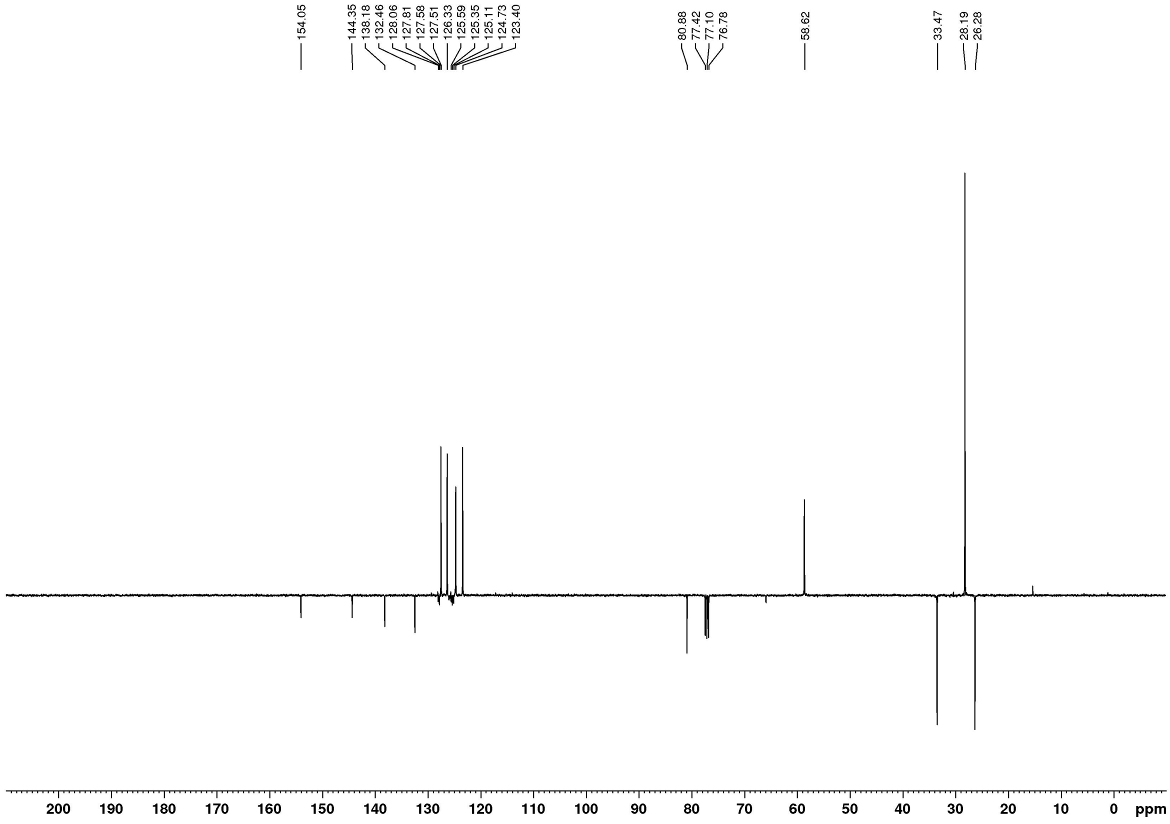
154.05
144.35
138.18
132.46
128.06
127.81
127.58
127.51
126.33
125.59
125.35
125.11
124.73
123.40
80.88
77.42
77.10
76.78
58.62
33.47
28.19
26.28
200 190 180 170 160 150 140 130 120 110 100 90 80 70 60 50 40 30 20 10 0 ppm

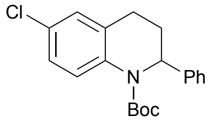
Cl
N
Ph
Boc

2c

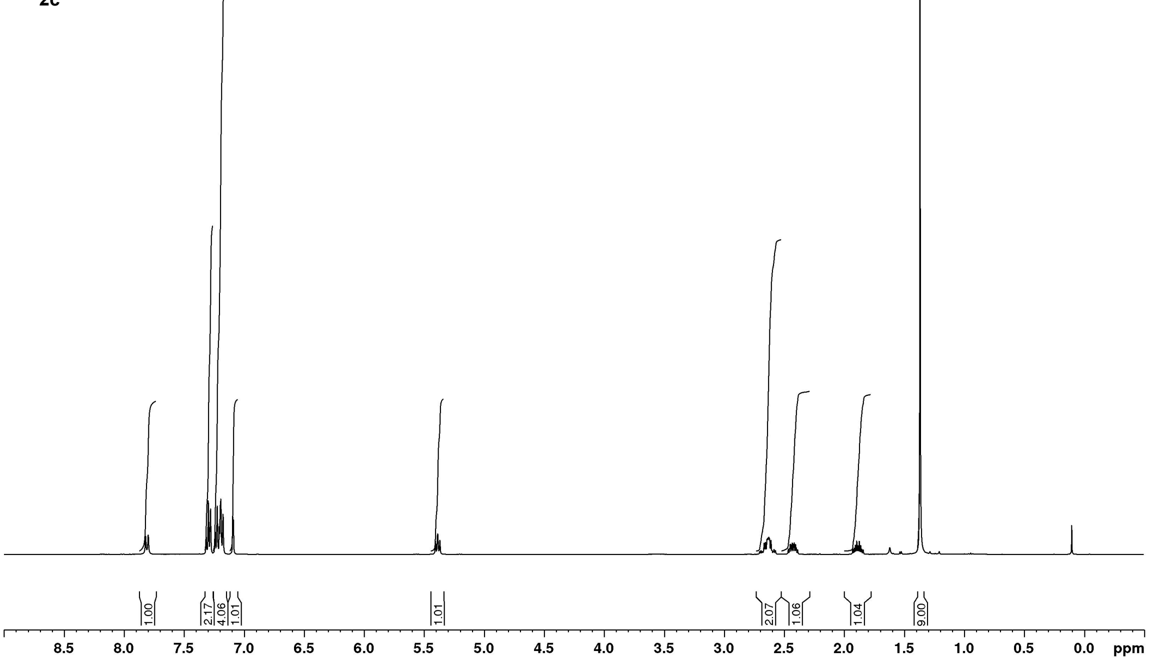
1.00
2.17
4.06
1.01
1.01
2.07
1.06
1.04
9.00
8.5
8.0
7.5
7.0
6.5
6.0
5.5
5.0
4.5
4.0
3.5
3.0
2.5
2.0
1.5
1.0
0.5
0.0
ppm

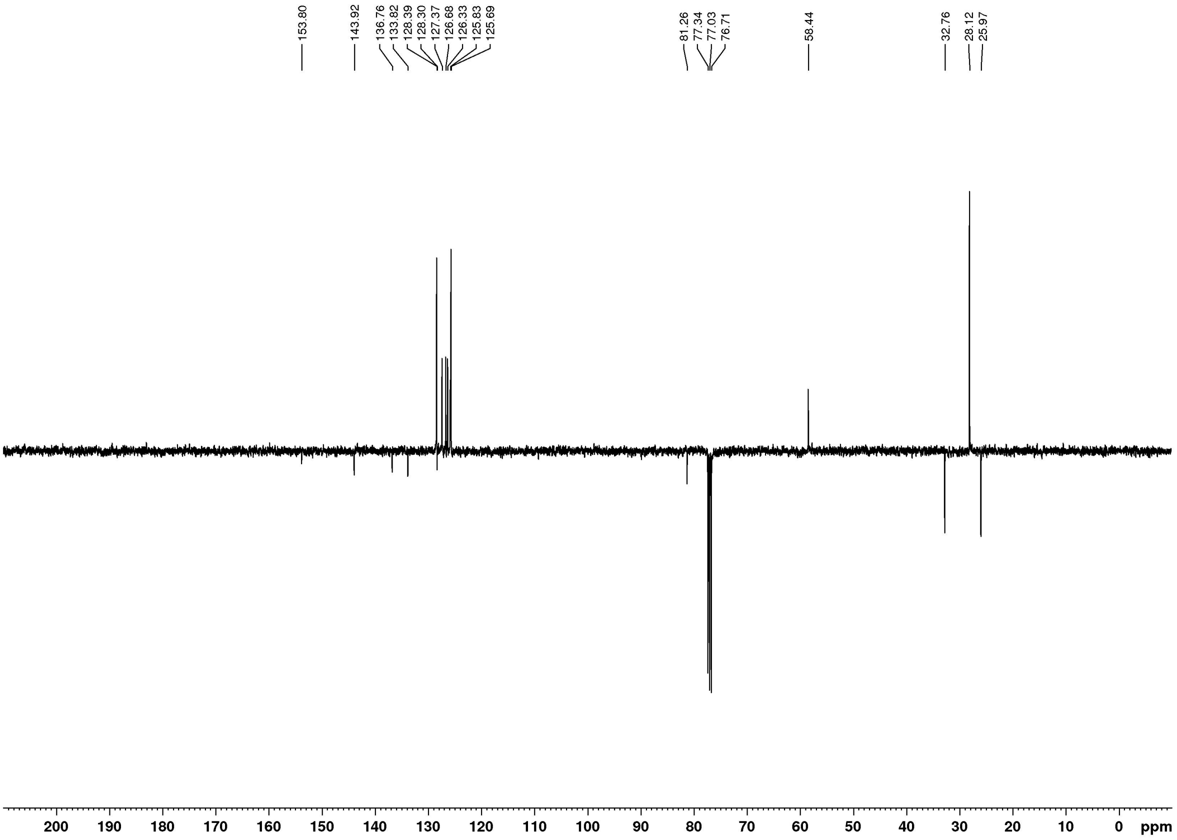
153.80
143.92
136.76
133.82
128.39
128.30
127.37
126.68
126.33
125.83
125.69
81.26
77.34
77.03
76.71
58.44
32.76
28.12
25.97
200
190
180
170
160
150
140
130
120
110
100
90
80
70
60
50
40
30
20
10
0
ppm

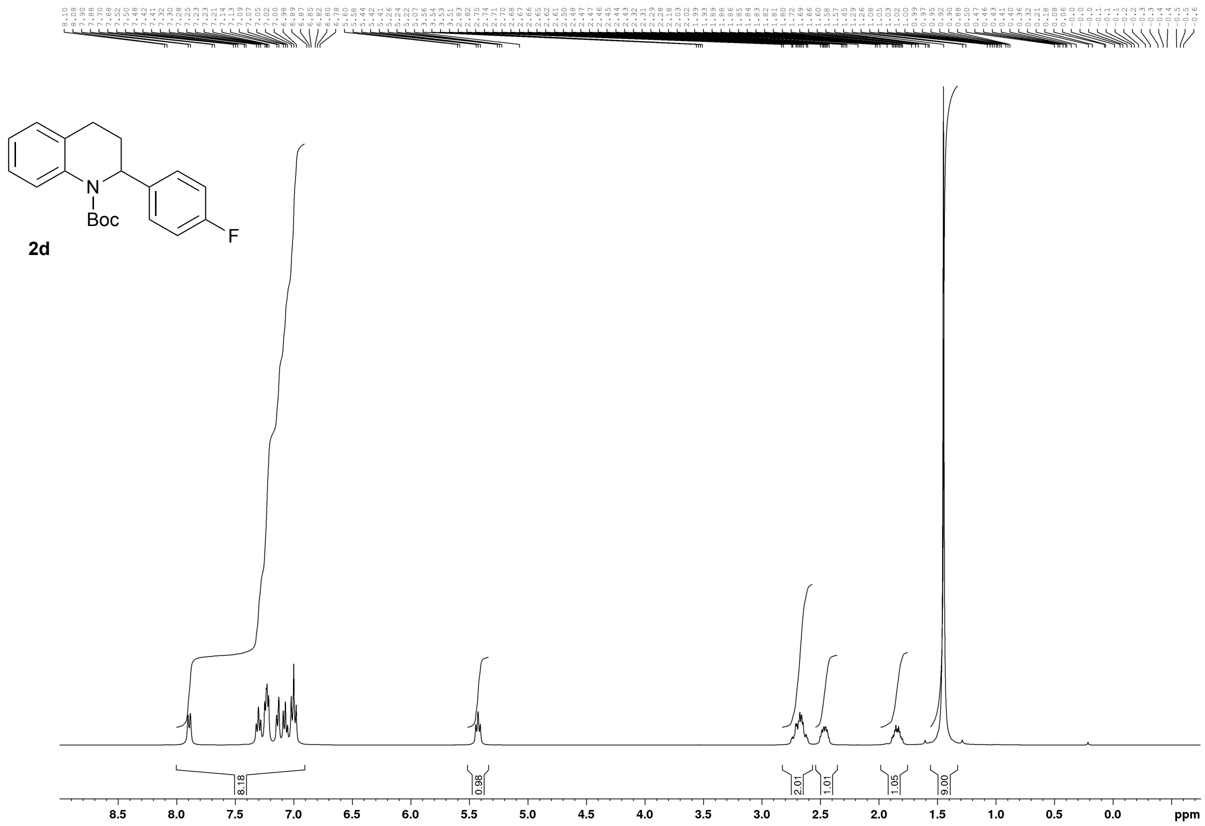
N
Boc
F
8.18
0.98
2.01
1.01
1.05
9.00
8.5 8.0 7.5 7.0 6.5 6.0 5.5 5.0 4.5 4.0 3.5 3.0 2.5 2.0 1.5 1.0 0.5 0.0 ppm

2d

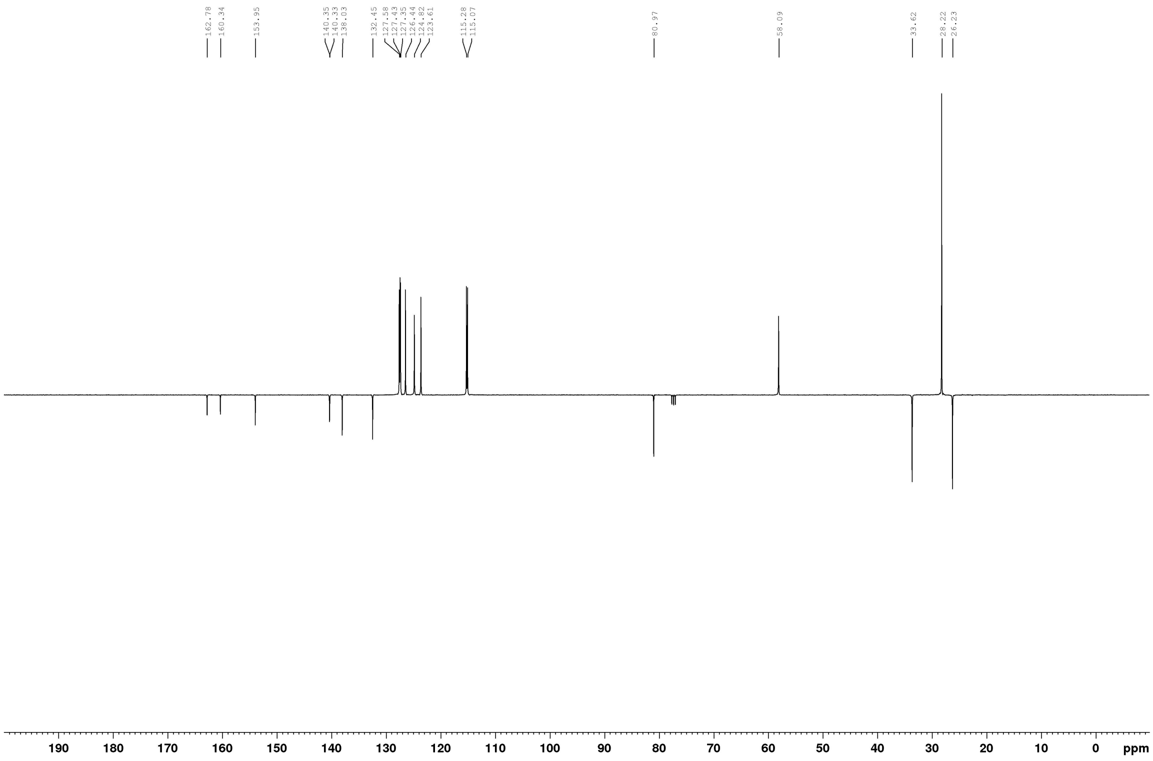
162.78
160.34
153.95
140.35
140.33
138.03
132.45
127.58
127.43
127.35
126.44
124.82
123.61
115.28
115.07
80.97
58.09
33.62
28.22
26.23
190 180 170 160 150 140 130 120 110 100 90 80 70 60 50 40 30 20 10 0 ppm

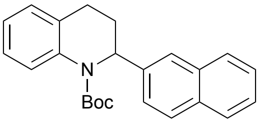
N
Boc

2e

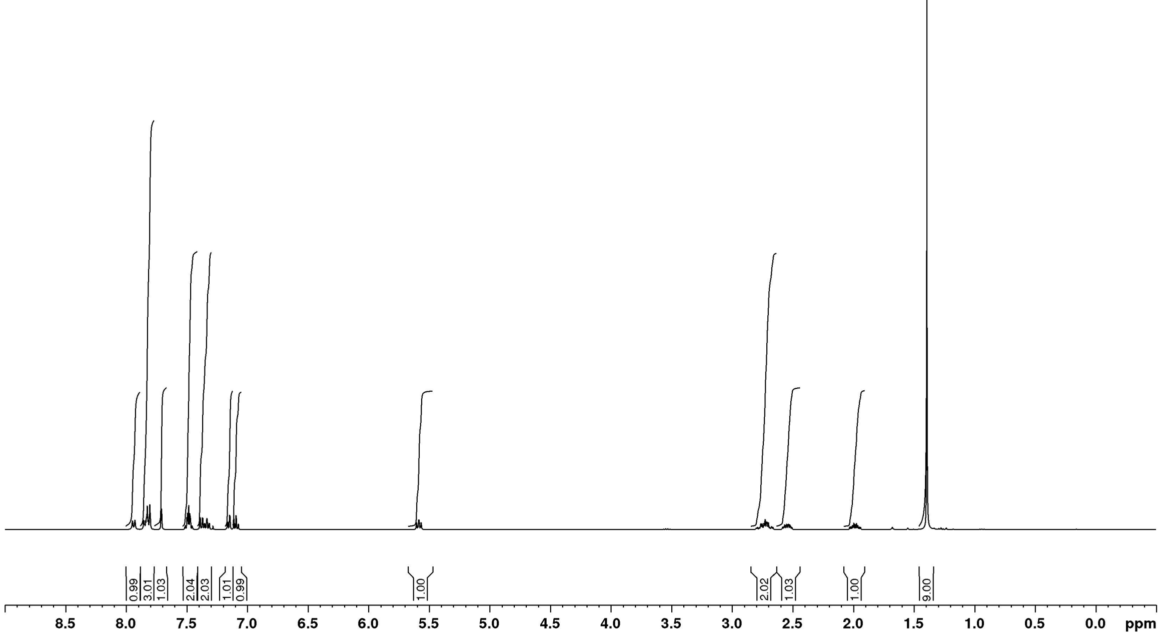
0.99
3.01
1.03
2.04
2.03
1.01
0.99
1.00
2.02
1.03
1.00
9.00
8.5 8.0 7.5 7.0 6.5 6.0 5.5 5.0 4.5 4.0 3.5 3.0 2.5 2.0 1.5 1.0 0.5 0.0 ppm

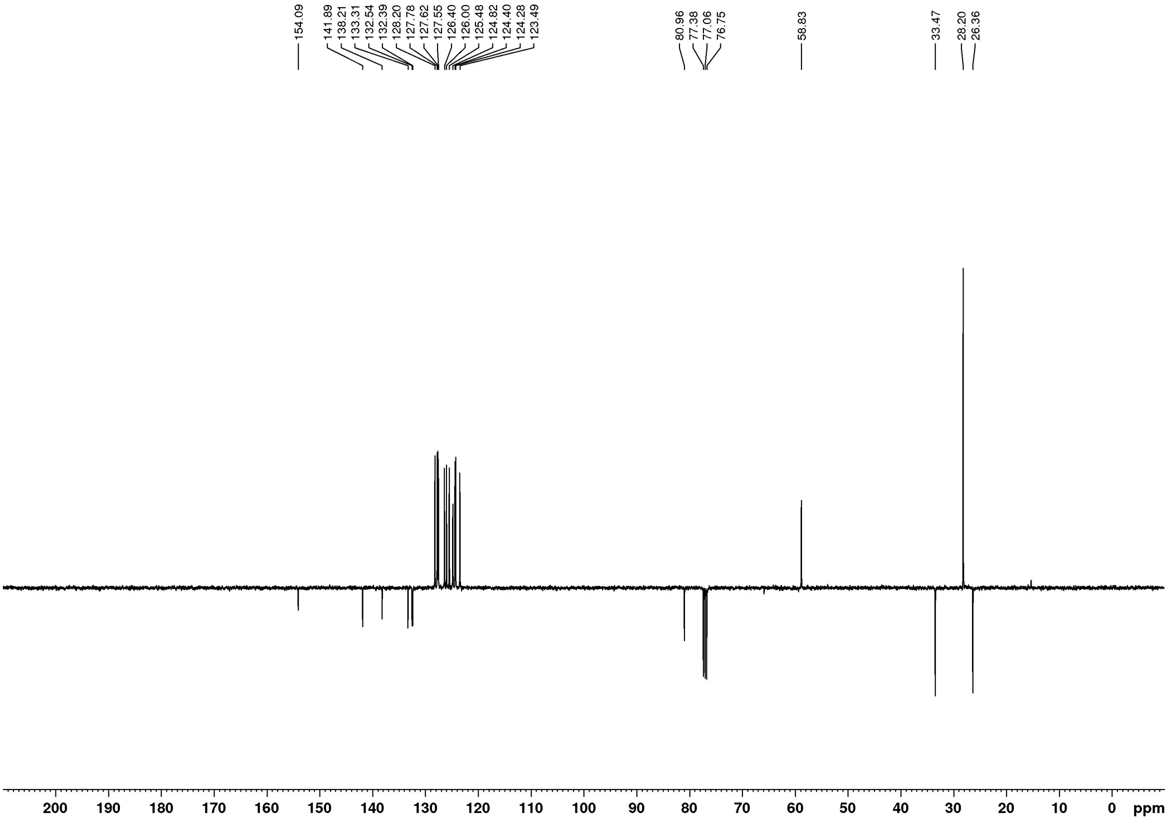
154.09
141.89
138.21
133.31
132.54
132.39
128.20
127.78
127.62
127.55
126.40
126.00
125.48
124.82
124.40
124.28
123.49
80.96
77.38
77.06
76.75
58.83
33.47
28.20
26.36
200 190 180 170 160 150 140 130 120 110 100 90 80 70 60 50 40 30 20 10 0 ppm

3a

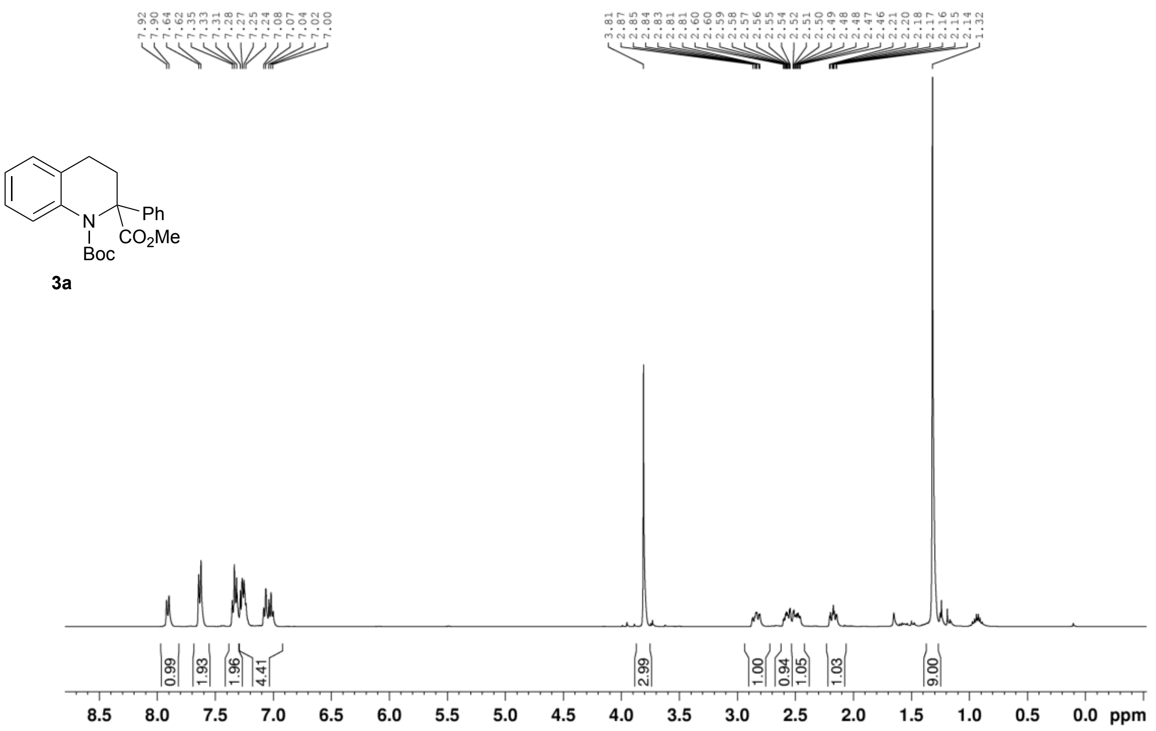

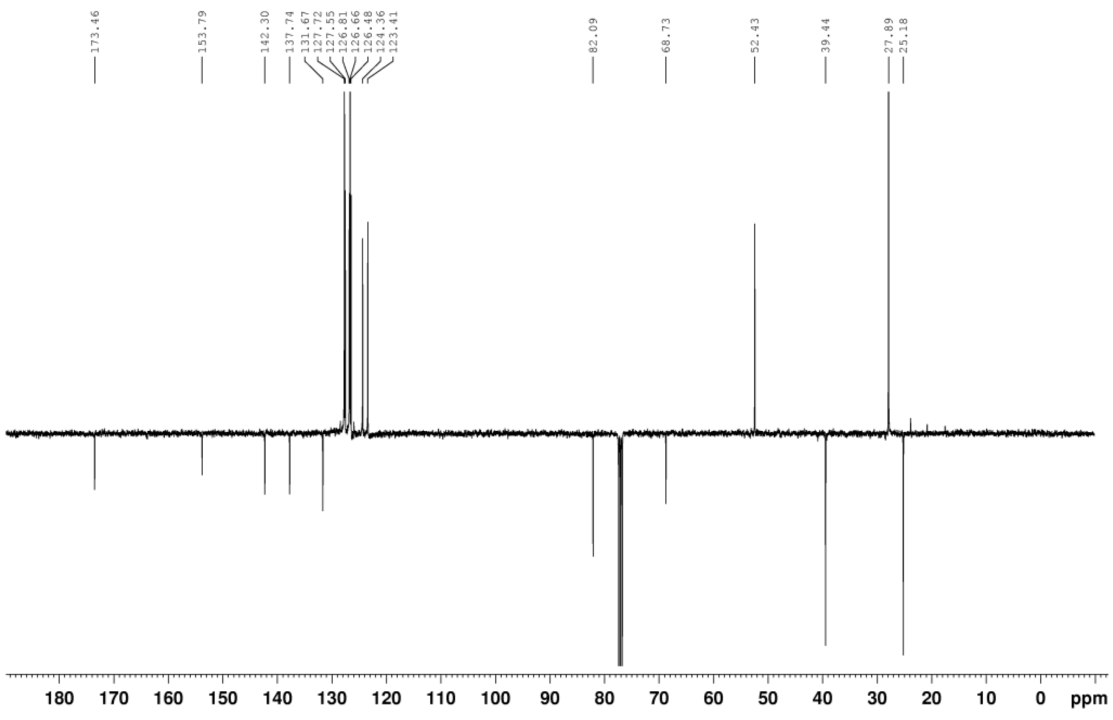

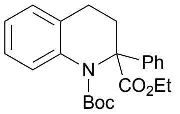
Ph
N
CO2Et
Boc

3b

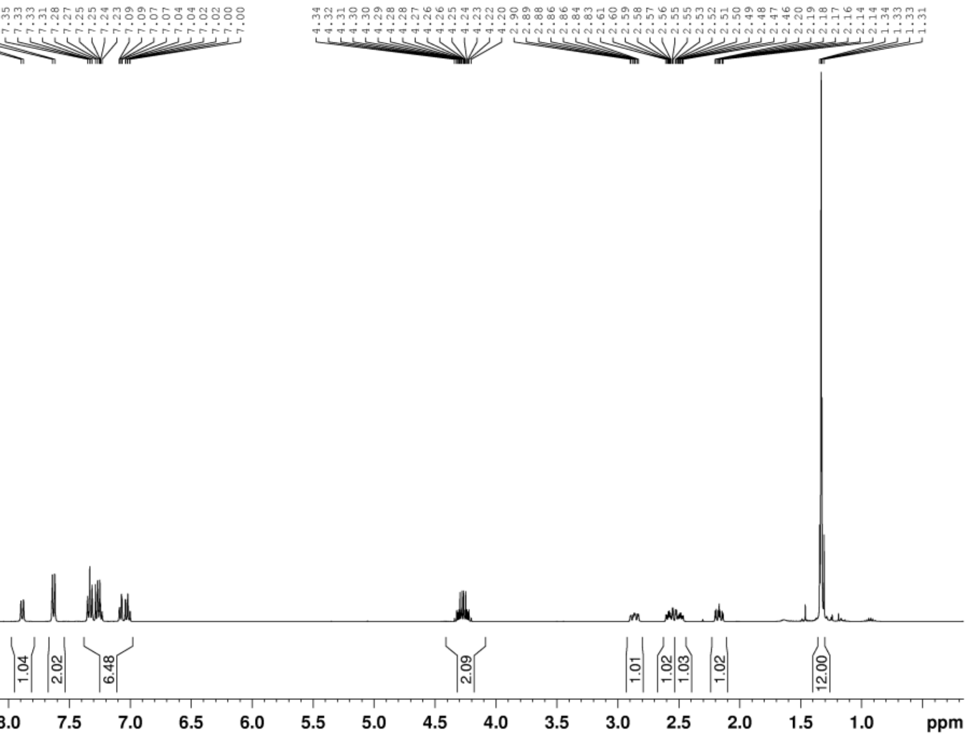
7.89 7.87 7.64 7.62 7.35 7.33 7.33 7.31 7.28 7.27 7.25 7.25 7.24 7.23 7.09 7.09 7.07 7.07 7.04 7.04 7.02 7.02 7.00 7.00
4.34 4.32 4.31 4.30 4.29 4.28 4.28 4.27 4.26 4.25 4.24 4.23 4.22 4.20
2.90 2.89 2.88 2.86 2.84 2.83 2.61 2.60 2.59 2.58 2.57 2.56 2.55 2.55 2.53 2.52 2.51 2.50 2.49 2.48 2.47 2.46 2.20 2.19 2.18 2.17 2.16 2.14 2.14 1.34 1.33 1.33 1.31
1.04
2.02
6.48
2.09
1.01
1.02
1.03
1.02
12.00
8.5 8.0 7.5 7.0 6.5 6.0 5.5 5.0 4.5 4.0 3.5 3.0 2.5 2.0 1.5 1.0 ppm

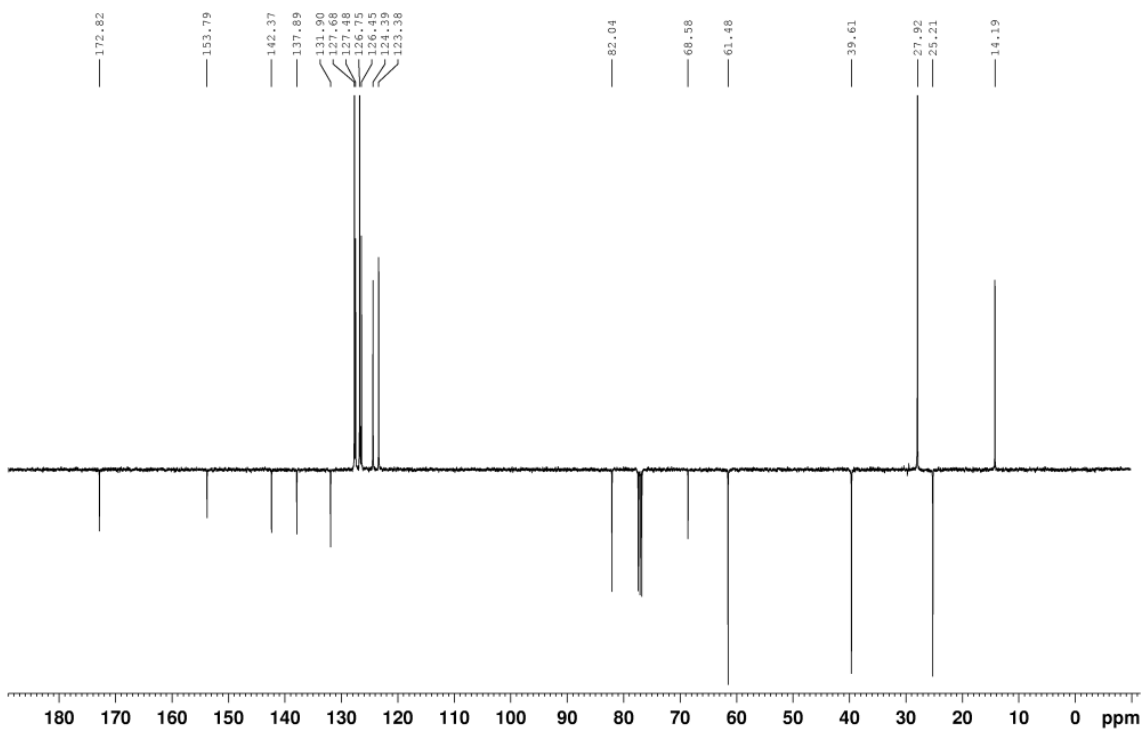
172.82
153.79
142.37
137.89
131.90
127.68
127.48
126.75
126.45
124.39
123.38
82.04
68.58
61.48
39.61
27.92
25.21
14.19
180 170 160 150 140 130 120 110 100 90 80 70 60 50 40 30 20 10 0 ppm

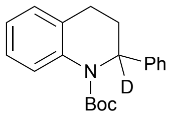
N
Ph
D
Boc

3c

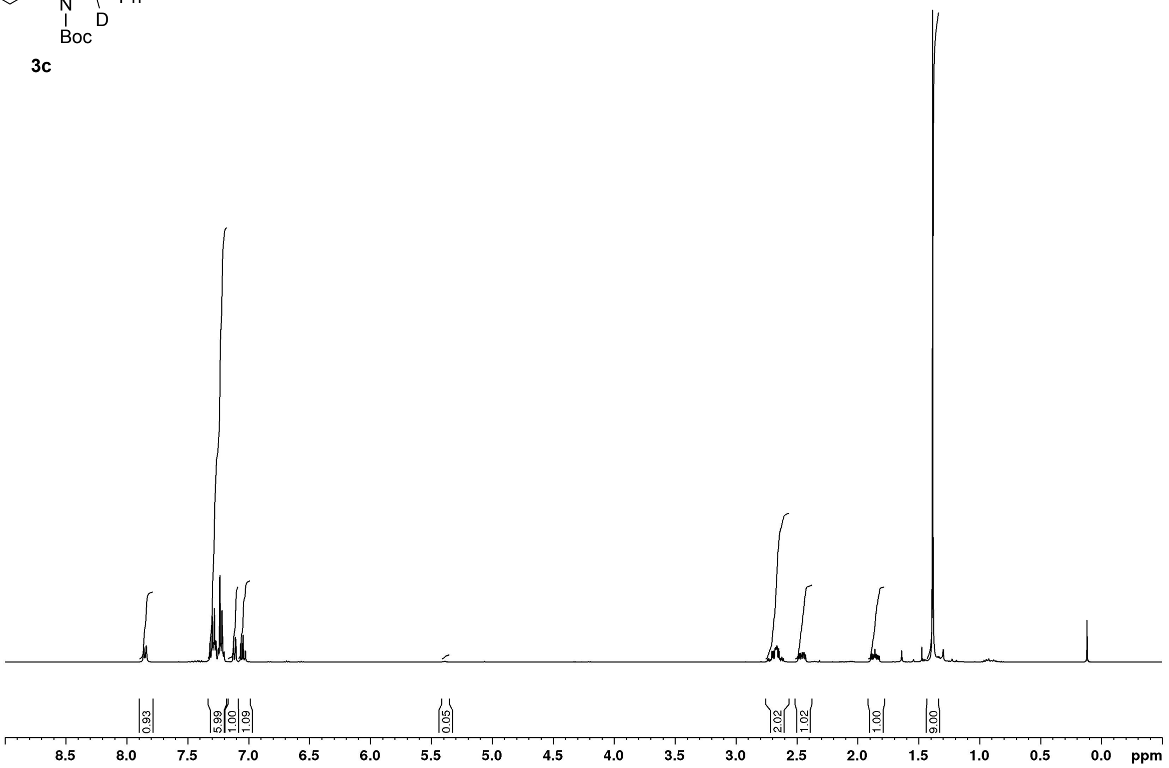
0.93
5.99
1.00
1.09
0.05
2.02
1.02
1.00
9.00
8.5 8.0 7.5 7.0 6.5 6.0 5.5 5.0 4.5 4.0 3.5 3.0 2.5 2.0 1.5 1.0 0.5 0.0 ppm

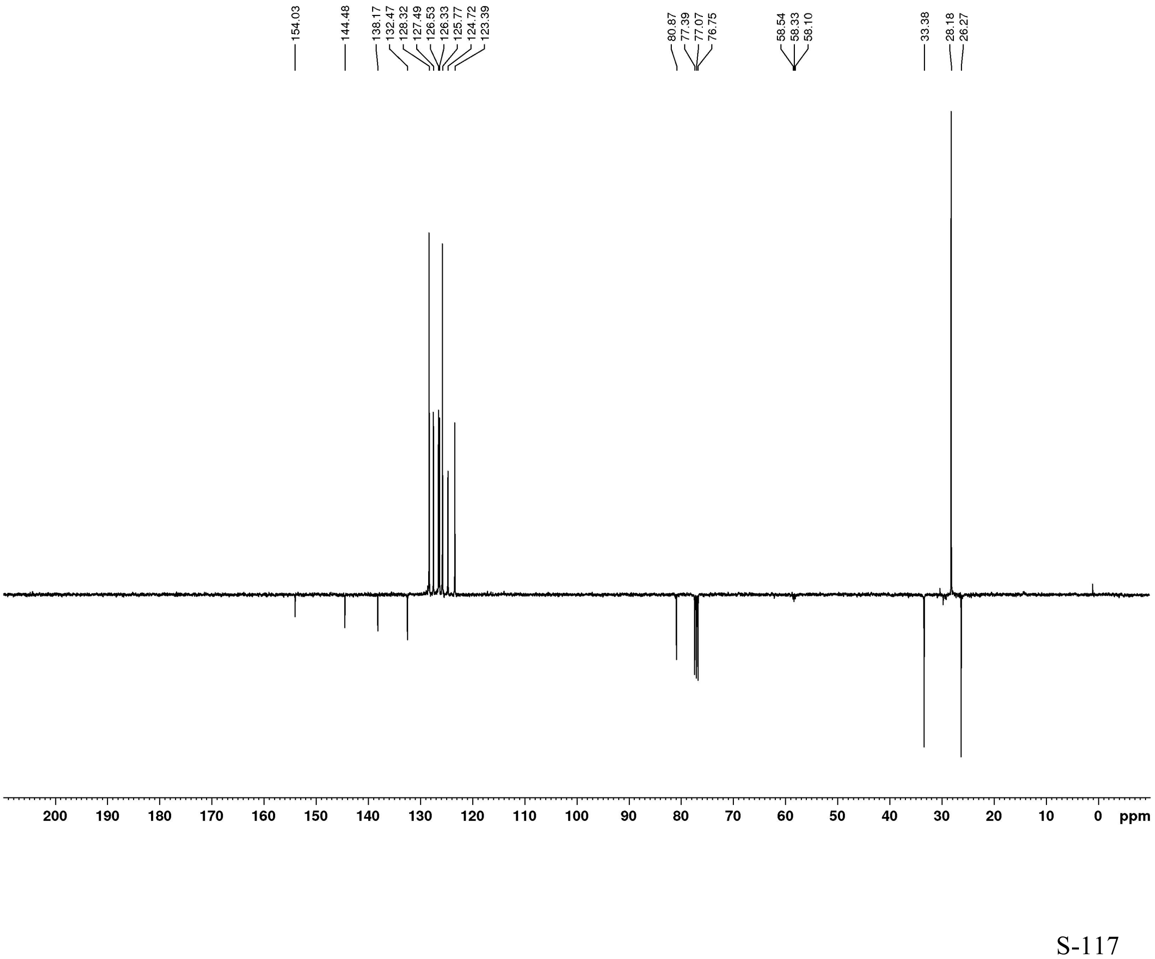
154.03
144.48
138.17
132.47
128.32
127.49
126.53
126.33
125.77
124.72
123.39
80.87
77.39
77.07
76.75
58.54
58.33
58.10
33.38
28.18
26.27
200 190 180 170 160 150 140 130 120 110 100 90 80 70 60 50 40 30 20 10 0 ppm

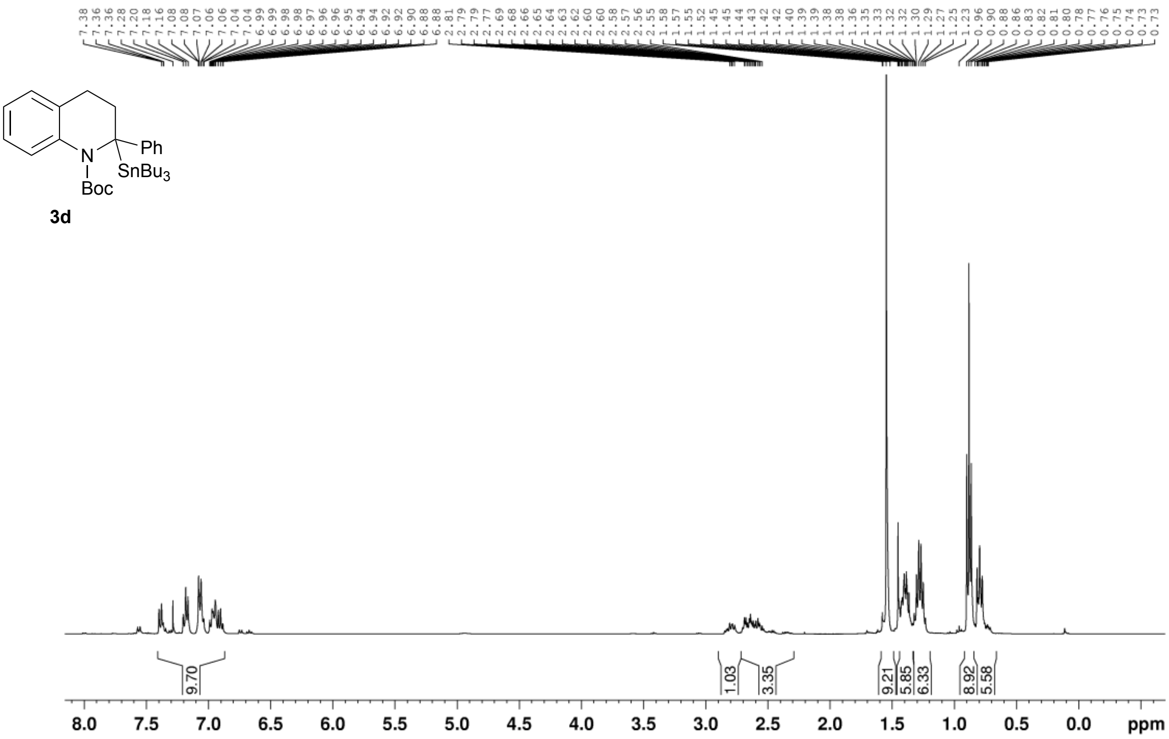
N
Ph
SnBu3
Boc
3d
7.38 7.36 7.28 7.20 7.18 7.16 7.08 7.07 7.06 7.06 7.04 6.99 6.99 6.98 6.98 6.97 6.96 6.96 6.95 6.94 6.92 6.90 6.88 2.81 2.79 2.77 2.69 2.68 2.66 2.65 2.64 2.63 2.62 2.60 2.60 2.58 2.57 2.56 2.55 1.58 1.57 1.55 1.52 1.45 1.45 1.44 1.43 1.42 1.42 1.40 1.39 1.39 1.38 1.38 1.36 1.35 1.33 1.32 1.32 1.30 1.29 1.27 1.25 1.23 0.96 0.90 0.88 0.86 0.83 0.82 0.81 0.80 0.78 0.77 0.76 0.75 0.74 0.73 0.73
9.70
1.03
3.35
9.21
5.85
6.33
8.92
5.58
8.0 7.5 7.0 6.5 6.0 5.5 5.0 4.5 4.0 3.5 3.0 2.5 2.0 1.5 1.0 0.5 0.0 ppm

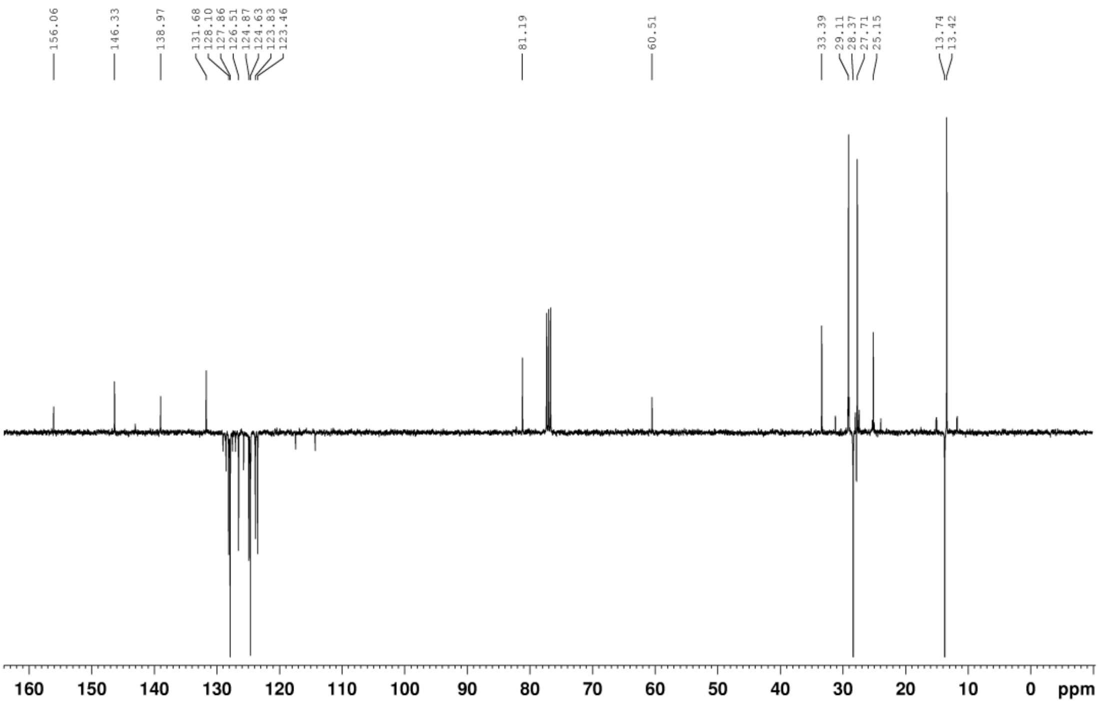
156.06
146.33
138.97
131.68 128.10 127.86 126.51 124.87 124.63 123.83 123.46
81.19
60.51
33.39 29.11 28.37 27.71 25.15
13.74 13.42
160 150 140 130 120 110 100 90 80 70 60 50 40 30 20 10 0 ppm

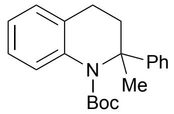
N
Ph
Me
Boc

3e

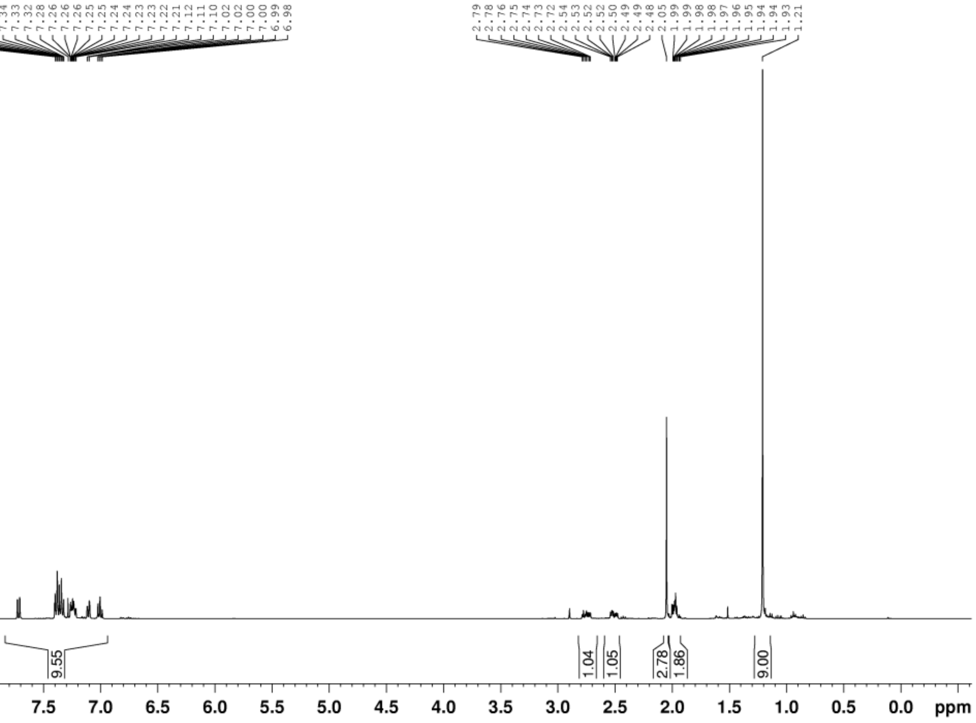
7.40 7.39 7.38 7.37 7.36 7.35 7.34 7.34 7.33 7.32 7.28 7.26 7.26 7.26 7.25 7.25 7.24 7.24 7.23 7.23 7.22 7.21 7.12 7.11 7.10 7.02 7.00 6.99 6.98
2.79 2.78 2.76 2.75 2.74 2.73 2.72 2.54 2.53 2.52 2.52 2.50 2.49 2.49 2.48 2.05 1.99 1.99 1.98 1.98 1.97 1.96 1.95 1.94 1.94 1.93 1.21
9.55
1.04
1.05
2.78
1.86
9.00
8.5 8.0 7.5 7.0 6.5 6.0 5.5 5.0 4.5 4.0 3.5 3.0 2.5 2.0 1.5 1.0 0.5 0.0 ppm

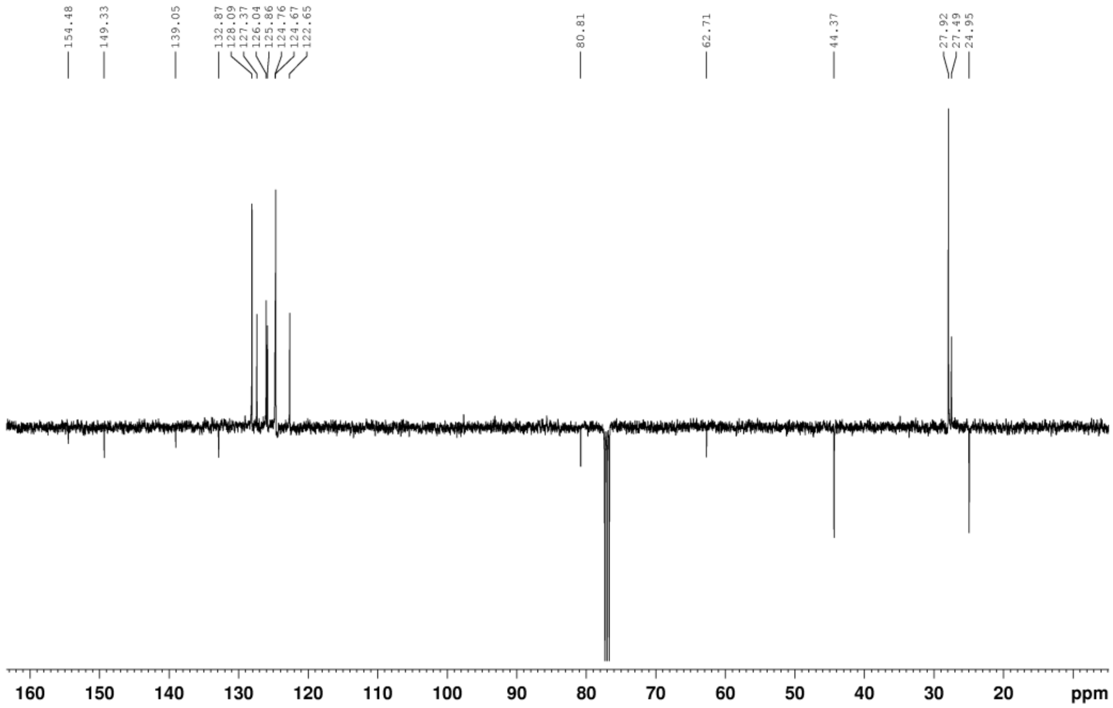
154.48
149.33
139.05
132.87 128.09 127.37 126.04 125.86 124.76 124.67 122.65
80.81
62.71
44.37
27.92 27.49 24.95
160 150 140 130 120 110 100 90 80 70 60 50 40 30 20 ppm

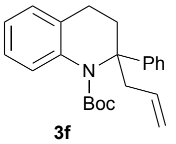
N
Ph
Boc

3f

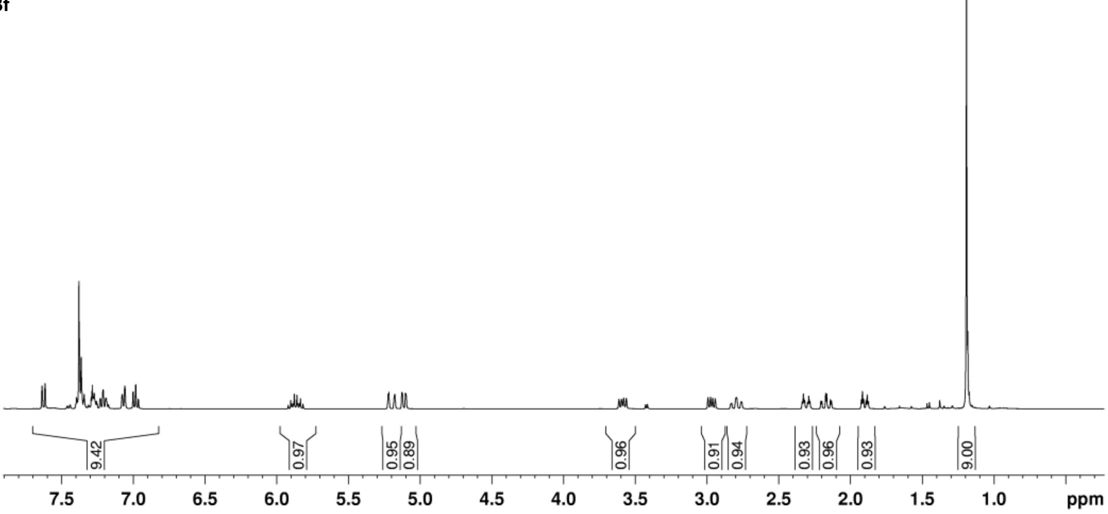
7.61 7.40 7.39 7.39 7.38 7.37 7.36 7.35 7.34 7.29 7.28 7.28 7.28 7.27 7.26 7.26 7.26 7.25 7.23 7.23 7.21 7.19 7.19 7.08 7.06 7.00 7.00 6.98 6.98 6.96 6.96 5.90 5.89 5.88 5.88 5.86 5.85 5.84 5.83 5.82 5.22 5.22 5.18 5.18 5.17 5.13 5.12 5.12 5.10 5.10 5.10 3.61 3.60 3.58 3.56 2.99 2.98 2.96 2.94 2.84 2.83 2.79 2.76 2.34 2.33 2.32 2.30 2.29 2.28 2.21 2.20 2.20 2.17 2.17 2.14 2.13 1.92 1.92 1.91 1.89 1.88 1.88 1.19
9.42
0.97
0.95
0.89
0.96
0.91
0.94
0.93
0.96
0.93
9.00
7.5 7.0 6.5 6.0 5.5 5.0 4.5 4.0 3.5 3.0 2.5 2.0 1.5 1.0 ppm

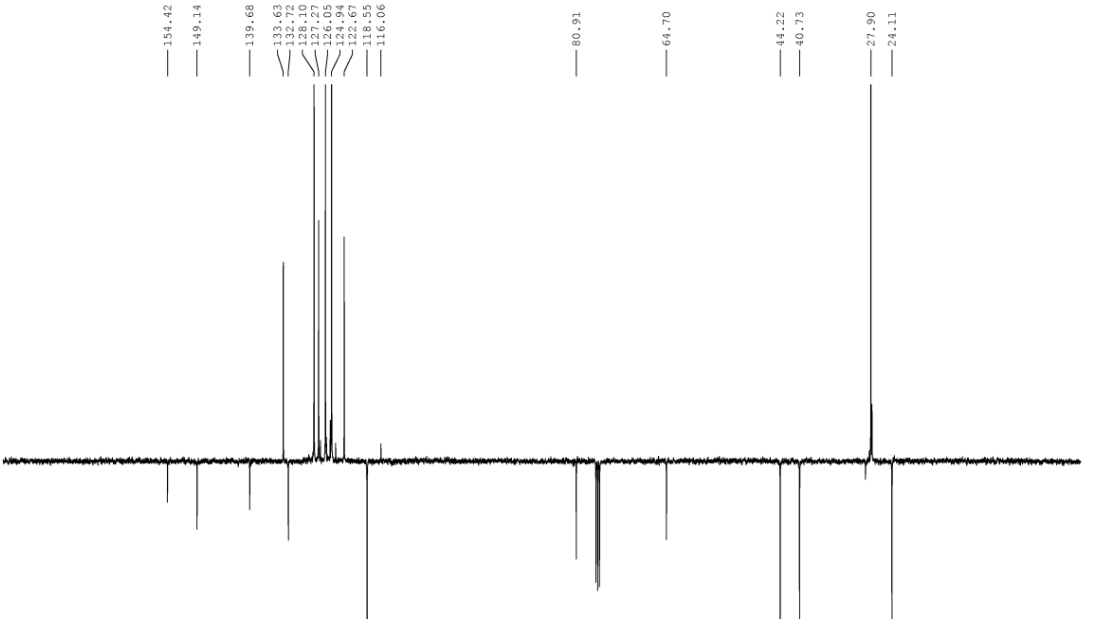
154.42
149.14
139.68
133.63
132.72
128.10
127.27
126.05
124.94
122.67
118.55
116.06
80.91
64.70
44.22
40.73
27.90
24.11
180 170 160 150 140 130 120 110 100 90 80 70 60 50 40 30 20 10 0 ppm

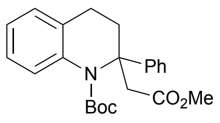
N
Ph
Boc
CO_2Me

3g

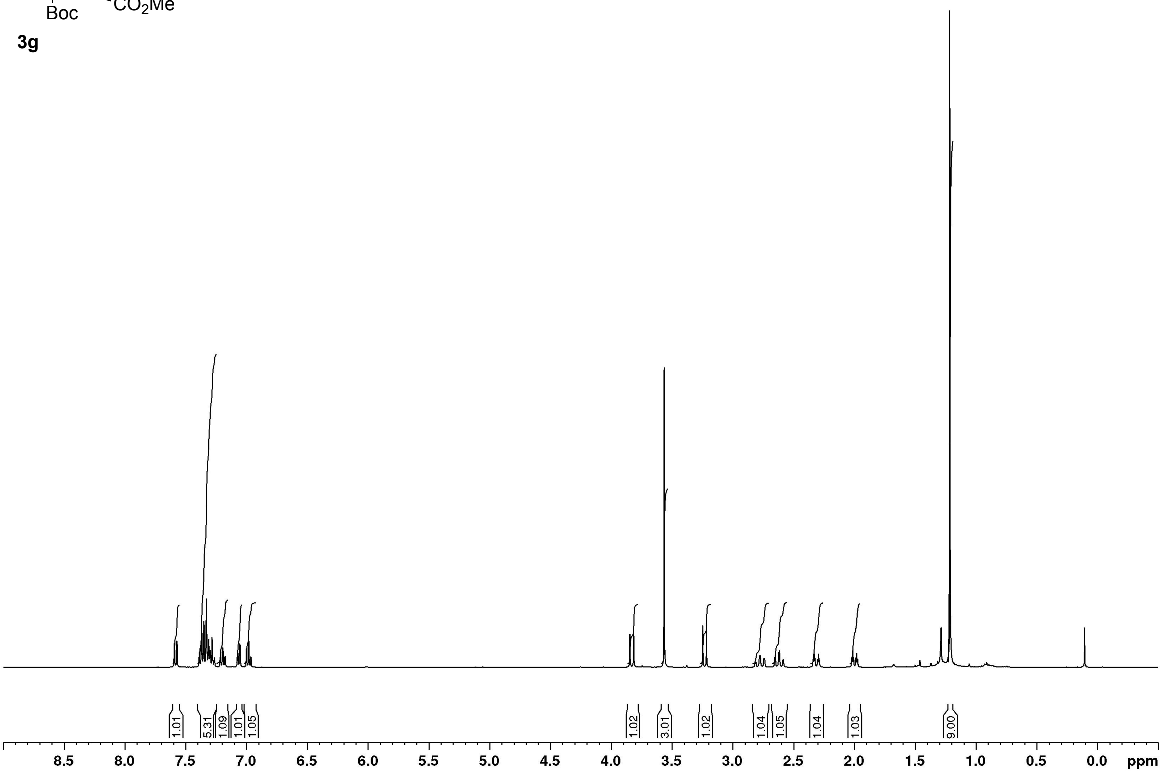
1.01
5.31
1.09
1.01
1.05
1.02
3.01
1.02
1.04
1.05
1.04
1.03
9.00
8.5 8.0 7.5 7.0 6.5 6.0 5.5 5.0 4.5 4.0 3.5 3.0 2.5 2.0 1.5 1.0 0.5 0.0 ppm

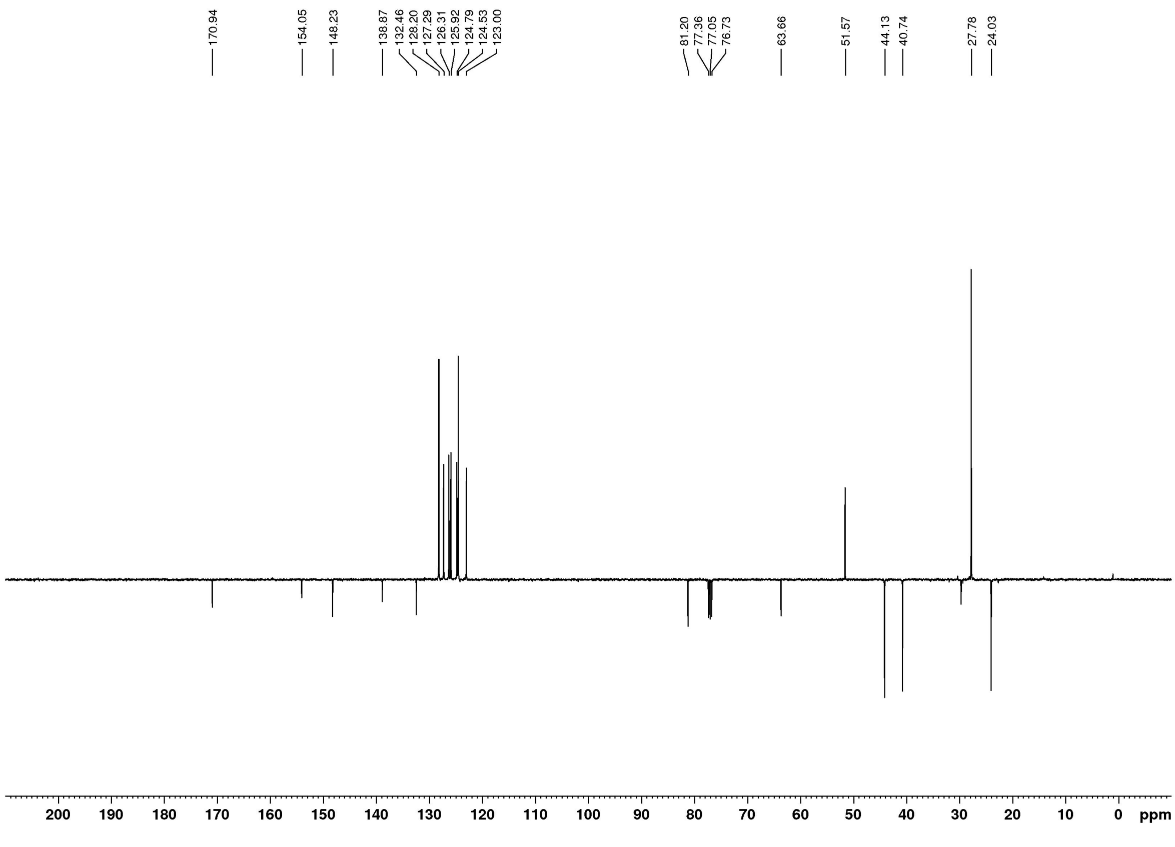
170.94
154.05
148.23
138.87
132.46
128.20
127.29
126.31
125.92
124.79
124.53
123.00
81.20
77.36
77.05
76.73
63.66
51.57
44.13
40.74
27.78
24.03
200 190 180 170 160 150 140 130 120 110 100 90 80 70 60 50 40 30 20 10 0 ppm

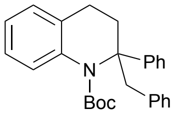
N
Ph
Boc
Ph

3h

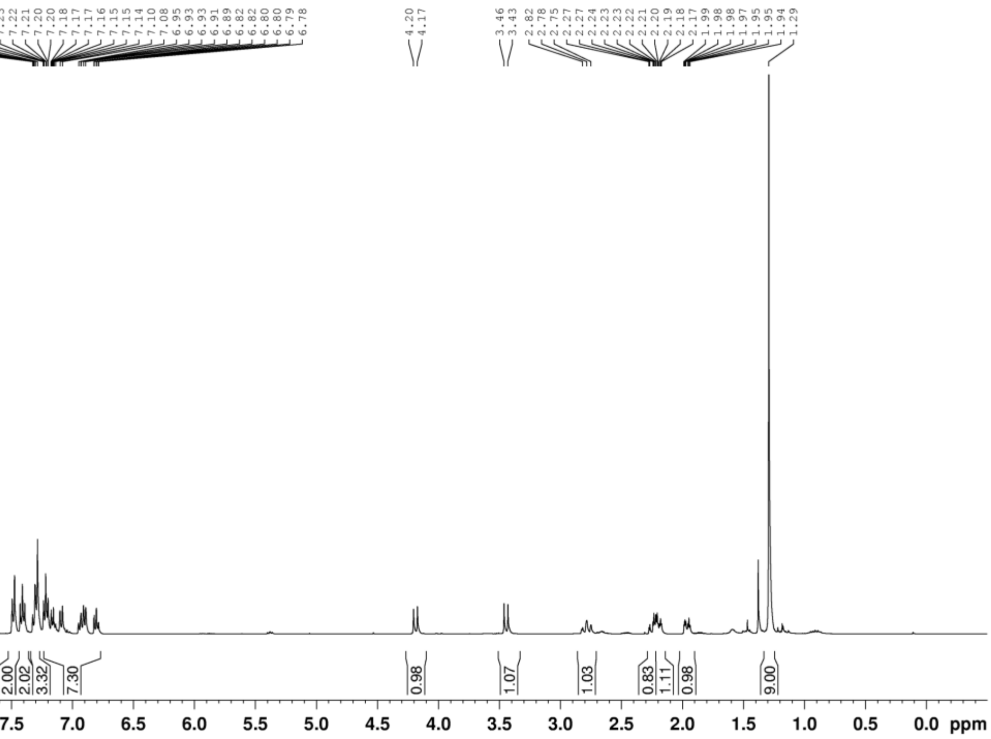
7.32 7.32 7.31 7.30 7.28 7.24 7.23 7.22 7.21 7.20 7.20 7.18 7.17 7.17 7.16 7.15 7.14 7.10 7.08 6.95 6.93 6.93 6.91 6.89 6.82 6.80 6.80 6.79 6.78
4.20 4.17
3.46 3.43 2.82 2.78 2.75 2.27 2.27 2.24 2.23 2.22 2.21 2.20 2.19 2.18 2.17 1.99 1.98 1.98 1.97 1.95 1.95 1.94 1.29
2.00 2.02 3.32 7.30 0.98 1.07 1.03 0.83 1.11 0.98 9.00
8.0 7.5 7.0 6.5 6.0 5.5 5.0 4.5 4.0 3.5 3.0 2.5 2.0 1.5 1.0 0.5 0.0 ppm

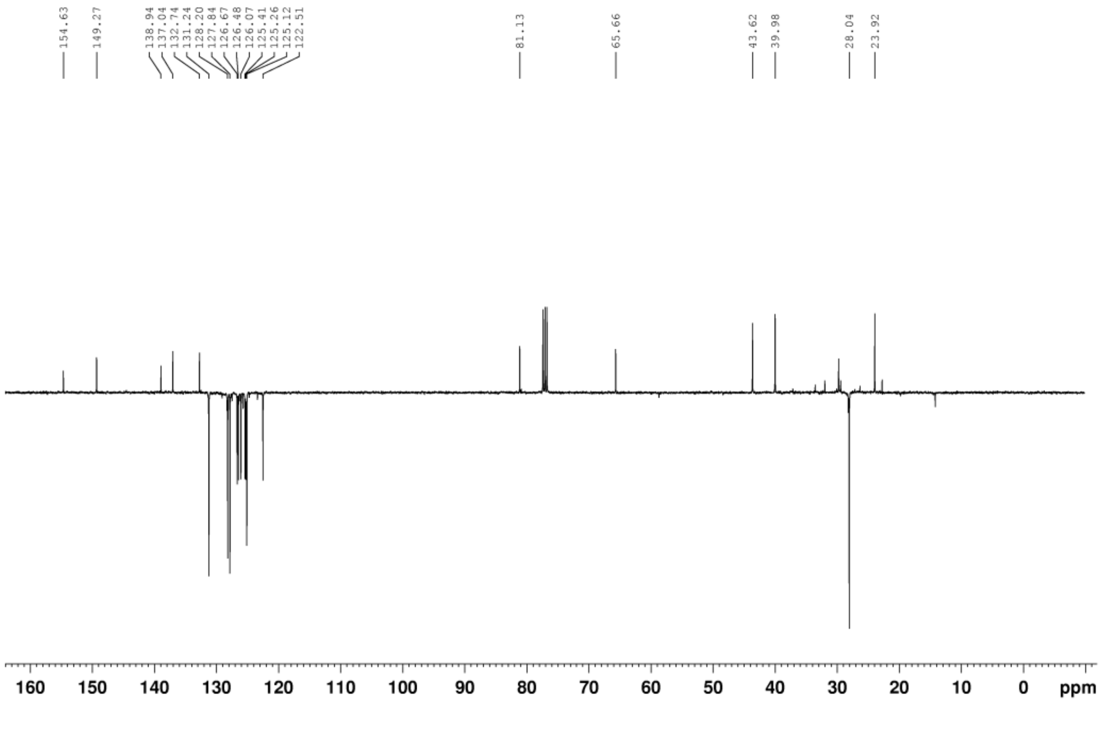
154.63
149.27
138.94 137.04 132.74 131.24 128.20 127.84 126.67 126.48 126.07 125.41 125.26 125.12 122.51
81.13
65.66
43.62
39.98
28.04
23.92
160 150 140 130 120 110 100 90 80 70 60 50 40 30 20 10 0 ppm

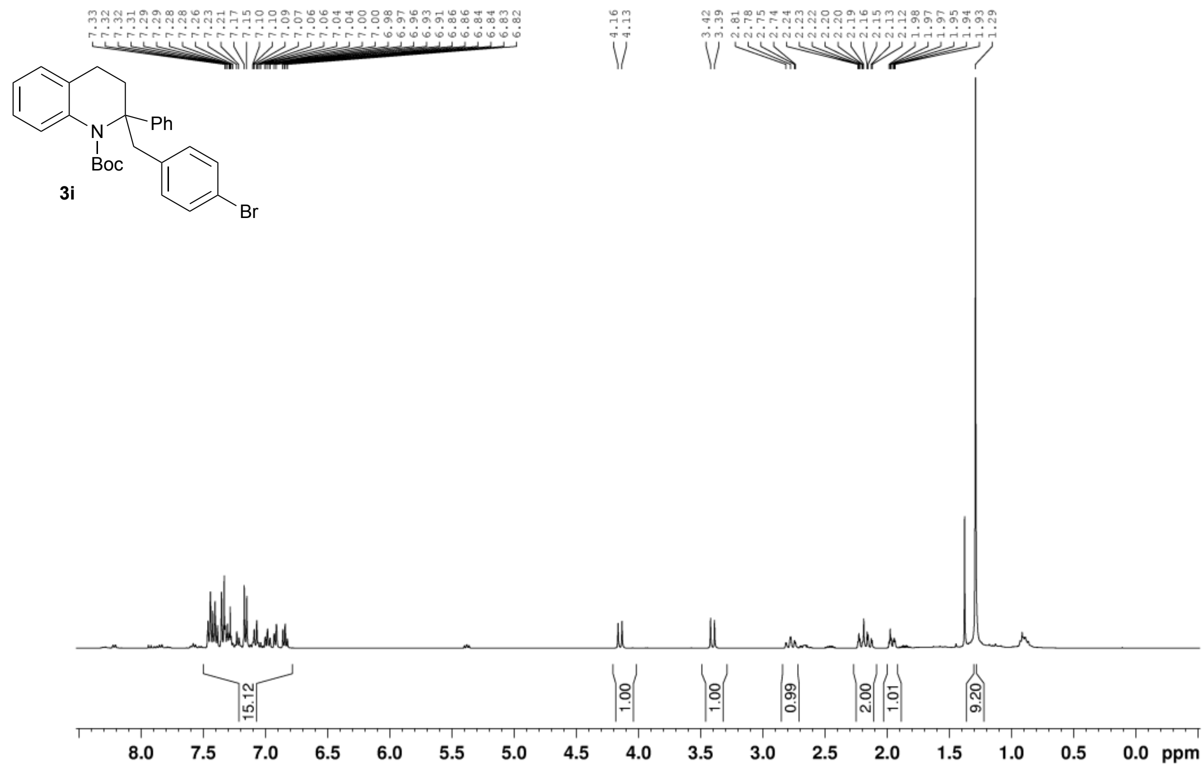

7.33 7.32 7.31 7.29 7.29 7.28 7.26 7.23 7.21 7.17 7.15 7.10 7.10 7.09 7.07 7.06 7.06 7.04 7.04 7.00 6.98 6.97 6.96 6.93 6.91 6.86 6.86 6.84 6.83 6.82
4.16 4.13
3.42 3.39 2.81 2.78 2.75 2.74 2.24 2.23 2.22 2.20 2.20 2.19 2.16 2.15 2.13 2.12 1.98 1.97 1.95 1.94 1.93 1.29
N
Ph
Boc
Br
3i
15.12
1.00
1.00
0.99
2.00
1.01
9.20
8.0 7.5 7.0 6.5 6.0 5.5 5.0 4.5 4.0 3.5 3.0 2.5 2.0 1.5 1.0 0.5 0.0 ppm

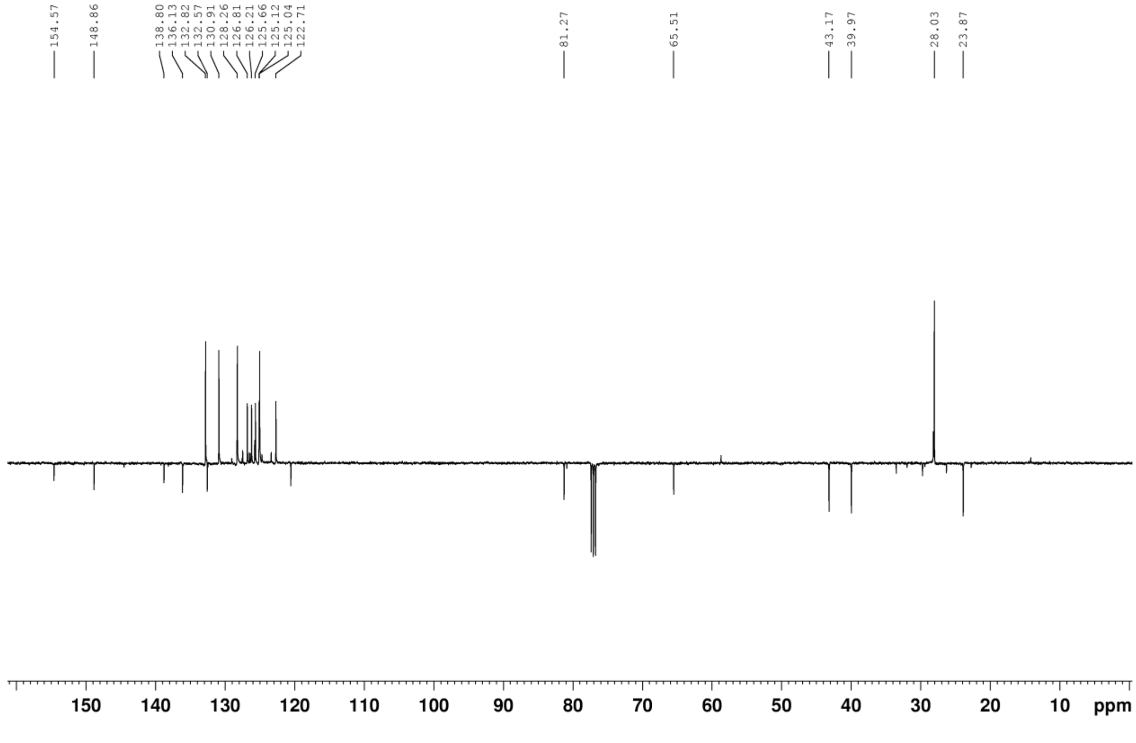

154.57
148.86
138.80 136.13 132.82 132.57 130.91 128.26 126.81 126.21 125.66 125.12 125.04 122.71
81.27
65.51
43.17 39.97
28.03 23.87
150 140 130 120 110 100 90 80 70 60 50 40 30 20 10 ppm

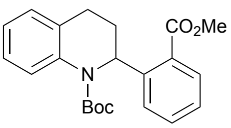
CO2Me
N
Boc

4

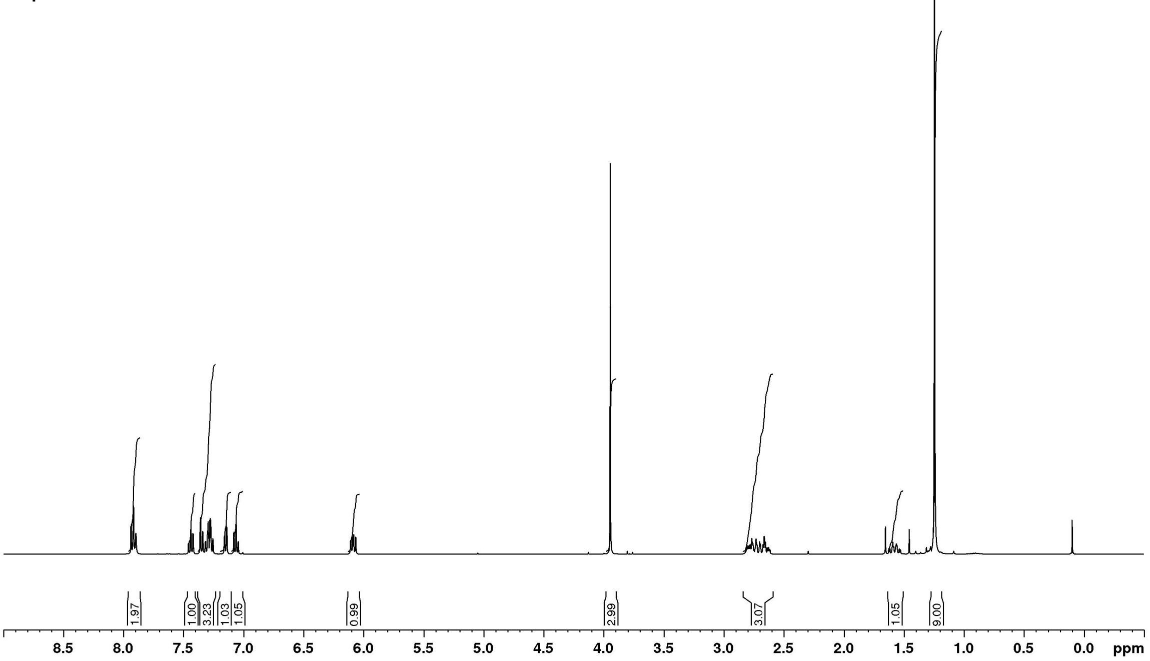
1.97
1.00
3.23
1.03
1.05
0.99
2.99
3.07
1.05
9.00
8.5 8.0 7.5 7.0 6.5 6.0 5.5 5.0 4.5 4.0 3.5 3.0 2.5 2.0 1.5 1.0 0.5 0.0 ppm

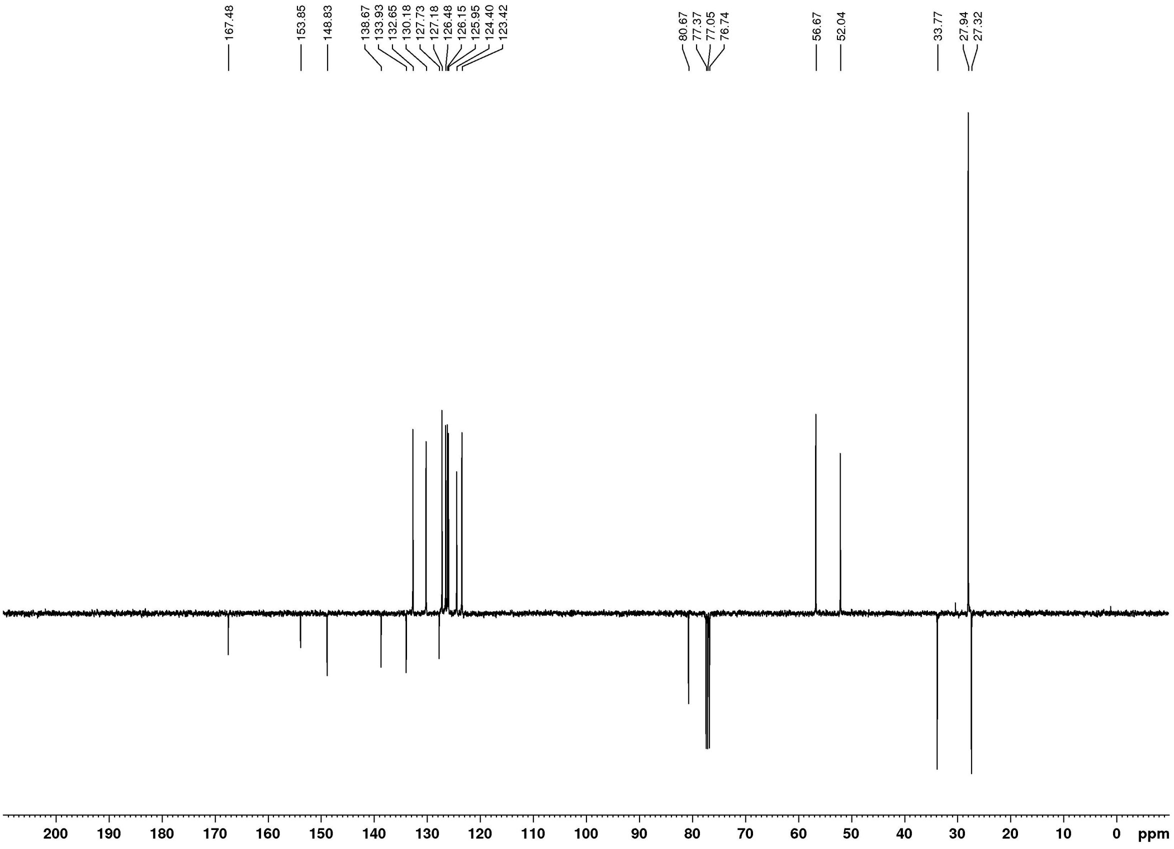
167.48
153.85
148.83
138.67
133.93
132.65
130.18
127.73
127.18
126.48
126.15
125.95
124.40
123.42
80.67
77.37
77.05
76.74
56.67
52.04
33.77
27.94
27.32
200 190 180 170 160 150 140 130 120 110 100 90 80 70 60 50 40 30 20 10 0 ppm

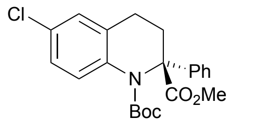

Cl
N
Ph
CO_2Me
Boc

5

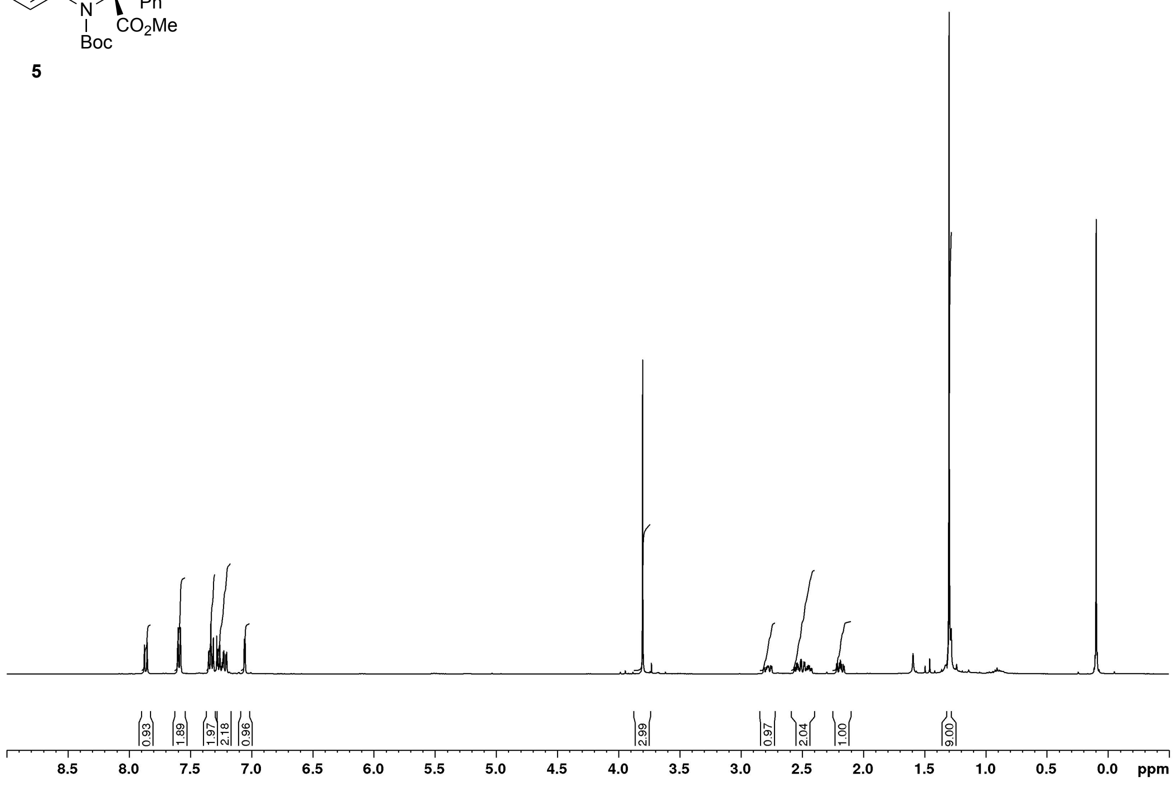

0.93
1.89
1.97
2.18
0.96
2.99
0.97
2.04
1.00
9.00
8.5 8.0 7.5 7.0 6.5 6.0 5.5 5.0 4.5 4.0 3.5 3.0 2.5 2.0 1.5 1.0 0.5 0.0 ppm

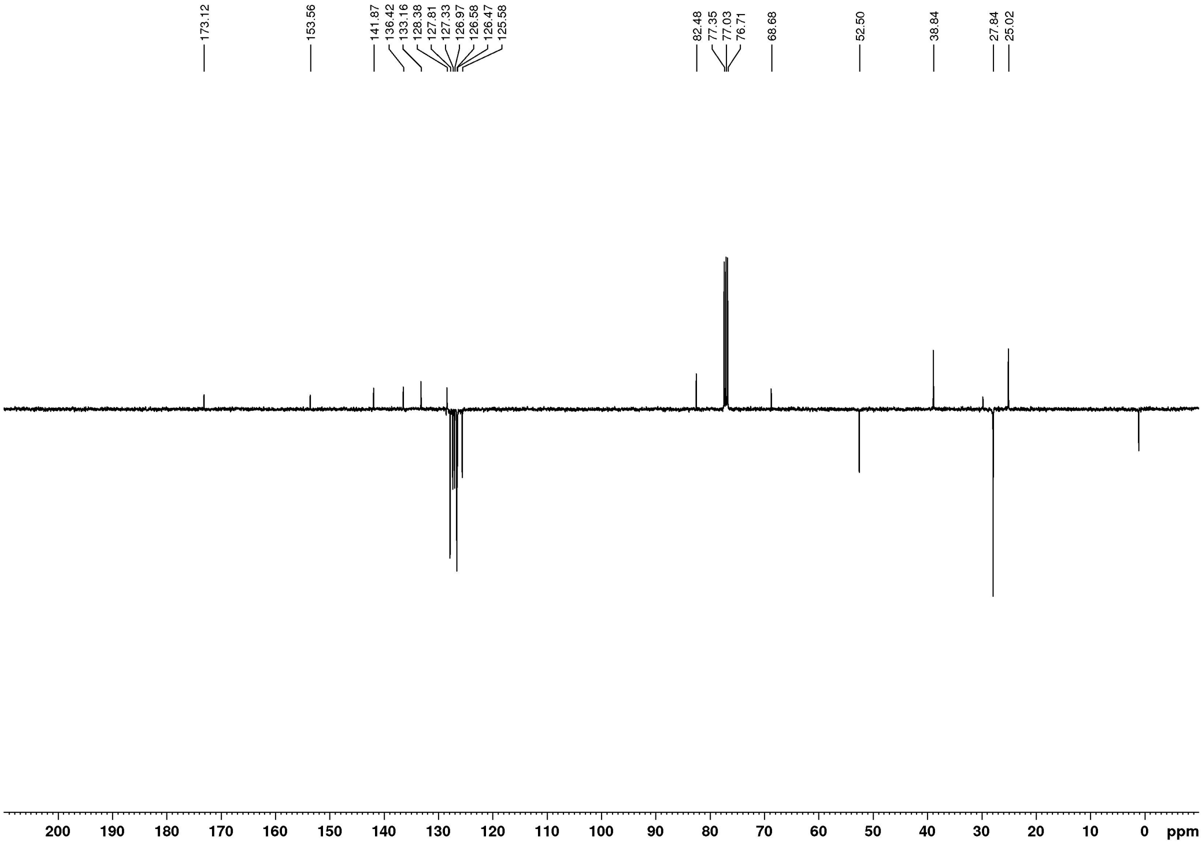

173.12
153.56
141.87
136.42
133.16
128.38
127.81
127.33
126.97
126.58
126.47
125.58
82.48
77.35
77.03
76.71
68.68
52.50
38.84
27.84
25.02
200 190 180 170 160 150 140 130 120 110 100 90 80 70 60 50 40 30 20 10 0 ppm

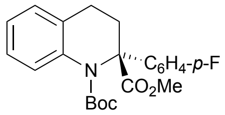
N
C6H4-p-F
CO2Me
Boc

6

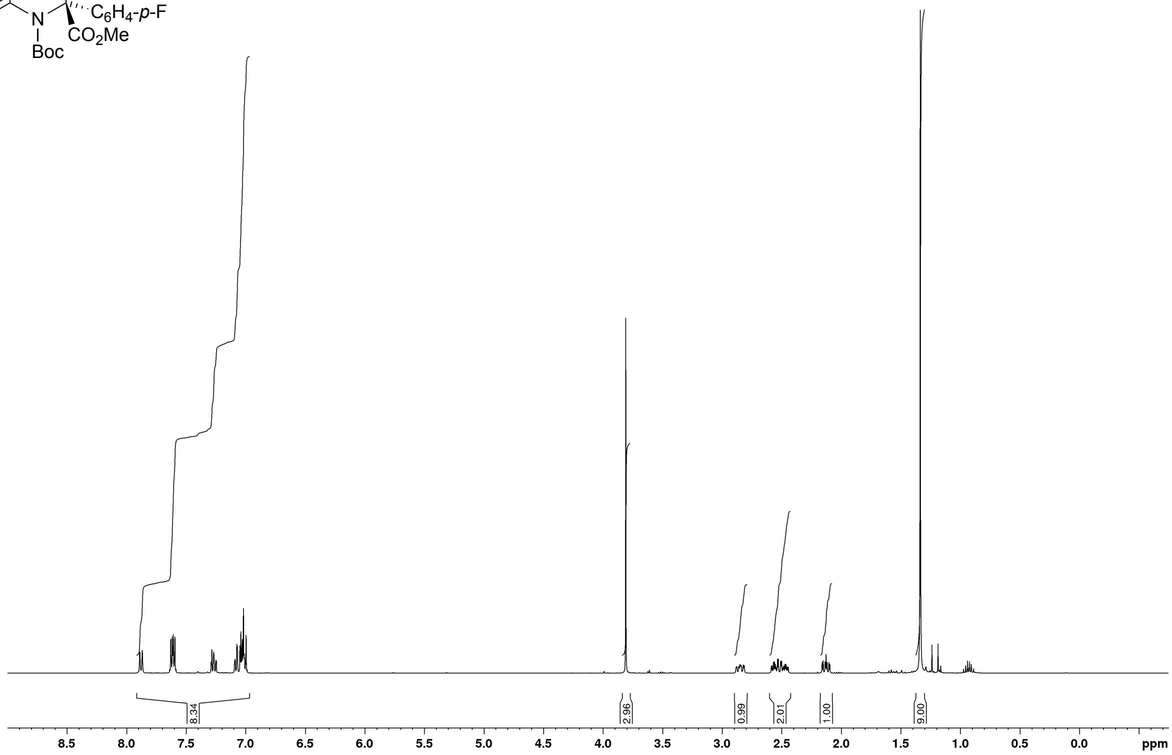
8.34
2.96
0.99
2.01
1.00
9.00
8.5 8.0 7.5 7.0 6.5 6.0 5.5 5.0 4.5 4.0 3.5 3.0 2.5 2.0 1.5 1.0 0.5 0.0 ppm

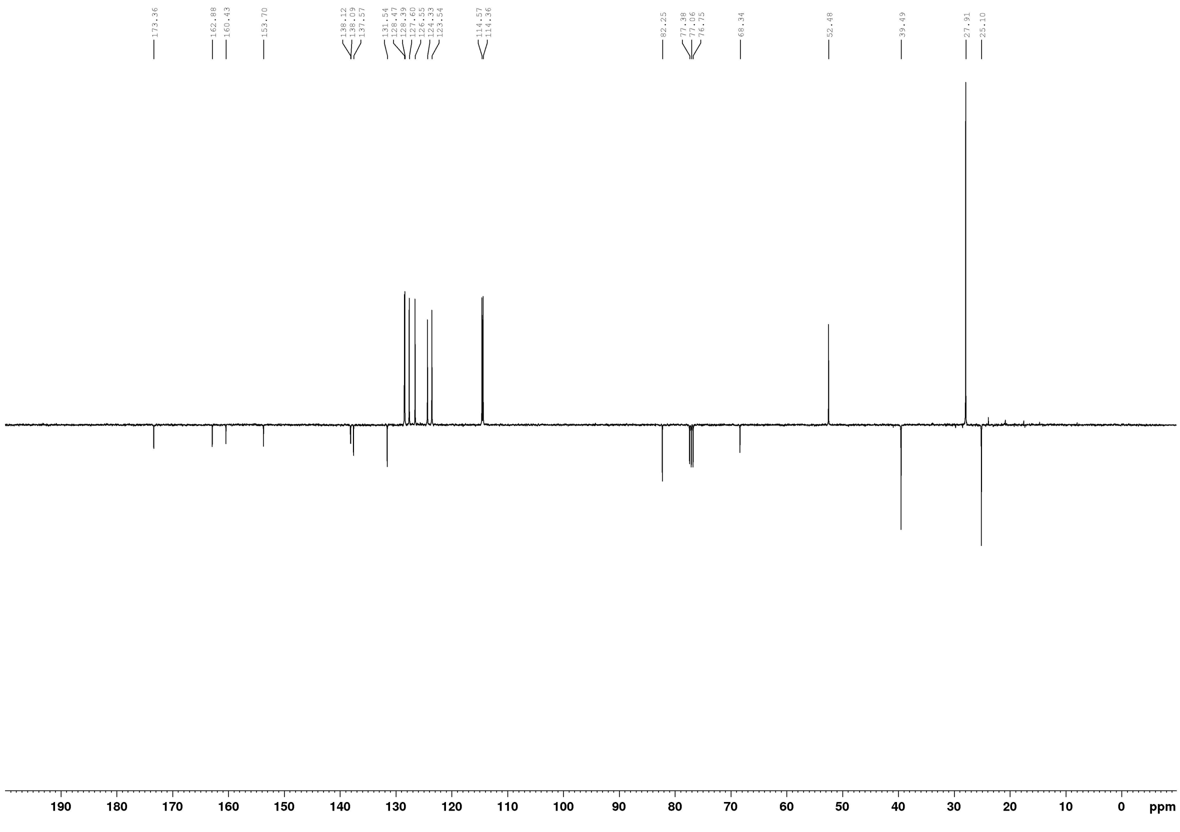
173.36
162.88
160.43
153.70
138.12
138.09
137.57
131.54
128.47
128.39
127.60
126.55
124.33
123.54
114.57
114.36
82.25
77.38
77.06
76.75
68.34
52.48
39.49
27.91
25.10
190 180 170 160 150 140 130 120 110 100 90 80 70 60 50 40 30 20 10 0 ppm

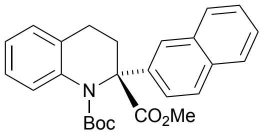
N
Boc
CO2Me

7

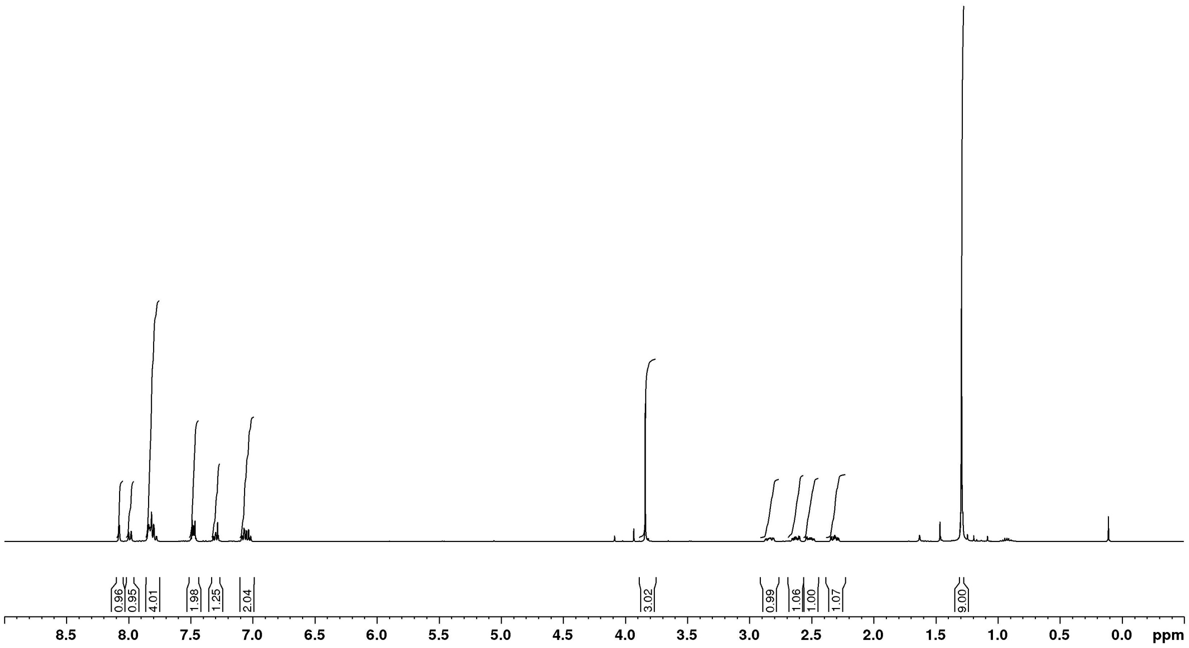
0.96
0.95
4.01
1.98
1.25
2.04
3.02
0.99
1.06
1.00
1.07
9.00
8.5 8.0 7.5 7.0 6.5 6.0 5.5 5.0 4.5 4.0 3.5 3.0 2.5 2.0 1.5 1.0 0.5 0.0 ppm

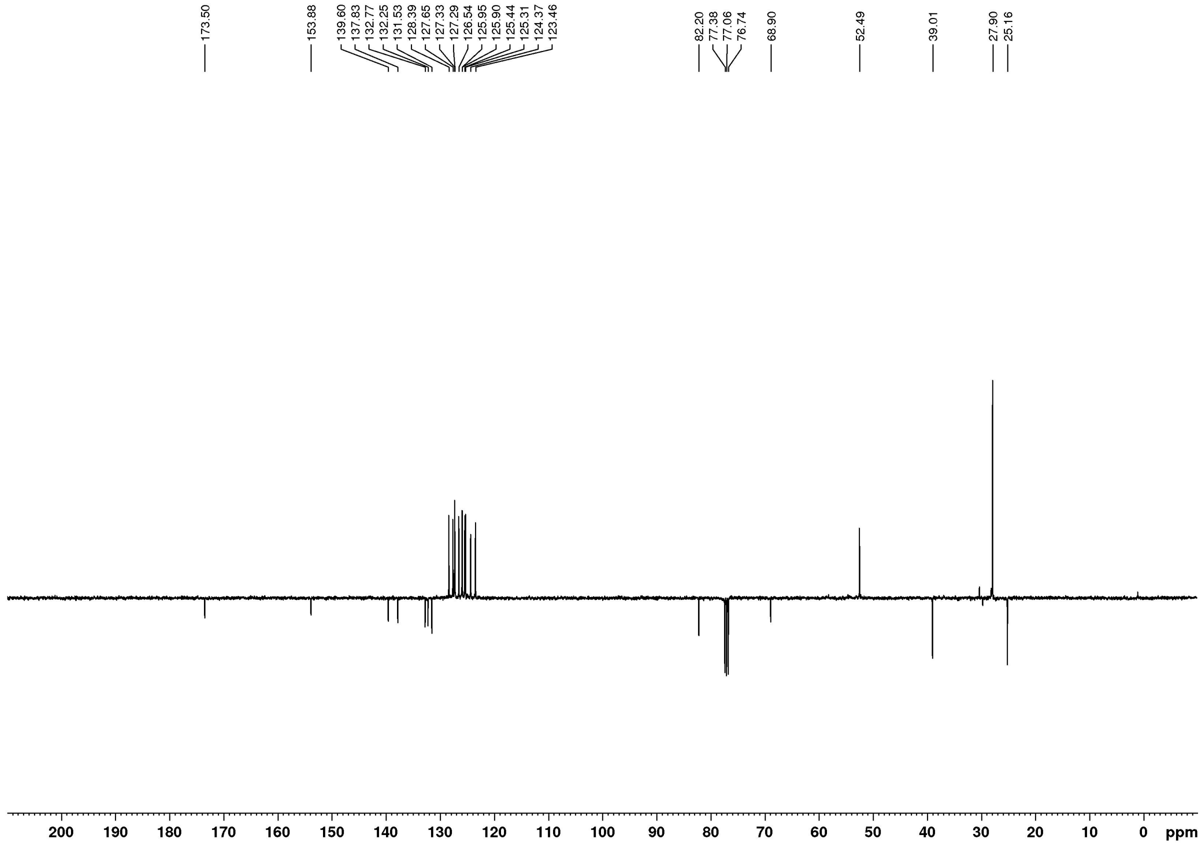
173.50
153.88
139.60
137.83
132.77
132.25
131.53
128.39
127.65
127.33
127.29
126.54
125.95
125.90
125.44
125.31
124.37
123.46
82.20
77.38
77.06
76.74
68.90
52.49
39.01
27.90
25.16
200 190 180 170 160 150 140 130 120 110 100 90 80 70 60 50 40 30 20 10 0 ppm

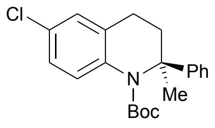
Cl
N
Ph
Me
Boc

8

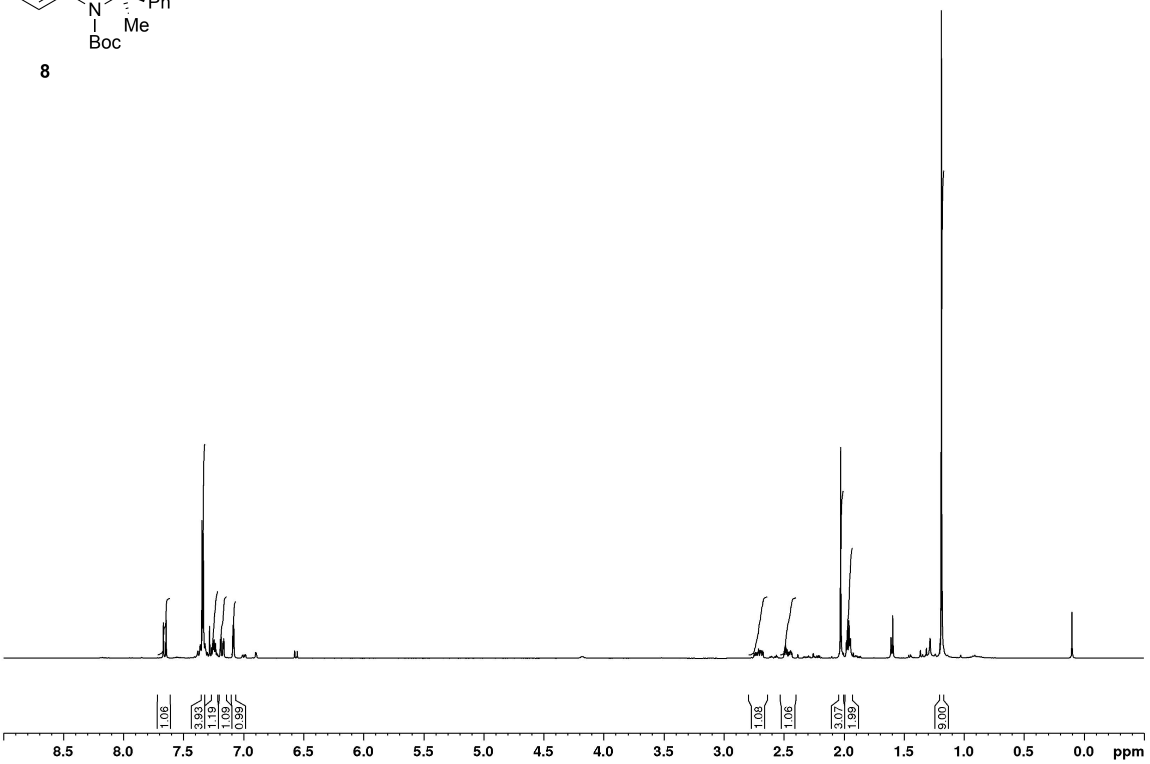
1.06
3.93
1.19
1.09
0.99
1.08
1.06
3.07
1.99
9.00
8.5 8.0 7.5 7.0 6.5 6.0 5.5 5.0 4.5 4.0 3.5 3.0 2.5 2.0 1.5 1.0 0.5 0.0 ppm

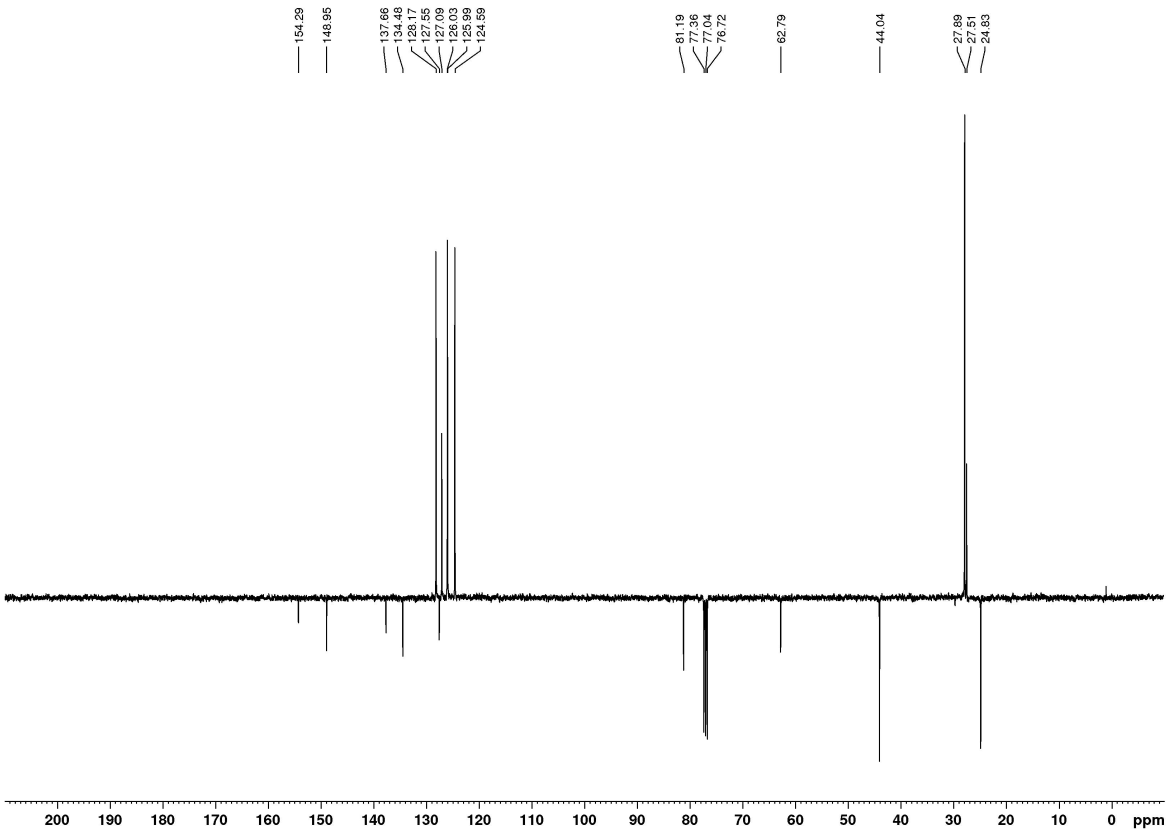
154.29
148.95
137.66
134.48
128.17
127.55
127.09
126.03
125.99
124.59
81.19
77.36
77.04
76.72
62.79
44.04
27.89
27.51
24.83
200 190 180 170 160 150 140 130 120 110 100 90 80 70 60 50 40 30 20 10 0 ppm

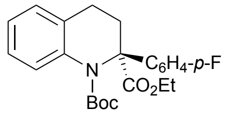

N
Boc
C_6H_4-*p*-F
CO_2Et

9

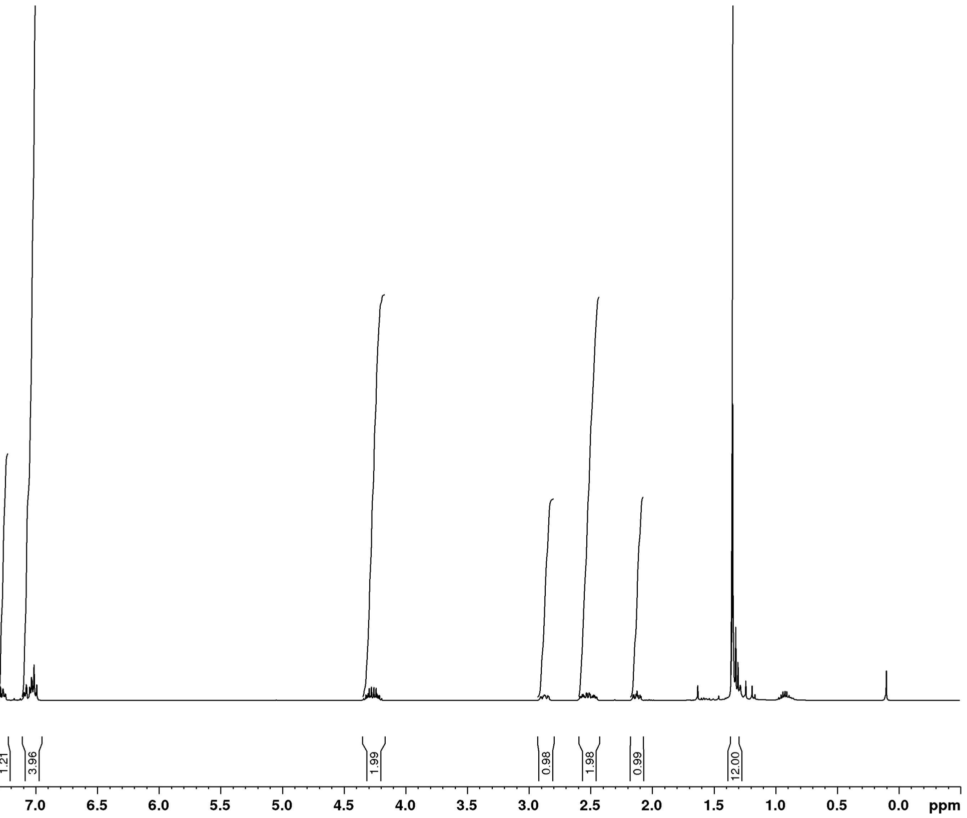

0.96
1.97
1.21
3.96
1.99
0.98
1.98
0.99
12.00
8.5 8.0 7.5 7.0 6.5 6.0 5.5 5.0 4.5 4.0 3.5 3.0 2.5 2.0 1.5 1.0 0.5 0.0 ppm

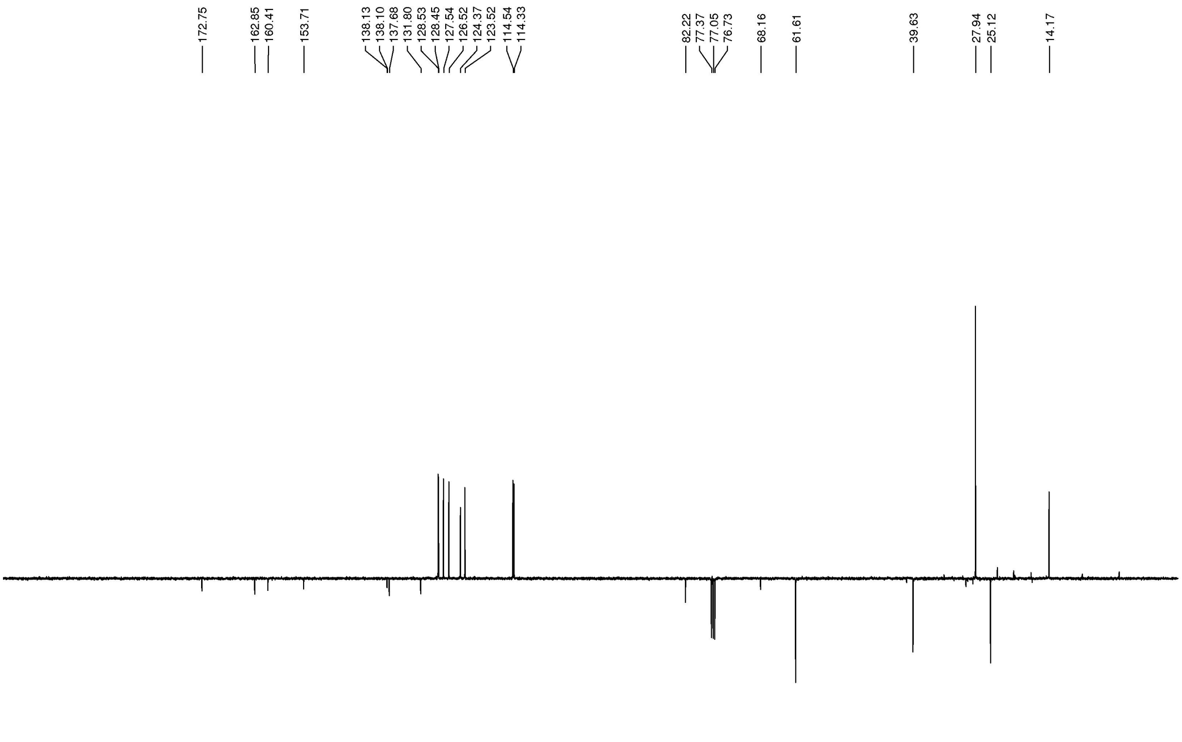

172.75
162.85
160.41
153.71
138.13
138.10
137.68
131.80
128.53
128.45
127.54
126.52
124.37
123.52
114.54
114.33
82.22
77.37
77.05
76.73
68.16
61.61
39.63
27.94
25.12
14.17
200 190 180 170 160 150 140 130 120 110 100 90 80 70 60 50 40 30 20 10 0 ppm

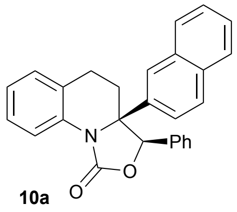

N
Ph
O
O

10a

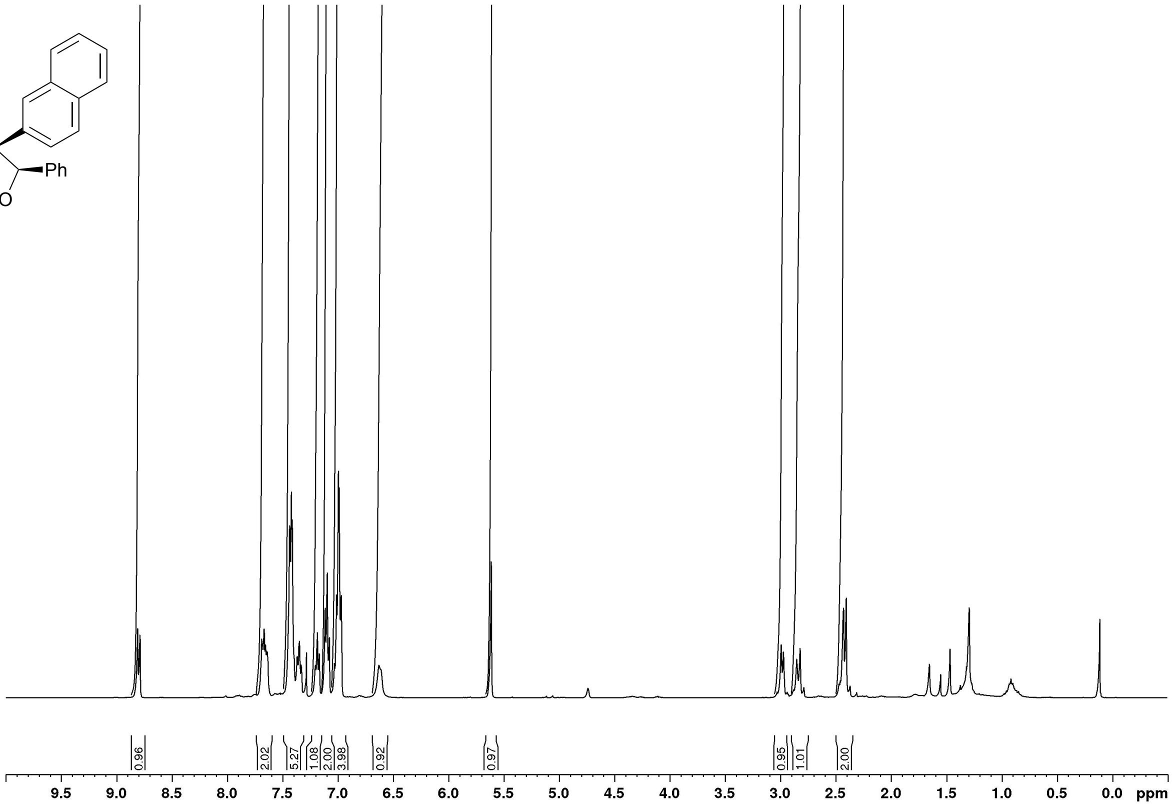

0.96
2.02
5.27
1.08
2.00
3.98
0.92
0.97
0.95
1.01
2.00
9.5 9.0 8.5 8.0 7.5 7.0 6.5 6.0 5.5 5.0 4.5 4.0 3.5 3.0 2.5 2.0 1.5 1.0 0.5 0.0 ppm

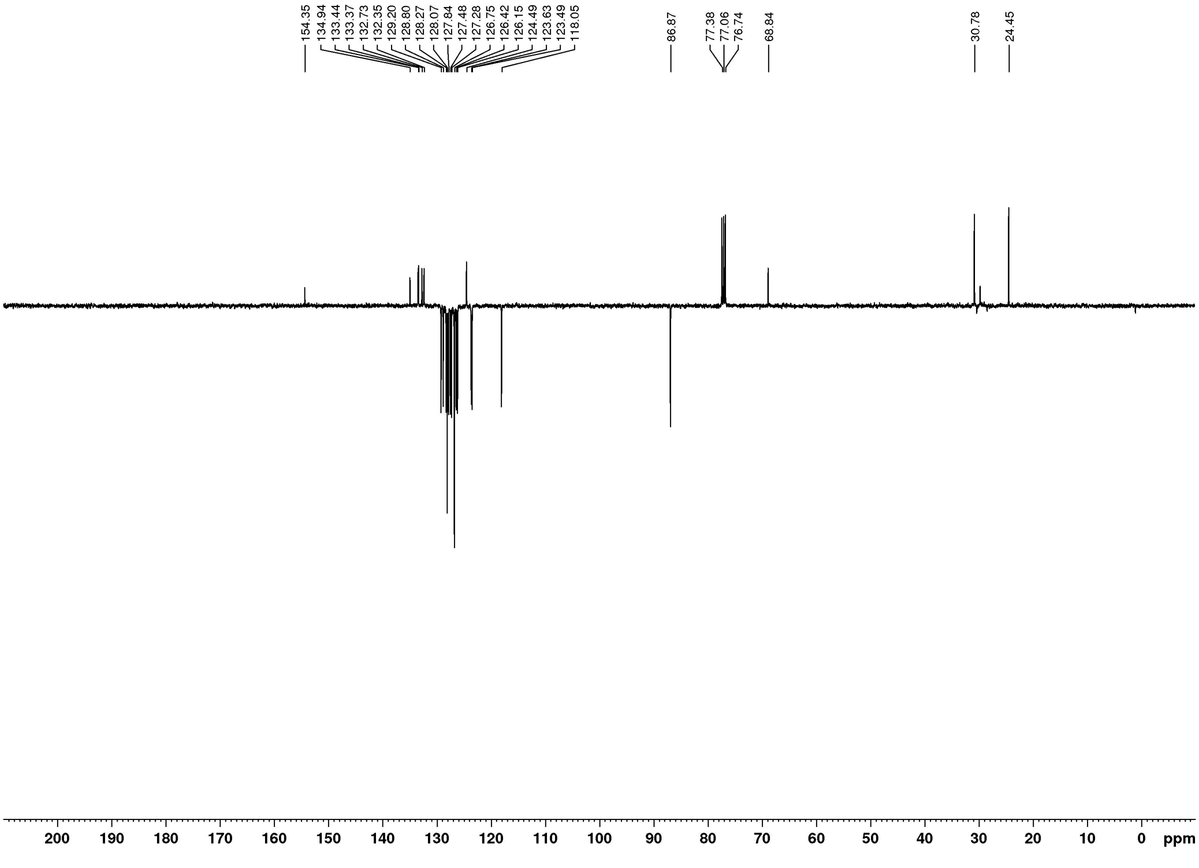

154.35
134.94
133.44
133.37
132.73
132.35
129.20
128.80
128.27
128.07
127.84
127.48
127.28
126.75
126.42
126.15
124.49
123.63
123.49
118.05
86.87
77.38
77.06
76.74
68.84
30.78
24.45
200 190 180 170 160 150 140 130 120 110 100 90 80 70 60 50 40 30 20 10 0 ppm

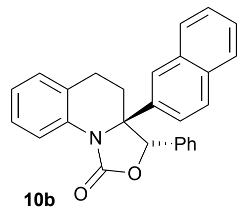

10b

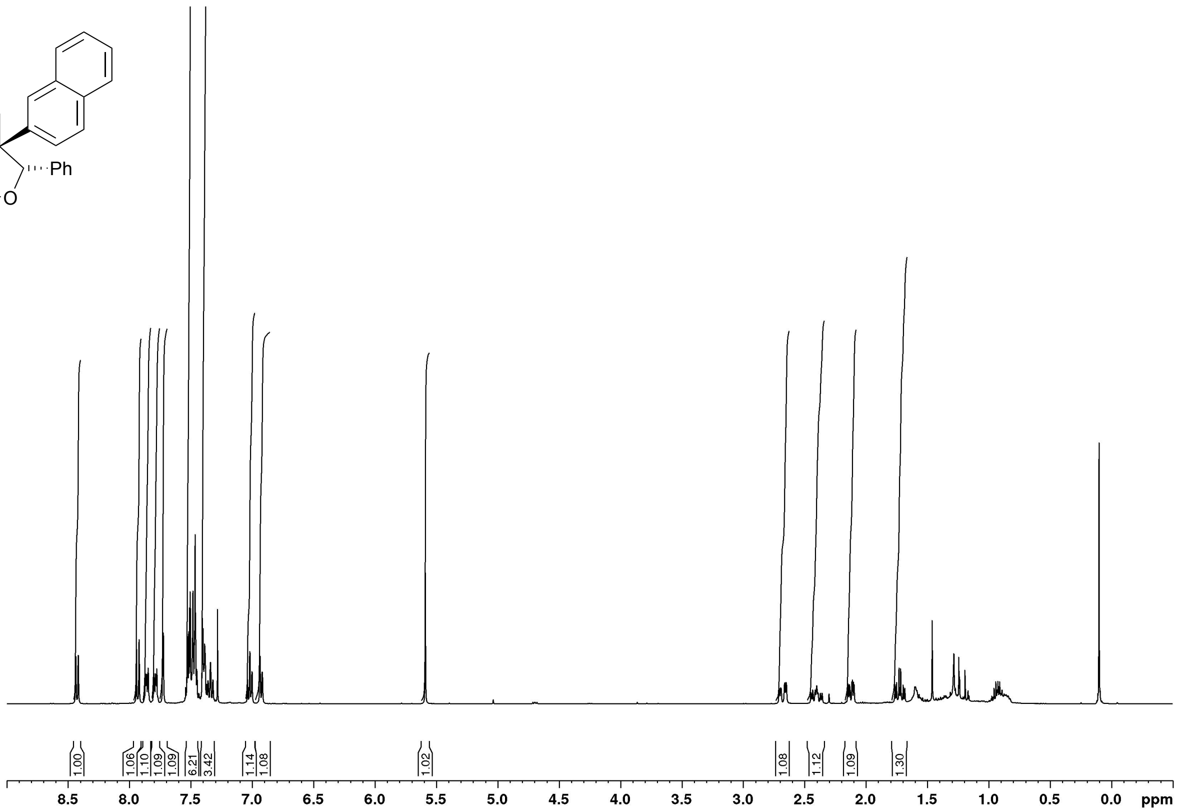

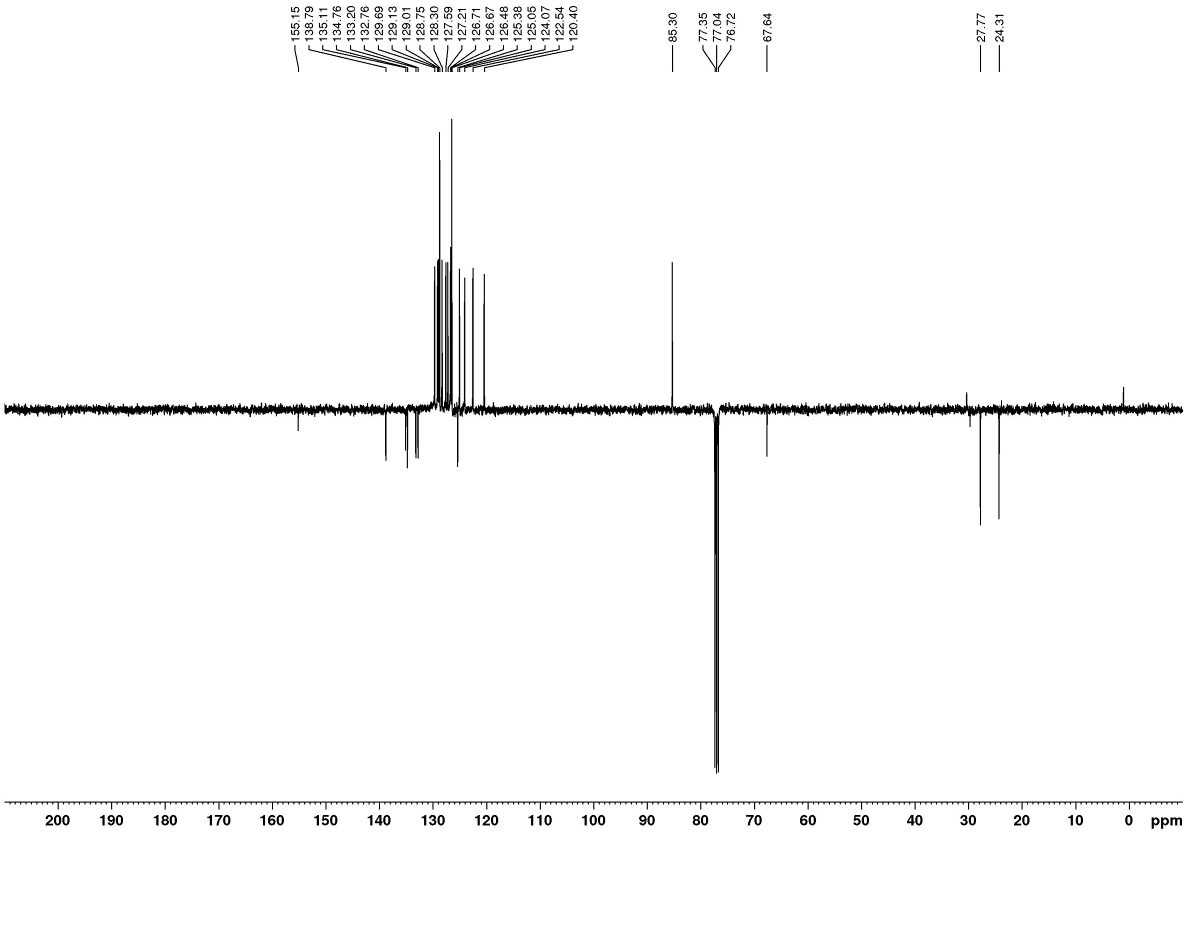

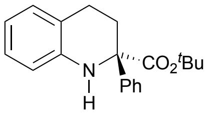

CO2tBu
N
H
Ph

11

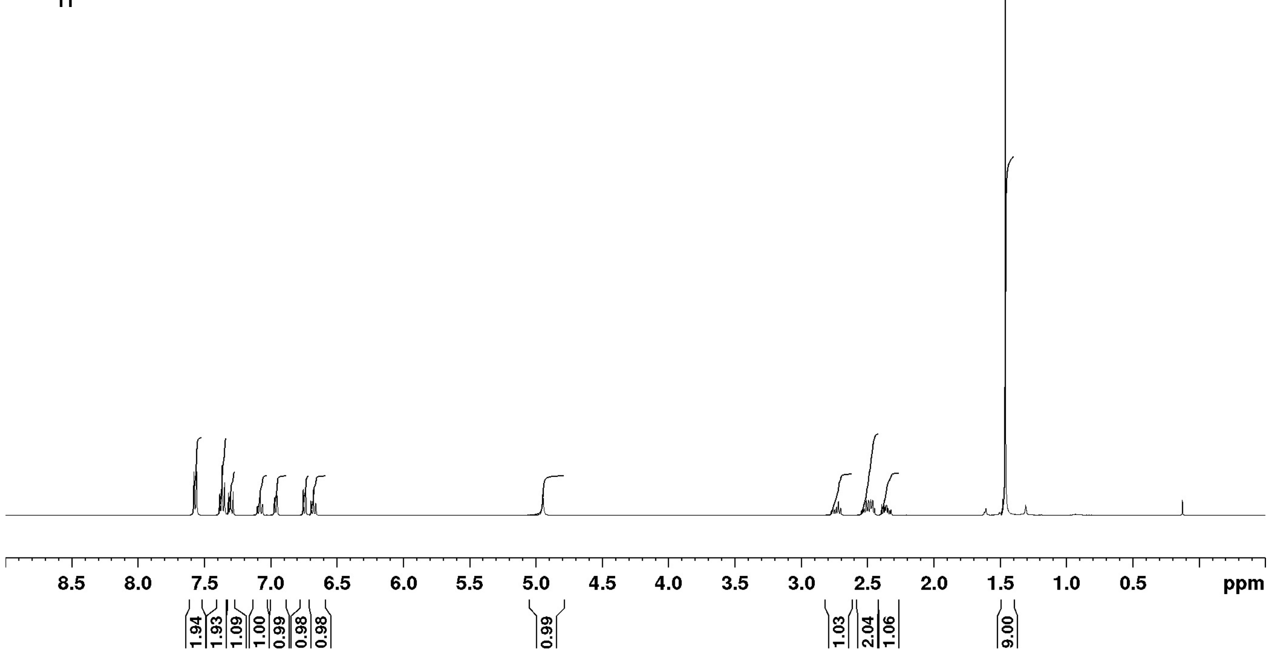

7.58 7.58 7.57 7.56 7.39 7.39 7.38 7.37 7.35 7.32 7.32 7.31 7.30 7.30 7.28 7.10 7.08 7.06 6.97 6.95 6.76 6.74 6.70 6.68 6.66
4.95
2.77 2.76 2.75 2.73 2.72 2.70 2.55 2.53 2.52 2.51 2.49 2.47 2.46 2.45 2.39 2.38 2.37 2.36 2.35 2.33 2.32 1.46
8.5 8.0 7.5 7.0 6.5 6.0 5.5 5.0 4.5 4.0 3.5 3.0 2.5 2.0 1.5 1.0 0.5 ppm
1.94 1.93 1.09 1.00 0.99 0.98 0.98
0.99
1.03 2.04 1.06
9.00

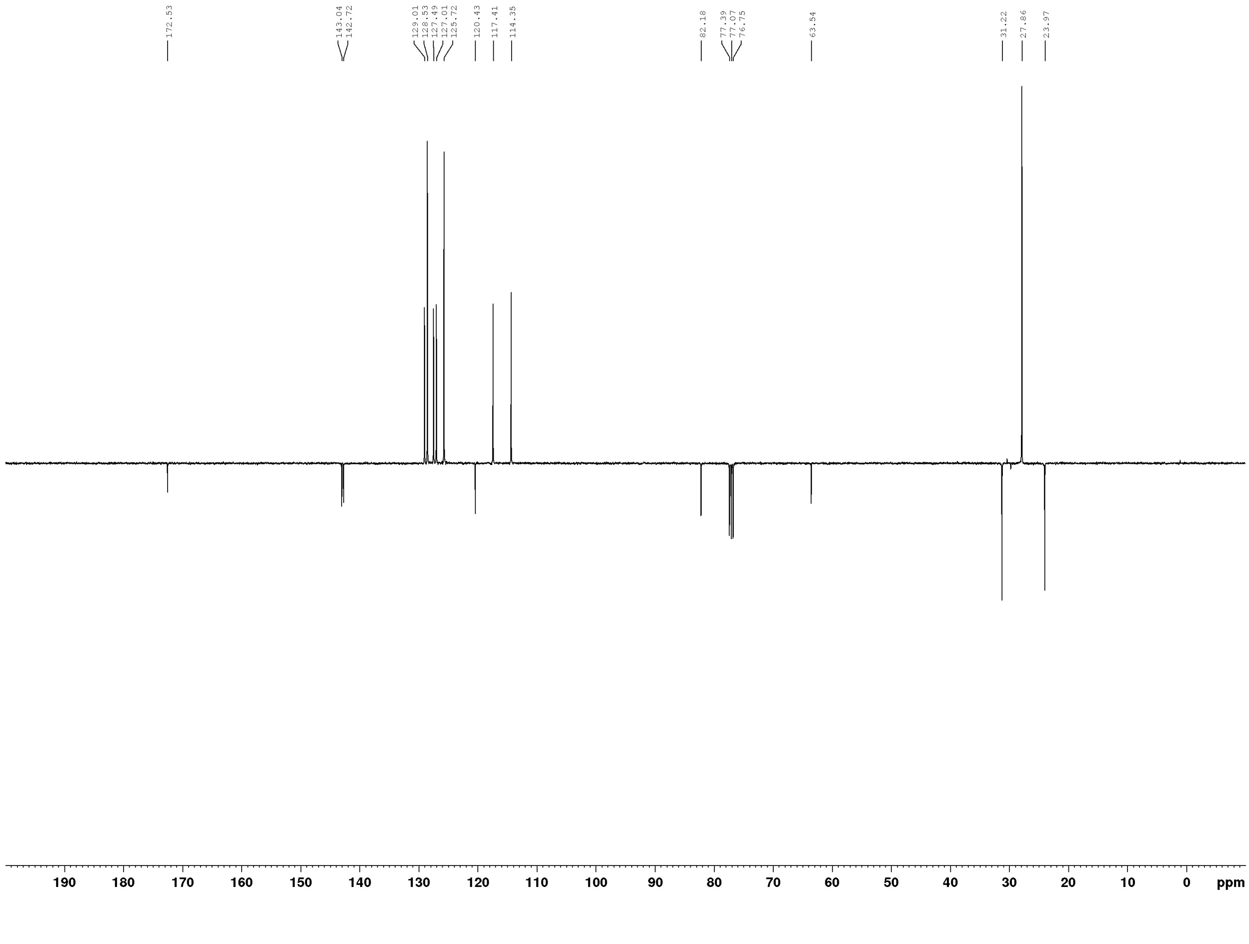

172.53
143.04 142.72
129.01 128.53 127.49 127.01 125.72
120.43
117.41
114.35
82.18
77.39 77.07 76.75
63.54
31.22
27.86
23.97
190 180 170 160 150 140 130 120 110 100 90 80 70 60 50 40 30 20 10 0 ppm

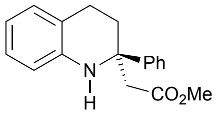
N
H
Ph
CO2Me

12

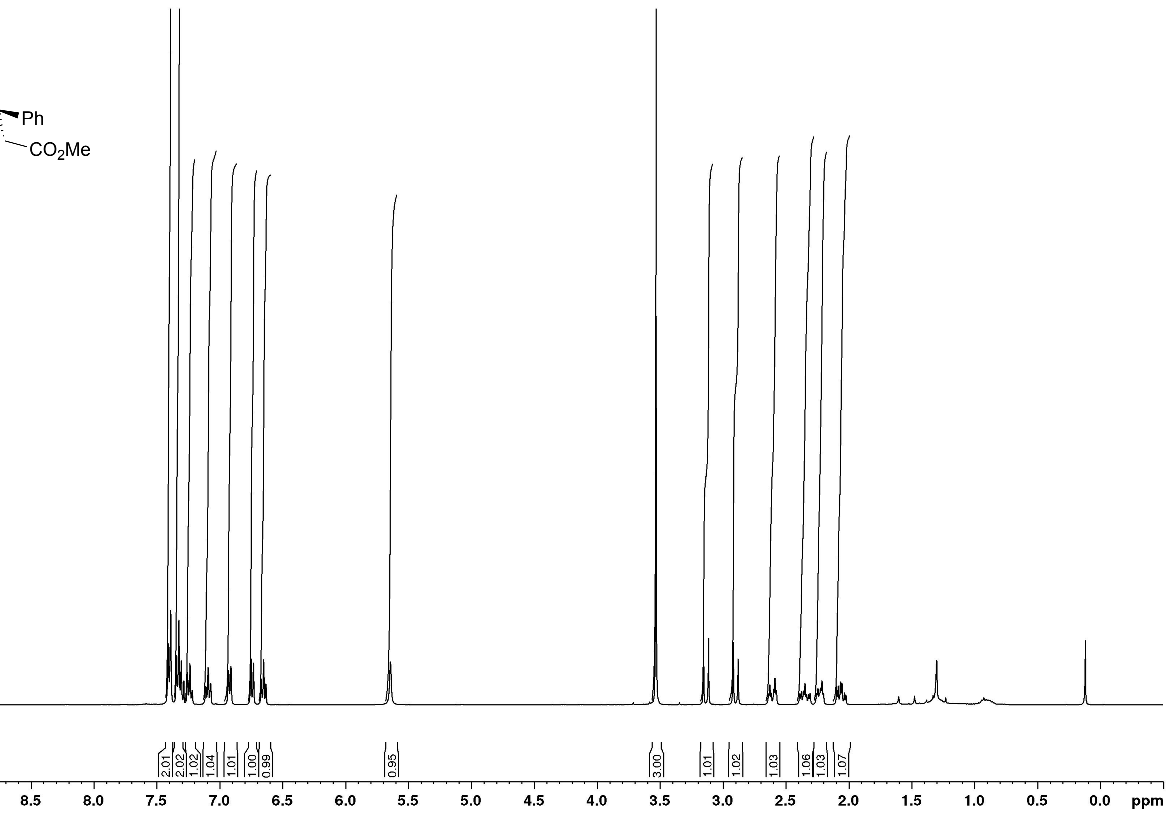
2.01 2.02 1.02 1.04 1.01 1.00 0.99 0.95 3.00 1.01 1.02 1.03 1.06 1.03 1.07
8.5 8.0 7.5 7.0 6.5 6.0 5.5 5.0 4.5 4.0 3.5 3.0 2.5 2.0 1.5 1.0 0.5 0.0 ppm

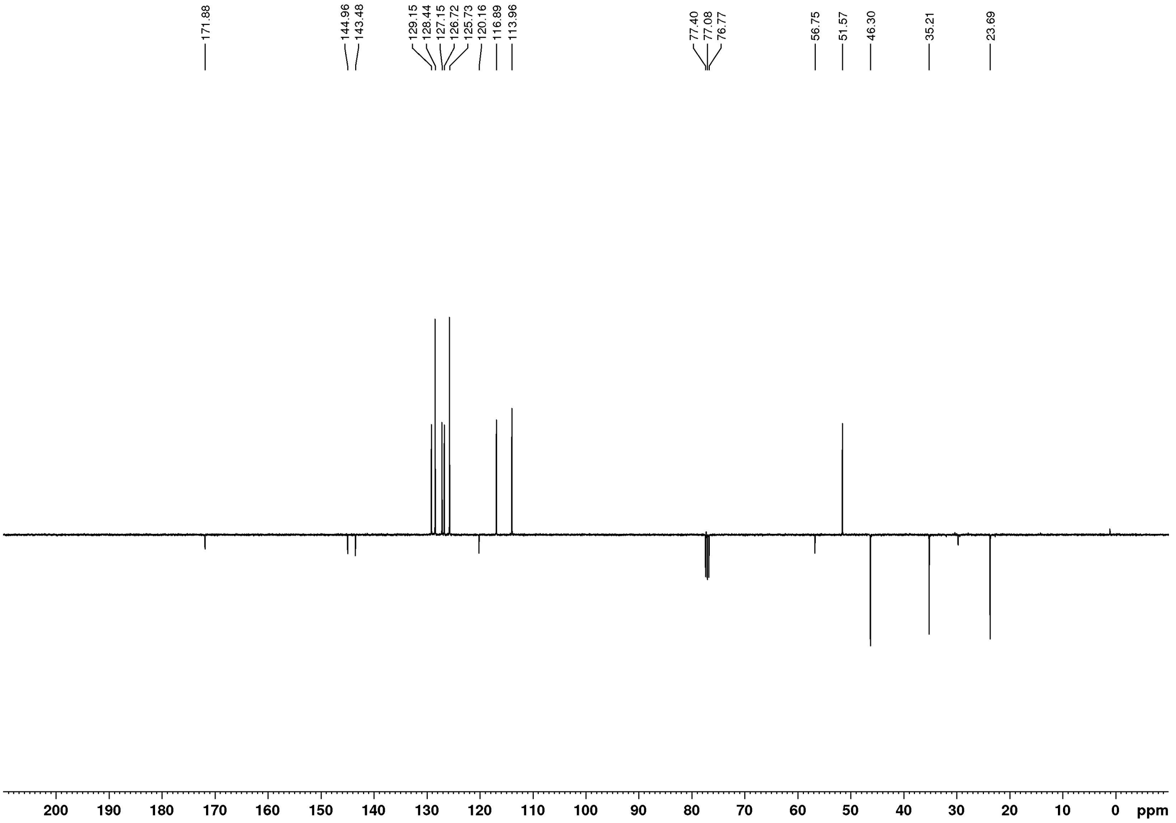
171.88
144.96
143.48
129.15
128.44
127.15
126.72
125.73
120.16
116.89
113.96
77.40
77.08
76.77
56.75
51.57
46.30
35.21
23.69
200 190 180 170 160 150 140 130 120 110 100 90 80 70 60 50 40 30 20 10 0 ppm

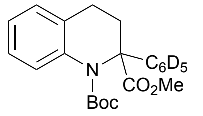
N
C6D5
CO2Me
Boc

13

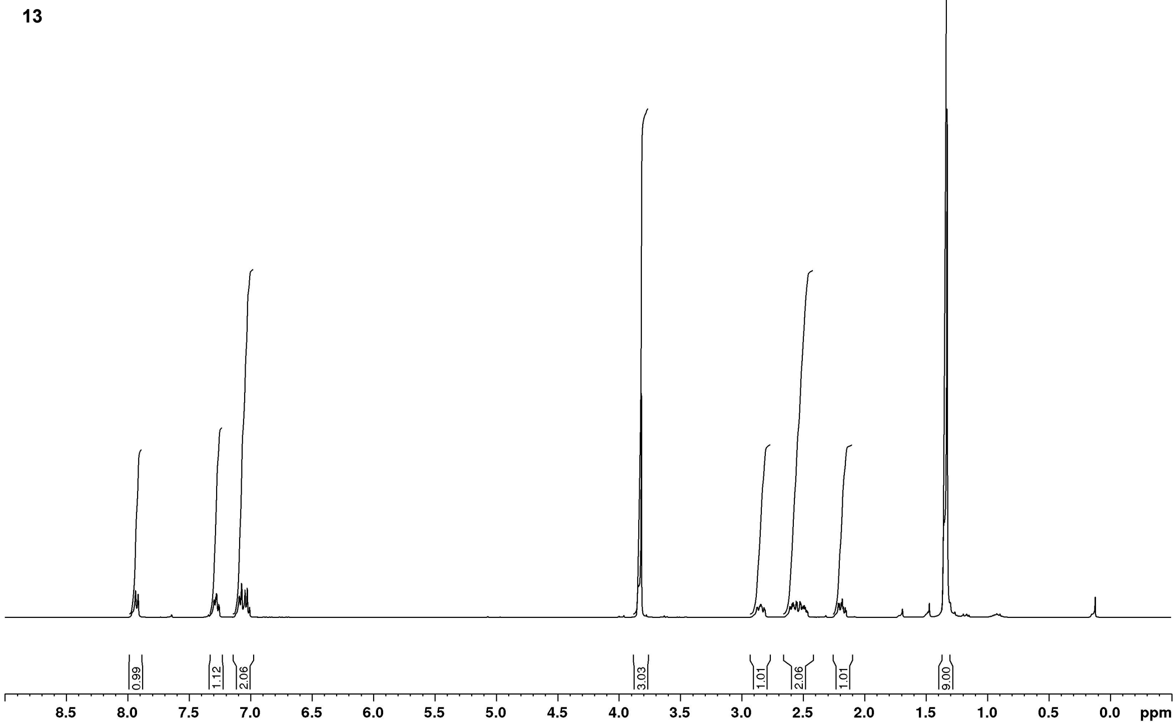
0.99
1.12
2.06
3.03
1.01
2.06
1.01
9.00
8.5 8.0 7.5 7.0 6.5 6.0 5.5 5.0 4.5 4.0 3.5 3.0 2.5 2.0 1.5 1.0 0.5 0.0 ppm

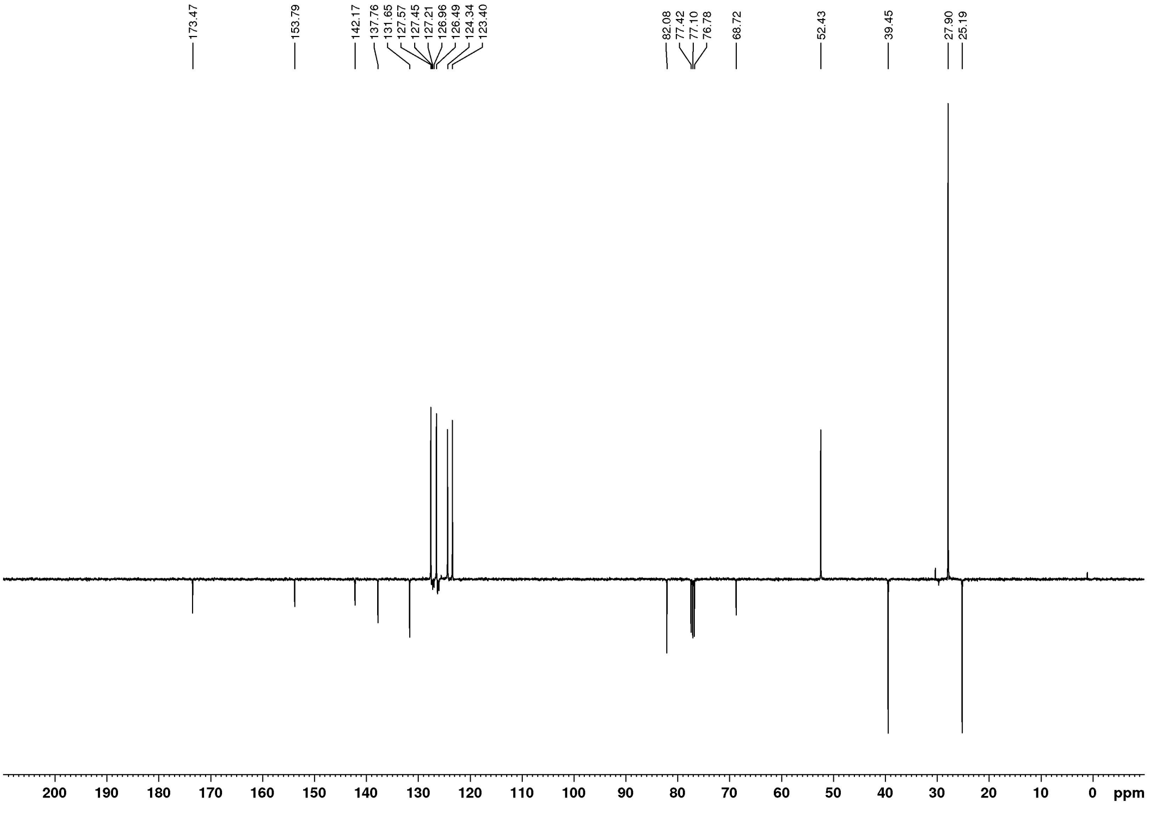
173.47
153.79
142.17
137.76
131.65
127.57
127.45
127.21
126.96
126.49
124.34
123.40
82.08
77.42
77.10
76.78
68.72
52.43
39.45
27.90
25.19
200 190 180 170 160 150 140 130 120 110 100 90 80 70 60 50 40 30 20 10 0 ppm

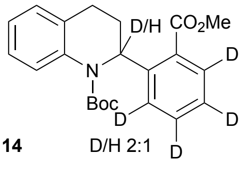
D/H
CO2Me
D
D
D
D
N
Boc

14 D/H 2:1

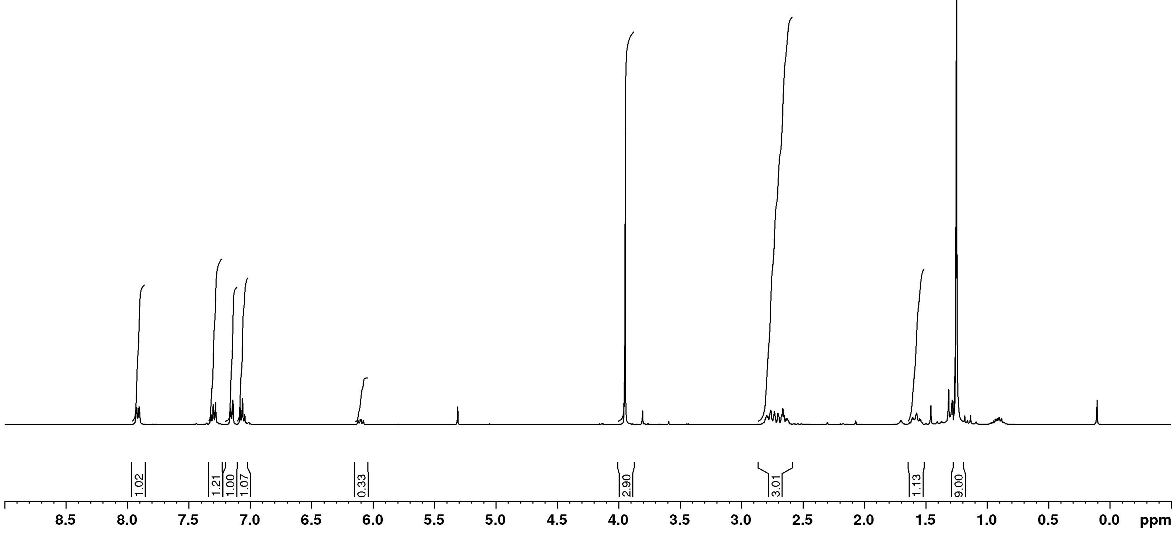
1.02
1.21
1.00
1.07
0.33
2.90
3.01
1.13
9.00
8.5 8.0 7.5 7.0 6.5 6.0 5.5 5.0 4.5 4.0 3.5 3.0 2.5 2.0 1.5 1.0 0.5 0.0 ppm

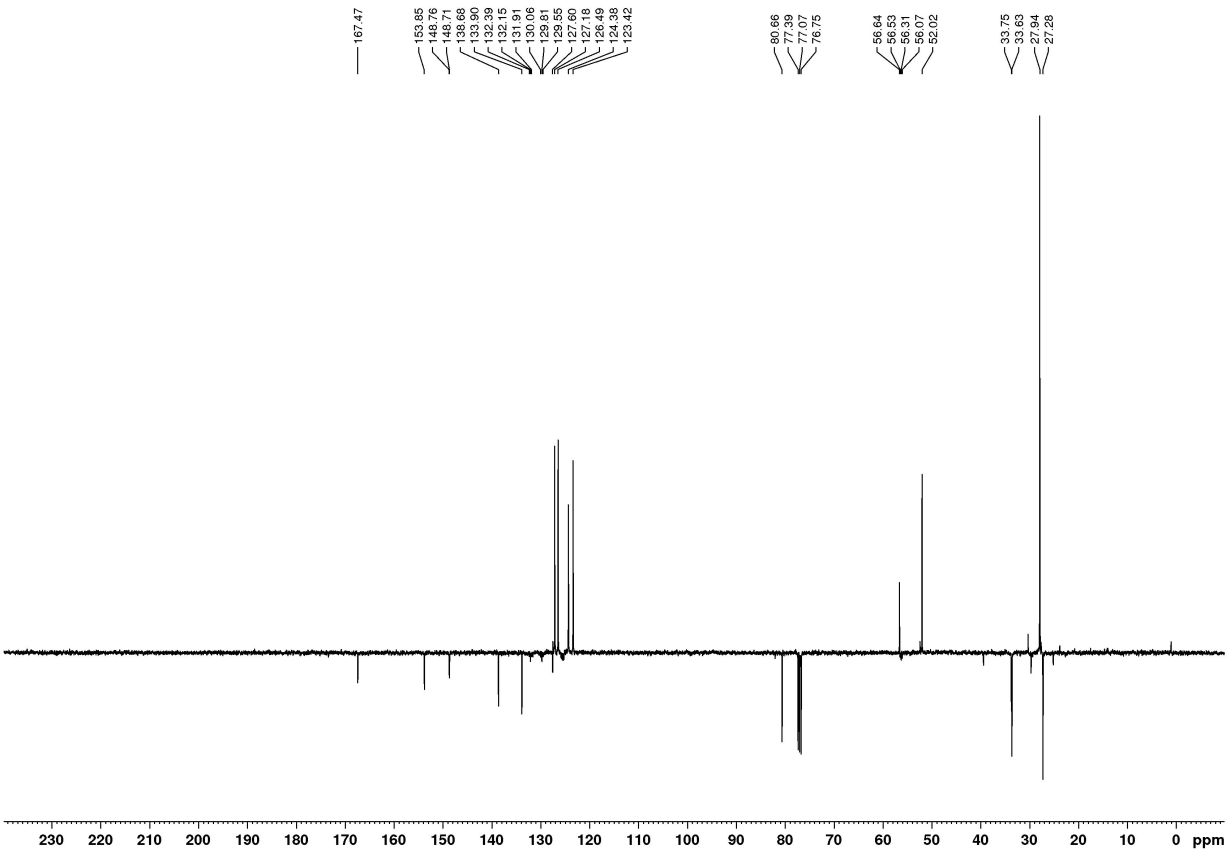
167.47
153.85
148.76
148.71
138.68
133.90
132.39
132.15
131.91
130.06
129.81
129.55
127.60
127.18
126.49
124.38
123.42
80.66
77.39
77.07
76.75
56.64
56.53
56.31
56.07
52.02
33.75
33.63
27.94
27.28
230 220 210 200 190 180 170 160 150 140 130 120 110 100 90 80 70 60 50 40 30 20 10 0 ppm

10. References

1. Z. Zhang, A. M. Olland, Y. Zhu, J. Cohen, T. Berrodin, S. Chippari, C. Appavu, S. Li, J. Wilhem, R. Chopra, A. Fensome, P. Zhang, J. Wrobel, R. J. Unwalla, C. R. Lyttle and R. C. Winneker, *J. Biol. Chem.*, 2005, **280**, 28468.
2. N. T. Patil and V. S. Raut, *J. Org. Chem.*, 2010, **75**, 6961.
3. A. D. Lackner, A. V. Samant and F. D. Toste, *J. Am. Chem. Soc.*, 2013, **135**, 14090.
4. M. Rueping, A. P. Antonchick and T. Theissmann, *Angew. Chem., Int. Ed.*, 2006, **45**, 3683.
5. Y. K. Liu, S. J. Lou, D. Q. Xu and Z. Y. Xu, *Chem. Eur. J.*, 2010, **16**, 13590.
6. X. Ji, H. Huang, Y. Li, H. Chen and H. Jiang, *Angew. Chem., Int. Ed.*, 2012, **51**, 7292.
7. B. Bortolotti, R. Leardini, D. Nanni and G. Zanardi, *Tetrahedron*, 1993, **49**, 10157.
8. Z-Y. Han, H. Xiao, X.-H. Chen and L.-Z. Gong, *J. Am. Chem. Soc.*, 2009, **131**, 9182.
9. C. S. Cho, B. T. Kim, H.-J. Choi, T.-J. Kim and S. C. Shim, *Tetrahedron*, 2003, **59**, 7997.
10. M. Tobisu, I. Hyodo and N. Chatani, *J. Am. Chem. Soc.*, 2009, **131**, 12070.
11. T. Aeyad, J. D. Williams, A. J. H. M. Meyer and I. Coldham, *Synlett*, 2017, **28**, 2765.

11. DFT data

Synthesis and Kinetic Resolution of Substituted Tetrahydroquinolines by Lithiation then Electrophilic Quench
Supplementary information
DFT Calculations

Nicholas Carter,[1] Xiabing Li,[1] Lewis Reavey,[1] Anthony J. H. M. Meijer,[1] and Iain Coldham[1]

[1] *University of Sheffield, Department of Chemistry, Sheffield S3 7HF, United Kingdom*

(Dated: November 30, 2017)

In this section of the supporting information we provide information about the calculations we performed using the methodology outlined in the main paper. The first three sections describe the different rotamers for **2a**. The next four sections describe the different stereo isomers for the lithiated species with a single electrophile [either MeOC(O)Cl or MeOC(O)CN] and a single solvent molecule. The final three sections describe the lithiated species with two coordinated molecules of electrophile [either MeOC(O)Cl or MeOC(O)CN] or solvent, respectively.

CONTENTS

S11.1. **2a**, Boc group pointing away from Phenyl S-139
S11.1.1. Cartesian Co-ordinates (XYZ format) S-139
S11.1.2. Frequencies S-140

S11.2. **2a**, TS between Boc group away and to Phenyl S-143
S11.2.1. Cartesian Co-ordinates (XYZ format) S-143
S11.2.2. Frequencies S-144

S11.3. **2a**, Boc group pointing to Phenyl S-147
S11.3.1. Cartesian Co-ordinates (XYZ format) S-147
S11.3.2. Frequencies S-148

S11.4. Lithiated intermediate from **2a** with coordinated Chloroformate and THF (1st isomer) S-151
S11.4.1. Cartesian Co-ordinates (XYZ format) S-151
S11.4.2. Frequencies S-153

S11.5. Lithiated intermediate from **2a** with coordinated Chloroformate and THF (2nd isomer) S-157
S11.5.1. Cartesian Co-ordinates (XYZ format) S-157
S11.5.2. Frequencies S-159

S11.6. Lithiated intermediate from **2a** with coordinated Cyanoformate and THF (1st isomer) S-163
S11.6.1. Cartesian Co-ordinates (XYZ format) S-163
S11.6.2. Frequencies S-165

S11.7. Lithiated intermediate from **2a** with coordinated Cyanoformate and THF (2nd isomer) S-169
S11.7.1. Cartesian Co-ordinates (XYZ format) S-169
S11.7.2. Frequencies S-171

S11.8. Lithiated intermediate from **2a** with coordinated Chloroformate S-175
S11.8.1. Cartesian Co-ordinates (XYZ format) S-175
S11.8.2. Frequencies S-177

S11.9. Lithiated intermediate from **2a** with coordinated Cyanoformate S-180
S11.9.1. Cartesian Co-ordinates (XYZ format) S-180
S11.9.2. Frequencies S-182

S11.10. Lithiated intermediate from **2a** with coordinated THF S-185
S11.10.1. Cartesian Co-ordinates (XYZ format) S-185
S11.10.2. Frequencies S-187

S11.1. 2a, BOC GROUP POINTING AWAY FROM PHENYL

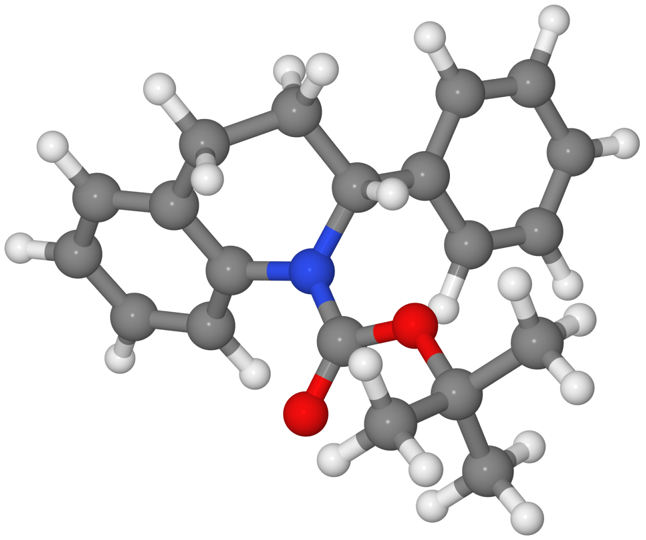

Route	: # opt freq b3lyp/6-311g(d,p) scrf=(solvent=thf) geom=connectivity empiricaldispersion=gd3bj int=ultrafine pop=(regular,mk)	
SMILES	: CC(C)(C)OC(=O)N1c2ccccc2CCC1c3ccccc3	
Formula	: $C_{20}H_{23}NO_2$	
Charge	: 0	
Multiplicity	: 1	
Dipole	: 9.1910	Debye
Energy	: -981.57965409	a.u.
Gibbs Energy	: -981.24190500	a.u.
Number of imaginary frequencies	: 0	

S11.1.1. Cartesian Co-ordinates (XYZ format)

46

C	1.58104396	3.88230610	1.34155297
C	1.93761206	2.62221694	1.81536400
C	1.45609903	1.47163904	1.19717205
C	0.62616903	1.57740402	0.07710600
C	0.25035501	2.84446597	-0.39835599
C	0.72743303	3.98355603	0.24577300
N	0.11251300	0.43614501	-0.60411602
C	-1.26039696	0.45425200	-1.18224502
C	-1.83911097	1.87672198	-1.19658506
C	-0.74404699	2.89496994	-1.52427006
C	0.88705099	-0.69609499	-0.76883000
O	2.00706410	-0.84740400	-0.31876501
O	0.22460000	-1.59950304	-1.51247501
C	0.67816401	-2.99999499	-1.61281800
C	0.75435603	-3.61177802	-0.21512000
C	2.00771189	-3.06875110	-2.36068010
C	-0.44068700	-3.64721107	-2.42399096
C	-2.18244004	-0.50479603	-0.45317999
C	-2.11920094	-0.64981902	0.93395799

C	-2.98685694	-1.51720905	1.59300697
C	-3.93340302	-2.24471211	0.87251502
C	-4.00563097	-2.09829807	-0.51143497
C	-3.13208890	-1.23416400	-1.16809201
H	1.95323098	4.77658892	1.82714105
H	2.58743310	2.52778411	2.67759800
H	1.73646605	0.49745199	1.56524599
H	0.42212701	4.95820904	-0.11955200
H	-2.26777911	2.11715698	-0.21990199
H	-2.65317512	1.89723396	-1.92391801
H	-1.15595198	3.90021396	-1.62797403
H	-0.26969299	2.63297200	-2.47796702
H	0.95640397	-4.68194008	-0.29989299
H	-0.19861300	-3.48014307	0.30262700
H	1.54648602	-3.15206909	0.37327799
H	2.26788497	-4.11469507	-2.54110003
H	1.92109799	-2.56604600	-3.32693791
H	2.80280209	-2.59803796	-1.78656006
H	-0.22428299	-4.70698500	-2.57386804
H	-0.52907097	-3.16821408	-3.40144205
H	-1.39338899	-3.55006289	-1.90088201
H	-1.37983704	-0.09348100	1.49821699
H	-2.92296004	-1.62671304	2.66957808
H	-4.60608101	-2.92225003	1.38522100
H	-4.73385715	-2.66436291	-1.08092403
H	-3.17558408	-1.13787198	-2.24770498
H	-1.16899204	0.12253500	-2.21630192

S11.1.2. Frequencies

Mode	IR frequency	IR intensity	Raman intensity
1	20.74870000	0.33460000	0.00000000
2	23.68350000	0.02710000	0.00000000
3	34.21480000	1.35250000	0.00000000
4	45.12910000	0.55480000	0.00000000
5	67.08520000	1.64340000	0.00000000
6	90.95160000	1.15710000	0.00000000
7	101.23510000	1.62680000	0.00000000
8	126.92380000	0.45430000	0.00000000
9	166.04180000	0.32150000	0.00000000
10	168.80240000	4.10410000	0.00000000
11	209.57470000	0.35240000	0.00000000
12	215.47030000	3.53170000	0.00000000
13	237.23740000	4.26530000	0.00000000
14	251.07900000	0.28650000	0.00000000
15	256.60170000	1.00270000	0.00000000
16	274.24180000	0.25840000	0.00000000
17	277.84850000	0.21760000	0.00000000
18	297.36340000	3.43250000	0.00000000
19	332.70360000	12.88520000	0.00000000
20	348.52120000	7.07700000	0.00000000
21	357.17720000	3.22120000	0.00000000
22	397.37980000	4.93640000	0.00000000
23	414.01440000	0.16260000	0.00000000
24	416.25060000	0.22670000	0.00000000
25	431.46630000	2.90010000	0.00000000
26	459.43650000	6.55250000	0.00000000
27	472.66170000	3.81170000	0.00000000
28	477.05970000	7.90930000	0.00000000
29	501.29520000	6.88470000	0.00000000
30	539.64710000	9.32130000	0.00000000
31	556.91710000	1.42300000	0.00000000

32	578.21650000	10.29290000	0.00000000
33	613.45780000	10.79260000	0.00000000
34	635.78100000	0.32200000	0.00000000
35	647.09240000	1.64410000	0.00000000
36	703.81660000	11.56030000	0.00000000
37	715.85320000	69.12920000	0.00000000
38	745.62140000	7.78110000	0.00000000
39	750.68200000	36.49150000	0.00000000
40	768.63530000	11.84760000	0.00000000
41	771.48170000	34.44190000	0.00000000
42	780.40420000	59.88660000	0.00000000
43	796.04570000	19.52880000	0.00000000
44	823.94910000	3.85490000	0.00000000
45	841.21460000	29.90230000	0.00000000
46	859.23650000	0.50050000	0.00000000
47	862.57010000	19.95500000	0.00000000
48	876.54090000	0.61570000	0.00000000
49	917.53200000	12.22810000	0.00000000
50	928.42870000	2.01190000	0.00000000
51	929.24290000	0.29720000	0.00000000
52	933.37990000	4.07600000	0.00000000
53	950.24520000	20.95450000	0.00000000
54	953.96310000	2.02700000	0.00000000
55	972.40410000	0.20930000	0.00000000
56	985.80120000	0.14030000	0.00000000
57	988.35670000	0.05790000	0.00000000
58	1004.43980000	0.19020000	0.00000000
59	1019.88560000	4.11280000	0.00000000
60	1023.62970000	46.13460000	0.00000000
61	1046.98410000	59.45930000	0.00000000
62	1050.70560000	6.50190000	0.00000000
63	1052.27440000	0.95990000	0.00000000
64	1059.47360000	15.54830000	0.00000000
65	1069.03650000	40.29220000	0.00000000
66	1073.91150000	0.10880000	0.00000000
67	1104.07570000	33.62840000	0.00000000
68	1134.51060000	26.25680000	0.00000000
69	1153.12380000	283.09810000	0.00000000
70	1176.78600000	13.96590000	0.00000000
71	1180.29880000	2.94290000	0.00000000
72	1181.76610000	458.31670000	0.00000000
73	1195.34430000	73.88500000	0.00000000
74	1199.36360000	21.92880000	0.00000000
75	1213.57740000	1.25360000	0.00000000
76	1222.84460000	4.64170000	0.00000000
77	1234.90420000	18.53430000	0.00000000
78	1260.33030000	106.00190000	0.00000000
79	1270.11040000	20.09890000	0.00000000
80	1282.75270000	40.31240000	0.00000000
81	1305.19520000	4.18810000	0.00000000
82	1318.74150000	4.94750000	0.00000000
83	1336.83940000	33.70720000	0.00000000
84	1341.64930000	77.23350000	0.00000000
85	1352.53360000	620.19080000	0.00000000
86	1354.15300000	6.36850000	0.00000000
87	1361.80150000	2.68770000	0.00000000
88	1386.42310000	137.40740000	0.00000000
89	1394.56500000	30.30930000	0.00000000
90	1398.27160000	48.88870000	0.00000000
91	1407.47470000	39.97870000	0.00000000
92	1421.28490000	23.12640000	0.00000000
93	1466.92820000	0.38720000	0.00000000
94	1478.30800000	13.11620000	0.00000000
95	1484.72210000	4.14990000	0.00000000

96	1485.80300000	15.20780000	0.00000000
97	1487.20770000	6.56180000	0.00000000
98	1489.25600000	15.25660000	0.00000000
99	1491.96040000	7.39910000	0.00000000
100	1493.10490000	20.31650000	0.00000000
101	1499.11100000	2.25410000	0.00000000
102	1519.61800000	22.46330000	0.00000000
103	1524.36390000	124.73600000	0.00000000
104	1528.20270000	16.13520000	0.00000000
105	1623.68560000	8.19890000	0.00000000
106	1629.02810000	0.88250000	0.00000000
107	1644.56440000	17.64780000	0.00000000
108	1646.75610000	10.84950000	0.00000000
109	1735.43250000	550.73700000	0.00000000
110	3012.23150000	40.98450000	0.00000000
111	3036.87570000	12.53510000	0.00000000
112	3038.64890000	37.20660000	0.00000000
113	3044.33430000	29.95430000	0.00000000
114	3045.29920000	24.53960000	0.00000000
115	3076.09340000	4.24760000	0.00000000
116	3086.94150000	43.55050000	0.00000000
117	3095.74570000	61.52550000	0.00000000
118	3099.27760000	15.83420000	0.00000000
119	3104.56200000	20.29920000	0.00000000
120	3110.37140000	60.80610000	0.00000000
121	3118.95800000	44.30770000	0.00000000
122	3138.41960000	10.85490000	0.00000000
123	3146.43500000	18.06820000	0.00000000
124	3158.88380000	8.40560000	0.00000000
125	3161.30500000	8.86490000	0.00000000
126	3165.59110000	0.21040000	0.00000000
127	3172.36420000	23.10880000	0.00000000
128	3174.16130000	20.12280000	0.00000000
129	3181.64860000	41.05600000	0.00000000
130	3188.61240000	40.89790000	0.00000000
131	3191.16490000	23.68940000	0.00000000
132	3236.97300000	2.05350000	0.00000000

S11.2. 2a, TS BETWEEN BOC GROUP AWAY AND TO PHENYL

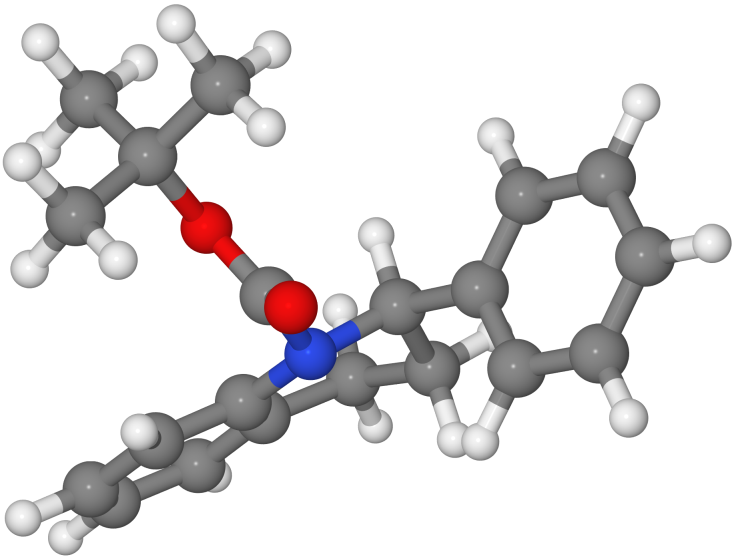

Route	: # opt=(calcfc,qst3) freq b3lyp/6-311g(d,p) scrf=(solvent=thf) geom=connectivity empiricaldispersion=gd3bj int=ultrafine pop=(regular,mk)	
SMILES	: CC(C)(C)OC(=O)N1c2ccccc2CCC1c3ccccc3	
Formula	: $C_{20}H_{23}NO_2$	
Charge	: 0	
Multiplicity	: 1	
Dipole	: 7.5805	Debye
Energy	: -981.56168593	a.u.
Gibbs Energy	: -981.22334300	a.u.
Number of imaginary frequencies	: 1	

S11.2.1. Cartesian Co-ordinates (XYZ format)

```
46

C   2.33552194  3.93180394  0.48271501
C   2.74672389  2.68653297  0.95692998
C   1.92991197  1.57572103  0.79894698
C   0.69394702  1.67927694  0.14129600
C   0.26942599  2.93201303 -0.33316901
C   1.09880495  4.03925323 -0.14416599
N  -0.11972400  0.52800697  0.02959000
C  -1.37445104  0.59254402 -0.75887001
C  -2.02104497  1.96635604 -0.56586403
C  -1.06919897  3.06974506 -1.01720703
C   0.56685799 -0.71815097 -0.13731100
O   0.41398299 -1.66380799  0.59297299
O   1.34507704 -0.67348599 -1.21999002
C   2.20999408 -1.81601405 -1.61403501
C   1.33507299 -3.01963592 -1.95349002
C   3.21054506 -2.10679293 -0.49871799
C   2.91349196 -1.27736104 -2.85454988
C  -2.31787801 -0.52668703 -0.37234199
```

C	-2.67171407	-0.73607302	0.96325701
C	-3.57960010	-1.73242497	1.30263603
C	-4.14411783	-2.53568697	0.31009099
C	-3.79164100	-2.33666801	-1.02152801
C	-2.88008094	-1.33587003	-1.35850596
H	2.96304107	4.80542517	0.61077899
H	3.69893789	2.57953691	1.46339297
H	2.24304104	0.61859399	1.19913101
H	0.75913101	5.00469780	-0.50541300
H	-2.27863908	2.09364700	0.48965800
H	-2.95061707	1.99012804	-1.13769603
H	-1.49469900	4.05309391	-0.80215001
H	-0.93997598	3.01667309	-2.10501003
H	1.96209598	-3.81542397	-2.36224508
H	0.59320098	-2.74613595	-2.70731807
H	0.82119697	-3.39506292	-1.07076001
H	3.92605090	-2.85506105	-0.84666300
H	3.76264691	-1.20023704	-0.24076800
H	2.71342492	-2.48839808	0.39120999
H	3.59251809	-2.03370690	-3.25312710
H	3.48996401	-0.38367000	-2.60814500
H	2.18481708	-1.02127099	-3.62622190
H	-2.21396804	-0.12847300	1.73423004
H	-3.84441710	-1.88918602	2.34199095
H	-4.84891701	-3.31474495	0.57652301
H	-4.21919298	-2.96094894	-1.79758000
H	-2.60060310	-1.18477201	-2.39581800
H	-1.13058102	0.48141199	-1.82468903

S11.2.2. Frequencies

Mode	IR frequency	IR intensity	Raman intensity
1	-26.89060000	1.43020000	0.00000000
2	22.29020000	0.32750000	0.00000000
3	33.45220000	0.18200000	0.00000000
4	44.15500000	0.69710000	0.00000000
5	50.98530000	1.38770000	0.00000000
6	71.31290000	0.79360000	0.00000000
7	109.26020000	2.13690000	0.00000000
8	136.38180000	1.60400000	0.00000000
9	153.25360000	1.34910000	0.00000000
10	175.53900000	4.75380000	0.00000000
11	187.21900000	2.02820000	0.00000000
12	204.24170000	0.26760000	0.00000000
13	226.10770000	4.23140000	0.00000000
14	247.32390000	0.54360000	0.00000000
15	253.55030000	0.15110000	0.00000000
16	267.40770000	2.16380000	0.00000000
17	269.68280000	0.10680000	0.00000000
18	288.83710000	1.19260000	0.00000000
19	321.65320000	8.46620000	0.00000000
20	354.49900000	2.28340000	0.00000000
21	358.14600000	3.87650000	0.00000000
22	387.18360000	1.93950000	0.00000000
23	403.19590000	5.81520000	0.00000000
24	416.14900000	0.40890000	0.00000000
25	433.96230000	1.40290000	0.00000000
26	462.05240000	14.22680000	0.00000000
27	469.81600000	7.35540000	0.00000000
28	486.52620000	1.14660000	0.00000000
29	500.33860000	14.95930000	0.00000000
30	537.32140000	9.19330000	0.00000000

31	554.18700000	9.77820000	0.00000000
32	565.10950000	0.61940000	0.00000000
33	614.70950000	1.84760000	0.00000000
34	635.81910000	0.75620000	0.00000000
35	650.05510000	8.73460000	0.00000000
36	677.84860000	4.41140000	0.00000000
37	714.26300000	62.41720000	0.00000000
38	732.89810000	14.94910000	0.00000000
39	758.50720000	68.18770000	0.00000000
40	760.26690000	15.63470000	0.00000000
41	775.40890000	44.63900000	0.00000000
42	783.38530000	15.15730000	0.00000000
43	803.10310000	3.75430000	0.00000000
44	827.59350000	14.85260000	0.00000000
45	854.02120000	34.14730000	0.00000000
46	855.75280000	32.00790000	0.00000000
47	858.77770000	5.45720000	0.00000000
48	868.07420000	0.75310000	0.00000000
49	907.42890000	2.77570000	0.00000000
50	929.08040000	6.67810000	0.00000000
51	931.16500000	0.26700000	0.00000000
52	932.79710000	0.16250000	0.00000000
53	949.68130000	3.07920000	0.00000000
54	963.34790000	5.82550000	0.00000000
55	972.22950000	0.19540000	0.00000000
56	981.53220000	0.07900000	0.00000000
57	986.47330000	0.15940000	0.00000000
58	1006.80770000	1.95470000	0.00000000
59	1016.08340000	36.65940000	0.00000000
60	1021.47820000	6.27740000	0.00000000
61	1038.92300000	11.80990000	0.00000000
62	1049.57290000	7.13620000	0.00000000
63	1051.79010000	0.78590000	0.00000000
64	1059.06710000	0.86960000	0.00000000
65	1068.23020000	19.09850000	0.00000000
66	1077.18450000	11.39500000	0.00000000
67	1104.02600000	18.86920000	0.00000000
68	1119.74620000	66.86910000	0.00000000
69	1140.34410000	297.26020000	0.00000000
70	1164.01170000	570.49840000	0.00000000
71	1176.81300000	0.39090000	0.00000000
72	1182.16480000	21.88560000	0.00000000
73	1193.43020000	15.23020000	0.00000000
74	1197.27980000	9.67960000	0.00000000
75	1217.17030000	1.20530000	0.00000000
76	1225.32650000	4.27110000	0.00000000
77	1240.43090000	74.08130000	0.00000000
78	1248.49310000	194.08910000	0.00000000
79	1271.89250000	32.83100000	0.00000000
80	1284.69740000	16.54940000	0.00000000
81	1295.78110000	195.24400000	0.00000000
82	1309.48220000	97.42370000	0.00000000
83	1320.18980000	20.96950000	0.00000000
84	1338.99530000	66.26380000	0.00000000
85	1347.04320000	7.34010000	0.00000000
86	1350.82040000	2.79050000	0.00000000
87	1374.48190000	11.13860000	0.00000000
88	1382.07920000	5.13830000	0.00000000
89	1397.18290000	27.01670000	0.00000000
90	1399.72820000	21.18910000	0.00000000
91	1401.14900000	29.90330000	0.00000000
92	1422.36030000	20.01150000	0.00000000
93	1467.52680000	0.41850000	0.00000000
94	1475.07400000	13.07250000	0.00000000

95	1482.41690000	12.05630000	0.00000000
96	1485.22250000	1.95630000	0.00000000
97	1485.32360000	1.61220000	0.00000000
98	1486.98850000	36.80970000	0.00000000
99	1489.15820000	24.68290000	0.00000000
100	1491.10870000	9.05100000	0.00000000
101	1498.83910000	1.90220000	0.00000000
102	1519.56740000	21.57640000	0.00000000
103	1527.44540000	36.27140000	0.00000000
104	1528.34820000	120.50750000	0.00000000
105	1619.62560000	65.21390000	0.00000000
106	1629.88570000	1.11350000	0.00000000
107	1646.48670000	49.84060000	0.00000000
108	1647.74390000	5.87480000	0.00000000
109	1780.38830000	544.09140000	0.00000000
110	2976.58000000	46.07240000	0.00000000
111	3009.74480000	44.13780000	0.00000000
112	3038.56510000	13.77420000	0.00000000
113	3041.40170000	33.28090000	0.00000000
114	3043.26990000	30.49710000	0.00000000
115	3047.25660000	15.89190000	0.00000000
116	3067.55890000	48.99490000	0.00000000
117	3093.85990000	40.57400000	0.00000000
118	3103.73560000	10.54510000	0.00000000
119	3106.03100000	20.18890000	0.00000000
120	3112.13820000	45.01950000	0.00000000
121	3116.54130000	62.61980000	0.00000000
122	3140.01600000	5.30450000	0.00000000
123	3143.94430000	21.24220000	0.00000000
124	3155.78840000	18.17590000	0.00000000
125	3158.51530000	9.55950000	0.00000000
126	3166.46210000	0.42430000	0.00000000
127	3167.44180000	7.75240000	0.00000000
128	3175.89490000	29.59680000	0.00000000
129	3179.53520000	33.02180000	0.00000000
130	3185.64250000	43.88140000	0.00000000
131	3191.68990000	30.48990000	0.00000000
132	3192.76850000	14.43210000	0.00000000

S11.3. 2a, BOC GROUP POINTING TO PHENYL

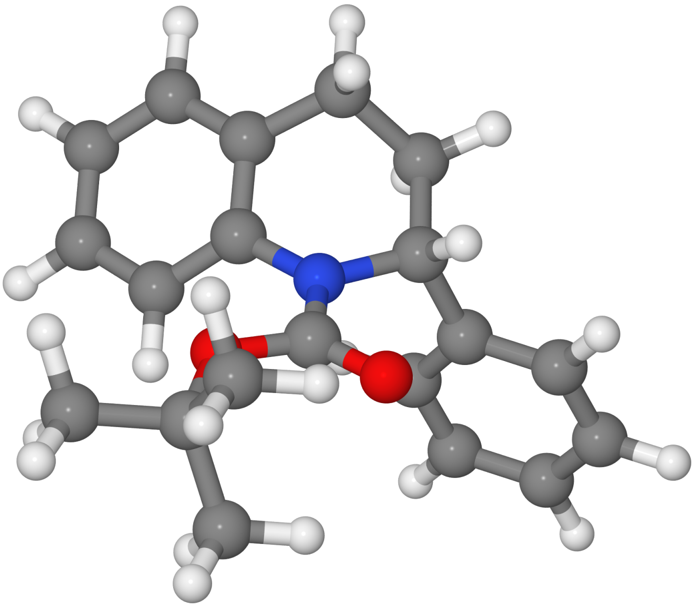

Route	: # opt freq b3lyp/6-311g(d,p) scrf=(solvent=thf) geom=connectivity empiricaldispersion=gd3bj int=ultrafine pop=(regular,mk)	
SMILES	: CC(C)(C)OC(=O)N1c2ccccc2CCC1c3ccccc3	
Formula	: $C_{20}H_{23}NO_2$	
Charge	: 0	
Multiplicity	: 1	
Dipole	: 4.8944	Debye
Energy	: -981.57837288	a.u.
Gibbs Energy	: -981.24209400	a.u.
Number of imaginary frequencies	: 0	

S11.3.1. Cartesian Co-ordinates (XYZ format)

```
46

C   1.44592595  3.84447098  1.39000905
C   1.66128397  2.61798096  2.01641893
C   1.19952095  1.44068301  1.43597901
C   0.54170901  1.48535097  0.20494799
C   0.30520299  2.71642208 -0.42461699
C   0.75604302  3.88761497  0.18073399
N   0.04188000  0.32038099 -0.44322500
C  -1.29048204  0.34786901 -1.10160506
C  -1.73722994  1.79296100 -1.38767099
C  -0.52670902  2.68932390 -1.67636895
C   0.75011498 -0.85074699 -0.60641903
O   0.28426600 -1.82422805 -1.17194700
O   1.98832202 -0.76325798 -0.09778700
C   2.91651893 -1.91096199 -0.13307200
C   3.23844790 -2.27500892 -1.58126998
C   2.32652903 -3.08322191  0.64854598
C   4.14882898 -1.35289502  0.57312399
```

C	-2.32401490	-0.40746701	-0.28893200
C	-2.45650101	-0.17926200	1.08380997
C	-3.42860603	-0.84974301	1.82037497
C	-4.28567314	-1.75320005	1.19167399
C	-4.15898085	-1.98467898	-0.17594700
C	-3.17945504	-1.31696606	-0.90927100
H	1.80068803	4.76077414	1.84673703
H	2.18045998	2.57532191	2.96672702
H	1.35896802	0.49031401	1.92388701
H	0.56223601	4.83886909	-0.30306399
H	-2.27369499	2.19613004	-0.52486002
H	-2.43899012	1.77001500	-2.22385693
H	-0.83981901	3.69681692	-1.95523500
H	0.04652100	2.27636099	-2.51520300
H	4.02524805	-3.03301692	-1.59716904
H	3.59978604	-1.39474106	-2.11822796
H	2.36023593	-2.66649508	-2.09037495
H	3.07260203	-3.87778902	0.72378498
H	2.06117392	-2.76630712	1.65999699
H	1.44006395	-3.47677994	0.15557501
H	4.93203306	-2.11283493	0.60900402
H	3.90301609	-1.05603099	1.59465301
H	4.53136110	-0.48039100	0.04003600
H	-1.79163599	0.51960301	1.57848501
H	-3.51834393	-0.66796798	2.88532090
H	-5.04232693	-2.27563095	1.76538002
H	-4.81499100	-2.69166899	-0.67080498
H	-3.06935191	-1.51179695	-1.97046304
H	-1.16180301	-0.16777600	-2.05295300

S11.3.2. Frequencies

Mode	IR frequency	IR intensity	Raman intensity
1	11.58310000	0.20930000	0.00000000
2	18.68490000	0.57000000	0.00000000
3	26.22490000	0.20780000	0.00000000
4	35.57710000	0.57800000	0.00000000
5	66.28910000	2.01350000	0.00000000
6	80.26720000	0.52330000	0.00000000
7	107.69510000	1.41270000	0.00000000
8	131.76650000	1.24050000	0.00000000
9	163.75600000	1.51100000	0.00000000
10	167.75530000	2.93100000	0.00000000
11	207.21260000	0.33940000	0.00000000
12	215.16890000	0.85470000	0.00000000
13	227.33460000	6.56570000	0.00000000
14	240.39490000	1.30660000	0.00000000
15	247.37610000	0.17750000	0.00000000
16	271.31590000	0.09970000	0.00000000
17	279.74030000	0.18360000	0.00000000
18	313.15810000	4.84980000	0.00000000
19	328.68970000	11.31270000	0.00000000
20	351.02460000	2.42180000	0.00000000
21	366.26170000	2.65900000	0.00000000
22	391.60920000	8.06820000	0.00000000
23	411.31010000	1.61340000	0.00000000
24	413.70560000	0.47000000	0.00000000
25	430.32450000	3.48960000	0.00000000
26	458.54710000	4.28960000	0.00000000
27	472.16970000	3.06320000	0.00000000
28	477.78600000	12.96580000	0.00000000
29	504.98440000	9.78230000	0.00000000

30	546.05590000	19.15740000	0.00000000
31	556.02220000	0.68850000	0.00000000
32	570.74920000	7.71530000	0.00000000
33	625.31120000	4.95890000	0.00000000
34	638.04810000	1.91520000	0.00000000
35	647.34240000	4.74240000	0.00000000
36	693.68390000	7.35140000	0.00000000
37	714.90240000	64.07870000	0.00000000
38	752.13200000	57.89880000	0.00000000
39	757.46950000	6.75190000	0.00000000
40	770.12620000	16.79070000	0.00000000
41	772.50580000	37.46440000	0.00000000
42	782.67130000	44.32070000	0.00000000
43	794.13310000	7.68010000	0.00000000
44	821.33780000	6.61540000	0.00000000
45	841.92320000	2.88740000	0.00000000
46	857.15820000	0.22690000	0.00000000
47	869.19500000	67.42940000	0.00000000
48	879.29570000	2.27210000	0.00000000
49	911.15200000	12.75190000	0.00000000
50	928.35120000	0.18330000	0.00000000
51	929.06000000	3.98300000	0.00000000
52	930.92710000	0.20930000	0.00000000
53	945.32630000	11.33710000	0.00000000
54	957.33520000	3.00240000	0.00000000
55	971.57040000	0.23430000	0.00000000
56	983.57670000	0.14280000	0.00000000
57	989.98300000	0.00470000	0.00000000
58	1004.18410000	0.35570000	0.00000000
59	1019.56490000	0.18250000	0.00000000
60	1025.99040000	9.40710000	0.00000000
61	1041.97780000	83.53590000	0.00000000
62	1050.78110000	9.14730000	0.00000000
63	1052.56870000	0.54480000	0.00000000
64	1057.78930000	4.82150000	0.00000000
65	1066.27060000	9.56140000	0.00000000
66	1069.25130000	48.18410000	0.00000000
67	1105.00520000	16.86470000	0.00000000
68	1135.97250000	56.45240000	0.00000000
69	1143.76430000	101.68400000	0.00000000
70	1176.94680000	0.91850000	0.00000000
71	1179.49230000	1.51930000	0.00000000
72	1186.49170000	458.10130000	0.00000000
73	1193.95710000	50.40380000	0.00000000
74	1199.78670000	6.50130000	0.00000000
75	1213.80960000	5.72580000	0.00000000
76	1222.56830000	7.26170000	0.00000000
77	1234.84400000	20.80580000	0.00000000
78	1268.97400000	23.22830000	0.00000000
79	1276.23100000	197.88090000	0.00000000
80	1283.68970000	29.26000000	0.00000000
81	1302.07260000	3.05790000	0.00000000
82	1316.65250000	79.33130000	0.00000000
83	1337.27250000	14.90990000	0.00000000
84	1341.18290000	88.51140000	0.00000000
85	1354.26530000	44.01240000	0.00000000
86	1358.85700000	508.93480000	0.00000000
87	1360.47710000	6.65800000	0.00000000
88	1384.24770000	67.53290000	0.00000000
89	1394.83130000	31.23290000	0.00000000
90	1398.00290000	56.45610000	0.00000000
91	1417.39030000	32.62130000	0.00000000
92	1420.89580000	13.08740000	0.00000000
93	1466.92810000	0.45030000	0.00000000

94	1478.29110000	10.96050000	0.00000000
95	1484.45670000	2.63280000	0.00000000
96	1485.58860000	0.17260000	0.00000000
97	1487.15300000	4.80410000	0.00000000
98	1488.19890000	42.09070000	0.00000000
99	1494.06580000	13.99930000	0.00000000
100	1494.64130000	15.73290000	0.00000000
101	1499.16870000	1.79290000	0.00000000
102	1518.93770000	21.74520000	0.00000000
103	1523.30890000	135.78700000	0.00000000
104	1528.49860000	20.10550000	0.00000000
105	1622.73020000	29.76510000	0.00000000
106	1628.93400000	1.97750000	0.00000000
107	1646.49860000	25.18730000	0.00000000
108	1646.85400000	13.01160000	0.00000000
109	1724.70250000	667.69180000	0.00000000
110	3014.31070000	40.02440000	0.00000000
111	3037.32770000	15.88130000	0.00000000
112	3038.15180000	34.60350000	0.00000000
113	3045.42930000	26.34620000	0.00000000
114	3046.24300000	28.53870000	0.00000000
115	3079.51190000	2.74600000	0.00000000
116	3088.91270000	44.73240000	0.00000000
117	3097.49050000	57.17880000	0.00000000
118	3099.91120000	10.63980000	0.00000000
119	3101.99580000	32.02130000	0.00000000
120	3110.86680000	47.06160000	0.00000000
121	3115.23910000	66.80430000	0.00000000
122	3143.08510000	0.88320000	0.00000000
123	3146.09250000	25.52490000	0.00000000
124	3160.12150000	6.95940000	0.00000000
125	3162.73330000	7.72360000	0.00000000
126	3165.01320000	0.37950000	0.00000000
127	3173.27450000	19.53790000	0.00000000
128	3173.56430000	21.07100000	0.00000000
129	3180.58690000	47.40630000	0.00000000
130	3188.86540000	39.96430000	0.00000000
131	3190.48930000	24.59870000	0.00000000
132	3219.50720000	6.60310000	0.00000000

S11.4. LITHIATED INTERMEDIATE FROM 2a WITH COORDINATED CHLOROFORMATE AND THF (1st ISOMER)

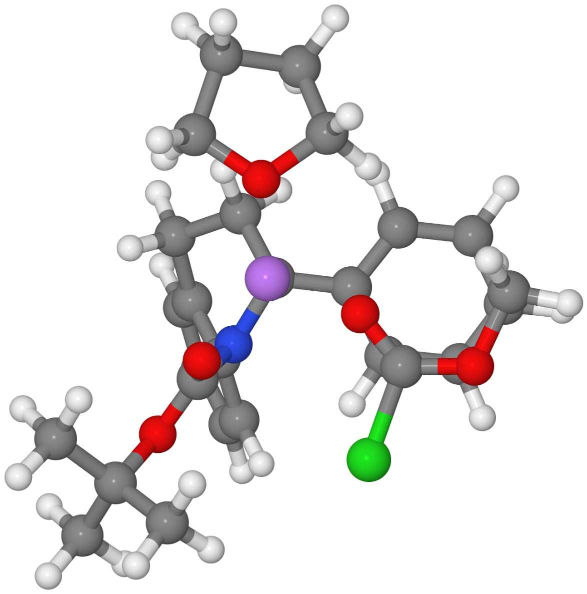

Route	: # opt freq b3lyp/6-311g(d,p) scrf=(solvent=thf) geom=connectivity empi
	: ricaldispersion=gd3bj int=ultrafine pop=(regular,mk)
SMILES	: [Li].CC(C)(C)OC(=O)N1c2ccccc2CC[C]1c3ccccc3.COC(=O)Cl.C1CCOC1
Formula	: $C_{26}H_{33}ClLiNO_5$
Charge	: 0
Multiplicity	: 1
Dipole	: 18.0608 Debye
Energy	: -1909.89325350 a.u.
Gibbs Energy	: -1909.41809300 a.u.
Number of imaginary frequencies	: 0

S11.4.1. Cartesian Co-ordinates (XYZ format)

67

C	-4.07383013	2.66007996	-2.30114508
C	-3.94841290	1.29754901	-2.56836295
C	-3.04176903	0.52119201	-1.84725201
C	-2.27803612	1.11972296	-0.85069799
C	-2.39692307	2.48757601	-0.56652302
C	-3.29442501	3.25304389	-1.30576003
N	-1.25627196	0.44973800	-0.10966800
C	0.06172200	1.10441196	-0.15597200
C	-0.03283300	2.58138204	0.24463400
C	-1.49518001	3.00662303	0.51836902
C	-1.51689100	-0.51371503	0.80501002
O	-0.65612501	-0.97798300	1.56506300
O	-2.79455590	-0.91212499	0.79110402
C	-3.28969097	-1.99696100	1.66164601
C	-3.15724897	-1.59054899	3.12814999
C	-2.55345893	-3.29722905	1.34519601
C	-4.75780106	-2.09102392	1.25549901

C	0.83341402	0.76648498	-1.32210803
Li	1.10852802	-0.23281500	1.31437898
C	0.56074703	-0.37931100	-2.12364411
C	1.34992802	-0.72311199	-3.21091008
C	2.45817900	0.04750000	-3.57888794
C	2.75129795	1.17750895	-2.81072807
C	1.97259402	1.52886498	-1.71484101
H	-4.77399302	3.26148605	-2.86949611
H	-4.55025578	0.83881903	-3.34449697
H	-2.93142700	-0.53661799	-2.04708505
H	-3.38906789	4.31408024	-1.09948599
H	0.34783000	3.21153092	-0.57064402
H	0.56688100	2.83807707	1.12628400
H	-1.57307506	4.09198380	0.61091697
H	-1.81348002	2.57629991	1.47621500
H	-3.65694094	-2.33277392	3.75536799
H	-3.63704300	-0.62310702	3.29441190
H	-2.11200595	-1.52495694	3.42316699
H	-3.02357101	-4.11961889	1.88979900
H	-2.61600590	-3.51438093	0.27616999
H	-1.50606096	-3.23460507	1.63148606
H	-5.25581503	-2.86787391	1.83914804
H	-4.84518909	-2.33869505	0.19583200
H	-5.26422215	-1.14023304	1.43273997
H	-0.29239899	-0.99675900	-1.87335896
H	1.09508002	-1.60865903	-3.78534389
H	3.07170701	-0.22584100	-4.42894077
H	3.60539889	1.79726100	-3.06840110
H	2.24768710	2.40609908	-1.14149404
C	2.23067904	-2.44876099	-0.37994999
O	2.26373005	-1.55964506	0.42416701
O	3.14856100	-2.77210903	-1.24940503
C	4.30939817	-1.88432395	-1.28820002
H	3.97843289	-0.88816297	-1.57230794
H	4.79372501	-1.88204896	-0.31330201
H	4.95514011	-2.31343389	-2.04731798
O	2.42474508	0.69815600	2.39082909
C	3.57588506	1.20917296	1.67329895
C	2.02230906	1.63864696	3.41977596
C	3.56453300	2.70981693	1.92799497
H	4.47979212	0.73906201	2.07476306
H	3.46210599	0.93583000	0.62475097
C	3.03693700	2.78459001	3.36831903
H	1.00908601	1.97689795	3.18689489
H	2.01029801	1.11884403	4.37925911
H	2.87590909	3.20330596	1.23844600
H	4.55188513	3.15813303	1.81120098
H	2.58072996	3.74561596	3.60748911
H	3.84977293	2.60665202	4.07663822
Cl	0.86999100	-3.57337499	-0.48684001

S11.4.2. Frequencies

Mode	IR frequency	IR intensity	Raman intensity
1	4.42270000	0.80260000	0.00000000
2	16.33360000	1.12960000	0.00000000
3	22.75280000	0.55600000	0.00000000
4	27.78000000	1.45600000	0.00000000
5	34.50340000	0.80450000	0.00000000
6	37.35250000	0.69840000	0.00000000
7	39.26580000	1.32280000	0.00000000
8	44.69480000	1.70460000	0.00000000
9	51.00280000	0.16740000	0.00000000
10	57.56080000	0.71610000	0.00000000
11	67.11390000	2.46590000	0.00000000
12	75.95190000	1.88060000	0.00000000
13	77.55670000	1.31700000	0.00000000
14	87.16620000	6.10480000	0.00000000
15	91.11380000	2.82770000	0.00000000
16	94.75370000	2.04050000	0.00000000
17	104.23590000	0.98250000	0.00000000
18	115.31620000	1.41570000	0.00000000
19	124.35740000	1.69890000	0.00000000
20	145.29970000	5.43270000	0.00000000
21	148.27340000	1.80890000	0.00000000
22	153.60820000	2.06360000	0.00000000
23	168.09090000	0.53210000	0.00000000
24	179.27260000	2.35880000	0.00000000
25	185.90950000	7.12710000	0.00000000
26	206.84520000	0.22000000	0.00000000
27	208.84600000	0.24540000	0.00000000
28	223.33740000	9.53690000	0.00000000
29	246.53180000	0.07070000	0.00000000
30	259.28380000	1.25970000	0.00000000
31	262.15710000	0.81130000	0.00000000
32	268.13340000	0.39790000	0.00000000
33	268.97240000	12.66210000	0.00000000
34	274.07130000	1.73740000	0.00000000
35	310.35920000	24.01480000	0.00000000
36	328.21330000	0.53010000	0.00000000
37	349.10690000	9.47230000	0.00000000
38	352.32820000	33.29760000	0.00000000
39	380.67260000	45.07900000	0.00000000
40	401.20170000	31.36930000	0.00000000
41	412.31750000	56.33070000	0.00000000
42	423.39160000	87.52450000	0.00000000
43	431.23790000	10.23420000	0.00000000
44	433.32330000	4.82340000	0.00000000
45	449.13060000	6.09230000	0.00000000
46	456.02350000	11.61510000	0.00000000
47	460.07560000	37.15990000	0.00000000
48	477.82490000	13.33020000	0.00000000
49	481.49560000	19.57760000	0.00000000
50	500.97310000	23.17990000	0.00000000
51	532.13300000	193.86880000	0.00000000
52	538.80000000	7.78150000	0.00000000
53	553.18520000	58.51090000	0.00000000
54	574.42270000	4.91340000	0.00000000
55	583.81280000	2.42850000	0.00000000
56	603.06340000	25.81750000	0.00000000
57	635.09300000	0.10340000	0.00000000
58	647.77160000	1.33620000	0.00000000
59	673.11160000	76.91920000	0.00000000
60	690.39230000	42.65060000	0.00000000

61	691.67160000	33.15230000	0.00000000
62	712.76830000	65.01050000	0.00000000
63	728.70140000	22.61710000	0.00000000
64	748.45110000	75.87020000	0.00000000
65	753.88210000	41.64660000	0.00000000
66	769.31920000	6.37980000	0.00000000
67	778.74720000	28.40060000	0.00000000
68	790.25170000	17.06670000	0.00000000
69	810.13240000	2.93640000	0.00000000
70	828.27820000	10.89250000	0.00000000
71	841.49400000	80.40690000	0.00000000
72	843.41680000	100.51010000	0.00000000
73	844.44260000	3.67450000	0.00000000
74	859.30490000	27.44690000	0.00000000
75	874.02060000	23.75410000	0.00000000
76	875.28660000	1.76280000	0.00000000
77	890.99190000	62.84230000	0.00000000
78	896.48490000	25.85930000	0.00000000
79	902.38570000	29.82940000	0.00000000
80	917.04300000	13.40170000	0.00000000
81	927.45840000	8.78670000	0.00000000
82	928.68860000	0.10590000	0.00000000
83	931.08540000	1.24550000	0.00000000
84	944.88010000	2.50250000	0.00000000
85	946.14530000	2.39690000	0.00000000
86	953.03850000	5.16520000	0.00000000
87	962.68060000	0.18490000	0.00000000
88	971.03990000	0.20470000	0.00000000
89	973.53010000	2.31070000	0.00000000
90	976.01850000	1.18390000	0.00000000
91	985.27060000	0.46920000	0.00000000
92	989.17090000	68.21710000	0.00000000
93	1008.93180000	39.66400000	0.00000000
94	1040.79190000	27.66140000	0.00000000
95	1042.63330000	9.19280000	0.00000000
96	1043.64380000	15.92490000	0.00000000
97	1051.65870000	0.82610000	0.00000000
98	1054.79200000	8.07850000	0.00000000
99	1055.35340000	131.42810000	0.00000000
100	1059.35070000	2.91920000	0.00000000
101	1070.65670000	63.45260000	0.00000000
102	1102.30840000	34.90580000	0.00000000
103	1131.63720000	109.38720000	0.00000000
104	1152.84500000	123.02720000	0.00000000
105	1167.19310000	1.55720000	0.00000000
106	1168.50080000	3.26680000	0.00000000
107	1172.20810000	33.57660000	0.00000000
108	1173.89330000	0.17760000	0.00000000
109	1181.94450000	315.39650000	0.00000000
110	1185.45320000	78.97640000	0.00000000
111	1187.44350000	17.41850000	0.00000000
112	1191.87810000	321.71960000	0.00000000
113	1201.33570000	58.00680000	0.00000000
114	1207.35670000	10.78340000	0.00000000
115	1208.84060000	3.77380000	0.00000000
116	1224.03080000	5.39640000	0.00000000
117	1234.52560000	372.28240000	0.00000000
118	1257.39270000	0.21860000	0.00000000
119	1268.64840000	23.67530000	0.00000000
120	1270.74330000	3.12670000	0.00000000
121	1275.74620000	227.86690000	0.00000000
122	1280.28840000	7.94680000	0.00000000
123	1306.86010000	35.71270000	0.00000000
124	1311.51110000	46.33560000	0.00000000

125	1324.37630000	23.28310000	0.00000000
126	1324.90550000	6.21780000	0.00000000
127	1341.29900000	2.66560000	0.00000000
128	1344.34770000	13.11360000	0.00000000
129	1346.13070000	68.76650000	0.00000000
130	1359.85480000	12.61900000	0.00000000
131	1368.75520000	28.79970000	0.00000000
132	1369.86050000	4.06710000	0.00000000
133	1394.33220000	29.94670000	0.00000000
134	1396.92940000	57.83410000	0.00000000
135	1400.80850000	5.50740000	0.00000000
136	1413.16060000	680.23440000	0.00000000
137	1420.82360000	74.48720000	0.00000000
138	1466.70150000	0.23470000	0.00000000
139	1470.50470000	37.73080000	0.00000000
140	1471.88170000	3.03820000	0.00000000
141	1477.17910000	8.49310000	0.00000000
142	1477.67370000	7.31740000	0.00000000
143	1484.16520000	15.19560000	0.00000000
144	1484.43370000	2.85720000	0.00000000
145	1484.61660000	3.06340000	0.00000000
146	1486.72410000	3.64460000	0.00000000
147	1487.75920000	48.10530000	0.00000000
148	1490.53470000	22.06600000	0.00000000
149	1495.46450000	10.05050000	0.00000000
150	1498.82750000	1.15000000	0.00000000
151	1502.45570000	48.32950000	0.00000000
152	1510.83980000	150.76850000	0.00000000
153	1518.18620000	19.26260000	0.00000000
154	1519.58130000	85.07070000	0.00000000
155	1522.01660000	17.31450000	0.00000000
156	1529.86200000	3.09270000	0.00000000
157	1571.40620000	21.88000000	0.00000000
158	1620.15920000	78.19900000	0.00000000
159	1628.69540000	280.22560000	0.00000000
160	1639.85620000	190.65800000	0.00000000
161	1664.30240000	596.74340000	0.00000000
162	1797.59660000	623.15590000	0.00000000
163	2974.97500000	104.79520000	0.00000000
164	2991.69880000	67.96090000	0.00000000
165	3008.61170000	140.93820000	0.00000000
166	3021.99730000	89.17410000	0.00000000
167	3036.94280000	13.54730000	0.00000000
168	3038.09260000	35.62120000	0.00000000
169	3041.12010000	55.51300000	0.00000000
170	3045.18050000	24.54740000	0.00000000
171	3051.68940000	26.28320000	0.00000000
172	3058.74810000	26.05270000	0.00000000
173	3071.22630000	39.41680000	0.00000000
174	3075.88400000	66.16870000	0.00000000
175	3086.66350000	51.60430000	0.00000000
176	3099.69330000	9.61980000	0.00000000
177	3101.19940000	34.53540000	0.00000000
178	3105.38140000	17.52810000	0.00000000
179	3110.63470000	22.40440000	0.00000000
180	3110.86730000	45.29970000	0.00000000
181	3115.01150000	69.89650000	0.00000000
182	3117.18720000	65.33060000	0.00000000
183	3138.67720000	30.51500000	0.00000000
184	3143.00480000	9.89680000	0.00000000
185	3145.06590000	31.65450000	0.00000000
186	3149.92400000	16.99510000	0.00000000
187	3157.12220000	9.21470000	0.00000000
188	3166.04980000	2.48850000	0.00000000

189	3167.92120000	22.68670000	0.00000000
190	3170.61080000	50.30660000	0.00000000
191	3178.94720000	53.74960000	0.00000000
192	3182.82450000	51.17360000	0.00000000
193	3186.73320000	12.03390000	0.00000000
194	3193.10610000	5.96400000	0.00000000
195	3199.01210000	14.60190000	0.00000000

S11.5. LITHIATED INTERMEDIATE FROM 2a WITH COORDINATED CHLOROFORMATE AND THF (2nd ISOMER)

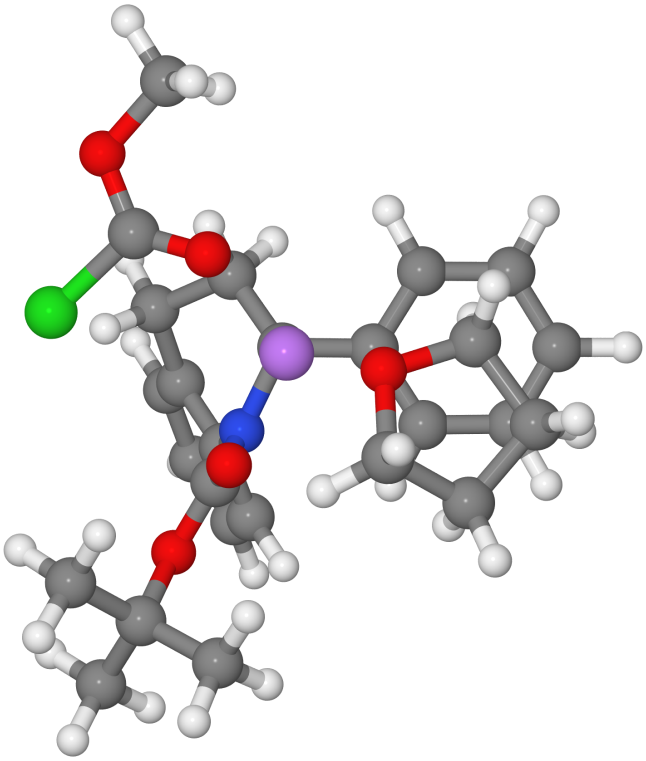

Route	: # opt freq b3lyp/6-311g(d,p) scrf=(solvent=thf) geom=connectivity empi
	: ricaldispersion=gd3bj int=ultrafine pop=(regular,mk)
SMILES	: [Li].CC(C)(C)OC(=O)N1c2ccccc2CC[C]1c3ccccc3.COC(=O)Cl.C1CCOC1
Formula	: $C_{26}H_{33}ClLiNO_5$
Charge	: 0
Multiplicity	: 1
Dipole	: 17.6782 Debye
Energy	: -1909.89232036 a.u.
Gibbs Energy	: -1909.41351500 a.u.
Number of imaginary frequencies	: 1 (-9.6 cm^{-1})

S11.5.1. Cartesian Co-ordinates (XYZ format)

67

C	-4.24135208	-1.66082597	-2.72607803
C	-3.85209203	-2.46563601	-1.65634000
C	-2.80553508	-2.07017303	-0.82433999
C	-2.16646791	-0.85726500	-1.06435204
C	-2.55028605	-0.03546900	-2.13329792
C	-3.58599401	-0.45170900	-2.96542692
N	-1.02716899	-0.39389300	-0.33956501
C	0.13565899	-0.03569500	-1.16918802
C	-0.24964000	0.93033201	-2.28999805
C	-1.76576805	1.23618901	-2.29239607
C	-1.03330195	-0.13202900	0.98636699

O	-0.05009400	0.32150301	1.58855104
O	-2.20074892	-0.41946599	1.57500803
C	-2.40415597	-0.25662601	3.02794909
C	-2.25103402	1.21302605	3.41594005
C	-1.44754696	-1.16927302	3.79351091
C	-3.84862089	-0.71646100	3.20454192
C	0.98955297	-1.15118003	-1.48511899
Li	1.46612704	0.73769802	0.46545199
C	0.98114699	-2.36462998	-0.73752898
C	1.86798596	-3.39742303	-1.00211000
C	2.82043910	-3.29649901	-2.02167296
C	2.84893298	-2.12029409	-2.77524900
C	1.96615195	-1.07782805	-2.52148890
H	-5.04993677	-1.97579706	-3.37558389
H	-4.35660410	-3.40749002	-1.47353196
H	-2.49198794	-2.68849611	0.00635600
H	-3.88484406	0.17420000	-3.79976702
H	-0.00010900	0.48878199	-3.26440597
H	0.29068601	1.88751101	-2.25560308
H	-2.05269909	1.76598203	-3.20321107
H	-1.99399602	1.89572501	-1.44746006
H	-2.54380488	1.34274399	4.46066380
H	-2.90356588	1.83392298	2.79751706
H	-1.22333705	1.54659104	3.29052711
H	-1.70223296	-1.14974403	4.85582113
H	-1.54328096	-2.19783711	3.43751311
H	-0.41541201	-0.84711200	3.67303801
H	-4.13732576	-0.64112598	4.25483704
H	-3.96118307	-1.75395095	2.88374901
H	-4.52113295	-0.09445800	2.61080408
H	0.26109800	-2.48024201	0.06325100
H	1.81742001	-4.30022383	-0.40052301
H	3.51379299	-4.10488510	-2.22032499
H	3.57584000	-2.01053596	-3.57472301
H	2.04142189	-0.17452800	-3.11551309
C	0.92651200	3.53611588	-0.23574600
O	1.69877195	2.65491796	0.02594900
O	1.09099996	4.51255512	-1.08986700
C	2.34897208	4.49540091	-1.83218300
H	3.18307590	4.55622196	-1.13598001
H	2.40569091	3.58152103	-2.42119789
H	2.30279803	5.37146711	-2.47020411
Cl	-0.65138501	3.66639090	0.54273999
O	3.05222988	0.02541100	1.30870700
C	3.97956896	-0.84762001	0.60359401
C	2.87747407	-0.47531399	2.65106392
C	4.13498783	-2.10878992	1.47189701
H	3.54324508	-1.06382501	-0.36936399
H	4.92515182	-0.31581300	0.47106901
C	2.99786592	-1.98409998	2.49945903
H	3.66606903	-0.07042500	3.29667211
H	1.90530705	-0.13216600	3.00003004
H	5.10299015	-2.11109209	1.97850704
H	4.05710697	-3.01346707	0.87053502
H	3.21685910	-2.48707795	3.44259596
H	2.06824899	-2.38557696	2.09029007

S11.5.2. Frequencies

Mode	IR frequency	IR intensity	Raman intensity
1	-9.64570000	0.87870000	0.00000000
2	13.61020000	0.50670000	0.00000000
3	18.00780000	0.76600000	0.00000000
4	27.40750000	1.97000000	0.00000000
5	31.56740000	2.07270000	0.00000000
6	36.73820000	3.00490000	0.00000000
7	38.82220000	0.47980000	0.00000000
8	43.14640000	0.11020000	0.00000000
9	52.89830000	0.81930000	0.00000000
10	60.27200000	1.00430000	0.00000000
11	70.35300000	1.31540000	0.00000000
12	74.62880000	1.77500000	0.00000000
13	83.35560000	0.79170000	0.00000000
14	87.90070000	0.40540000	0.00000000
15	94.12460000	0.73950000	0.00000000
16	108.73620000	1.48280000	0.00000000
17	113.37920000	1.98980000	0.00000000
18	116.21900000	1.12550000	0.00000000
19	128.37240000	2.36200000	0.00000000
20	136.92800000	0.64230000	0.00000000
21	142.99400000	3.15620000	0.00000000
22	159.96340000	3.44600000	0.00000000
23	166.00170000	1.82700000	0.00000000
24	170.76200000	2.63390000	0.00000000
25	181.87110000	2.46920000	0.00000000
26	204.61650000	0.67330000	0.00000000
27	206.16450000	2.06470000	0.00000000
28	222.56100000	7.16140000	0.00000000
29	244.85960000	0.08810000	0.00000000
30	263.46420000	6.65640000	0.00000000
31	265.80830000	0.23480000	0.00000000
32	268.42270000	3.64290000	0.00000000
33	273.55070000	1.82220000	0.00000000
34	289.32710000	5.23720000	0.00000000
35	314.45120000	0.75770000	0.00000000
36	327.14400000	1.06570000	0.00000000
37	350.34960000	1.76360000	0.00000000
38	362.95370000	19.90710000	0.00000000
39	394.03660000	81.54750000	0.00000000
40	400.21810000	23.46180000	0.00000000
41	419.48340000	37.95490000	0.00000000
42	423.82530000	37.76790000	0.00000000
43	426.97400000	2.01270000	0.00000000
44	446.36260000	11.30380000	0.00000000
45	454.96880000	24.59210000	0.00000000
46	459.84280000	27.62390000	0.00000000
47	469.50730000	80.39700000	0.00000000
48	480.39290000	10.25590000	0.00000000
49	482.68230000	48.44080000	0.00000000
50	494.52180000	66.09880000	0.00000000
51	504.82780000	112.57570000	0.00000000
52	537.75670000	12.18860000	0.00000000
53	547.97950000	65.81050000	0.00000000
54	574.20130000	5.02790000	0.00000000
55	589.18100000	6.74940000	0.00000000
56	602.01510000	35.84640000	0.00000000
57	634.75130000	0.10310000	0.00000000
58	645.32320000	0.52200000	0.00000000
59	687.11720000	40.39480000	0.00000000
60	690.13960000	45.65390000	0.00000000

61	695.60590000	15.85210000	0.00000000
62	706.63660000	69.53960000	0.00000000
63	728.46000000	24.65630000	0.00000000
64	747.28570000	74.13140000	0.00000000
65	754.21190000	37.51640000	0.00000000
66	769.56960000	5.86080000	0.00000000
67	776.67910000	32.29880000	0.00000000
68	789.33190000	15.14940000	0.00000000
69	809.43010000	4.72630000	0.00000000
70	821.02750000	6.12920000	0.00000000
71	824.68340000	6.41630000	0.00000000
72	840.33040000	103.25460000	0.00000000
73	843.67680000	62.94730000	0.00000000
74	852.40160000	23.45660000	0.00000000
75	874.59820000	3.11780000	0.00000000
76	879.08250000	20.49720000	0.00000000
77	893.90310000	48.35550000	0.00000000
78	895.43230000	40.98240000	0.00000000
79	903.62710000	24.31340000	0.00000000
80	920.34930000	17.26720000	0.00000000
81	927.82480000	0.06550000	0.00000000
82	930.62220000	1.93150000	0.00000000
83	931.62840000	5.54510000	0.00000000
84	945.44130000	2.24440000	0.00000000
85	946.59980000	2.54800000	0.00000000
86	953.61870000	7.52900000	0.00000000
87	961.70940000	0.25210000	0.00000000
88	969.47220000	0.29850000	0.00000000
89	971.46060000	0.82040000	0.00000000
90	972.94420000	0.53480000	0.00000000
91	984.70140000	0.34750000	0.00000000
92	989.48400000	73.04910000	0.00000000
93	1011.09690000	42.17270000	0.00000000
94	1042.86690000	19.72410000	0.00000000
95	1043.41660000	2.74250000	0.00000000
96	1044.90760000	29.00360000	0.00000000
97	1049.87490000	1.62950000	0.00000000
98	1051.50290000	113.28900000	0.00000000
99	1055.50820000	8.50220000	0.00000000
100	1061.17910000	4.45340000	0.00000000
101	1073.02780000	77.82400000	0.00000000
102	1102.52080000	42.96350000	0.00000000
103	1133.11050000	109.95690000	0.00000000
104	1155.40370000	134.96620000	0.00000000
105	1159.85420000	1.22180000	0.00000000
106	1168.89040000	12.68010000	0.00000000
107	1169.40300000	3.56600000	0.00000000
108	1174.57570000	0.08270000	0.00000000
109	1179.65970000	418.64270000	0.00000000
110	1187.67170000	171.98750000	0.00000000
111	1191.99990000	202.16170000	0.00000000
112	1193.93550000	17.78540000	0.00000000
113	1201.88710000	52.12710000	0.00000000
114	1211.79740000	8.43540000	0.00000000
115	1224.88770000	24.02600000	0.00000000
116	1227.75120000	8.37750000	0.00000000
117	1228.93210000	369.55110000	0.00000000
118	1254.17130000	3.90120000	0.00000000
119	1268.10000000	24.61910000	0.00000000
120	1277.05060000	195.87000000	0.00000000
121	1279.39670000	44.98380000	0.00000000
122	1279.98320000	8.47530000	0.00000000
123	1308.18560000	50.39420000	0.00000000
124	1311.45860000	32.12720000	0.00000000

125	1323.91410000	2.17410000	0.00000000
126	1325.15160000	34.79480000	0.00000000
127	1338.54730000	1.36060000	0.00000000
128	1342.13110000	14.24500000	0.00000000
129	1346.97850000	81.60570000	0.00000000
130	1359.86400000	13.90450000	0.00000000
131	1369.03300000	23.31150000	0.00000000
132	1369.76100000	9.08980000	0.00000000
133	1393.26890000	27.46420000	0.00000000
134	1396.59950000	40.89540000	0.00000000
135	1401.69460000	4.93120000	0.00000000
136	1418.65540000	64.69990000	0.00000000
137	1420.88610000	690.66430000	0.00000000
138	1465.87490000	0.21160000	0.00000000
139	1470.18720000	36.24650000	0.00000000
140	1471.62990000	4.22020000	0.00000000
141	1478.68290000	5.04810000	0.00000000
142	1479.62610000	16.04620000	0.00000000
143	1482.08550000	16.78760000	0.00000000
144	1482.12950000	8.05400000	0.00000000
145	1483.82800000	3.56650000	0.00000000
146	1484.87350000	3.67670000	0.00000000
147	1487.24830000	42.86140000	0.00000000
148	1490.58710000	23.47280000	0.00000000
149	1494.71960000	16.48990000	0.00000000
150	1497.96280000	2.90960000	0.00000000
151	1506.47540000	101.38270000	0.00000000
152	1508.63810000	13.13800000	0.00000000
153	1511.27370000	150.23390000	0.00000000
154	1518.21550000	20.10560000	0.00000000
155	1521.05470000	96.33110000	0.00000000
156	1525.84300000	8.75500000	0.00000000
157	1571.91100000	19.45540000	0.00000000
158	1620.41450000	92.80330000	0.00000000
159	1628.19270000	324.96320000	0.00000000
160	1640.79270000	138.25120000	0.00000000
161	1666.55030000	651.52630000	0.00000000
162	1786.01270000	686.67370000	0.00000000
163	2955.01050000	278.61140000	0.00000000
164	2977.45560000	58.43200000	0.00000000
165	3007.32070000	96.70270000	0.00000000
166	3022.67970000	71.75540000	0.00000000
167	3037.31700000	13.72810000	0.00000000
168	3038.64220000	35.69220000	0.00000000
169	3041.32370000	52.52100000	0.00000000
170	3045.66770000	24.85370000	0.00000000
171	3055.69760000	94.08290000	0.00000000
172	3058.72610000	20.92710000	0.00000000
173	3072.29970000	17.43390000	0.00000000
174	3077.98900000	61.68480000	0.00000000
175	3100.13220000	11.61600000	0.00000000
176	3101.28760000	30.41290000	0.00000000
177	3106.58970000	37.75910000	0.00000000
178	3111.78410000	43.80920000	0.00000000
179	3115.33990000	67.55890000	0.00000000
180	3117.24300000	34.18250000	0.00000000
181	3129.87310000	17.34680000	0.00000000
182	3139.04910000	29.53540000	0.00000000
183	3140.17880000	8.34150000	0.00000000
184	3143.32230000	30.16190000	0.00000000
185	3144.51330000	6.31510000	0.00000000
186	3148.43100000	22.86620000	0.00000000
187	3157.49220000	9.04440000	0.00000000
188	3159.98450000	11.27020000	0.00000000

189	3168.10450000	23.98290000	0.00000000
190	3168.55530000	47.36120000	0.00000000
191	3175.31610000	54.36170000	0.00000000
192	3182.54350000	20.98930000	0.00000000
193	3183.33190000	53.10150000	0.00000000
194	3196.43110000	6.77030000	0.00000000
195	3200.77280000	12.94720000	0.00000000

S11.6. LITHIATED INTERMEDIATE FROM 2a WITH COORDINATED CYANOFORMATE AND THF (1st ISOMER)

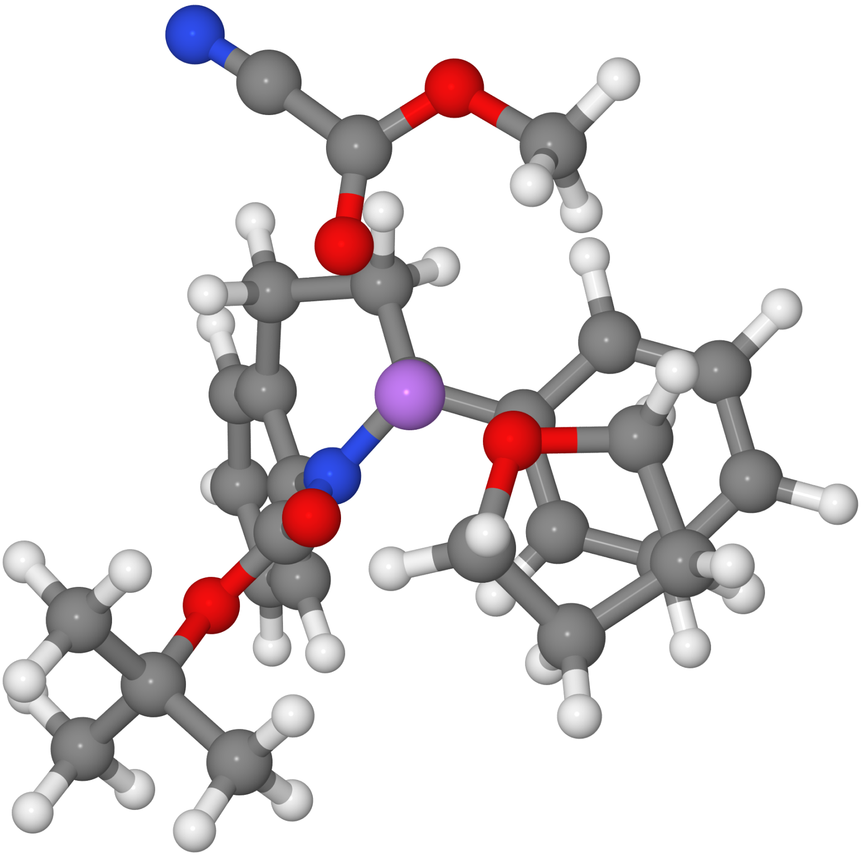

Route	: # opt freq b3lyp/6-311g(d,p) scrf=(solvent=thf) geom=connectivity empi	
	: ricaldispersion=gd3bj int=ultrafine pop=(regular,mk)	
SMILES	: [Li].CC(C)(C)OC(=O)N1c2ccccc2CC[C]1c3ccccc3.COC(=O)C#N.C1CCOC1	
Formula	: $C_{27}H_{33}LiN_2O_5$	
Charge	: 0	
Multiplicity	: 1	
Dipole	: 19.8996	Debye
Energy	: -1542.52284385	a.u.
Gibbs Energy	: -1542.03543600	a.u.
Number of imaginary frequencies	: 0	

S11.6.1. Cartesian Co-ordinates (XYZ format)

```
68

C  -4.74668884  1.86405504 -1.98543406
C  -4.48983717  0.51373100 -2.21842790
C  -3.36150098 -0.09154300 -1.66777694
C  -2.50583005  0.66369998 -0.87192601
C  -2.74976206  2.02146196 -0.62986499
C  -3.87326908  2.61579704 -1.19902599
N  -1.29216897  0.15936500 -0.30362901
C  -0.09675200  0.96474802 -0.55986500
```

C	-0.32188699	2.44212794	-0.36625901
C	-1.72149503	2.73558497	0.20364900
C	-1.25886095	-0.87950301	0.56692600
O	-0.22026999	-1.24840999	1.13108802
O	-2.44645000	-1.46448398	0.73584503
C	-2.61356592	-2.67518806	1.56943297
C	-2.26971602	-2.35989404	3.02360892
C	-1.77322698	-3.81607509	0.99877101
C	-4.10318804	-2.96711397	1.41426396
C	0.76941901	0.50219101	-1.59819400
Li	1.34126306	-0.18326600	0.94455397
C	0.71341801	-0.82270998	-2.12680602
C	1.60099006	-1.25893199	-3.09651399
C	2.60495996	-0.42056599	-3.59673905
C	2.68268704	0.88199103	-3.10009003
C	1.79814899	1.33830595	-2.12992692
H	-5.62168217	2.33251095	-2.42080092
H	-5.16329288	-0.06901700	-2.83608389
H	-3.15088701	-1.13803804	-1.84254706
H	-4.06624413	3.66862893	-1.02317798
H	-0.22176200	2.97787404	-1.32169700
H	0.42773399	2.91298389	0.29390800
H	-1.91241503	3.80948997	0.23242100
H	-1.76978099	2.36675501	1.23523498
H	-2.53527308	-3.21519589	3.64961505
H	-2.84234691	-1.49478996	3.36574292
H	-1.20837700	-2.15162301	3.14121509
H	-2.01216602	-4.74006081	1.53038394
H	-2.00394392	-3.96133995	-0.05926900
H	-0.70940000	-3.61437392	1.10556805
H	-4.37019491	-3.85186791	1.99549997
H	-4.35025120	-3.14866590	0.36649299
H	-4.69582987	-2.12220001	1.76994097
H	-0.05127700	-1.49981499	-1.76787806
H	1.51233006	-2.27383900	-3.47136307
H	3.29883599	-0.77211100	-4.35041714
H	3.44403005	1.55825806	-3.47637606
H	1.88105905	2.36178899	-1.78516996
C	1.82184398	2.53988791	2.05107808
O	1.48559594	1.36701095	2.04990697
C	1.02809095	3.55760503	2.71333694
N	0.36389399	4.32036018	3.27097988
O	2.92561412	3.04625201	1.51607394
C	3.80075788	2.11120200	0.83635002
H	4.01750612	1.26382005	1.48504901
H	3.32897711	1.77126002	-0.08799300
H	4.70147085	2.67658091	0.61898202
O	2.86537600	-1.36737394	0.85771197
C	3.79513001	-1.35659003	-0.26171601
C	2.61314511	-2.73982406	1.23190498
C	3.85804605	-2.80033112	-0.78998798
H	3.40424705	-0.66143900	-1.00178099
H	4.76639986	-1.00375295	0.09418700
C	2.68217301	-3.49719596	-0.08523700
H	3.38828301	-3.07331395	1.93162501
H	1.64082801	-2.76914310	1.71987498
H	4.80165577	-3.27222991	-0.50738102
H	3.77108788	-2.82879400	-1.87540102
H	2.84065199	-4.56780815	0.05146100
H	1.75585198	-3.34430599	-0.64317000

S11.6.2. Frequencies

Mode	IR frequency	IR intensity	Raman intensity
1	14.18100000	0.48270000	0.00000000
2	18.94420000	0.31250000	0.00000000
3	24.79670000	2.05730000	0.00000000
4	32.75570000	0.88440000	0.00000000
5	36.24090000	1.68810000	0.00000000
6	43.67920000	0.88150000	0.00000000
7	53.15610000	1.86030000	0.00000000
8	57.70280000	2.25480000	0.00000000
9	65.57430000	3.84660000	0.00000000
10	69.72900000	0.64440000	0.00000000
11	83.73040000	0.88940000	0.00000000
12	89.01490000	0.99350000	0.00000000
13	91.71940000	0.81760000	0.00000000
14	98.66720000	7.03660000	0.00000000
15	107.58760000	0.40920000	0.00000000
16	111.05220000	7.00020000	0.00000000
17	121.67200000	4.30300000	0.00000000
18	123.05090000	2.58100000	0.00000000
19	136.42480000	3.48660000	0.00000000
20	146.66740000	3.03540000	0.00000000
21	151.98510000	5.03680000	0.00000000
22	163.16180000	0.83350000	0.00000000
23	184.68510000	8.64430000	0.00000000
24	191.96250000	1.20890000	0.00000000
25	204.97430000	10.54010000	0.00000000
26	206.64220000	2.05650000	0.00000000
27	215.28760000	3.25130000	0.00000000
28	235.31650000	18.19860000	0.00000000
29	243.09680000	0.68870000	0.00000000
30	263.36380000	6.89530000	0.00000000
31	267.87140000	0.09460000	0.00000000
32	269.77860000	0.68500000	0.00000000
33	288.40330000	31.43120000	0.00000000
34	294.38190000	1.98270000	0.00000000
35	305.35050000	16.64530000	0.00000000
36	324.89780000	4.35270000	0.00000000
37	330.36770000	25.68090000	0.00000000
38	343.54040000	30.41010000	0.00000000
39	350.77940000	1.70510000	0.00000000
40	359.56320000	47.18690000	0.00000000
41	374.63340000	25.78560000	0.00000000
42	415.73720000	1.61930000	0.00000000
43	425.70660000	2.94740000	0.00000000
44	429.99540000	1.00000000	0.00000000
45	435.75710000	8.83230000	0.00000000
46	455.61690000	11.05300000	0.00000000
47	457.80460000	3.84530000	0.00000000
48	473.26030000	55.84470000	0.00000000
49	487.08870000	43.99770000	0.00000000
50	494.49500000	6.19400000	0.00000000
51	529.53720000	18.33680000	0.00000000
52	533.53350000	119.39030000	0.00000000
53	543.45080000	24.29810000	0.00000000
54	548.15060000	11.70370000	0.00000000
55	571.04930000	15.04380000	0.00000000
56	589.55010000	5.85920000	0.00000000
57	597.55750000	40.08620000	0.00000000
58	620.50050000	24.74460000	0.00000000
59	633.91900000	0.19540000	0.00000000
60	644.11050000	0.96460000	0.00000000

61	686.52720000	16.52110000	0.00000000
62	692.48230000	82.36730000	0.00000000
63	696.03280000	15.77920000	0.00000000
64	707.00720000	53.99930000	0.00000000
65	730.62080000	16.91050000	0.00000000
66	749.24840000	45.84280000	0.00000000
67	756.29500000	26.09050000	0.00000000
68	767.48700000	11.53270000	0.00000000
69	775.15360000	48.95470000	0.00000000
70	791.43930000	10.93020000	0.00000000
71	810.29480000	5.68680000	0.00000000
72	821.62470000	3.29970000	0.00000000
73	824.41280000	6.07550000	0.00000000
74	844.79670000	32.03060000	0.00000000
75	853.53830000	5.23480000	0.00000000
76	873.04600000	165.36200000	0.00000000
77	878.40270000	2.59930000	0.00000000
78	879.84360000	24.61220000	0.00000000
79	894.44470000	37.53130000	0.00000000
80	895.65230000	44.42710000	0.00000000
81	916.31300000	21.23200000	0.00000000
82	922.67030000	13.32490000	0.00000000
83	928.38430000	0.09630000	0.00000000
84	930.85350000	2.38740000	0.00000000
85	933.09590000	7.14110000	0.00000000
86	952.12170000	0.15190000	0.00000000
87	961.47010000	16.69620000	0.00000000
88	968.32880000	0.09070000	0.00000000
89	970.82780000	0.12160000	0.00000000
90	972.51420000	0.80410000	0.00000000
91	976.56170000	0.19070000	0.00000000
92	988.40640000	46.41990000	0.00000000
93	988.99650000	55.39700000	0.00000000
94	994.86860000	39.99910000	0.00000000
95	1019.31050000	40.96750000	0.00000000
96	1042.98890000	11.17850000	0.00000000
97	1044.12310000	9.41650000	0.00000000
98	1045.79930000	24.89560000	0.00000000
99	1050.80930000	3.15380000	0.00000000
100	1051.76520000	116.05610000	0.00000000
101	1055.94020000	6.17350000	0.00000000
102	1061.48850000	22.01700000	0.00000000
103	1083.25150000	71.78040000	0.00000000
104	1105.52990000	44.35890000	0.00000000
105	1136.36730000	117.79510000	0.00000000
106	1159.45870000	0.82070000	0.00000000
107	1166.93020000	57.52410000	0.00000000
108	1173.01010000	47.38610000	0.00000000
109	1176.23760000	2.90650000	0.00000000
110	1179.76000000	10.57500000	0.00000000
111	1187.47390000	448.43580000	0.00000000
112	1194.48700000	106.91380000	0.00000000
113	1194.85960000	25.36070000	0.00000000
114	1203.28540000	66.46650000	0.00000000
115	1212.62990000	150.31780000	0.00000000
116	1216.47070000	66.34370000	0.00000000
117	1225.01870000	677.25580000	0.00000000
118	1229.12010000	21.68990000	0.00000000
119	1244.03060000	444.19070000	0.00000000
120	1256.99860000	5.50830000	0.00000000
121	1269.00090000	23.52140000	0.00000000
122	1277.70540000	112.95430000	0.00000000
123	1279.74730000	5.25270000	0.00000000
124	1280.50080000	20.43860000	0.00000000

125	1309.25110000	31.82610000	0.00000000
126	1314.82020000	35.15140000	0.00000000
127	1324.49310000	2.43710000	0.00000000
128	1335.20260000	32.54640000	0.00000000
129	1338.27330000	1.67440000	0.00000000
130	1339.27130000	1.30200000	0.00000000
131	1359.46570000	32.44940000	0.00000000
132	1363.12870000	98.60780000	0.00000000
133	1371.67320000	1.50570000	0.00000000
134	1379.49790000	173.35870000	0.00000000
135	1394.78020000	25.98200000	0.00000000
136	1397.67040000	49.22790000	0.00000000
137	1403.51050000	6.76080000	0.00000000
138	1414.98180000	723.42020000	0.00000000
139	1421.97560000	54.80490000	0.00000000
140	1466.61390000	0.18410000	0.00000000
141	1471.12690000	8.95830000	0.00000000
142	1473.44790000	40.26350000	0.00000000
143	1477.80820000	6.40980000	0.00000000
144	1481.98980000	6.77130000	0.00000000
145	1484.37270000	4.04350000	0.00000000
146	1484.90740000	2.41760000	0.00000000
147	1487.85080000	38.90450000	0.00000000
148	1491.39150000	19.54000000	0.00000000
149	1491.77710000	12.18330000	0.00000000
150	1495.30650000	21.06400000	0.00000000
151	1495.52450000	5.54970000	0.00000000
152	1498.21160000	1.49510000	0.00000000
153	1510.29520000	34.41200000	0.00000000
154	1512.17340000	280.96770000	0.00000000
155	1518.18740000	39.54610000	0.00000000
156	1519.84170000	61.77220000	0.00000000
157	1520.89940000	81.68950000	0.00000000
158	1527.52820000	4.89780000	0.00000000
159	1569.69600000	19.73220000	0.00000000
160	1622.30540000	201.96800000	0.00000000
161	1625.34390000	319.53770000	0.00000000
162	1642.58810000	124.40950000	0.00000000
163	1663.39260000	450.71570000	0.00000000
164	1698.11580000	644.93490000	0.00000000
165	2312.74290000	851.78620000	0.00000000
166	2903.82640000	739.72710000	0.00000000
167	2961.86680000	76.78330000	0.00000000
168	3013.20740000	83.77610000	0.00000000
169	3015.37050000	51.21060000	0.00000000
170	3038.62780000	13.82870000	0.00000000
171	3039.58390000	32.44130000	0.00000000
172	3041.52170000	44.04420000	0.00000000
173	3046.68540000	24.31650000	0.00000000
174	3050.16290000	44.89250000	0.00000000
175	3058.40070000	68.09010000	0.00000000
176	3058.92050000	32.45820000	0.00000000
177	3088.74360000	46.61880000	0.00000000
178	3101.45550000	7.95470000	0.00000000
179	3103.15660000	30.55840000	0.00000000
180	3107.38260000	30.89670000	0.00000000
181	3112.48130000	40.54430000	0.00000000
182	3116.82400000	29.59080000	0.00000000
183	3116.91780000	74.02540000	0.00000000
184	3132.11190000	17.71150000	0.00000000
185	3136.03620000	2.27870000	0.00000000
186	3140.13920000	11.92240000	0.00000000
187	3143.38850000	0.50610000	0.00000000
188	3146.31060000	36.10400000	0.00000000

189	3146.44670000	30.31190000	0.00000000
190	3150.71870000	27.54140000	0.00000000
191	3161.10370000	4.90910000	0.00000000
192	3171.24720000	22.29190000	0.00000000
193	3176.00470000	27.53270000	0.00000000
194	3182.29740000	57.96470000	0.00000000
195	3182.75170000	1.93280000	0.00000000
196	3185.78760000	48.05740000	0.00000000
197	3188.37630000	29.14340000	0.00000000
198	3204.48720000	12.23840000	0.00000000

S11.7. LITHIATED INTERMEDIATE FROM 2a WITH COORDINATED CYANOFORMATE AND THF (2nd ISOMER)

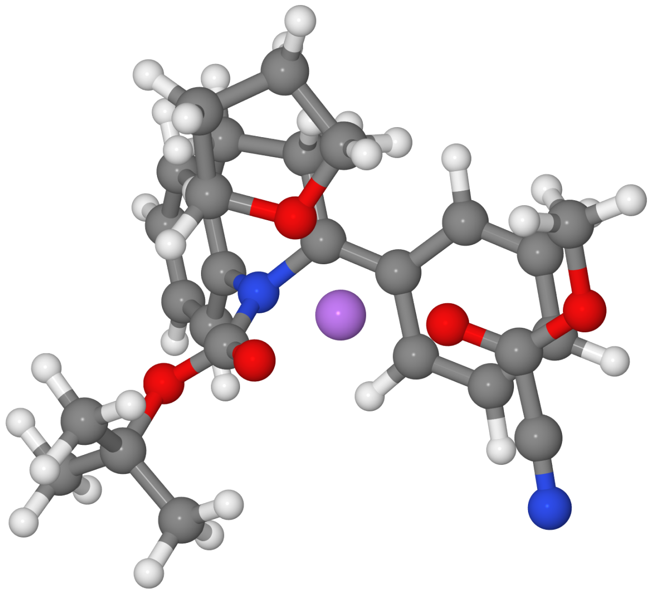

Route	: # opt freq b3lyp/6-311g(d,p) scrf=(solvent=thf) geom=connectivity empi	
	: ricaldispersion=gd3bj int=ultrafine pop=(regular,mk)	
SMILES	: [Li].CC(C)(C)OC(=O)N1c2ccccc2CC[C]1c3ccccc3.COC(=O)C#N.C1CCOC1	
Formula	: $C_{27}H_{33}LiN_2O_5$	
Charge	: 0	
Multiplicity	: 1	
Dipole	: 27.5512	Debye
Energy	: -1542.52179113	a.u.
Gibbs Energy	: -1542.03634000	a.u.
Number of imaginary frequencies	: 0	

S11.7.1. Cartesian Co-ordinates (XYZ format)

68

C	-5.14303780	1.13559699	-2.46853900
C	-4.83616400	-0.22018400	-2.36355901
C	-3.67315102	-0.62807697	-1.71303999
C	-2.83392501	0.33146399	-1.15658104
C	-3.12853193	1.69754899	-1.25135303
C	-4.28681707	2.08955097	-1.91864395
N	-1.58013296	0.02153000	-0.53360099
C	-0.46359000	0.80373502	-1.01955199
C	-0.70151502	2.29316092	-1.05372500
C	-2.14076209	2.64243102	-0.62169403
C	-1.46494198	-0.78036702	0.55864900
O	-0.39766100	-0.97706598	1.14922798
O	-2.62440300	-1.34559202	0.90898198
C	-2.71391797	-2.33912992	2.00064898
C	-2.32526588	-1.69221103	3.32875395
C	-1.85572302	-3.55718589	1.66513002
C	-4.19701385	-2.69803309	1.98550296

C	0.63300800	0.17433999	-1.58045697
Li	1.35910797	-0.29852599	0.97218198
C	0.75395000	-1.26339698	-1.70597398
C	1.88893497	-1.85411203	-2.20569491
C	3.01771593	-1.09697902	-2.58423710
C	2.90510297	0.30639899	-2.53730202
C	1.78118801	0.92726701	-2.04218698
H	-6.04397297	1.44968295	-2.98250198
H	-5.49618483	-0.96243399	-2.79701209
H	-3.42008305	-1.67646897	-1.63087296
H	-4.52048588	3.14536905	-2.00402308
H	-0.54017299	2.67678094	-2.07090211
H	0.00318800	2.84229994	-0.41381201
H	-2.37816501	3.67826295	-0.86993903
H	-2.21779895	2.54822612	0.46842301
H	-2.54663801	-2.38456511	4.14444304
H	-2.90662408	-0.78071702	3.48665690
H	-1.26574898	-1.44653296	3.35182595
H	-2.03637099	-4.33970022	2.40581298
H	-2.12581801	-3.94903111	0.68172902
H	-0.79648101	-3.30802798	1.66911495
H	-4.40781689	-3.43683505	2.76116490
H	-4.47884989	-3.11661601	1.01748300
H	-4.80596781	-1.81123698	2.17145300
H	-0.08508400	-1.88523495	-1.42179704
H	1.92670798	-2.93555188	-2.28437090
H	3.88304591	-1.57160699	-3.02764297
H	3.73428512	0.91723597	-2.88167405
H	1.74932098	2.00943303	-1.99919498
C	4.11828518	-1.08647501	-0.07171700
O	3.19705296	-0.55703402	0.53745002
C	4.18551922	-2.51472497	-0.30331600
N	4.22870398	-3.66764498	-0.38770100
O	5.26746416	-0.48624501	-0.38907000
C	5.34563398	0.92300200	-0.07866100
H	4.56189585	1.46200502	-0.60697001
H	5.24529696	1.07470500	0.99612802
H	6.32833004	1.23349798	-0.42137000
O	1.36602902	1.37475300	1.92071605
C	2.26604009	2.47264504	1.62797797
C	0.19776200	1.85635304	2.62548304
C	1.51720202	3.74201202	2.03063798
H	3.17625308	2.33365297	2.21764302
H	2.51830411	2.43073511	0.56820899
C	0.58316499	3.23811102	3.13962507
H	-0.63921797	1.90351200	1.92365801
H	-0.04139500	1.14044094	3.41166496
H	0.93349898	4.12603807	1.19023895
H	2.19366789	4.52972698	2.36360002
H	-0.28524101	3.87945008	3.29401898
H	1.12423897	3.15491509	4.08589792

S11.7.2. Frequencies

Mode	IR frequency	IR intensity	Raman intensity
1	18.73030000	3.48300000	0.00000000
2	21.31390000	1.27460000	0.00000000
3	30.02350000	0.73630000	0.00000000
4	33.40750000	0.51180000	0.00000000
5	37.25940000	5.81210000	0.00000000
6	38.31040000	1.61350000	0.00000000
7	45.91160000	1.60140000	0.00000000
8	55.69710000	0.98490000	0.00000000
9	61.56440000	8.03730000	0.00000000
10	69.57120000	0.23930000	0.00000000
11	77.07620000	0.25310000	0.00000000
12	88.55840000	2.40550000	0.00000000
13	94.04070000	2.17260000	0.00000000
14	99.47030000	2.75060000	0.00000000
15	111.33630000	7.14830000	0.00000000
16	112.06990000	2.67740000	0.00000000
17	122.22300000	9.94710000	0.00000000
18	124.52660000	2.32180000	0.00000000
19	132.73880000	0.66310000	0.00000000
20	139.63850000	21.30040000	0.00000000
21	151.84810000	19.25430000	0.00000000
22	158.70920000	0.47100000	0.00000000
23	168.05930000	3.65850000	0.00000000
24	175.30820000	21.20860000	0.00000000
25	181.38560000	16.94320000	0.00000000
26	196.42330000	4.99170000	0.00000000
27	205.21110000	50.33680000	0.00000000
28	209.94670000	3.82210000	0.00000000
29	212.43900000	13.28840000	0.00000000
30	228.20830000	3.39040000	0.00000000
31	243.86500000	2.89190000	0.00000000
32	254.96140000	4.85300000	0.00000000
33	262.48030000	1.95350000	0.00000000
34	270.41200000	0.21040000	0.00000000
35	279.79360000	9.07550000	0.00000000
36	313.45120000	28.21170000	0.00000000
37	324.03870000	5.94870000	0.00000000
38	330.80530000	10.47510000	0.00000000
39	350.32260000	2.88220000	0.00000000
40	353.54970000	1.79540000	0.00000000
41	358.33260000	73.51930000	0.00000000
42	397.54140000	19.63460000	0.00000000
43	414.53350000	61.20140000	0.00000000
44	425.89250000	18.10950000	0.00000000
45	430.16040000	28.05780000	0.00000000
46	444.71750000	1.06850000	0.00000000
47	454.98010000	7.84080000	0.00000000
48	457.19780000	2.60730000	0.00000000
49	478.20590000	66.56910000	0.00000000
50	483.00950000	15.91780000	0.00000000
51	508.64210000	73.64020000	0.00000000
52	536.45780000	4.18630000	0.00000000
53	543.97510000	38.89950000	0.00000000
54	562.27690000	44.96750000	0.00000000
55	564.85440000	6.24720000	0.00000000
56	583.28800000	1.30680000	0.00000000
57	598.03950000	12.50740000	0.00000000
58	613.84410000	43.65360000	0.00000000
59	626.48360000	125.20140000	0.00000000
60	628.05970000	127.75250000	0.00000000

61	644.90760000	45.89600000	0.00000000
62	684.69140000	21.44570000	0.00000000
63	695.66700000	20.80960000	0.00000000
64	707.25380000	32.92460000	0.00000000
65	725.79000000	6.99260000	0.00000000
66	743.55210000	101.87680000	0.00000000
67	753.78840000	22.99670000	0.00000000
68	760.09000000	15.99090000	0.00000000
69	772.27700000	57.67730000	0.00000000
70	789.27730000	0.69870000	0.00000000
71	793.11520000	1.52300000	0.00000000
72	809.24230000	0.51800000	0.00000000
73	820.94850000	31.65290000	0.00000000
74	845.42480000	14.60320000	0.00000000
75	852.65430000	14.67260000	0.00000000
76	874.42250000	126.39400000	0.00000000
77	875.99290000	23.11620000	0.00000000
78	877.35790000	3.19190000	0.00000000
79	891.52120000	55.89430000	0.00000000
80	896.32470000	68.19620000	0.00000000
81	912.75730000	21.97100000	0.00000000
82	918.29380000	8.01720000	0.00000000
83	927.98840000	12.67380000	0.00000000
84	928.66870000	0.27870000	0.00000000
85	930.93260000	2.59460000	0.00000000
86	952.62340000	1.01080000	0.00000000
87	964.90020000	17.38900000	0.00000000
88	968.08340000	23.99020000	0.00000000
89	970.52480000	0.44640000	0.00000000
90	971.67430000	7.19030000	0.00000000
91	971.92190000	1.80650000	0.00000000
92	985.35540000	25.44370000	0.00000000
93	989.43010000	0.16510000	0.00000000
94	1007.33930000	60.62680000	0.00000000
95	1022.07800000	42.93880000	0.00000000
96	1027.81100000	37.22120000	0.00000000
97	1040.06250000	20.97120000	0.00000000
98	1042.42270000	9.66450000	0.00000000
99	1050.75210000	0.96890000	0.00000000
100	1056.25370000	4.70690000	0.00000000
101	1059.39380000	2.30270000	0.00000000
102	1061.85180000	129.52120000	0.00000000
103	1077.83210000	82.29840000	0.00000000
104	1102.08290000	65.09110000	0.00000000
105	1136.21740000	125.50040000	0.00000000
106	1165.58760000	36.96980000	0.00000000
107	1166.76460000	1.57770000	0.00000000
108	1170.96750000	0.54600000	0.00000000
109	1176.23830000	8.25300000	0.00000000
110	1178.21480000	83.98990000	0.00000000
111	1187.72420000	485.37310000	0.00000000
112	1191.41000000	16.17790000	0.00000000
113	1195.66260000	4.18330000	0.00000000
114	1202.08910000	482.21970000	0.00000000
115	1205.19950000	19.50530000	0.00000000
116	1209.13360000	14.32520000	0.00000000
117	1218.32690000	8.63390000	0.00000000
118	1224.06850000	1397.43980000	0.00000000
119	1242.70460000	29.45390000	0.00000000
120	1258.24690000	0.21150000	0.00000000
121	1264.63970000	14.75570000	0.00000000
122	1268.72040000	24.67110000	0.00000000
123	1278.40940000	152.26920000	0.00000000
124	1279.48490000	5.16660000	0.00000000

125	1311.11360000	13.50620000	0.00000000
126	1320.46890000	18.09280000	0.00000000
127	1323.38170000	1.33880000	0.00000000
128	1335.84990000	15.21750000	0.00000000
129	1339.00270000	4.37400000	0.00000000
130	1344.84750000	1.31710000	0.00000000
131	1368.76450000	5.40030000	0.00000000
132	1369.28890000	7.21360000	0.00000000
133	1373.14140000	59.50310000	0.00000000
134	1394.83710000	128.05840000	0.00000000
135	1395.54150000	232.79460000	0.00000000
136	1398.72990000	2.64270000	0.00000000
137	1402.00740000	389.99440000	0.00000000
138	1421.79200000	25.51960000	0.00000000
139	1449.08210000	25.25780000	0.00000000
140	1466.92740000	0.14960000	0.00000000
141	1467.31430000	35.86110000	0.00000000
142	1476.85090000	18.25170000	0.00000000
143	1477.53380000	12.30340000	0.00000000
144	1484.35760000	4.45580000	0.00000000
145	1485.09050000	2.13250000	0.00000000
146	1485.09910000	10.87280000	0.00000000
147	1487.84630000	29.51370000	0.00000000
148	1490.03920000	41.18140000	0.00000000
149	1491.64770000	52.14580000	0.00000000
150	1492.68340000	9.18670000	0.00000000
151	1494.42360000	4.77190000	0.00000000
152	1498.02830000	12.49710000	0.00000000
153	1498.71770000	2.93050000	0.00000000
154	1518.11420000	33.89530000	0.00000000
155	1519.62280000	128.40180000	0.00000000
156	1522.01410000	12.21770000	0.00000000
157	1532.01470000	1.48220000	0.00000000
158	1534.82720000	6.82670000	0.00000000
159	1555.51480000	159.93170000	0.00000000
160	1623.40060000	81.56390000	0.00000000
161	1630.97860000	357.60860000	0.00000000
162	1644.57410000	95.20790000	0.00000000
163	1662.99030000	201.11140000	0.00000000
164	1678.50160000	715.40880000	0.00000000
165	2291.97790000	1132.02570000	0.00000000
166	2967.27020000	196.34130000	0.00000000
167	2976.70570000	66.43110000	0.00000000
168	3011.35590000	81.05800000	0.00000000
169	3038.43080000	59.52140000	0.00000000
170	3038.49410000	37.55590000	0.00000000
171	3039.58330000	35.49630000	0.00000000
172	3046.43270000	19.72640000	0.00000000
173	3046.74010000	19.81830000	0.00000000
174	3049.10980000	32.98090000	0.00000000
175	3053.18780000	39.56410000	0.00000000
176	3063.13570000	45.57490000	0.00000000
177	3085.72720000	68.27000000	0.00000000
178	3099.47940000	29.90220000	0.00000000
179	3101.38670000	9.67850000	0.00000000
180	3102.89370000	29.01440000	0.00000000
181	3103.12640000	26.00510000	0.00000000
182	3108.01030000	20.43310000	0.00000000
183	3112.35700000	42.15940000	0.00000000
184	3115.21330000	74.17270000	0.00000000
185	3116.77250000	63.86440000	0.00000000
186	3141.93090000	23.59130000	0.00000000
187	3142.33970000	3.05220000	0.00000000
188	3145.90530000	30.49380000	0.00000000

189	3146.53010000	23.51640000	0.00000000
190	3155.51660000	13.47710000	0.00000000
191	3161.43830000	8.50210000	0.00000000
192	3172.28200000	21.24250000	0.00000000
193	3173.96880000	40.19030000	0.00000000
194	3177.45600000	24.00810000	0.00000000
195	3183.43170000	32.93520000	0.00000000
196	3186.69630000	52.44330000	0.00000000
197	3194.20620000	9.89770000	0.00000000
198	3205.44570000	9.83060000	0.00000000

S11.8. LITHIATED INTERMEDIATE FROM 2a WITH COORDINATED CHLOROFORMATE

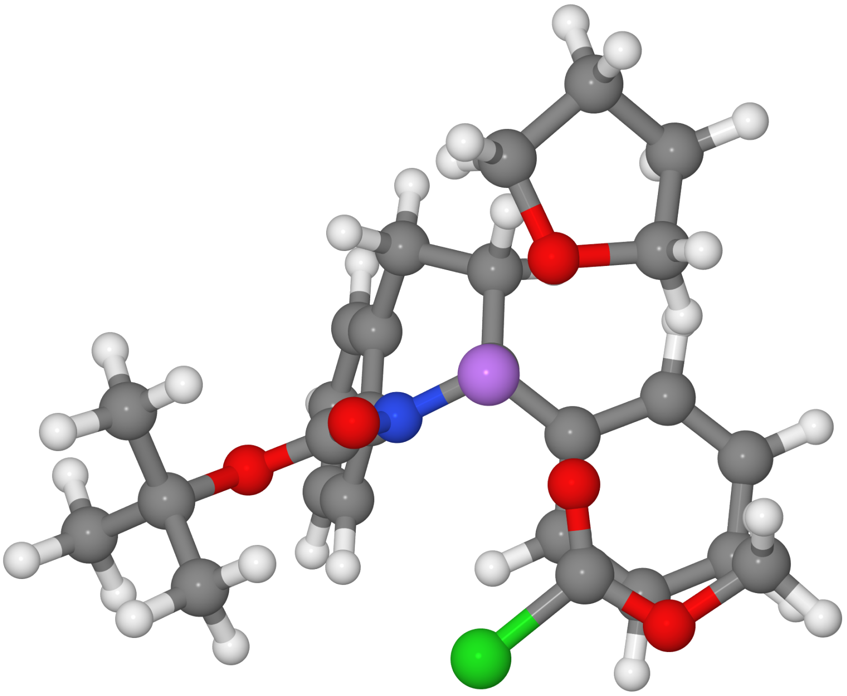

Route	: # opt=tight freq b3lyp/6-311g(d,p) scrf=(solvent=thf) geom=connectivity empiricaldispersion=gd3bj int=superfinegrid pop=(regular,mk) scf=tight nosymm	
SMILES	: [Li].CC(C)(C)OC(=O)N1c2ccccc2CC[C]1c3ccccc3.COC(=O)Cl.COC(=O)Cl	
Formula	: $C_{24}H_{28}Cl_2LiNO_6$	
Charge	: 0	
Multiplicity	: 1	
Dipole	: 16.4584	Debye
Energy	: -2366.12483791	a.u.
Gibbs Energy	: -2365.71579800	a.u.
Number of imaginary frequencies	: 0	

S11.8.1. Cartesian Co-ordinates (XYZ format)

62

C	-3.96777892	-0.42875001	-3.56802392
C	-3.70955205	-1.55750704	-2.79165101
C	-2.79462600	-1.49335694	-1.74146497
C	-2.15748501	-0.28691900	-1.46910405
C	-2.41047502	0.85776800	-2.23781300
C	-3.31418109	0.77385300	-3.29348207
N	-1.13914597	-0.12018700	-0.48144999
C	0.12366300	0.45908299	-0.97834200
C	-0.12859900	1.75537205	-1.75643301
C	-1.63607502	2.08684111	-1.85184896
C	-1.35114896	-0.27000001	0.84477502
O	-0.49243701	-0.00008500	1.69911206
O	-2.57136512	-0.73101801	1.13712895

C	-2.99705505	-0.99374902	2.52792811
C	-2.98473001	0.30562600	3.33170009
C	-2.11319089	-2.07458591	3.14692307
C	-4.42391777	-1.49962902	2.33693004
C	1.00408196	-0.52125901	-1.57168698
Li	1.17486703	0.61884201	0.97769201
C	0.89244002	-1.91535103	-1.31141102
C	1.78470600	-2.83716106	-1.84130299
C	2.83844495	-2.43549109	-2.66686702
C	2.97568488	-1.07103097	-2.93571305
C	2.09550595	-0.13802101	-2.40201807
H	-4.67318010	-0.48554599	-4.38899088
H	-4.21277094	-2.49268198	-3.00873590
H	-2.58192110	-2.36347103	-1.13464797
H	-3.51087809	1.65180302	-3.89975309
H	0.25059301	1.65333605	-2.78181410
H	0.38824201	2.63042808	-1.34055603
H	-1.80959105	2.90139198	-2.55790091
H	-1.98963296	2.43041801	-0.87186402
H	-3.45052505	0.13242801	4.30470705
H	-3.55908203	1.07527995	2.81036901
H	-1.96810102	0.66115201	3.48600292
H	-2.52161193	-2.36104989	4.11901712
H	-2.09847808	-2.96035600	2.50745797
H	-1.09411395	-1.71886802	3.28183007
H	-4.86703110	-1.73607504	3.30631399
H	-4.42979097	-2.40034795	1.72016299
H	-5.03611183	-0.73954600	1.84773397
H	0.08556800	-2.26675010	-0.68086803
H	1.65419304	-3.88922811	-1.60694098
H	3.53245497	-3.15858889	-3.07820296
H	3.78796506	-0.72567201	-3.56861091
H	2.25665402	0.91205001	-2.61529899
C	2.81519103	-1.80946505	1.29401004
O	2.65081692	-0.62017202	1.24831104
O	3.83375907	-2.48840404	0.84828401
C	4.85205221	-1.70349598	0.14972000
H	4.38740015	-1.19099903	-0.68888301
H	5.30113077	-0.99956101	0.84822601
H	5.57532310	-2.43750000	-0.18943299
O	1.68924797	2.50099897	1.20252502
C	1.34957099	3.61316490	0.90678197
O	0.20785999	4.20141220	1.14668798
C	-0.77419698	3.41029406	1.88787997
H	-0.94924700	2.46879196	1.37545800
H	-0.40014401	3.22976899	2.89417100
H	-1.66573203	4.02786112	1.90478301
Cl	1.65127099	-2.89039397	2.06781912
Cl	2.42787910	4.71313381	0.04318800

S11.8.2. Frequencies

Mode	IR frequency	IR intensity	Raman intensity
1	3.15620000	0.08760000	0.00000000
2	10.01550000	2.01430000	0.00000000
3	24.01110000	0.05120000	0.00000000
4	31.52490000	1.80690000	0.00000000
5	33.96480000	0.57150000	0.00000000
6	34.23520000	0.57780000	0.00000000
7	36.59010000	1.19300000	0.00000000
8	41.03600000	0.92460000	0.00000000
9	48.66540000	2.78900000	0.00000000
10	56.63960000	2.51520000	0.00000000
11	61.47620000	1.08690000	0.00000000
12	71.72280000	2.36960000	0.00000000
13	76.54290000	1.00370000	0.00000000
14	81.25830000	2.28610000	0.00000000
15	86.74910000	0.80840000	0.00000000
16	90.61920000	0.87120000	0.00000000
17	98.44830000	0.36000000	0.00000000
18	116.92230000	1.48210000	0.00000000
19	127.70490000	1.91000000	0.00000000
20	130.93360000	0.54480000	0.00000000
21	148.89110000	3.03310000	0.00000000
22	153.88500000	2.58220000	0.00000000
23	166.43770000	2.31580000	0.00000000
24	169.33800000	1.61990000	0.00000000
25	175.29480000	3.90880000	0.00000000
26	185.54800000	6.01560000	0.00000000
27	207.85860000	1.21730000	0.00000000
28	209.80710000	0.55730000	0.00000000
29	222.74650000	8.12950000	0.00000000
30	246.86830000	0.09260000	0.00000000
31	261.96830000	5.10140000	0.00000000
32	265.92830000	11.76080000	0.00000000
33	269.77870000	0.54100000	0.00000000
34	272.16290000	4.40190000	0.00000000
35	274.26190000	0.13880000	0.00000000
36	312.22320000	4.80380000	0.00000000
37	327.67040000	0.25310000	0.00000000
38	350.28600000	2.09140000	0.00000000
39	356.97660000	30.06380000	0.00000000
40	386.43670000	71.95010000	0.00000000
41	395.54030000	11.01570000	0.00000000
42	401.18390000	22.72650000	0.00000000
43	422.43410000	28.81000000	0.00000000
44	427.62330000	16.44570000	0.00000000
45	429.98280000	17.76510000	0.00000000
46	440.84300000	141.71110000	0.00000000
47	451.00540000	24.74520000	0.00000000
48	456.74550000	6.66390000	0.00000000
49	462.84210000	61.00660000	0.00000000
50	477.16850000	9.73900000	0.00000000
51	481.69500000	25.80860000	0.00000000
52	492.96700000	82.65700000	0.00000000
53	501.12050000	31.91310000	0.00000000
54	522.52320000	103.12160000	0.00000000
55	538.90330000	6.00870000	0.00000000
56	556.22230000	62.76580000	0.00000000
57	575.96510000	10.08130000	0.00000000
58	603.09960000	38.14570000	0.00000000
59	635.35620000	0.11210000	0.00000000
60	647.35840000	0.93430000	0.00000000

61	679.08250000	60.92800000	0.00000000
62	687.22130000	52.73350000	0.00000000
63	691.19060000	53.52200000	0.00000000
64	710.49870000	64.19320000	0.00000000
65	728.31160000	24.02640000	0.00000000
66	752.61240000	77.08620000	0.00000000
67	754.87000000	38.00090000	0.00000000
68	769.85000000	7.06810000	0.00000000
69	779.46370000	27.91440000	0.00000000
70	789.61490000	19.02350000	0.00000000
71	810.07320000	3.33270000	0.00000000
72	827.68850000	5.37630000	0.00000000
73	836.93180000	119.76120000	0.00000000
74	842.07150000	16.26540000	0.00000000
75	845.32840000	184.31310000	0.00000000
76	861.52440000	22.71470000	0.00000000
77	875.52890000	5.82140000	0.00000000
78	894.42910000	31.40490000	0.00000000
79	903.41980000	20.14690000	0.00000000
80	928.25620000	0.21410000	0.00000000
81	931.39960000	0.86560000	0.00000000
82	941.35220000	1.81420000	0.00000000
83	942.18520000	2.75760000	0.00000000
84	947.45510000	3.57210000	0.00000000
85	952.51830000	7.65740000	0.00000000
86	964.67130000	0.52960000	0.00000000
87	972.05010000	0.31770000	0.00000000
88	975.26050000	0.79400000	0.00000000
89	986.68690000	0.80620000	0.00000000
90	990.81010000	75.71160000	0.00000000
91	1009.83840000	37.75350000	0.00000000
92	1042.08760000	24.68780000	0.00000000
93	1045.32650000	15.59000000	0.00000000
94	1051.19140000	0.82820000	0.00000000
95	1055.45400000	6.40400000	0.00000000
96	1060.46120000	4.55520000	0.00000000
97	1071.56400000	71.72690000	0.00000000
98	1103.14620000	39.10640000	0.00000000
99	1132.96790000	101.56030000	0.00000000
100	1151.25280000	138.22910000	0.00000000
101	1165.63430000	24.15390000	0.00000000
102	1169.33110000	3.08510000	0.00000000
103	1171.78090000	2.24140000	0.00000000
104	1174.85070000	0.47280000	0.00000000
105	1179.05510000	384.09920000	0.00000000
106	1184.33070000	318.99260000	0.00000000
107	1187.46720000	253.27890000	0.00000000
108	1191.11870000	182.20150000	0.00000000
109	1202.73380000	47.23600000	0.00000000
110	1209.53310000	8.32320000	0.00000000
111	1224.74320000	11.58700000	0.00000000
112	1229.93100000	317.95960000	0.00000000
113	1238.95190000	440.95480000	0.00000000
114	1268.38770000	25.18380000	0.00000000
115	1276.68420000	244.26090000	0.00000000
116	1280.93210000	7.08130000	0.00000000
117	1306.15030000	70.67540000	0.00000000
118	1311.77880000	34.11890000	0.00000000
119	1320.38180000	43.81560000	0.00000000
120	1340.65530000	29.79800000	0.00000000
121	1342.84650000	24.47150000	0.00000000
122	1360.11590000	9.18850000	0.00000000
123	1369.35640000	25.13870000	0.00000000
124	1394.04820000	30.42480000	0.00000000

125	1397.56970000	42.23400000	0.00000000
126	1419.74710000	88.97750000	0.00000000
127	1422.09460000	674.19790000	0.00000000
128	1466.37470000	0.14740000	0.00000000
129	1470.91640000	44.21690000	0.00000000
130	1471.87780000	29.18250000	0.00000000
131	1472.25210000	7.83190000	0.00000000
132	1477.30770000	12.82250000	0.00000000
133	1477.34690000	12.94190000	0.00000000
134	1481.83160000	15.32480000	0.00000000
135	1481.85930000	3.87020000	0.00000000
136	1482.30470000	8.39140000	0.00000000
137	1485.03220000	4.61880000	0.00000000
138	1485.92700000	8.34100000	0.00000000
139	1487.05350000	50.63940000	0.00000000
140	1491.32790000	20.11100000	0.00000000
141	1500.64060000	0.50540000	0.00000000
142	1505.68510000	68.71560000	0.00000000
143	1511.71600000	146.34190000	0.00000000
144	1519.79620000	23.09560000	0.00000000
145	1521.38220000	103.52520000	0.00000000
146	1576.40480000	19.27130000	0.00000000
147	1620.72110000	85.90000000	0.00000000
148	1628.78640000	284.46270000	0.00000000
149	1640.08500000	203.57170000	0.00000000
150	1660.55820000	556.06500000	0.00000000
151	1783.36790000	1259.92990000	0.00000000
152	1794.87990000	62.13430000	0.00000000
153	2972.88810000	236.05140000	0.00000000
154	2984.82360000	41.45870000	0.00000000
155	3010.35710000	122.36580000	0.00000000
156	3037.64950000	21.59530000	0.00000000
157	3038.62290000	28.36560000	0.00000000
158	3045.84840000	22.85160000	0.00000000
159	3073.07310000	38.79450000	0.00000000
160	3078.80420000	59.80370000	0.00000000
161	3080.09620000	21.03560000	0.00000000
162	3099.71930000	11.50960000	0.00000000
163	3101.85020000	33.88430000	0.00000000
164	3112.19830000	40.22750000	0.00000000
165	3116.15420000	66.97350000	0.00000000
166	3140.63340000	28.07580000	0.00000000
167	3144.34440000	9.10130000	0.00000000
168	3146.82140000	29.24520000	0.00000000
169	3149.46100000	18.83660000	0.00000000
170	3158.62720000	8.09990000	0.00000000
171	3168.26220000	3.04800000	0.00000000
172	3169.20070000	22.50010000	0.00000000
173	3170.83370000	44.63250000	0.00000000
174	3173.91950000	7.46630000	0.00000000
175	3178.73890000	54.91100000	0.00000000
176	3184.10220000	47.58180000	0.00000000
177	3185.49990000	14.99520000	0.00000000
178	3196.54900000	4.82680000	0.00000000
179	3201.38370000	13.11480000	0.00000000
180	3203.34780000	2.37340000	0.00000000

S11.9. LITHIATED INTERMEDIATE FROM 2a WITH COORDINATED CYANOFORMATE

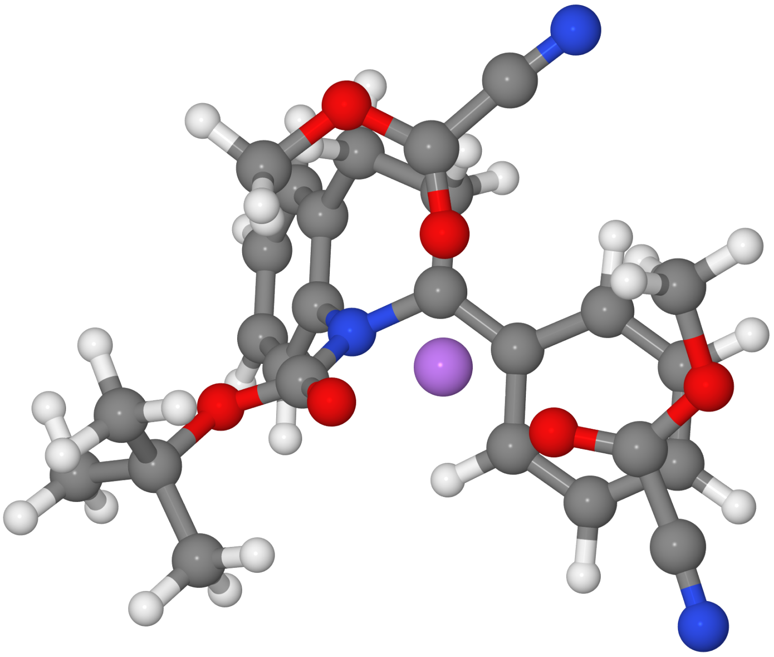

Route	:	# opt=tight freq b3lyp/6-311g(d,p) scrf=(solvent=thf) geom=connectivity empiricaldispersion=gd3bj int=superfinegrid pop=(regular,mk) scf=tight nosymm	
SMILES	:	[Li].CC(C)(C)OC(=O)N1c2ccccc2CC[C]1c3ccccc3.COC(=O)C#N.COC(=O)C#N	
Formula	:	$C_{26}H_{28}LiN_3O_6$	
Charge	:	0	
Multiplicity	:	1	
Dipole	:	25.1214	Deby
Energy	:	-1631.38251786	a.u.
Gibbs Energy	:	-1630.95441900	a.u.
Number of imaginary frequencies	:	1	

S11.9.1. Cartesian Co-ordinates (XYZ format)

64

C	-4.79495478	1.21578097	-2.76135492
C	-4.54556417	-0.15022600	-2.63872194
C	-3.46136999	-0.60138899	-1.88874698
C	-2.64368296	0.32633001	-1.25134802
C	-2.87947607	1.70134497	-1.36555398
C	-3.95870209	2.13674498	-2.13108706
N	-1.46335804	-0.03352600	-0.51679802
C	-0.27516499	0.70802897	-0.88535601
C	-0.46110100	2.18998408	-0.95036298
C	-1.91457200	2.60913205	-0.65265101
C	-1.47486198	-0.89336097	0.53723103
O	-0.46720001	-1.16434002	1.20091105
O	-2.68040204	-1.41384304	0.77035898
C	-2.91409802	-2.41282701	1.83865905
C	-2.61618900	-1.79291999	3.20252395

C	-2.08699012	-3.66696501	1.56675398
C	-4.40487289	-2.69903898	1.68633497
C	0.80513102	0.04007100	-1.48557603
Li	1.28278100	-0.48386899	1.13189101
C	0.85460299	-1.38249600	-1.67160904
C	1.96501303	-2.00512505	-2.20801091
C	3.10429907	-1.27796698	-2.58162689
C	3.07057691	0.11921800	-2.44355392
C	1.97358799	0.76311302	-1.90816903
H	-5.63418198	1.56322598	-3.35235691
H	-5.18819618	-0.86742502	-3.13566399
H	-3.25474095	-1.65842998	-1.79200196
H	-4.14482784	3.20051193	-2.23148489
H	-0.17627200	2.56925893	-1.94071400
H	0.20370500	2.73298502	-0.24908200
H	-2.07732010	3.65115309	-0.93145198
H	-2.09429598	2.53747010	0.42591000
H	-2.93564510	-2.48231101	3.98745799
H	-3.17255998	-0.86004800	3.32127690
H	-1.55325794	-1.59334195	3.32187009
H	-2.37292504	-4.44581223	2.27738190
H	-2.28386497	-4.03675222	0.55782801
H	-1.02216494	-3.46936107	1.67145300
H	-4.71901417	-3.43119597	2.43267894
H	-4.61793518	-3.09838700	0.69303000
H	-4.98522902	-1.78512299	1.82606399
H	-0.00643100	-1.97941899	-1.40034103
H	1.95663095	-3.08350205	-2.32908893
H	3.96161699	-1.77235401	-3.01897502
H	3.93149996	0.70732999	-2.74480510
H	1.99809694	1.83985102	-1.79336095
C	4.10189581	-1.25621700	0.21550700
O	3.01769710	-1.19197798	0.76323801
C	4.63062382	-2.51597309	-0.28911299
N	5.04591703	-3.55357695	-0.57786298
O	4.97984600	-0.27915499	0.09742500
C	4.58556414	1.01007605	0.64344698
H	3.66338611	1.33790803	0.17217600
H	4.45237494	0.92218000	1.72121096
H	5.40858603	1.67608094	0.40570700
O	1.32735503	1.33651602	1.65906000
C	1.02231097	2.50137091	1.85326803
C	1.84794796	3.59389091	1.38214195
O	-0.03976800	2.92329311	2.52946305
N	2.58590603	4.41105795	1.02847695
C	-0.89126301	1.88485098	3.08032107
H	-1.26311398	1.24512899	2.28336000
H	-0.33015800	1.29096699	3.80118108
H	-1.70373404	2.41679597	3.56463909

S11.9.2. Frequencies

Mode	IR frequency	IR intensity	Raman intensity
1	-16.18610000	0.86570000	0.00000000
2	12.43400000	1.35420000	0.00000000
3	22.93420000	0.80970000	0.00000000
4	28.60250000	0.38310000	0.00000000
5	32.94950000	3.98900000	0.00000000
6	34.87240000	0.35710000	0.00000000
7	45.52290000	2.92970000	0.00000000
8	55.60280000	0.72910000	0.00000000
9	65.81170000	2.00700000	0.00000000
10	68.63630000	1.83100000	0.00000000
11	74.81480000	0.59800000	0.00000000
12	86.31000000	4.23390000	0.00000000
13	87.06490000	18.73280000	0.00000000
14	96.27060000	1.99040000	0.00000000
15	101.12790000	8.50090000	0.00000000
16	101.92740000	16.32660000	0.00000000
17	112.81640000	0.76030000	0.00000000
18	114.33930000	4.80710000	0.00000000
19	120.84940000	15.96280000	0.00000000
20	136.00340000	10.40230000	0.00000000
21	140.22730000	2.28700000	0.00000000
22	153.44380000	10.25030000	0.00000000
23	158.31150000	18.90350000	0.00000000
24	168.71890000	0.17240000	0.00000000
25	171.99250000	9.04510000	0.00000000
26	180.78170000	1.32600000	0.00000000
27	194.38800000	3.83300000	0.00000000
28	195.92440000	12.88880000	0.00000000
29	211.87640000	0.45430000	0.00000000
30	214.27610000	7.76190000	0.00000000
31	225.96920000	42.60510000	0.00000000
32	229.30330000	17.66620000	0.00000000
33	249.37360000	2.27460000	0.00000000
34	264.21330000	20.05550000	0.00000000
35	271.89160000	0.17740000	0.00000000
36	277.14420000	10.67590000	0.00000000
37	305.41510000	23.94250000	0.00000000
38	309.35410000	56.03990000	0.00000000
39	319.26340000	8.34530000	0.00000000
40	330.90660000	11.20150000	0.00000000
41	342.08230000	57.68090000	0.00000000
42	350.99340000	4.47790000	0.00000000
43	352.42350000	48.02660000	0.00000000
44	361.01310000	12.83390000	0.00000000
45	400.54850000	1.58710000	0.00000000
46	414.48740000	14.91960000	0.00000000
47	427.74600000	8.10880000	0.00000000
48	430.79000000	2.01550000	0.00000000
49	433.31350000	1.59180000	0.00000000
50	455.83620000	12.71310000	0.00000000
51	457.37270000	4.68680000	0.00000000
52	480.42060000	12.68550000	0.00000000
53	483.98970000	38.31590000	0.00000000
54	516.25870000	89.75290000	0.00000000
55	537.71040000	11.15150000	0.00000000
56	544.02210000	6.99970000	0.00000000
57	546.56530000	4.57850000	0.00000000
58	556.18370000	109.10550000	0.00000000
59	565.15370000	12.36460000	0.00000000
60	599.31150000	33.83060000	0.00000000

61	618.22470000	29.46830000	0.00000000
62	618.43490000	17.46800000	0.00000000
63	630.70540000	1.35930000	0.00000000
64	643.57860000	2.92040000	0.00000000
65	679.11600000	11.64680000	0.00000000
66	688.45170000	40.91760000	0.00000000
67	698.97800000	42.95190000	0.00000000
68	711.72650000	82.35070000	0.00000000
69	728.69140000	11.91210000	0.00000000
70	750.95530000	66.22290000	0.00000000
71	754.52530000	36.55890000	0.00000000
72	760.56980000	21.51220000	0.00000000
73	772.85810000	47.53910000	0.00000000
74	791.35890000	15.61810000	0.00000000
75	805.64260000	0.99630000	0.00000000
76	813.23280000	3.95220000	0.00000000
77	844.05030000	34.42000000	0.00000000
78	846.03390000	28.00620000	0.00000000
79	874.82150000	159.24340000	0.00000000
80	879.53430000	73.77850000	0.00000000
81	883.49490000	80.03090000	0.00000000
82	893.74170000	30.25600000	0.00000000
83	912.48590000	61.34820000	0.00000000
84	929.05060000	0.16900000	0.00000000
85	931.70510000	3.36600000	0.00000000
86	953.72270000	0.61540000	0.00000000
87	966.90200000	20.04830000	0.00000000
88	968.35920000	5.28320000	0.00000000
89	971.87330000	0.08320000	0.00000000
90	974.57990000	28.56430000	0.00000000
91	986.54060000	93.48800000	0.00000000
92	988.09450000	121.64340000	0.00000000
93	991.00440000	0.61250000	0.00000000
94	993.63590000	23.04430000	0.00000000
95	1021.38640000	40.70300000	0.00000000
96	1035.24270000	32.82190000	0.00000000
97	1041.53780000	45.68010000	0.00000000
98	1051.28850000	0.86280000	0.00000000
99	1056.80280000	4.60810000	0.00000000
100	1061.53100000	24.84460000	0.00000000
101	1085.13500000	91.51750000	0.00000000
102	1107.52310000	53.48240000	0.00000000
103	1137.88740000	136.75570000	0.00000000
104	1167.77870000	9.21790000	0.00000000
105	1168.85400000	2.63990000	0.00000000
106	1169.87120000	49.04480000	0.00000000
107	1174.23460000	64.03240000	0.00000000
108	1177.45190000	6.70170000	0.00000000
109	1185.81640000	440.56090000	0.00000000
110	1194.38760000	1282.97210000	0.00000000
111	1203.46800000	153.75800000	0.00000000
112	1207.51960000	8.83870000	0.00000000
113	1210.63450000	30.10210000	0.00000000
114	1213.38480000	621.03960000	0.00000000
115	1219.78270000	405.25740000	0.00000000
116	1248.42220000	167.07590000	0.00000000
117	1255.91290000	931.98190000	0.00000000
118	1269.30850000	25.85060000	0.00000000
119	1277.74860000	240.81500000	0.00000000
120	1280.22050000	40.45070000	0.00000000
121	1308.37400000	14.11650000	0.00000000
122	1322.29240000	20.70500000	0.00000000
123	1335.73240000	14.65200000	0.00000000
124	1337.09240000	3.69550000	0.00000000

125	1366.25390000	13.18530000	0.00000000
126	1372.62250000	27.44970000	0.00000000
127	1395.42420000	67.18580000	0.00000000
128	1396.82970000	276.89320000	0.00000000
129	1403.28890000	567.96120000	0.00000000
130	1421.92650000	23.69930000	0.00000000
131	1427.03890000	207.23170000	0.00000000
132	1467.64140000	5.70700000	0.00000000
133	1468.90220000	24.04460000	0.00000000
134	1471.19840000	17.78110000	0.00000000
135	1475.28200000	30.96120000	0.00000000
136	1476.96720000	29.39720000	0.00000000
137	1477.23660000	14.64260000	0.00000000
138	1482.80300000	4.83360000	0.00000000
139	1484.82530000	2.61970000	0.00000000
140	1485.29140000	1.50260000	0.00000000
141	1486.40330000	3.09030000	0.00000000
142	1487.51690000	35.83470000	0.00000000
143	1489.16230000	20.53230000	0.00000000
144	1489.61250000	43.69510000	0.00000000
145	1494.12430000	8.98640000	0.00000000
146	1499.51970000	0.72300000	0.00000000
147	1518.13820000	214.05740000	0.00000000
148	1519.67630000	68.88230000	0.00000000
149	1523.21120000	301.29750000	0.00000000
150	1553.66240000	39.24060000	0.00000000
151	1621.26490000	660.55900000	0.00000000
152	1624.64470000	45.84110000	0.00000000
153	1644.50470000	58.57510000	0.00000000
154	1663.36970000	170.29650000	0.00000000
155	1698.53050000	460.67870000	0.00000000
156	1725.06030000	462.70100000	0.00000000
157	2290.47340000	1371.30450000	0.00000000
158	2320.05230000	577.56030000	0.00000000
159	2854.46470000	904.97090000	0.00000000
160	2985.45310000	80.97400000	0.00000000
161	3025.98620000	55.65100000	0.00000000
162	3039.20030000	16.30700000	0.00000000
163	3040.50930000	28.60320000	0.00000000
164	3047.22780000	20.37980000	0.00000000
165	3070.75550000	35.46740000	0.00000000
166	3072.67850000	21.97250000	0.00000000
167	3091.65500000	49.43260000	0.00000000
168	3102.16410000	12.22800000	0.00000000
169	3104.29450000	26.63810000	0.00000000
170	3113.85110000	38.41080000	0.00000000
171	3117.80160000	60.76930000	0.00000000
172	3142.63610000	1.61900000	0.00000000
173	3145.63270000	31.74870000	0.00000000
174	3151.86720000	28.45320000	0.00000000
175	3155.40430000	22.87750000	0.00000000
176	3155.51060000	14.97240000	0.00000000
177	3163.35690000	5.91180000	0.00000000
178	3164.49880000	16.51690000	0.00000000
179	3173.80110000	20.34240000	0.00000000
180	3179.74950000	19.93900000	0.00000000
181	3187.01320000	38.79360000	0.00000000
182	3188.15570000	50.54350000	0.00000000
183	3190.01470000	10.72990000	0.00000000
184	3195.41170000	13.26550000	0.00000000
185	3197.09560000	11.40660000	0.00000000
186	3207.96580000	9.03770000	0.00000000

S11.10. LITHIATED INTERMEDIATE FROM 2a WITH COORDINATED THF

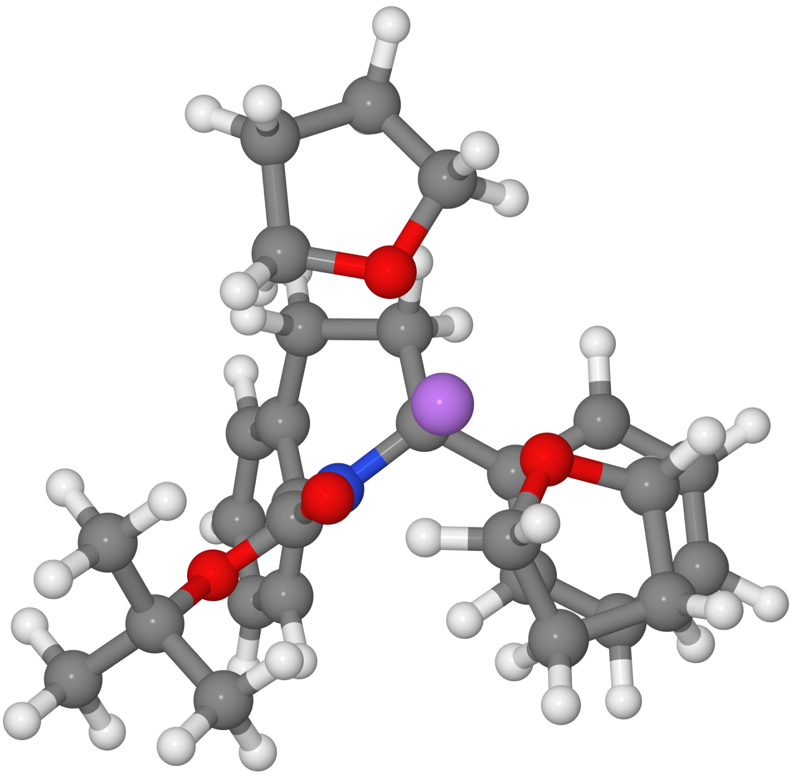

Route	: # opt=(tight,maxstep=1) freq b3lyp/6-311g(d,p) scrf=(solvent=thf) nosymm geom=connectivity empiricaldispersion=gd3bj int=superfinegrid pop=(regular,mk) scf=tight
SMILES	: [Li].CC(C)(C)OC(=O)N1c2ccccc2CC[C]1c3ccccc3.C1CCOC1.C1CCOC1
Formula	: $C_{28}H_{38}LiNO_4$
Charge	: 0
Multiplicity	: 1
Dipole	: 20.5998 Debye
Energy	: -1453.65977201 a.u.
Gibbs Energy	: -1453.11957300 a.u.
Number of imaginary frequencies	: 0

S11.10.1. Cartesian Co-ordinates (XYZ format)

```
72

C  -4.63538599 -2.22831988 -1.38085997
C  -4.16422987 -2.49609995 -0.09631300
C  -3.00132203 -1.88278306  0.36761999
C  -2.32647204 -0.98941702 -0.45942801
C  -2.79041409 -0.70814902 -1.75259805
C  -3.94430709 -1.34086096 -2.20742297
N  -1.08505404 -0.36777201 -0.12826100
C  -0.00938200 -0.55208701 -1.11790395
C  -0.47513199 -0.21268401 -2.53456402
C  -1.95042801  0.24522200 -2.55538702
C  -0.91607600  0.46474299  0.92306602
```

O	0.14699300	1.05781901	1.14921105
O	-2.00911689	0.57618099	1.69028103
C	-2.00898790	1.37354302	2.93219805
C	-1.75256503	2.84547496	2.61173201
C	-0.99006099	0.80192202	3.91694593
C	-3.43155193	1.17091703	3.44638205
C	0.76799101	-1.74411595	-0.91736501
Li	1.49403501	0.86572599	-0.24615100
C	0.81540000	-2.43646312	0.32846299
C	1.63554502	-3.53652310	0.52735502
C	2.46336293	-4.02282906	-0.49067399
C	2.43240190	-3.36932302	-1.72532594
C	1.61431503	-2.26709509	-1.93980706
H	-5.53560877	-2.71327305	-1.74041700
H	-4.69682693	-3.19020700	0.54369700
H	-2.62463403	-2.08458900	1.36152005
H	-4.30584908	-1.13581598	-3.20964408
H	-0.39938799	-1.10182095	-3.17711306
H	0.13365400	0.56008202	-3.02120900
H	-2.31802893	0.32754701	-3.58043504
H	-2.02001810	1.24333203	-2.10520291
H	-1.90534902	3.44341898	3.51349306
H	-2.45450711	3.19129491	1.84911895
H	-0.73663503	2.99685192	2.25358009
H	-1.09425104	1.30822003	4.87950993
H	-1.17235994	-0.26447201	4.06994009
H	0.02658500	0.93933100	3.55455899
H	-3.57107496	1.71834099	4.38073921
H	-3.62403893	0.11188400	3.62883496
H	-4.15642023	1.53474295	2.71561098
H	0.19367200	-2.08858991	1.14427495
H	1.63166797	-4.02586603	1.49703705
H	3.10599709	-4.87952995	-0.32695699
H	3.06019497	-3.72366810	-2.53779197
H	1.63958395	-1.77928305	-2.90722704
O	3.18300796	0.51590103	0.66282701
C	3.96593094	-0.69473398	0.46882099
C	3.17578697	0.83250600	2.06995106
C	4.14573097	-1.32623100	1.86191297
H	3.40792394	-1.33515704	-0.21052200
H	4.92156982	-0.42215601	0.01297700
C	3.17744994	-0.52783900	2.75114202
H	4.07467079	1.40957499	2.31949592
H	2.28840804	1.43177497	2.26403594
H	5.17266417	-1.19974804	2.21260095
H	3.91516805	-2.39041805	1.84491599
H	3.49649405	-0.47860199	3.79344392
H	2.17442894	-0.95790601	2.70723510
O	1.50546002	2.56099606	-1.19047797
C	0.31936899	3.39053106	-1.10402596
C	2.12294793	2.70780897	-2.49235797
C	0.38188300	4.31889105	-2.31254792
H	-0.55899298	2.74038696	-1.13769102
H	0.33690801	3.90888309	-0.14550400
C	1.10134399	3.44787598	-3.35108209
H	3.04581404	3.28385806	-2.37568498
H	2.37026191	1.71290600	-2.86750197
H	0.97730601	5.20658779	-2.08318210
H	-0.60840201	4.64226007	-2.63509607
H	1.57211304	4.02508688	-4.14753389
H	0.40097299	2.74149489	-3.80392098

S11.10.2. Frequencies

Mode	IR frequency	IR intensity	Raman intensity
1	5.78420000	0.43930000	0.00000000
2	9.93680000	0.21900000	0.00000000
3	16.33320000	1.34030000	0.00000000
4	25.50910000	1.61110000	0.00000000
5	33.83060000	0.76470000	0.00000000
6	35.01620000	0.04930000	0.00000000
7	42.43430000	1.46390000	0.00000000
8	49.15330000	0.59730000	0.00000000
9	56.01810000	2.60520000	0.00000000
10	65.17910000	0.69990000	0.00000000
11	69.95380000	1.74650000	0.00000000
12	75.76680000	1.60090000	0.00000000
13	80.62430000	1.79270000	0.00000000
14	91.88480000	1.77820000	0.00000000
15	93.10030000	0.83530000	0.00000000
16	99.66720000	0.72410000	0.00000000
17	112.64070000	2.24470000	0.00000000
18	115.35520000	1.55030000	0.00000000
19	128.42190000	5.26230000	0.00000000
20	133.88000000	0.94500000	0.00000000
21	146.21790000	11.96800000	0.00000000
22	162.33100000	3.34190000	0.00000000
23	169.52350000	1.07170000	0.00000000
24	175.07440000	3.38430000	0.00000000
25	205.50570000	2.26980000	0.00000000
26	208.81320000	0.46610000	0.00000000
27	223.49680000	8.62240000	0.00000000
28	241.99110000	0.01140000	0.00000000
29	259.62120000	0.35430000	0.00000000
30	262.72740000	5.49760000	0.00000000
31	269.63820000	0.45380000	0.00000000
32	273.11410000	0.95810000	0.00000000
33	296.04410000	5.37150000	0.00000000
34	322.16720000	2.06320000	0.00000000
35	330.54970000	1.03580000	0.00000000
36	350.58000000	2.77590000	0.00000000
37	368.43110000	3.19610000	0.00000000
38	400.42330000	70.32540000	0.00000000
39	419.75530000	19.24030000	0.00000000
40	426.84720000	8.29460000	0.00000000
41	427.98210000	34.93280000	0.00000000
42	441.36430000	16.54890000	0.00000000
43	454.88820000	16.36530000	0.00000000
44	459.04370000	12.55110000	0.00000000
45	466.87840000	109.06380000	0.00000000
46	481.49270000	23.69220000	0.00000000
47	494.56560000	62.91690000	0.00000000
48	504.73490000	136.54680000	0.00000000
49	537.87930000	13.85270000	0.00000000
50	545.32930000	62.61690000	0.00000000
51	573.94000000	4.63330000	0.00000000
52	580.90730000	1.38570000	0.00000000
53	589.98680000	6.00390000	0.00000000
54	599.32510000	35.77760000	0.00000000
55	634.36400000	0.14270000	0.00000000
56	644.48710000	0.51850000	0.00000000
57	686.70080000	12.61780000	0.00000000
58	689.17770000	36.35030000	0.00000000
59	694.54310000	24.25070000	0.00000000
60	706.43500000	66.79950000	0.00000000

61	728.23660000	21.85120000	0.00000000
62	745.11740000	76.56590000	0.00000000
63	753.85620000	35.53420000	0.00000000
64	769.59450000	5.30970000	0.00000000
65	776.09010000	31.17350000	0.00000000
66	788.67150000	11.71340000	0.00000000
67	808.11150000	3.69870000	0.00000000
68	819.57680000	4.74520000	0.00000000
69	822.22930000	3.62200000	0.00000000
70	843.75160000	17.43360000	0.00000000
71	849.28930000	30.57790000	0.00000000
72	851.27330000	16.40940000	0.00000000
73	873.91280000	2.48810000	0.00000000
74	876.68730000	14.71600000	0.00000000
75	878.83590000	26.86060000	0.00000000
76	890.55310000	105.29020000	0.00000000
77	894.48170000	30.21370000	0.00000000
78	897.72110000	17.12730000	0.00000000
79	905.40270000	22.91480000	0.00000000
80	916.56130000	12.32700000	0.00000000
81	919.64160000	15.42920000	0.00000000
82	927.93960000	0.65990000	0.00000000
83	928.52050000	10.08230000	0.00000000
84	930.08800000	1.53640000	0.00000000
85	931.84140000	5.94750000	0.00000000
86	946.45620000	0.88070000	0.00000000
87	954.06190000	9.81340000	0.00000000
88	961.26730000	0.26650000	0.00000000
89	969.63470000	0.92940000	0.00000000
90	970.02760000	0.17460000	0.00000000
91	971.14260000	1.89050000	0.00000000
92	971.89800000	0.39840000	0.00000000
93	984.71600000	0.56210000	0.00000000
94	988.45680000	72.14420000	0.00000000
95	1011.36420000	42.10980000	0.00000000
96	1040.88190000	10.78750000	0.00000000
97	1041.95270000	19.95160000	0.00000000
98	1042.53380000	3.81020000	0.00000000
99	1044.36830000	25.76020000	0.00000000
100	1050.59450000	0.79270000	0.00000000
101	1051.95180000	95.74060000	0.00000000
102	1054.96820000	5.26690000	0.00000000
103	1057.55650000	159.24020000	0.00000000
104	1061.04320000	11.27660000	0.00000000
105	1073.26310000	78.66340000	0.00000000
106	1101.82600000	45.90400000	0.00000000
107	1132.93190000	115.83670000	0.00000000
108	1154.18220000	101.63860000	0.00000000
109	1158.84980000	1.82580000	0.00000000
110	1167.59570000	0.78310000	0.00000000
111	1168.61380000	6.83400000	0.00000000
112	1174.37110000	0.12090000	0.00000000
113	1186.81610000	123.46170000	0.00000000
114	1190.50990000	10.62880000	0.00000000
115	1192.70140000	240.88780000	0.00000000
116	1194.26070000	19.72880000	0.00000000
117	1194.83220000	7.59670000	0.00000000
118	1202.51260000	49.45740000	0.00000000
119	1210.75420000	4.58120000	0.00000000
120	1226.42600000	6.10830000	0.00000000
121	1228.78100000	8.78270000	0.00000000
122	1252.75980000	4.50720000	0.00000000
123	1261.72210000	1.66920000	0.00000000
124	1265.18760000	7.99010000	0.00000000

125	1267.89850000	25.52580000	0.00000000
126	1276.67930000	224.31330000	0.00000000
127	1279.71260000	21.01370000	0.00000000
128	1279.86530000	9.08680000	0.00000000
129	1308.03700000	40.32120000	0.00000000
130	1311.25480000	35.96200000	0.00000000
131	1323.02090000	2.49670000	0.00000000
132	1323.62810000	2.58600000	0.00000000
133	1327.50630000	13.83060000	0.00000000
134	1334.06600000	1.48590000	0.00000000
135	1341.25220000	2.32190000	0.00000000
136	1345.75990000	2.48950000	0.00000000
137	1348.98860000	98.66320000	0.00000000
138	1359.95550000	12.69200000	0.00000000
139	1367.75590000	1.96710000	0.00000000
140	1369.76570000	20.00530000	0.00000000
141	1370.05360000	16.35610000	0.00000000
142	1393.34940000	28.13500000	0.00000000
143	1396.10920000	41.27410000	0.00000000
144	1399.81460000	5.78090000	0.00000000
145	1401.00900000	3.72420000	0.00000000
146	1418.22880000	430.62680000	0.00000000
147	1420.31200000	307.15830000	0.00000000
148	1466.47970000	0.36850000	0.00000000
149	1471.61460000	4.73260000	0.00000000
150	1476.18740000	7.67530000	0.00000000
151	1481.40370000	5.36250000	0.00000000
152	1484.41880000	3.08280000	0.00000000
153	1485.10950000	9.53980000	0.00000000
154	1485.29400000	1.09130000	0.00000000
155	1486.64760000	39.96220000	0.00000000
156	1490.57190000	27.25050000	0.00000000
157	1494.05260000	17.06730000	0.00000000
158	1495.20510000	15.23600000	0.00000000
159	1499.25930000	1.38810000	0.00000000
160	1503.15920000	50.27680000	0.00000000
161	1508.30950000	17.94440000	0.00000000
162	1511.08270000	191.27020000	0.00000000
163	1517.31640000	5.16180000	0.00000000
164	1518.83490000	9.64790000	0.00000000
165	1520.84610000	93.00810000	0.00000000
166	1524.70940000	11.61580000	0.00000000
167	1528.13610000	0.07850000	0.00000000
168	1570.34140000	20.34170000	0.00000000
169	1620.22300000	90.18540000	0.00000000
170	1628.46460000	307.13330000	0.00000000
171	1641.11910000	121.67340000	0.00000000
172	1669.56360000	697.86850000	0.00000000
173	2961.00730000	98.03250000	0.00000000
174	2983.88190000	105.51700000	0.00000000
175	3004.46020000	93.90670000	0.00000000
176	3007.54570000	130.58680000	0.00000000
177	3029.89520000	88.56620000	0.00000000
178	3037.14440000	18.94390000	0.00000000
179	3037.46000000	68.25370000	0.00000000
180	3038.36370000	30.87930000	0.00000000
181	3044.74070000	30.51960000	0.00000000
182	3045.51700000	27.60150000	0.00000000
183	3048.05180000	32.81570000	0.00000000
184	3053.08440000	30.86980000	0.00000000
185	3055.29750000	85.46050000	0.00000000
186	3057.77190000	23.42560000	0.00000000
187	3075.77230000	66.14460000	0.00000000
188	3083.07560000	46.75430000	0.00000000

189	3099.32100000	13.48750000	0.00000000
190	3101.56840000	30.20900000	0.00000000
191	3101.82730000	23.74770000	0.00000000
192	3105.76260000	42.67040000	0.00000000
193	3106.93230000	28.44070000	0.00000000
194	3111.46900000	43.57880000	0.00000000
195	3114.77090000	72.28170000	0.00000000
196	3115.28320000	76.51590000	0.00000000
197	3117.97200000	34.53660000	0.00000000
198	3131.38130000	17.49350000	0.00000000
199	3138.03880000	30.73500000	0.00000000
200	3140.89710000	6.77400000	0.00000000
201	3142.10930000	32.98960000	0.00000000
202	3143.79900000	1.60730000	0.00000000
203	3146.79170000	28.49940000	0.00000000
204	3157.60930000	9.05230000	0.00000000
205	3168.17530000	24.59380000	0.00000000
206	3168.70050000	47.10030000	0.00000000
207	3175.24060000	57.54060000	0.00000000
208	3182.32100000	20.62120000	0.00000000
209	3183.52440000	52.47800000	0.00000000
210	3201.81520000	11.80510000	0.00000000
